# The Versatile and Strategic *O*-Carbamate Directed Metalation Group in the Synthesis of Aromatic Molecules: An Update

**DOI:** 10.1021/acs.chemrev.3c00923

**Published:** 2024-06-12

**Authors:** Ross D. Jansen-van Vuuren, Susana Liu, M. A. Jalil Miah, Janez Cerkovnik, Janez Košmrlj, Victor Snieckus

**Affiliations:** † Department of Chemistry, Queen’s University, Chernoff Hall, 9 Bader Lane, Kingston, Ontario K7K 2N1, Canada; ‡ Department of Chemistry, Rajshahi University, Rajshahi-6205, Bangladesh; § Faculty of Chemistry and Chemical Technology, University of Ljubljana, Večna pot 113, 1000 Ljubljana, Slovenia

## Abstract

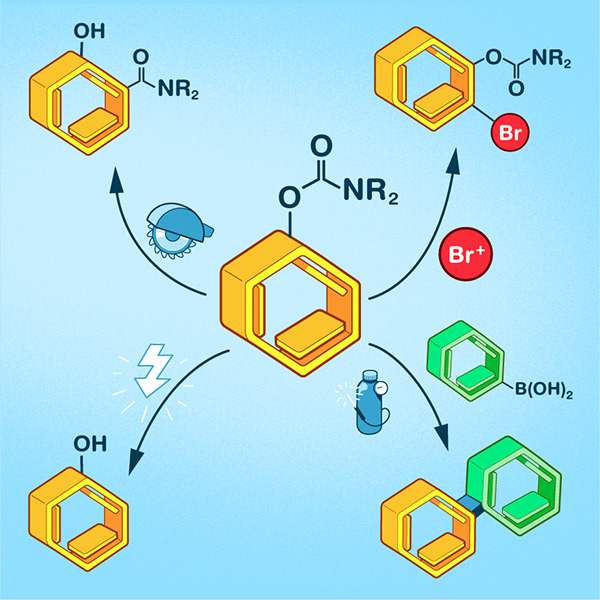

The aryl *O*-carbamate (ArOAm) group is among the strongest of the directed metalation groups (DMGs) in directed *ortho* metalation (D*o*M) chemistry, especially in the form Ar-OCONEt_2_. Since the last comprehensive review of metalation chemistry involving ArOAms (published more than 30 years ago), the field has expanded significantly. For example, it now encompasses new substrates, solvent systems, and metalating agents, while conditions have been developed enabling metalation of ArOAm to be conducted in a green and sustainable manner. The ArOAm group has also proven to be effective in the anionic *ortho*-Fries (A*o*F) rearrangement, Directed remote metalation (D*re*M), iterative D*o*M sequences, and D*o*M-halogen dance (HalD) synthetic strategies and has been transformed into a diverse range of functionalities and coupled with various groups through a range of cross-coupling (CC) strategies. Of ultimate value, the ArOAm group has demonstrated utility in the synthesis of a diverse range of bioactive and polycyclic aromatic compounds for various applications.

## 1 Introduction

### 1.1 The Aryl *O*-Carbamate (ArOAm): Significance of the Functional Group

#### 1.1.1 History of the Carbamate Group

**Figure 1:**
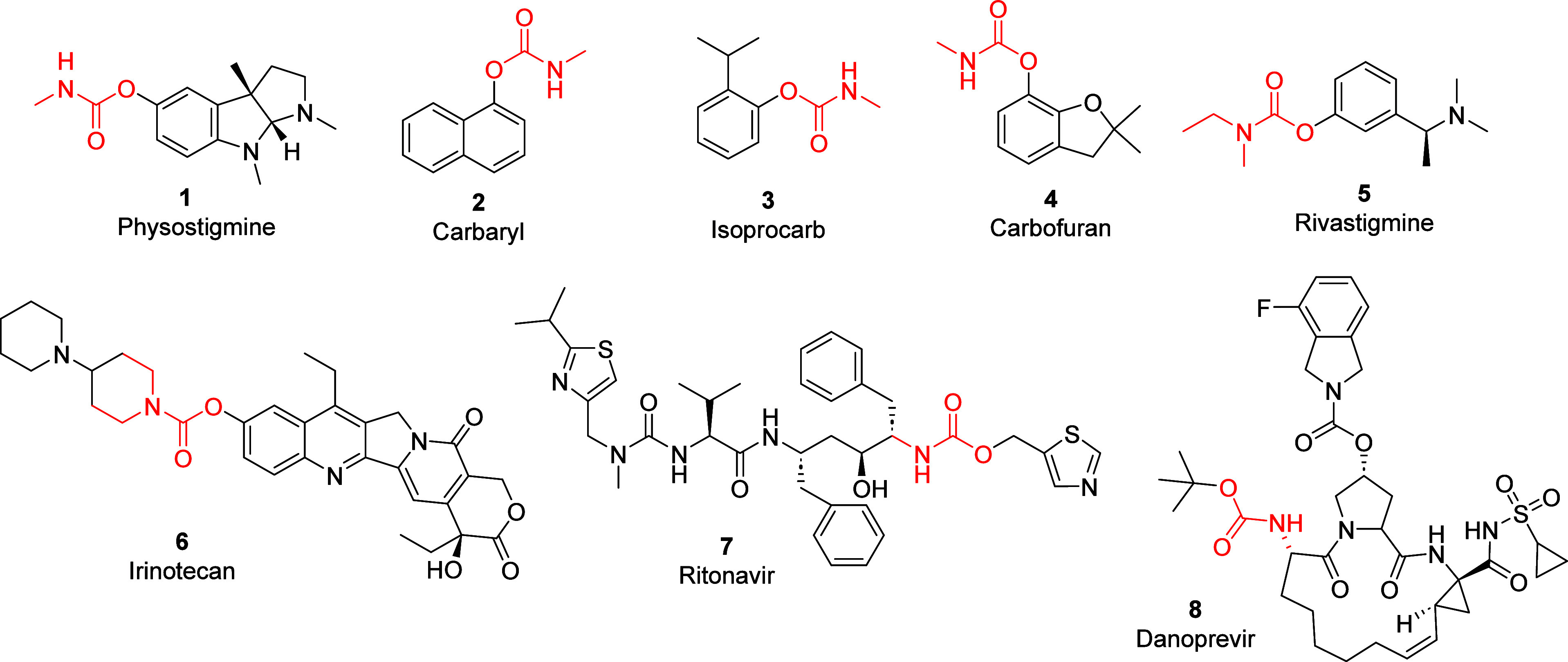
*O*-carbamate functional group in pesticides and drugs **1**–**8**.

The *O*-carbamate group, −OCONR_2_ (R = H or alkyl group), a salt or ester derived from carbamic acid, received its christening in 1845 with Wohler’s formulation of ethyl carbamate, a product of the reaction between urea with ethanol.^1^ While the carbamate is now a common functional group,^2^ its natural origins arose with the discovery of physostigmine (**1**), isolated from the calabar bean (*Physostigma venenosum*) of the Ivory Coast (Figure 1).^3^ Physostigmine holds a compelling folklore as a truth serum^4^ and more recently was found to show reversible acetyl cholinesterase (AChE) inhibition with an application toward treating glaucoma.^5^ Undoubtedly because of its AChE inhibitor activity and proteolytic stability, the carbamate group has been incorporated into a number of pesticide products and entered medicinal chemistry programs.^6^ The main action of these carbamate derivatives serves to inhibit the AChE enzyme by overstimulation of the nervous system.^5^

#### 1.1.2 Carbamates in Agriculture and Medicine

The first commercial carbamate product, Carbaryl (**2**), was introduced in 1956 and became the largest selling insecticide worldwide compared to all other carbamates combined (Figure 1).^7^ Unfortunately, the viewpoint of carbamate pesticides such as Carbaryl (**2**), Isoprocarb (**3**), and Carbofuran (**4**) received infamy as a result of the Bhopal pesticide plant tragedy in 1983.^8^

Presently, the bioactive significance of carbamates in drug design is evident in medications such as Rivastigmine (**5**),^6^ Irinotecan (**6**),^6^ Ritonavir (**7**),^6^ and Danoprevir (**8**). Danoprevir was found to be preferable as a treatment for COVID-19^9^ compared with Lopinavir/Ritonavir.^10^ These few examples are representative of a vast library of compounds. We refer the readers to two recent comprehensive reviews of pharmaceutical compounds containing organic carbamates.^6,11^

Traditionally, ArOAms have been prepared from the reaction of alcohols with toxic phosgene (or its derivatives), followed by treatment of the resulting phenyl carbonochloridates with amines.^12^ In more recent times, CO_2_ as a “nontoxic, renewable, and easily available C1 reagent”^13^ has been studied as a replacement for phosgene in this synthetic process.^14−16^ Building on growing interest in the synthesis of ArOAm, this review focuses on the *utilization* of OAm as a directed metalation group (DMG) in D*o*M and other metalation chemistry.

### 1.2 The Advent of the ArOAm in Directed *ortho* Metalation (D*o*M) Chemistry

In 1983, as part of early studies of the D*o*M reaction, the Snieckus’ group serendipitously discovered the ArOAm to serve as a directed metalation group (DMG).^17^ As D*o*M became more popular for the construction of polysubstituted aromatic molecules, a hierarchy of relative “DMG power” was established for *O*-based DMGs (see section 2, Table 1). Accordingly, the ArOAm DMG, especially as OCONEt_2_, demonstrated the greatest relative DMG power according to inter- and intramolecular competition studies.^18^ In these experiments, two DMG substrates and two DMGs on the same substrate, respectively, competed for 1 equiv of base, followed by quenching with a deuterium source (typically MeOD). Although the discovery of the ArOAm anionic *ortho-*Fries (A*o*F) rearrangement^17^ and the directed remote metalation (D*re*M) reaction^19^ fuelled interest in this functional group, this was initially somewhat dampened by the resistance of the ArOAm system to hydrolysis, a problem subsequently overcome via base-catalyzed hydrolysis (to phenols)^17^ and various cross-coupling (CC) strategies^20^ (see section 8).

### 1.3 Aims of This Review

In this review, we aim to update the literature related to D*o*M and A*o*F chemistry of the aryl *O*-carbamate (ArOAm) system since our previous comprehensive review in 1990^21^ and fragmentary appraisals thereafter.^22,23^ For a deeper discussion of A*o*F, we recommend Korb’s 2019 review.^24^

Following a general overview of the D*o*M reaction, the function and applications of the *O*-carbamate DMGs will be discussed with the inclusion of work from other research laboratories and highlights of preceding reviews.^22,23^ The contextual value of the OAm DMG will be refreshed alongside a comparison with other oxygen-based DMGs (e.g., OMe, OMOM). Furthermore, recent applications from our work and those of other research groups will be provided.

## 2 Directed *ortho* Metalation (D*o*M) Chemistry

### 2.1 Background: D*o*M History

In 1939–1940, the D*o*M reaction of anisole was independently discovered by Gilman^25^ and Wittig.^26^ Then, in the 1940–60 period, the breadth of DMG reactions was studied and expanded by the work of Puterbaugh (*N*-methylbenzamide),^27^ Shirley (indoles and phenoxathiine oxides),^28,29^ Wittig (substituted anisoles),^26,30,31^ Morton (cumene),^32^ and Gilman (aryl sulfides, naphthalenes, dibenzothiophene, and fluorene),^33−35^ further accelerating the field of synthetic organolithium chemistry. Additional development of the D*o*M reaction thereafter was strongly motivated by the contributions of Beak, Christensen, Meyers, and Parham,^22,36^ among others.

**Scheme 1:**
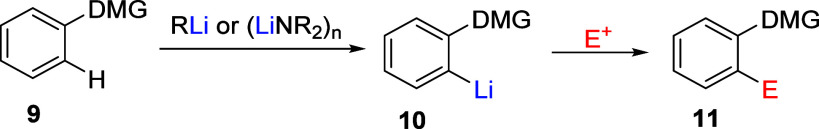
General D*o*M Reaction

The D*o*M process is well documented.^21^ A general regioselective D*o*M process (Scheme 1) involves treatment of a DMG bearing substrate **9** with a strong base, typically an alkyl lithium (represented as RLi) or lithium amide base (LiNR_2_)*_n_* at cryogenic temperatures (−78 °C). Coordination of the base as an aggregate to the heteroatom-containing DMG occurs and deprotonation leads to *ortho*-lithiated species **10**. Upon treatment with an electrophilic reagent, this species in turn yields a 1,2-disubstituted product **11**.

### 2.2 The D*o*M Reaction: Mechanism

The mechanism of D*o*M is not entirely understood and is controversial due to several considerable shortcomings in its mechanistic picture. The first main point of dispute involves the formation^37,38^ (or not^19,39^) of intermediate complexes as a step preceding the accepted rate-determining deprotonation.^40,41^ Further mechanistic concerns involve the determination of the actual existing structure of such complexes,^42^ the described mechanism of activation of the abstracted *ortho* H atoms,^43−47^ and the ambiguous role displayed by TMEDA in comparison with other common donor additives/solvents (e.g., ethers).^48−50^ As a result, two conflicting perspectives of the mechanism of D*o*M mechanism have been proposed: the complex-induced proximity effect (CIPE)^51^ and the kinetically enhanced metalation (KEM).^52^

**Scheme 2:**
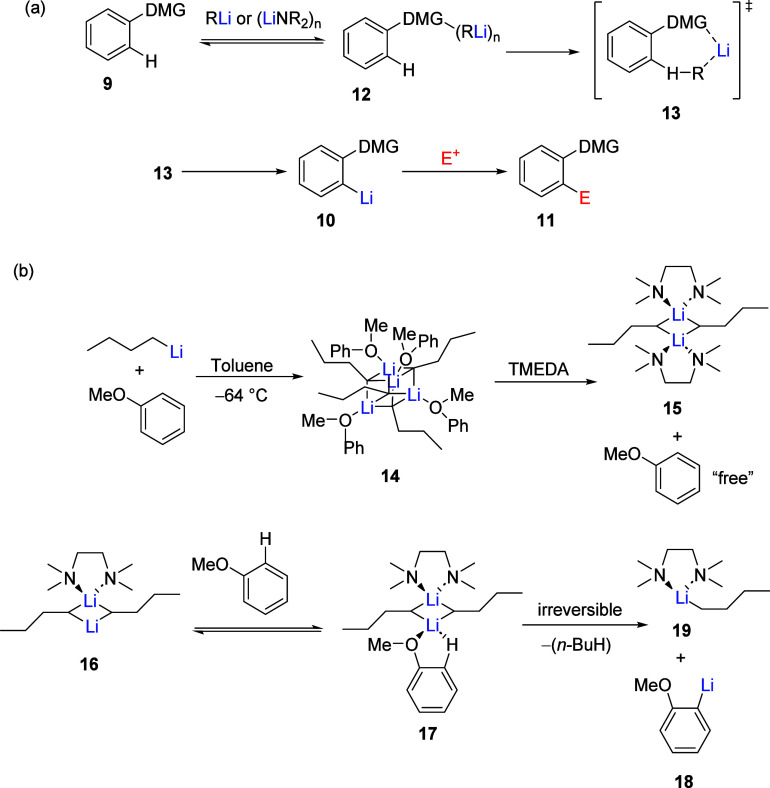
(a) General CIPE Concept (Adapted with Permission from ref (53), Copyright 2016 John Wiley and Sons); (b) Schleyer’s KEM Mechanism for *ortho*-Lithiation of Anisole with *n*-BuLi (Adapted from ref (52), Copyright 1989 American Chemical Society)^17,18,26,31,37,85−119^

The viewpoint of CIPE in deprotonation reactions follows the mechanisms which underlie carbanion chemistry. CIPE encompasses the idea that a momentarily bound species can promote a chemical reaction. Generally, the phenomenon of CIPE involves carbanion development by organolithium bases where the formation of a prelithiation complex gathers reactive groups into the same vicinity for a focused deprotonation reaction (Scheme 2a).^51^

The CIPE concept is qualitatively outlined for a D*o*M reaction as follows. Treatment of **9** with an organolithium reagent affords the complex **12**. Consequent directed lithiation of **12** through the transition state **13** leads to **10**, which, when treated with an electrophile, gives the disubstituted product **11**. CIPE has been considered in cases involved with deprotonative mono- and dilithiations, heteroatom–lithium exchanges, displacements, and additions.^42,47,54−56^ CIPE may also be suggested to govern the regioselectivity of D*o*M reactions by modifying the balance of inductive and association effects.^51,57^

Solid-state and solution studies provide circumstantial support for complexation preceding the deprotonation reaction. Stable organolithium compounds are well-established as highly aggregated and associated with ligands.^58−62^ The stabilization can be accredited based on a favorable binding energy between the positive charge of the lithium atom and the negative charge of the carbanion or the electron pair of a Lewis base in intermolecular and/or intramolecular interactions. Supportive data for the complexation of lithium–electron pair in ground states has been provided by X-ray crystallographic structures.^63^ Furthermore, HF and DFT *ab initio* calculations by Saá^64^ provide theoretical evidence for the requirement of precomplexation, either associative or dissociative, and thus offer additional inferred support for CIPE mechanism of *ortho*-lithiation.

The early Li NMR studies based on HOESY and MNDO calculations by Bauer and Schleyer^52^ offer contrasting mechanistic evidence for the formation of structure **10**, represented by **18** in Scheme 2b. The results revealed that the *ortho*-lithiation of anisole encompassed an unreactive complex between “free” anisole and *n*-BuLi, exhibiting Li–H interaction, detected by HOESY. In a toluene solution at −64 °C, anisole and *n*-BuLi exist as a tetrameric aggregate **14**. The addition of 1 equiv of TMEDA produces the 1:1 *n*-BuLi-TMEDA dimer **15** and “free” anisole (no HOESY anisole–Li interactions), which does not undergo *ortho*-lithiation. It appears that this *n*-BuLi·anisole complex **15** is seemingly uninvolved in the reaction. Exchange of one of the two coordination sites on species **15** (via **16**) by coordination of anisole leads to species **17** with oxygen agostic Li–H interactions. Irreversible deprotonation in turn gives *ortho*-lithiated species **18** (representative of **10**) and 1:1 *n*-BuLi-TMEDA species **19**, which can both undergo reaggregation. Therefore, in addition to CIPE, the kinetic (Li bears more than one available coordinating site) and thermodynamic (coordination of a heteroatom *ortho* to Li) aspects seem noteworthy mechanistic considerations of the D*o*M process for the OMe DMG.

#### 2.2.1 CIPE vs Schleyer’s KEM

**Scheme 3:**
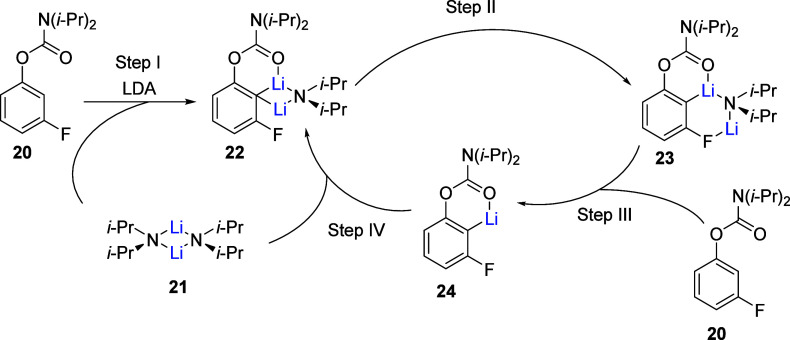
Collum’s Mechanism for the D*o*M Reaction of ArOAms Adapted from ref (75). Copyright 2008 American Chemical Society.

Both CIPE and Schleyer’s KEM mechanisms agree that proton transfer is the rate-determining step. The major difference between the two reaction pathways centers on whether a complex forms prior to proton transfer. In the case of CIPE, studies of intramolecular versus intermolecular isotope effects rule out a one-step process;^65^ i.e., CIPE postulates a complex as an intermediate along the reaction pathway whereas KEM does not.^51^ Furthermore, KEM necessitates proton transfer as the only step between the reactant and the lithiated product. Qualitatively, experimental observations^42,51,66,67^ based on geometrical consideration of a coordinated DMG-base species proximate to the reactive site are suggestive of the significance of the CIPE supposition. Furthermore, kinetic analyses on the *ortho*-lithiation of anisole^68^ and ArOAms^69^ provide evidence for mixed dimers^70−72^ or triple ions^49,73,74^ as plausible intermediates for the exhibited first-order kinetics of the reactions. The rate studies^69,75^ of the LDA-mediated *ortho*-lithiation of carbamate **20**(Scheme 3) offer no evidence for carbamate–LDA complexation; the resulting divergent rate behaviors for the *ortho*-lithiation of **20** seem to arise from mixed aggregates and autocatalysis instead.^76−81^ Increasing evidence suggests that autocatalysis may be widespread in LDA/THF-mediated reactions at −78 °C.^75^ Thus, the examination of LDA-mediated *ortho-*lithiation would provide further insights into how aggregation and solvation may influence organolithium reactivity. In the proposed mechanistic model for the LDA-mediated metalation of carbamate **20**, autocatalysis results from the transformation of interceding LDA–ArLi mixed dimer **22** into **23** (Scheme 3, steps I and II).

**Table 1:**
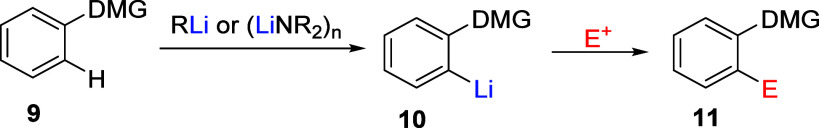

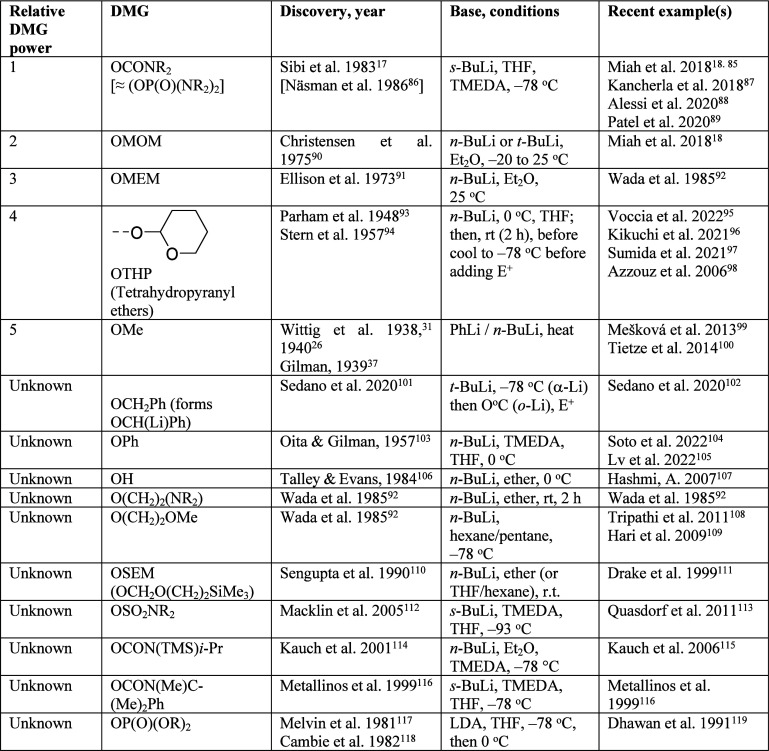
Hierarchy of Some *O*-Based DMGs in D*o*M^a^

a Adapted with permission from ref (85). Copyright 2018 John Wiley and Sons.

Metalation of another incoming arene **20** occurs via **24** (step III) and condensation of free species **24** with LDA dimer **21** (step IV) is rate limiting. Although no kinetic evidence is observed for precomplexation by Collum, there are implications of CIPE being important to *ortho*-lithiation in several mechanisms involving either Li–O or (C=C)–Li (π) interactions.^68^

At least two profoundly distinctive *ortho*-lithiation mechanisms are exhibited by the experiments of Pansegran et al. (involves the formation of a benzyne intermediate)^82^ and Maggi et al. (DMG- and solvent-dependent mechanism),^83^ just to complicate the discussion further.

### 2.3 The D*o*M Reaction: Synthetic Aspects

#### 2.3.1 DMG Hierarchy and Methodology

An effective DMG simultaneously involves the contrasting properties of being an appropriate coordinating-*ortho*-deprotonating group and a poor electrophilic target for nucleophilic alkyl lithium, enabling successful coordination-deprotonation. Steric hindrance and charge deactivation (or both) are also considered when crafting a favorable metalation director.^21^ Thus, more effective DMGs consist of stronger Lewis bases, such as the OCONR_2_ group, while weaker DMGs tend to also be weaker Lewis bases, e.g., the 2-methoxyethoxymethyl ether (OMEM) group.

For a complete list of carbon- and heteroatom-DMGs, we refer the reader to works by Snieckus.^18,21,22,84^ For the focus of this review, a summary list of the most reliable and widely used oxygen-based DMGs, ordered according to their *ortho*-directing ability (for the most powerful five DMGs, at least), is depicted in Table 1.

From the established but qualitative hierarchy of DMGs,^18,21,22,84^ we focus on a repertoire of valuable oxygen based DMGs (Table 1). The leading hierarchical position of the *O*-carbamate DMG strongly classifies it as a worthy choice for the regioselective construction of substituted aromatics by D*o*M chemistry, especially for substrates with Lewis acid stability requirements (Table 1).^85^ Of the ranked positions 2, 3, and 4, the mildly acid sensitive *O*-DMGs, OMOM (2-methoxymethyl ether), have experienced the most utility.^18,120−124^ Conversely, due to reactivity and convenience,^125,126^ it is unclear why OMEM (2-methoxyethoxymethyl ether) and OTHP (tetrahydropyranyl ether) have not been more widely tested as DMGs. It is interesting to note that the addition of 0.5 mol % LiCl as a catalyst slows down the lithiation of *O*-carbamates compared to other DMGs, especially halides.^127^

**Figure 2:**
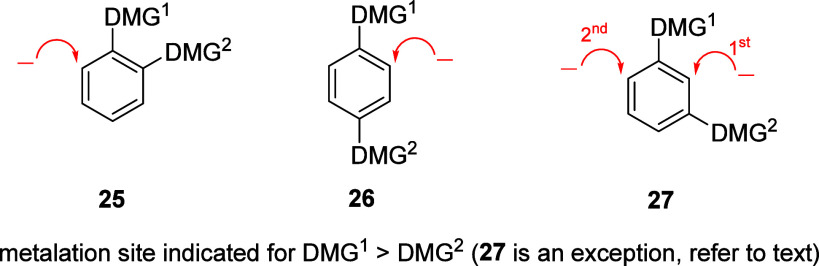
D*o*M hierarchy of all theoretically possible di-DMG-substituted aromatics. For **27**, the 1st and 2nd point of attack are indicated (1st versus 2nd). Adapted with permission from ref (18). Copyright 2018 John Wiley and Sons.

We also introduce the additional consideration of *ortho*-, *meta*-, and *para*-related di-DMG frameworks (Figure 2).^18^

A combination of two DMGs, systems **25** and **26**, are unremarkable in that metalation is predicted to be followed in hierarchical fashion (as denoted by the arrows), while *meta*-DMG system **27** possesses the advantageous attribute of synergistic metalation between the two DMGs.

In a comprehensive methodological investigation of the D*o*M reaction for *O*-phenyl *N*,*N*-diethylcarbamate **28a** under the formerly established standard conditions,^17^ a diversity of electrophiles was employed to afford products **28b**–**n** in good-to-excellent yields^85^ (Table 2). Among the alkylation and allylation products (**28b** and **28h**), *ortho*-sulfur (**28l**), and -halo (**28m** and **28n**) substituted compounds were also cleanly obtained. Products **28h** and **28i** are exceptions, possibly because of single electron transfer (SET) reactions and the presence of acidic C–H bonds in the electrophiles, respectively. These results demonstrate that aryl *N*,*N*-diethyl-*O*-carbamate is an outstanding DMG for the regioselective assembly of *ortho*-substituted phenol derivatives.

**Table 2:**
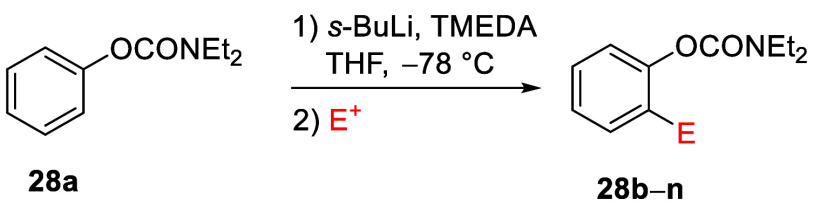
D*o*M Synthesis of *ortho*-Substituted *O*-Aryl *N*,*N*-Diethylcarbamates^a^

**E^+^**	product	**E**	yield (%)
MeI	**28b**	Me	80
DMF	**28c**	CHO	73^c^
CO_2_	**28d**	CO_2_H	73
ClCONEt_2_	**28e**	CONEt_2_	86
Me_3_SiCl (TMSCl)	**28f**	SiMe_3_	79
MeOD	**28g**	D	95^c^
3-bromoprop-1-ene	**28h**	–CH_2_C=CH_2_	75
Ac_2_O	**28i**	COMe	22
PhCHO	**28j**	CH(OH)Ph	90
Ph_2_CO	**28k**	C(OH)Ph_2_	22
(MeS)_2_	**28l**	SMe	79
BrCH_2_CH_2_Br	**28m**	Br	86
I_2_	**28n**	I	78

a Adapted with permission from ref (85). Copyright 2018 John Wiley and Sons.

b 42% of this product was isolated as salicylaldehyde.

c 96% d-incorporation.

The summarized results of a competitive metalation study^18^ featuring four DMG-bearing derivatives at either the *o*-, *m*-, or *p*- position (DMG = Cl, OMe, OMOM, and CONEt_2_, **28o**–**u**) adjacent to the OCONEt_2_ DMG are shown in Table 3. When in a 1,3-association with a stronger DMG (see Figure 2), the chloro group may contribute to regioselective 2-deprotonation to give **29a** (entry 1) even though it is a poor DMG on its own. Noteworthy is the fact that the 4-OMOM derivative **28s** gives the major product **29e** (70%; entry 5), a result which confirms the greater DMG power of OCONEt_2_ over OMOM. In considering the overall selected results in Table 3, the strength of the *O*-carbamate group as an *ortho* director is evident when compared to the chloro, methoxy, tertiary amide, and methoxymethoxy groups. Coupled with the results of *O*-based DMGs (Table 1), the *O*-carbamate is the principal choice for the preparation of polysubstituted phenol-based products.

**Table 3:**
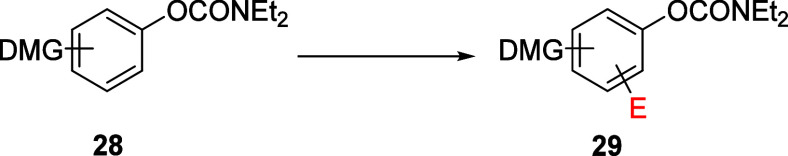
Selective D*o*M Reactions for DMG-Substituted ArOAms **28**^a^

entry	compd	DMG	**E^+^**	product	DMG	**E**	yield (%)
1	**28o**	3-Cl	PhCHO	**29a**	3-Cl	2-PhCH(OH)	81
2	**28p**	2-OMe	ClCONEt	**29b**	6-OMe	2-CONEt_2_	90
3	**28q**	3-OMe	I_2_	**29c**	3-OMe/5-OMe	2-I/2-I	75 (92:8)
4	**28r**	3-OMOM	ClCONEt_2_	**29d**	3-OMOM	2-CONEt_2_	24
5	**28s**	4-OMOM	ClCONEt_2_	**29e**	4-OMOM	2-CONEt_2_	70
6	**28t**	3-CONEt_2_	ClCONEt_2_	**29f**	3-CONEt_2_	2-CONEt_2_	7
7	**28u**	4-CONEt_2_	ClCONEt_2_	**29g**	4-CONEt_2_	2-/3-CONEt_2_	69 (3:2)

a Adapted with permission from ref (18). Copyright 2018 John Wiley and Sons.

#### 2.3.2 Comparison of ArOAm with Aryl *O*-Thiocarbamates

**Scheme 4:**
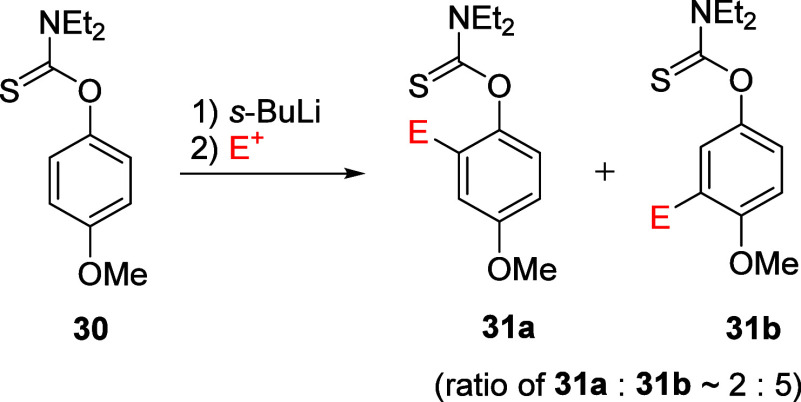
Regioselective Metalation of *p*-Methoxythiocarbamate **30**, Resulting in the Formation of **31a** and **31b** in a Stoichiometric Ratio of Approx 2:5 Adapted with permission from ref (130). Copyright 1997 Taylor & Francis.

Like the ArOAm functionality, the corresponding aryl *O*-thiocarbamate (ArOCSNR_2_) group also acts as an *ortho*-directing metalation group.^128^ However, the directing ability of −OCSNEt_2_ is far weaker than that of −OCONEt_2_.^129^ Indeed, the *O*-thiocarbamate group is weaker than OMe (the fifth most powerful DMG, see Table 1) according to intramolecular competition experiments in which *p*-methoxythiocarbamate **30** preferentially metalated *ortho* to the methoxy group over the *O*-thiocarbamate (mole ratio of **31a**:**31b** ∼ 2:5) (Scheme 4).^130^

**Scheme 5:**
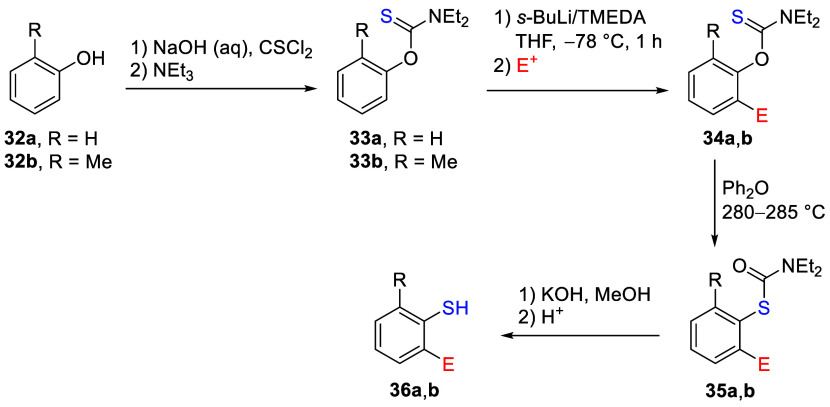
Conversion of Phenols **32** into *ortho*-Substituted Thiophenols **36** via the NKR (**34**→**35**) Adapted with permission from ref (131). Copyright 1992 Thieme.

In comparison, *p*-methoxycarbamate solely metalates *ortho* to the carbamate group.^17^ Nevertheless, aryl *O*-thiocarbamates are useful for the preparation of *ortho*-substituted thiophenols from phenols **32** (Scheme 5).^131^ This can be carried out by first forming the *O*-thiocarbamate from the corresponding phenol^132^ before performing D*o*M on the aryl *O*-thiocarbamates **33** to form the *ortho*-substituted aryl *O*-thiocarbamates **34**. This can be converted to *S*-phenyl diethylcarbamothioates **35** via a Newman–Kwart rearrangement (NKR)^131^ followed by acid/base hydrolysis to the corresponding thiophenols **36** (Scheme 5).^133,134^

The sequence is useful and circumvents the oxidative complications that arise when attempting to obtain **36** directly via electrophilic substitution.^135^

Similar chemistry was used by Kunz et al. in the preparation of benzothiadiazole-based plant activators.^136^ In this case, once the aryl *O*-thiocarbamate was converted to the aryl *S*-thiocarbamate (by NKR), the *S*-thiocarbamate functionality served as a useful protecting and leaving group in the final cyclization step to form the benzothiadiazole.

As a caution, Mondragón et al.^137^ reported that attempts to convert *ortho*-halogeno-aryl *O*-thiocarbamates to the corresponding aryl *S*-thiocarbamates via NKR were thwarted by competition with nucleophilic attack of the thiocarbamoyl S atom at the *ortho*-position. This difficulty was overcome by replacement of the halide with a carbonyl group.

Apart from the NKR, postsynthetic modifications of the aryl *O-* and *S*-thiocarbamate directing groups might include the A*o*F^24^ and *O*-neophyl^138^ rearrangements, as well as in *S*-thiocarbamate-Grignard cross-couplings, although more rigorous conditions are required compared with the corresponding *O*-carbamate coupling reactions.^135^

**Scheme 6:**
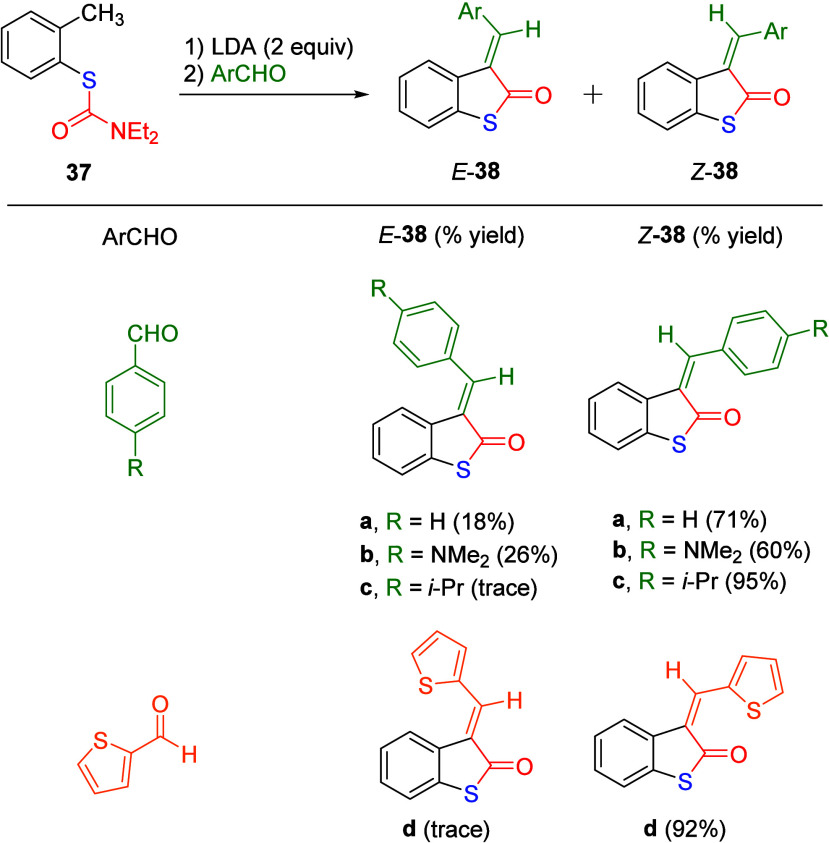
Synthesis of *E*- and *Z*-3-Benzylidenebenzo[*b*]thiophen-2(3*H*)-ones (*E*-**38a**–**d** and *Z*-**38a**–**d**) by Treatment of **37** with LDA Followed by Various Aromatic Aldehydes Adapted with permission from ref (139). Copyright 2004 The Japan Institute of Heterocyclic Chemistry.

We only located one report involving the aryl *S*-thiocarbamate group (ArSCONR_2_) in reaction with an organolithium base.^139^ In this case, treatment of *S*-(*o*-tolyl) diethylcarbamothioate (**37**) with LDA at −30 °C followed by quenching with various aromatic aldehydes provided 3-substituted *E*- and *Z*- derivatives of 3-benzylidenebenzo[*b*]thiophen-2(3*H*)-ones (Scheme 6). The results in the scheme show that the *Z*- diastereomers (*Z*-**38a**–**d**) were obtained as major products in reasonable to good yields with the *E*- diastereomers in smaller amounts (*E*-**38a**–**d**). Mechanistically, this is due to the initial abstraction of a proton from the *ortho*-methyl group by LDA to give an anion which undergoes cyclization followed by condensation with the aromatic aldehyde, forming isomers *E*-**38** and *Z*-**38** upon acidic workup.

In conclusion, we point out that phenols can be converted to selenophenols in the same way that phenols can be converted to thiophenols via NKR.^140^ However, the *ortho* metalation of aryl *Se*-carbamates is yet to be explored. In general, aryl *S*-carbamates and *Se*-carbamates have not been explored more fully, probably due to their toxicity to transition metals.^141,142^ Aryl dithiocarbamates have not yet been explored in the context of D*o*M; this could also be a worthy pursuit given their biological relevance.^143^

#### 2.3.3 D*o*M Reactions of ArOAm on Solid Support

**Scheme 7:**
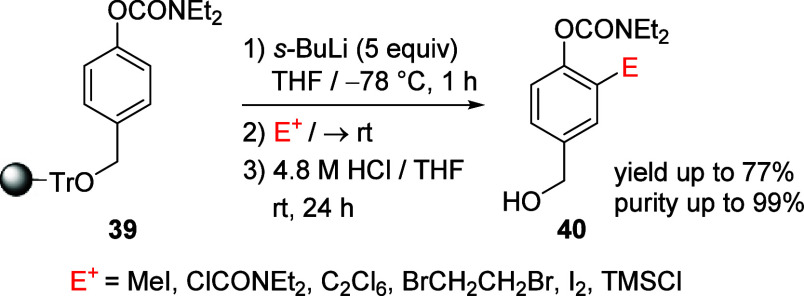
D*o*M Reaction of ArOAms on PS-DVB Resin Using a Trityl Linker Adapted from ref (146). Copyright 2005 American Chemical Society.

Organic synthesis involving reactants or reagents on solid support is a rapidly growing field as it offers multiple advantages including, for example, using excess reactants/reagents to drive reactions to completion, combined with their easy recovery and reuse in subsequent reactions.^144,145^ The first report of a solid-supported D*o*M reaction involving ArOAms was in 2005.^146^ In this work, ArOAm **39** on PS-DVB resin (immobilized using a trityl linker, “TrO”) was metalated and exposed to a range of electrophiles followed by removal of the support to give substituted benzyl alcohols (**40**) in high purity at low temperatures and short metalation times (Scheme 7).

Some reaction conditions had to be modified for the reaction of ArOAms on solid support. Normally, 1.2 equiv of *s*-BuLi base and TMEDA additive are used for the solution phase D*o*M reaction of the same substrate, whereas 5.0 equiv of base was required for the solid phase reaction while exclusion of TMEDA additive increased the yield/purity of the product **40** almost quantitatively. Furthermore, increasing the amount of THF in the reaction (THF/hexane ratio of 10:1) gave 99% yield for MeI electrophile.

#### 2.3.4 Alternative Metalation Reagents with the OAm Group

Traditionally, organolithiums are used to realize D*o*M, and these are typically alkyl lithium compounds or bulky lithium amides. However, these reagents are highly reactive and tend to attack electrophilic DMGs or sensitive functional groups, causing undesired side reactions.^147^ Subsequent research to establish alternative, less harsh metalation reagents has been fruitful, with the discovery of metalation reagents in the form of single metals (Na),^148^ sodium and metal amides,^53,149−151^ sodium isopropyl(trimethylsilyl)amide,^152^ NaTMP (TMP = 2,2′,6,6′-tetramethylpiperidine) with tridentate donor PMDTA (*N*,*N*,*N*′,*N*″,*N*″-pentamethyldiethylenetriamine),^153^ mixed metals,^154,155^ metalates (magnesiates, zincates, and aluminates),^156−159^ (2,2,6,6-tetramethylpiperidino)magnesium chloride (TMPMgCl),^160^ mixed aggregates,^161,162^ “superbases,”^53^ and “Turbo bases”.^163,164^ There are only a few examples which have been demonstrated for ArOAms, shown in Table 4.

**Table 4:**
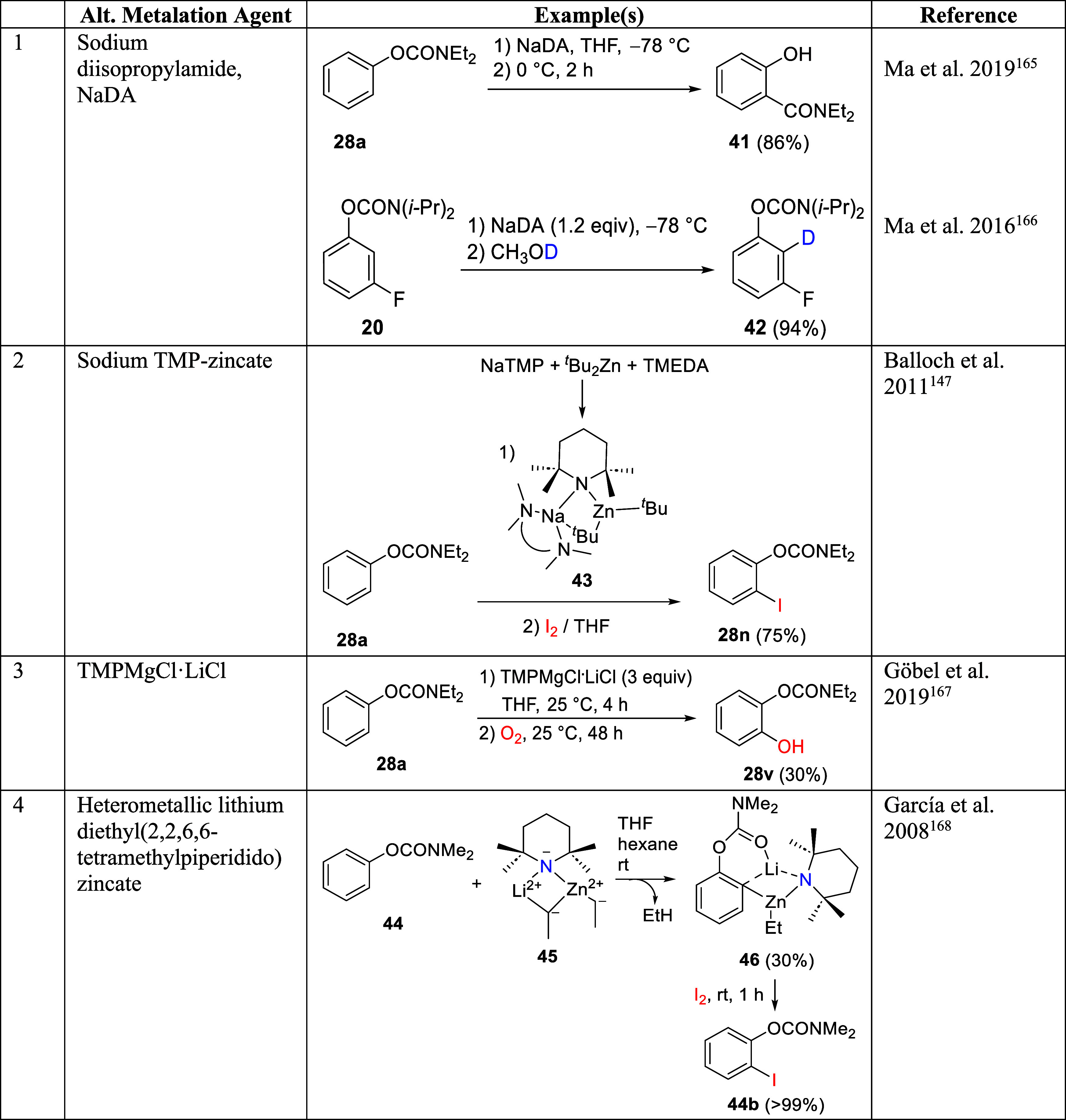
Alternative Metalation Agents to Organolithium Reagents^147,165−168^

Ma et al.^165^ demonstrated the possibility of performing A*o*F rearrangements using sodium diisopropylamide, NaDA, to obtain dialkyl-2-hydroxybenzamides in moderate to good yields (an example is shown in entry 1). A sodium-mediated TMP-zincation demonstrated by Balloch et al.^147^ resulted in *ortho*-metalation via a sodium TMP-zincate [(TMEDA)Na(μ-TMP)(μ-*t*-Bu)Zn(*t*-Bu)] (**43**), generated *in situ*, followed by electrophilic quench with a I_2_ solution (THF) to generate **28n** in 75% yield (entry 2). The yield is similar to that for the same product obtained using *s-*BuLi (Table 2, synthesis of **28n**).

Clear opportunities exist to understand other metalating reagents capable of metalation/functionalization of the ArOAms, e.g., sodium dispersion in sand or micronized (reduced to a fine powder).

## 3 ArOAm and *Remote* Metalation Chemistry

**Scheme 8:**
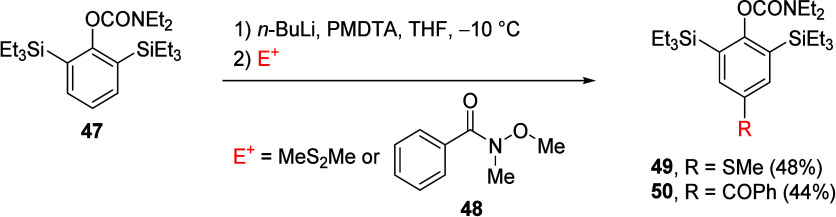
An Example of Direct Regioselective Remote Metalation of an Aromatic Molecule with Bulky TES Groups in the *ortho* Position Synthesis of **49** adapted with permission from ref (169). Copyright 2019 John Wiley and Sons. Synthesis of **50** adapted with permission from ref (170). Copyright 2019 Thieme.

As demonstrated by Bellan and Knochel,^169,170^ sterically hindered ArOAm can direct metalation (regioselectively) at the *para* position of aromatic molecules. For example, ArOAm with two bulky triethylsilyl groups at both *ortho* positions (**47**) can be regiospecifically lithiated in the *para* position with *n-*BuLi/PMDTA (PMDTA = *N*,*N*,*N*′,*N*″,*N*″-pentamethyldiethylenetriamine, a reactivity enhancing tridentate ligand^171^) (Scheme 8).

**Scheme 9:**
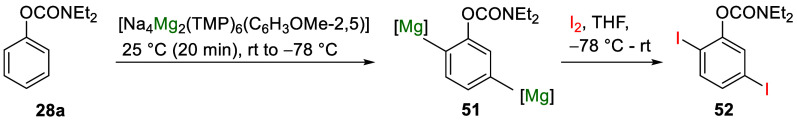
An Example of Remote Metalation to More Distant Arene Sites with OAm as DG Adapted with permission from ref (172). Copyright 2014 American Association for the Advancement of Science.

Martínez-Martínez et al.^172^ demonstrated that steric hindrance is not necessarily required: in this case, the use of a disodium-monomagnesium alkyl-amide could extend the regioselectivity of the metalation to more distant arene sites (*ortho*-*meta*′/*meta*-*meta*′, depending on the nature of the directing group). When the DG is OAm, *ortho*-*meta* magnesiated complex **51** forms (Scheme 9), which can be trapped with an electrophile, demonstrated using I_2_, to form trisubstituted **52**.

## 4 ArOAm and the Anionic *ortho*-Fries (A*o*F) Rearrangement

**Scheme 10:**
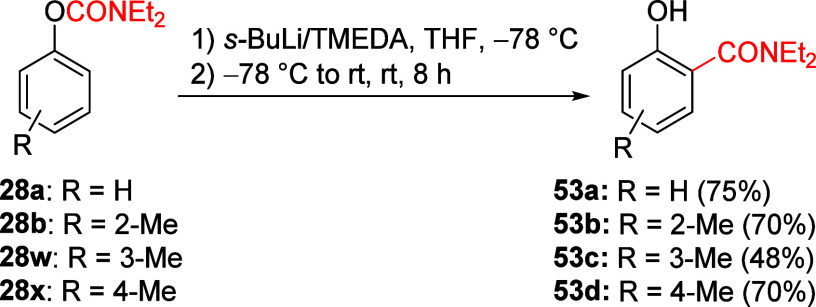
A*o*F Rearrangement to Salicylamide Derivatives (**53**) Adapted with permission from ref (85). Copyright 2018 John Wiley and Sons.

In the absence of an electrophile, when ArOAm **28a** forms an intermediate lithiated species under standard conditions of *s*-BuLi/TMEDA, this intermediate undergoes an A*o*F rearrangement upon slow warming to room temperature to produce the salicylamide derivative **53a** (Scheme 10).^18^ Similarly, methyl-substituted salicylamide derivatives **53b**–**d** (R, in relation to −OH) are formed from **28b**,**w**,**x**. Thus, tertiary *O*-carbamates are unstable at temperatures higher than −78 °C with respect to the 1,3-O→C carbamoyl rearrangement^24^ whose rate is primarily dependent upon the extent of substitution, especially *ortho* to the developing anion or to the OCONR_2_ group as well as the substituents on the N atom.^69^

**Scheme 11:**
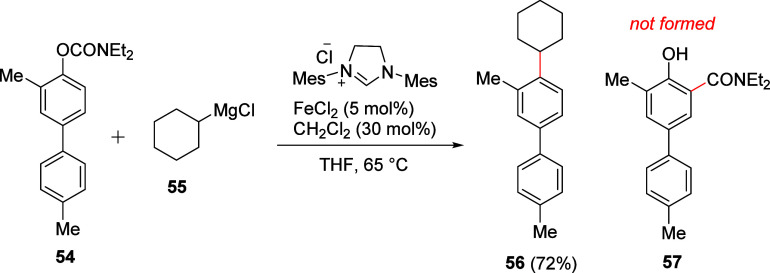
Iron-Catalyzed Coupling of *O*-Carbamate **54** with Cyclohexylmagnesium Chloride (**55**) to Furnish **56** Adapted from ref (174). Copyright 2012 American Chemical Society.

This rearrangement, more widely known for the transformation of aromatic esters to *ortho*- and *para*-acyl phenols with Lewis acids, is named after its discoverer, Karl Fries, who published the first examples of this process in 1908.^173^ ArOAms have yet to be transformed into *ortho*- and *para*-acyl phenols in this manner. An example of where this transformation may have been anticipated but was not observed can be found in work by Silberstein et al., in which the coupling of an *ortho*-substituted ArOAm **54** with an alkyl Grignard reagent **55** in the presence of a Lewis acid (FeCl_2_) provided the alkylated product **56** in 72% yield with no evidence for formation of the *ortho*-acyl phenol side-product **57** (Scheme 11).^174^ The anticipated Lewis-acid mediated Fries rearrangement product **57** was not observed.

The anionic version of this transformation, the A*o*F, was only discovered decades later in 1981 by Melvin,^117^ with the first examples reported by Snieckus’ group in 1983.^17^ In the A*o*F rearrangement, the *O*-carbamate can be conceptualized as acting as a “carrier” of the amide group into the *ortho* position and the creation of this new DMG may then facilitate further D*o*M chemistry.

**Scheme 12:**
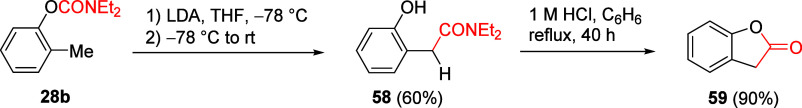
A*o*F Rearrangement Results in the Transformation of the *o*-Methylated Carbamate **28b** to Benzofuranone (**59**) Adapted with permission from ref (85). Copyright 2018 John Wiley and Sons.

A slight adjustment of the base employed changes the reaction outcome. For example, when treated with LDA instead of *s*-BuLi, 2-tolyl *O*-carbamate **28b** undergoes lateral metalation to give a phenylacetamide **58**, which can be hydrolyzed to a benzo[*b*]furan-2(3*H*)-one (**59**) in reasonable yield (Scheme 12).^18^

**Scheme 13:**
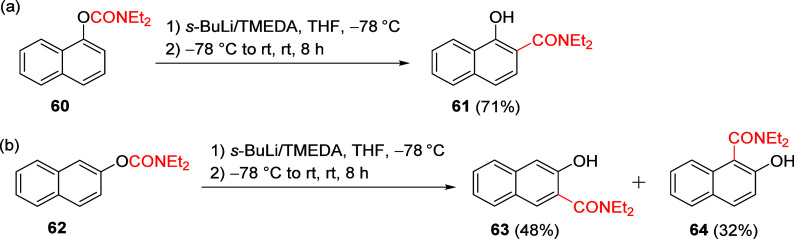
A*o*F Rearrangement of Naphthalenes **60** (a) and **62** (b) to Naphthamide Derivatives **61**, **63**, and **64** Adapted with permission from ref (85). Copyright 2018 John Wiley and Sons.

In a parallel study, the 1- and 2-naphthyl *O*-carbamates **60** and **62** were also subjected to standard A*o*F rearrangement conditions to give amides **61** and a mixture of amides **63** and **64** (Scheme 13). Alternative DMGs have also been applied to this reaction scheme in other work.^18^

### 4.1 The Scope of the A*o*F Rearrangement

The synthetic playground of aromatics is enlarged by considering substrates with two different DMGs in competition experiments (Table 5).^18^ Using standard A*o*F conditions, the 2-chloro and 2-methoxy ArOAms **28y** and **28p** afford the contiguously 1,2,3-trisubstituted salicylamides **65a** and **65b**, respectively (entries 1 and 2). By contrast, the 3-OMOM **28r** and 3-OCONEt_2_**28z** furnish the in-between A*o*F rearrangement products **65c** (68% yield; entry 3) and **65d** (86% yield; entry 4), respectively. Lastly, the amide ArOAm **28t** was subjected to the A*o*F rearrangement to afford **65e** (Table 5, entry 5) in relatively low yield. These results further emphasize the strength of the *O*-carbamate DMG as an *ortho* director in the A*o*F rearrangement and, not without synthetic consequence, furnish trisubstituted aromatics.

**Table 5:**
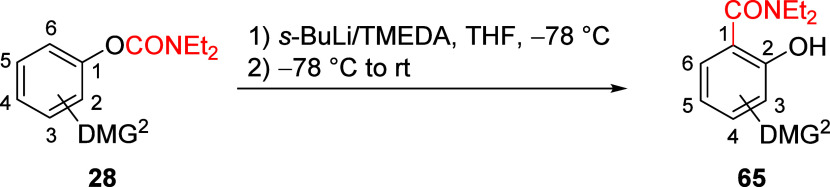
Competitive A*o*F Rearrangement of DMG-Substituted ArOAms **28**^a^

entry	compd	DMG^2^	product	DMG^2^ (product)	yield (%)
1	**28y**	2-Cl	**65a**	3-Cl	72
2	**28p**	2-OMe	**65b**	3-OMe	68
3	**28r**	3-OMOM	**65c**	6-OMOM	68
4	**28z**	3-OCONEt_2_	**65d**	6-OCONEt_2_	86
5	**28t**	3-CONEt_2_	**65e**	6-CONEt_2_	16

a Adapted with permission from ref (18). Copyright 2018 John Wiley and Sons.

It is noteworthy that the A*o*F of ArOAms can be suppressed using a zincate base (generated by the reaction of HTMP with *n*-BuLi and Et_2_Zn in THF at −78 °C under inert conditions), which reacts preferably with the aromatic component to give stable intermediates able to resist rearrangement.^168^

**Scheme 14:**
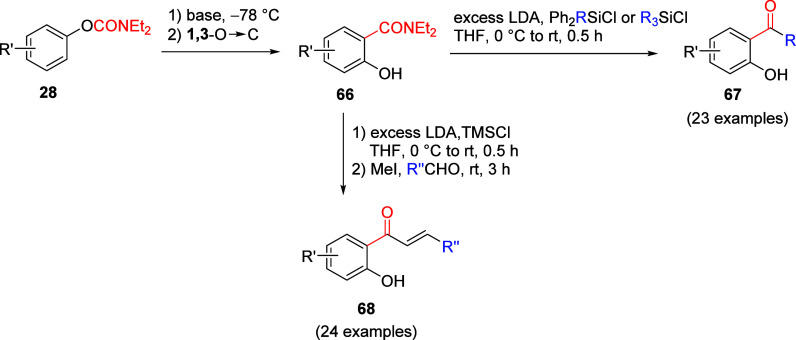
A*o*F Rearrangement of an ArOAm Forming Salicylamide **66** Followed by the Formation of Either a *o*-Hydroxyacetophenone Derivative **67** (Adapted with Permission from ref (175), Copyright 2014 John Wiley and Sons) or 2′-Hydroxychalcone **68** (Adapted from ref (176), Copyright 2018 American Chemical Society), Both via an Anionic Si → C Alkyl Rearrangement and Claisen–Schmidt Condensation

The A*o*F rearrangement also provides various opportunities for one-pot conversions of the ArOAm group to other functionalities. For example, the salicylamide **66** can be transformed into a *o*-hydroxyacetophenone derivative **67**(175) or a 2′-hydroxychalcone **68**(176) using the conditions shown in Scheme 14.

**Scheme 15:**
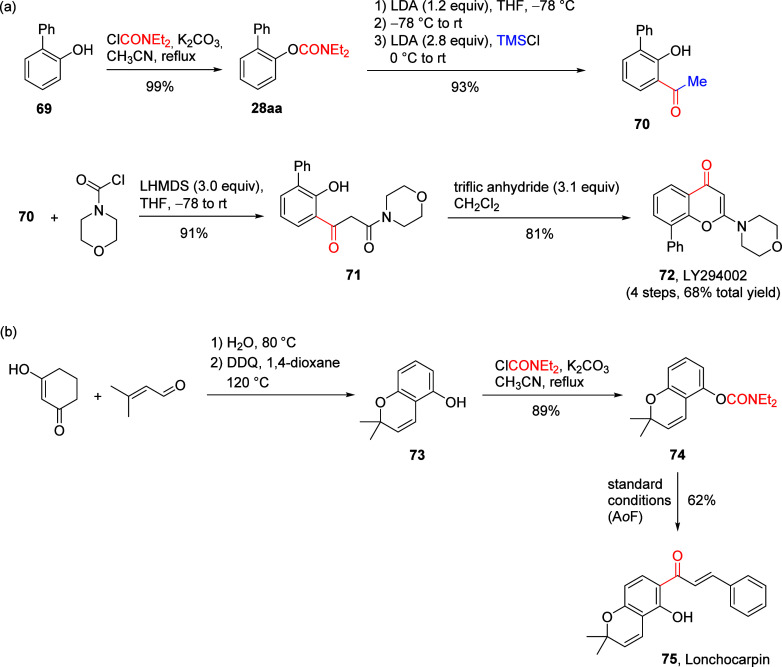
Synthesis of (a) the PI3K Inhibitor LY294002 (**72**) and (b) Lonchocarpin (**75**) by Application of the Extended A*o*F Rearrangement (a) Adapted with permission from ref (175). Copyright 2014 John Wiley and Sons. (b) Adapted from ref (176). Copyright 2018 American Chemical Society.

These transformations were shown to be useful in the formation of various medicinal compounds, namely the PI3K inhibitor LY294002 (**72**, Scheme 15a)^175^ used in cancer therapy, and the chalcone Lonchocarpin (**75**), a natural product with numerous pharmacological properties (Scheme 15b).^176^

Compound **72** was obtained in 4 steps with a 68% total yield, commencing with the preparation of diethylcarbamate **28aa** from *o*-phenylphenol (**69**) and diethylcarbamoyl chloride (Scheme 15a). Carbamate **28aa** was then subjected to an A*o*F rearrangement–methylation procedure to afford **70** which was coupled with 4-morpholinecarbonyl chloride to form salicylacetamide **71**. Subsequent cyclodehydration of **71** using trifluoromethanesulfonic anhydride provided LY294002 (**72**).^175^ Similarly, 2,2-dimethyl-2*H*-chromen-5-ol (**73**), prepared from 3-hydroxycyclohex-2-en-1-one and senecialdehyde,^177−179^ was transformed into carbamate **74**, which was then subjected to standard A*o*F reaction conditions to furnish the natural product Lonchocarpin (**75**) in 62% yield.^176^

## 5 OAm in Iterative (Walk-around-the-Ring) D*o*M

### 5.1 The Iterative D*o*M Strategy Conceptualized

**Scheme 16:**
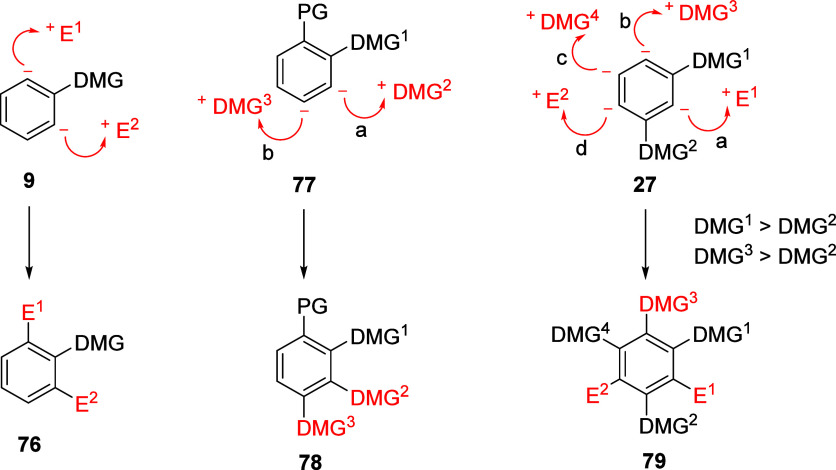
Conceptual Framework of Walk-around-the-Ring Iterative D*o*M Strategies to Various Generic Compounds **76**, **78**, and **79** Adapted with permission from ref (18). Copyright 2018 John Wiley and Sons.

The application of sequential multiple D*o*M reactions uncovers a plethora of opportunities. In general, successive metalation-electrophile quench with two electrophiles (same or different) can lead to a 1,2,3-substituted aromatic compound **76** (Scheme 16). Moreover, the use of a protecting group (PG) as in **77** allows another route to a contiguously trisubstituted derivative (step a).^18^ If DMG^1^ is reasonably strong, a further D*o*M reaction (step b) may allow for a third DMG (DMG^3^) introduction (to form **78**).^18^ Thus, the adaption to the walk-around-the-ring tactic for the conversion of an ArOAm to a tetrasubstituted aryl may be envisioned. Given two DMGs, the *ortho*, *meta*, and *para* relations may be considered. For the *meta*-DMG^1^–DMG^2^ isomer (**27**),^18^ the cooperative DMG effects influence the introduction of E^1^ (step a). If DMG^1^ > DMG^2^ in metalation power, DMG^3^ may be introduced (step b). Additional metalation may also occur if the introduced DMG^3^ > DMG^2^ in power which then permits the introduction of DMG^4^ (step c) followed by E^2^ (step d) to form **79**.

### 5.2 The OAm DMG Iterative D*o*M in Practice

**Scheme 17:**
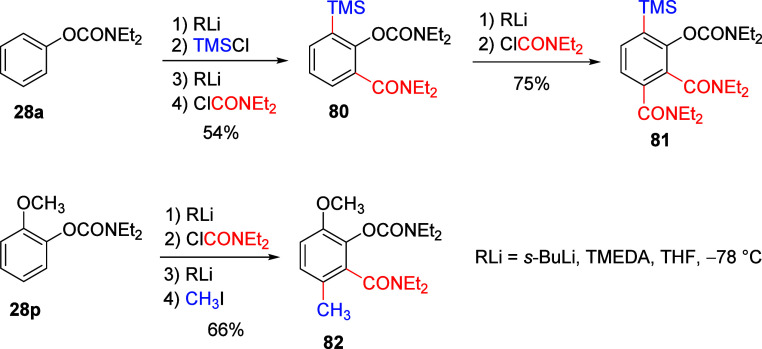
Two Tested Iterative D*o*M Cases Using ArOAm DMGs Adapted with permission from ref (18). Copyright 2018 John Wiley and Sons.

Two cases of the ArOAm DMG in iterative metalation experiments are presented herein (Scheme 17).^18^ In evidence of potential synthetic utility, *O*-phenyl *N*,*N*-diethylcarbamate **28a** was employed in sequential metalation, silylation, metalation, and carbamoylation with equivalent amounts of base and electrophile at −78 °C. The 1,2,3-substituted product **80** was obtained in reasonable 54% yield. Standard reaction of **80** with ClCONEt_2_ afforded the 1,2,3,4-substituted aromatic molecule **81** in good 75% yield.

In the second tested case, 2-methoxy *O*-carbamate **28p** was subjected to two metalation–electrophile quench sequences in one pot with quenching reagents ClCONEt_2_ and MeI to give a contiguously tetrasubstituted product **82** in an acceptable 66% yield.

### 5.3 Comparative Analysis of Different Approaches to Synthesis of Substituted Aromatic Compounds

A compiled collection of 1,2,3-substituted (entries 1–4) and 1,2,3,4-substituted aromatic systems (entries 5 and 6) is shown in Table 6 to provide a general summary of *O*-carbamate D*o*M methodologies in comparison to alternative synthetic procedures.

**Table 6:**
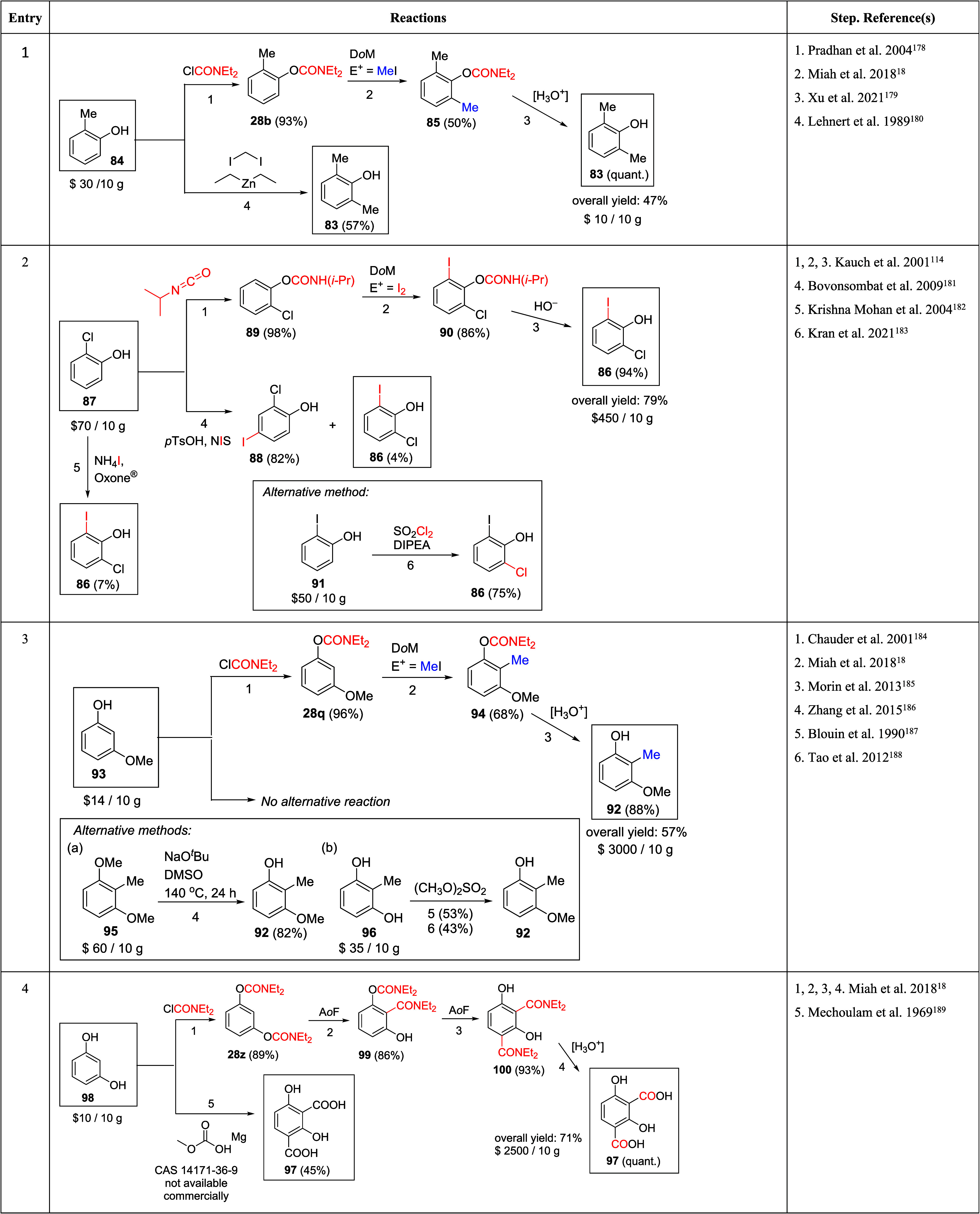

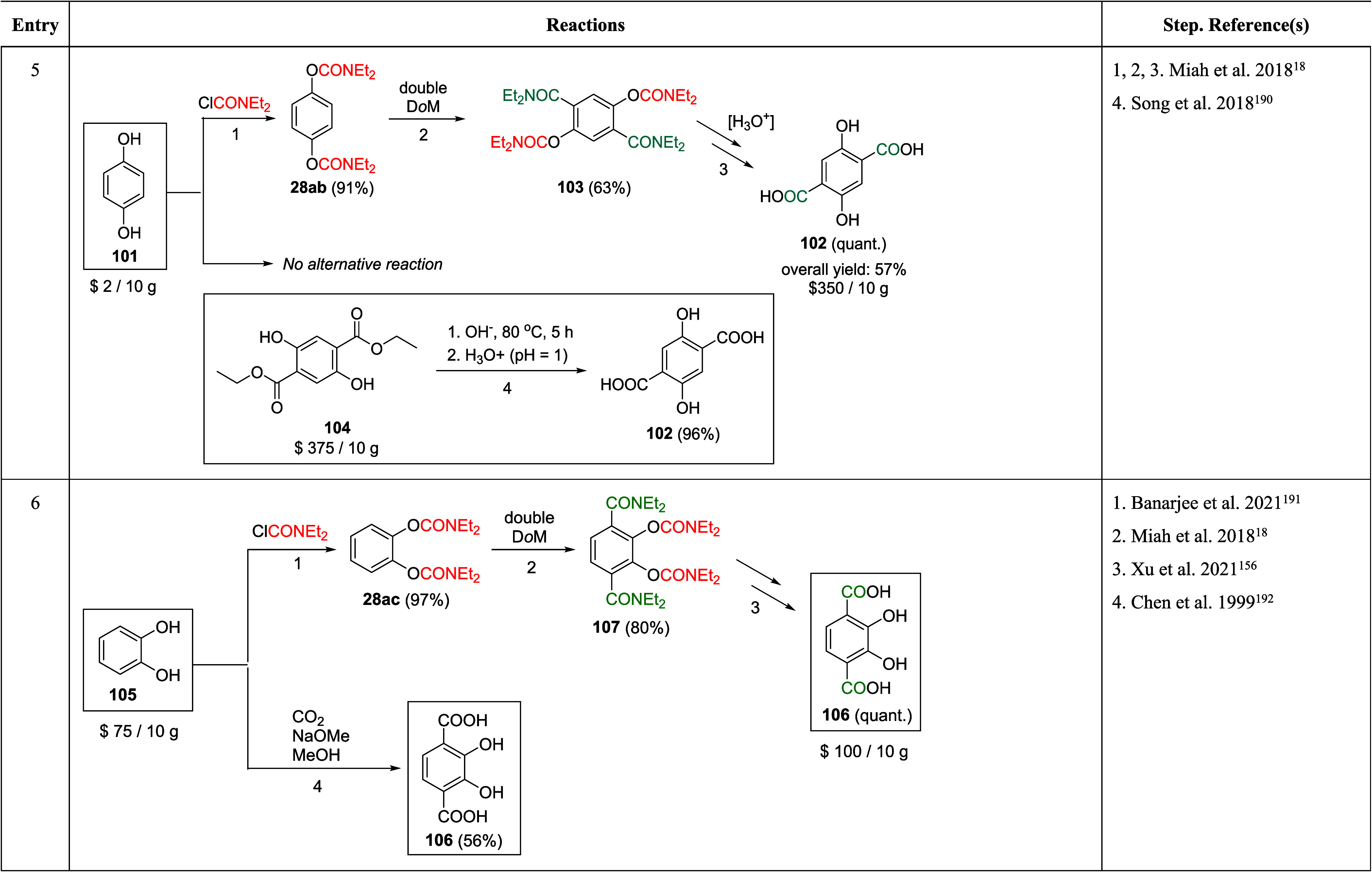
Competitive Methods (D*o*M-Based vs Alternative) for the Synthesis of Substituted Phenols. Approximate Value of Chemical Shown in USD (Average Calculated from the Price Supplied by Three Universal Vendors)^18,114,156,178−192^

The relatively inexpensive 2,6-dimethylphenol **83** (Table 6, entry 1) is produced in industrial chemistry from *ortho*-cresol (**84**) using high-pressure and high-temperature in two steps and 36% yield,^18^ whereas, with the opportunity for scale-up and consideration of cost, the same starting material, same number of steps, and 50% yield is attainable by *ortho*-lithiation of **28b** to 2,6-dimethylphenyl diethylcarbamate (**85**).^18^

Lehnert et al. prepared **83** in 57% in one-step using a Simmons–Smith reagent prepared *in situ* from diethylzinc and diiodomethane (entry 1).^180^**83** can also be prepared in a 3-step process involving D*o*M of **28b**, with OAm as DMG. The low cost of **83** makes neither route particularly appealing. By contrast, the comparatively expensive differentially halogenated phenol **86** (entry 2) has been prepared by D*o*M chemistry from 2-chlorophenol (**87**) in an overall acceptable yield (56%).^18,114^**86** has also been prepared in far lower yields starting from **87**, e.g., 4% yield, using *N*-iodosuccinimide and *p*-toluenesulfonic acid (*para*-substituted iodophenol is produced in 82% yield)^181^ or 7% yield via oxyiodination (*para*-substituted iodophenol is produced in 93% yield).^182^ A more attractive route commences with 2-iodophenol **91**, providing **86** in 75% yield via traditional electrophilic aromatic substitution using sulfuryl chloride.^183^

For the synthesis of 3-methoxy-2-methylphenol (**92**) (entry 3) starting from 3-methoxyphenol (**93**), the target molecule is accessible either using the carbamate **28q** (three steps: synthesis of ArOAm from **93** (96%),^184^ followed by D*o*M and installation of the methyl group (68%),^18^ and, finally, hydrolysis of OAm to OH (88%),^185^ giving a 57% overall yield) or relatively more expensive 1,3-dimethoxy-2-methylbenzene, **95** (one step, 82%),^186^ or cheaper 2-methylresorcinol, **96** (one step, 43%^187^ or 53% yield^188^).

For contiguously tetrasubstituted systems, the methods begin with resorcinol (**98**), hydroquinone (**101**), and catechol (**105**) via *O*-dicarbamates **28z**, **28ab**, and **28ac**, respectively, using D*o*M to provide the respective isophthalic (entry 4), and terephthalic (entries 5 and 6) target molecules (**97**, **102**, and **106**, respectively) in a reasonable number of steps with modest yields via reduction and hydrolysis reactions.

While alternative syntheses have been reported (e.g., selective carboxylation of **98** to **97**,^189^ hydrolysis of ester groups in **104** to form **102**(190) and selective carboxylation of **105** to form **106**(192)) and developed for large-scale commercial production,^191−195^ these routes typically involve high temperatures, dangerous materials, and corrosive reagents in variable yields. Therefore, the comparative examination of synthetic reactions in Table 6 competitively positions *O*-carbamate D*o*M methodology against other procedures for the manipulation of contiguously functionalized substituted aromatic derivatives. The provided approximations may guide informative decisions in synthetic efforts toward preparing alternative poly substituted aromatic targets.

### 5.4 Combined D*o*M-Halogen Dance (HalD) Synthetic Strategies

**Scheme 18:**
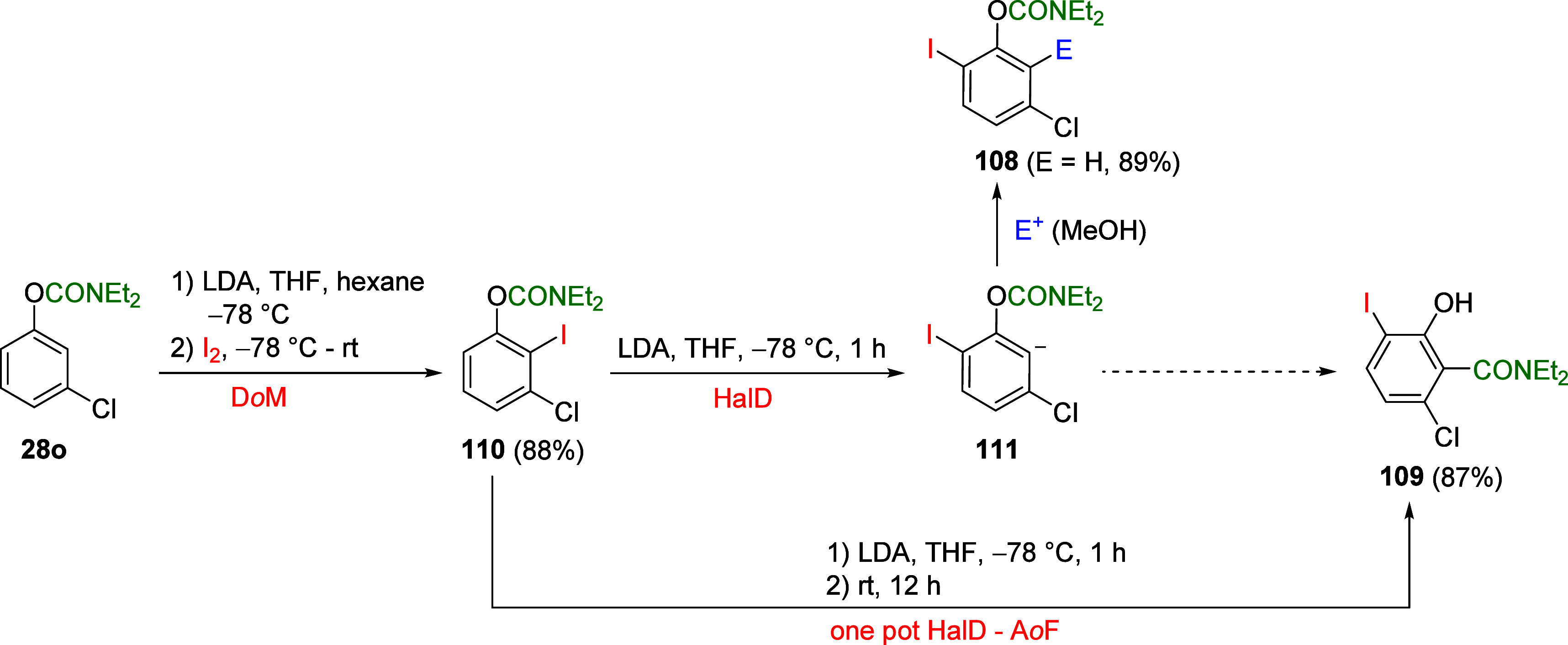
HalD-A*o*F of ArOAm **28o** via Initial Transformation to **110** via D*o*M (Adapted from ref (199), Copyright 2007 American Chemical Society), Which Can Be Transformed to **108** and **109** (Adapted with Permission from ref (198), Copyright 2010 John Wiley and Sons)

The HalD reaction involves the intraring migration of a halogen substituent and is essentially a 1,2-rearrangement reaction based on a combination of halogen/lithium exchange and deprotonative lithiation.^196,197^ Miller et al.^198^ have demonstrated one-pot sequential D*o*M and HalD of ArOAm **28o** forming either the tetrasubstituted **108** via HalD followed by electrophile quench or substituted phenol **109** via an *ortho*-Fries rearrangement (in the absence of an electrophile) (Scheme 18). Compound **110** was initially prepared by D*o*M of substrate **28o** with I_2_ as electrophile in 88% yield (Sanz et al.^199^).

**Scheme 19:**
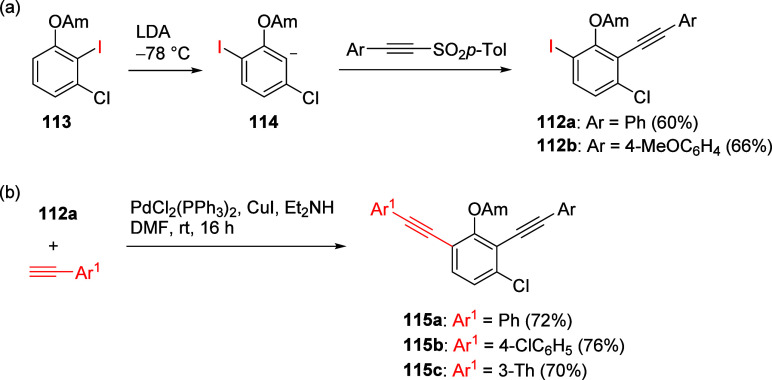
(a) Synthesis of Alkynyl-5-chloro-2-iodophenyl Carbamates **112**, and (b) Their Application in Cross-Coupling (CC) Reactions with Terminal Alkynes Adapted from ref (200). Copyright 2022 MDPI.

Feberero et al.^200^ applied this chemistry in the preparation of alkynyl-5-chloro-2-iodophenyl carbamates **112** from dihalogeno-ArOAm **113** via an anionic intermediate (Scheme 19). Such materials are useful in forming *o*,*o*′-dialkynyl carbamates (exemplified by **115a**–**c**) by Sonogashira coupling with select terminal alkynes.

Similarly, Miller et al.^198^ demonstrated the HalD with three iodo-pyridyl *O*-carbamates **119**–**121** (prepared by D*o*M from the corresponding isomeric *O*-carbamate substrates **116**–**118**) to form the trisubstituted pyridyl *O*-carbamates **122**–**124** with a variety of electrophiles (Table 7).

**Table 7:**
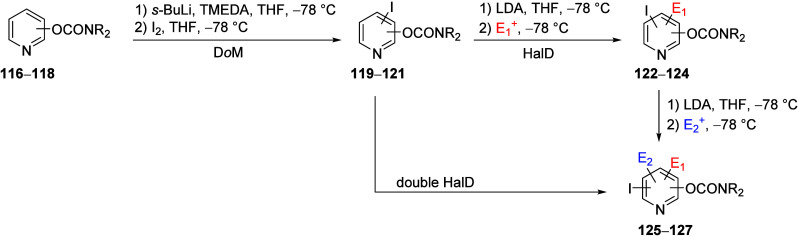

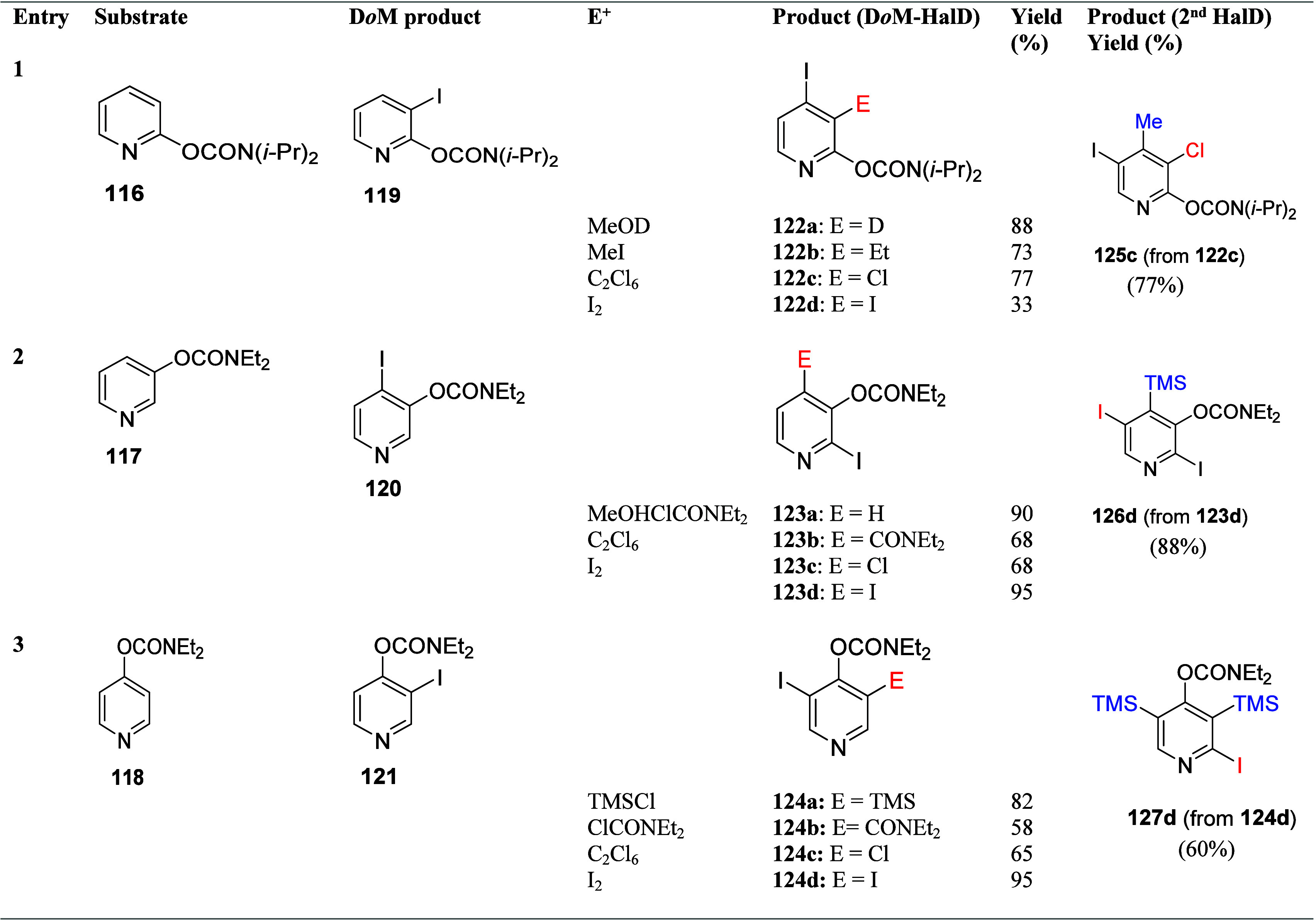
Selected Products of D*o*M-HalD on Isomeric Pyridine *O*-Carbamates (**122**–**124**) and Secondary HalD (**125c**, **126d**, and **127d**)^a^

a Adapted with permission from ref (198). Copyright 2010 John Wiley and Sons.

Subsequent HalD reactions of select products with LDA (2.2 equiv for **122c** and **123d**) and LiTMP (for **124d**) resulted in additional substitution on the aromatic ring, forming compounds **125**–**127**, further demonstrating the synthetic potential of the D*o*M-HalD (and secondary HalD) sequence.

## 6 ArOAm and Silyl Groups

The synthesis of *ortho*-silylated aromatic and heteroaromatic compounds can be facilitated by the *O*-carbamate group, quenching with chlorotrimethylsilane (TMSCl) as electrophile. However, silylated *O*-aryl carbamates themselves have significant utility. Three main approaches lend value to these materials, and each is discussed in detail in the following subsections.

### 6.1 *Ipso*-Substitution of Silyl Groups

**Figure 3:**
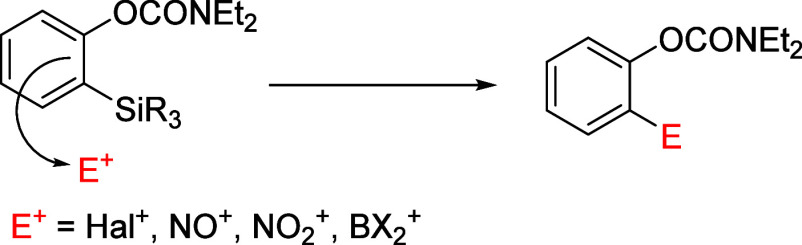
Schematic to show *ipso*-substitution of silylated ArOAms with various electrophiles.

*Ipso*-substitutions (substitutions of attachments other than H on the aromatic ring) of silyl groups can occur via nucleophilic or electrophilic aromatic substitution mechanisms. When the silyl group is positioned *ortho* to a DMG such as the *O*-carbamate group, *ipso*-substitutions can be of significant preparative importance, e.g., for the regioselective synthesis of conventionally inaccessible disubstituted arenes. The *ipso*-desilylation of silylated ArOAms can be halogeno-,^114,201^ boro-,^202^ and nitroso-induced,^203^ introducing a highly expedient approach to halogenoarenes, nitrosoarenes, nitroarenes, and arylboronopinacolates (Figure 3).

**Scheme 20:**
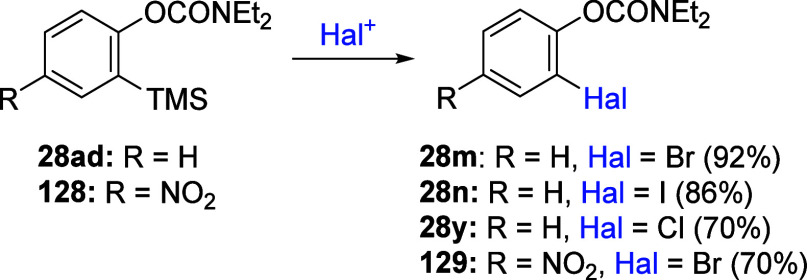
*Ipso*-Desilylation Products of Compounds **28ad** and **128** Adapted from ref (204). Copyright 2005 American Chemical Society.

For example, the treatment of D*o*M-derived silylated aromatics **28ad** and **128** under standard electrophilic halogenation conditions cleanly affords *ipso*-desilylated products **28m**,**n**,**y** and **129**, respectively, in good to excellent yields (Scheme 20).^204^ Similarly, D*o*M-derived silylated naphthalene substrates **130** and **132** afford halogeno *ipso*-desilylated products (Table 8).^205,206^

**Table 8:**
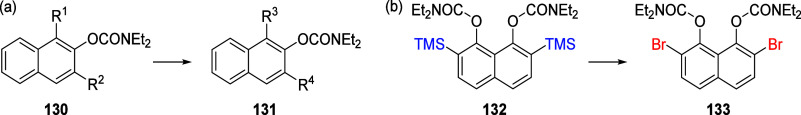
Halo-*ipso*-Desilylation Reactions of Naphthalene Substrates **130** and **132**^a^

entry	compd, R	E^+^ (equiv)	product, R	yield (%)
1	**130**, R^1^ = H; R^2^ = TMS	pyHBr_3_ (2.0)	**131**, R^3^ = H; R^4^ = Br	87
2	**130**, R^1^ = TMS; R^2^ = TMS	pyHBr_3_ (1.0)	**131**, R^3^ = Br; R^4^ = TMS	84
3	**130**, R^1^ = TMS; R^2^ = TMS	ICl (1.0)	**131**, R^3^ = I; R^4^ = TMS	81
4	**130**, R^1^ = I; R^2^ = TMS	Br_2_ (1.1)	**131**, R^3^ = I; R^4^ = Br	96
5	**130**, R^1^ = Br; R^2^ = TMS	ICl (2.5)	**131**, R^3^ = Br; R^4^ = I	96
6	**132**	Br_2_ (2.0)	**133**	87

a Adapted from ref (205). Copyright 2014 American Chemical Society.

Thus, treatment of naphthyl *O*-carbamate from entry 1 with pyridinium tribromide (pyHBr_3_) cleanly afforded the corresponding 3-bromo derivative in 87% yield.^205^ With only 1 equiv of pyHBr_3_, the 1,3-disilylated carbamate from entry 2 gave the corresponding derivative, also in high yield via regioselective 1-bromo *ipso*-desilylation. The authors attribute this to the extended conjugation and greater stabilization afforded by the β-arenium intermediate compared with when the bromonium ion attacks at C3. The same regioselectivity was observed upon iodination of the same substrate to form the 1-iodo derivative (entry 3) in 81% yield. When naphthyl *O*-carbamate **131** from entry 3 (i.e., **130**, R^1^ = I, R^2^ = TMS) was exposed to bromination by Br_2_ in CH_2_Cl_2_ at 80 °C in a microwave (the use of pyHBr_3_ was unsuccessful), the reaction provided the 3-bromo-1-iodo derivative in quantitative yield (entry 4). Conversely, the carbamate in entry 5 actively reacted with excess ICl at −20 °C to provide the inverted isomer of the product found in entry 4 in a high yield. Treatment of the bis(*O*-carbamate) **132** with Br_2_ caused bromodesilylation, providing **133** in 87% yield (entry 6).

**Scheme 21:**
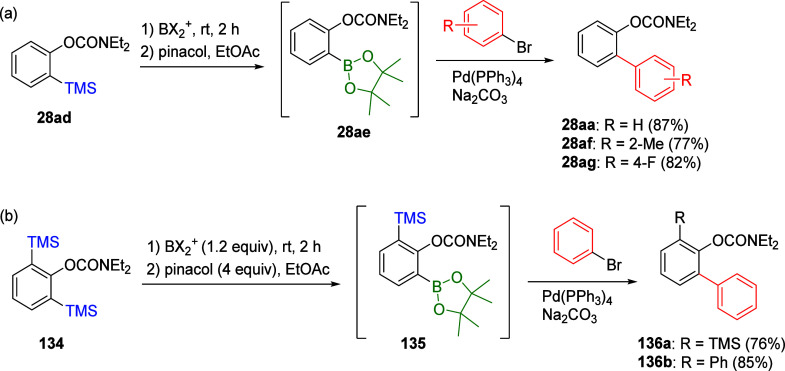
*In Situipso*-Borodesilylation and Suzuki CC of *ortho*-*O*-Carbamate-Substituted Arylsilanes, Commencing with (a) Monosilylated ArOAm **28ad** (Adapted with Permission from ref (202), Copyright 1987 Elsevier), and (b) bisilylated ArOAm **134** (Adapted from ref (204), Copyright 2005 American Chemical Society)

Following halogeno-induced *ipso*-desilylation, the new halogeno-aromatic compound can be treated under a plethora of cross-coupling conditions, e.g., with boronic acids, to obtain a versatile range of biaryl compounds. Similarly, but more advantageous, combining the *ipso*-borodesilylation procedure (Figure 3, E^+^ = BX_2_^+^) with Suzuki–Miyaura cross-coupling (CC) chemistry constitutes an *in situ* protocol to unsymmetrical biaryls and heterobiaryls and *m*-terphenyls.^202,204^ For example, sequential *ipso*-borodesilylation of **28ad** to **28ae** followed by treatment with aryl halides under Pd-catalyzed conditions provides biaryl compounds **28aa**,**af**,**ag** (Scheme 21a).

In the case of bisilyl **134**, reaction with 1.2 equiv of BX_2_^+^ and subsequent excess of pinacol leads to the formation of **135**, which can form either **136a** or **136b** when reacted with bromobenzene under Suzuki coupling conditions (**136b** forms when 2 equiv of bromobenzene are used) (Scheme 21b).^204^

**Scheme 22:**
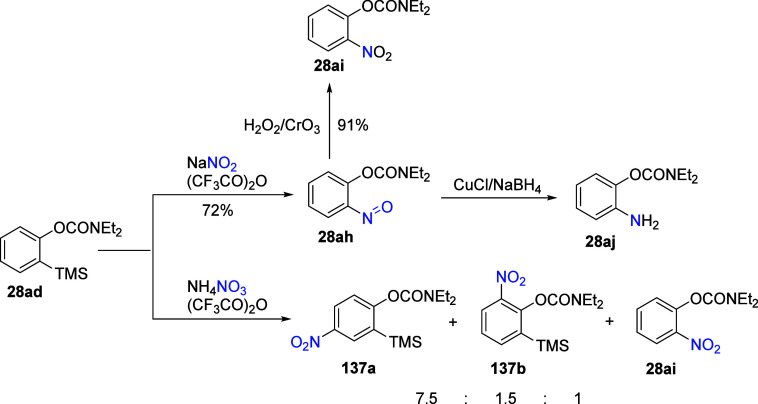
*ipso*-Nitrosodesilylation of **28ad** to Nitroso **28ah**, Which Can Either Be Oxidized to **28ai** or Reduced to **28aj**. Under Mild Nitration Conditions, **28ad** Instead Forms a Mixture of **137a**, **137b**, and **28ai** Adapted from ref (204). Copyright 2005 American Chemical Society.

Zhao and Snieckus^204^ described the nitroso-induced desilylation of the *ortho*-silylated *O*-carbamate **28ad** to the *ipso*-nitroso product **28ah** in a good (72%) yield with sodium nitrite and trifluoroacetic anhydride (Scheme 22).

Compound **28ah** was then either oxidized to nitrobenzene **28ai** or reduced to aniline **28aj** under standard conditions in good yields. Interestingly, exposing **28ad** instead to mild nitration conditions (ammonium nitrate) led to the formation of the corresponding *para*-, *ortho*-, and *ipso*-nitro-substituted products, **137a**, **137b**, and **28ai**, respectively, in a ratio of 7.5:1.5:1, via a mix of non-*ipso*-electrophilic substitution and nitro-induced *ipso*-desilylation.

The silyl group can also be used in CC, opening the final products of the above chemistry to further manipulation, although we only found one example of this: in the formation of *ortho*-aryl silanes.^207^ This is an area ripe for further exploration.

### 6.2 Silyl-Mediated Non-*ipso*-Substitutions

**Figure 4:**
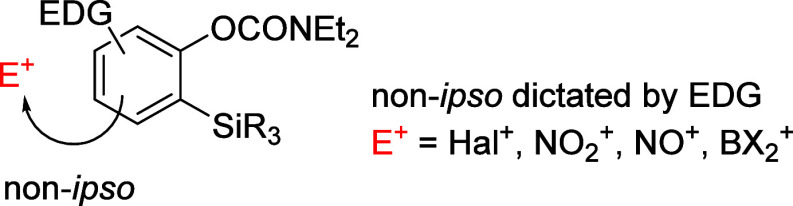
Schematic to show non-*ipso*-substitution with various electrophiles as directed by silylated ArOAms.

In the presence of electron-donating groups (EDGs) along the aromatic component, the steric effect of the silyl groups instead promotes non-*ipso*-electrophilic substitution (particularly with E^+^ = NO_2_^+^), as shown in Figure 4 (compare with Figure 3).

**Scheme 23:**
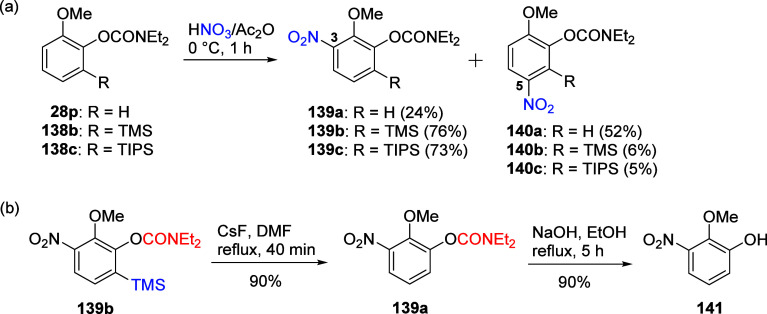
(a) Nitration of *O*-Aryl Carbamates **28p** and **138b**–**c** to Form a Mixture of Isomers **139** and **140**; (b) Synthesis of 2-Methoxy-3-nitrophenol (**141**) via Removal of TMS From **139b** to Form **139a**, Followed by Hydrolysis of the *O*-Carbamate Group Adapted from ref (204). Copyright 2005 American Chemical Society.

For example, the nitration of methoxy substituted **28p** and **138** leads to corresponding non-*ipso* isomers **139** and **140** (Scheme 23a).^204^

Compared with the unsubstituted **28p**, TMS- and TIPS-substituted *O*-carbamates **138b** and **138c** favor electrophilic nitration at C3 to a greater extent, as dictated by a silicon steric effect, forming **139b** and **139c**, respectively.^204^ To demonstrate further utility of this chemistry, the silyl group of **139b** can then be removed (by F^–^) to form **139a** and the *O*-carbamate functionality hydrolyzed to −OH to form 2-methoxy-3-nitrophenol (**141**, CAS no. 20734-71-8, costs USD188/0.1 g) (Scheme 23b), otherwise only accessible via a fastidious, lengthy (5 step) and low yielding protocol.^208^

### 6.3 Silyl Groups as Blocking Groups

**Scheme 24:**
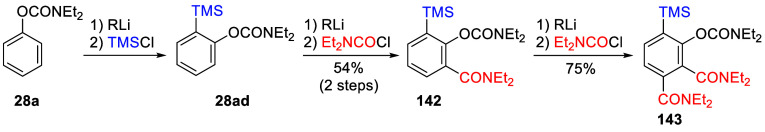
Utilization of the Silyl Group (TMS) as a Blocking Group as the OAm Group Directs the Lithiation Followed by E^+^ (Et_2_NCOCl) Quench *ortho* to the OAm Group to Form **142** and *ortho* to the Am Group to Form **143** Adapted with permission from ref (18). Copyright 2018 John Wiley and Sons.

Silyl groups can also be used as blocking groups that prevent metalation of an ArOAm *ortho*-site during the D*o*M method. *Ortho*-silyl and -siloxane substituted ArOAms can be utilized for the regiospecific synthesis of specific *ortho*-substituted carbamates.^18,209^ For example, Miah et al.^18^ demonstrated the sequential metalation, silylation, and carbamoylation (with equivalent amounts of base and electrophile and the reaction temperature maintained at −78 °C) of *O*-phenyl *N*,*N*-diethylcarbamate (**28a**), through **28ad**, to obtain the trisubstituted **142** in reasonable yield (Scheme 24).

If **142** is isolated, purified, and subjected to standard conditions for deprotonation before being treated with ClCONEt_2_, the contiguously tetrasubstituted aromatic molecule **143** is formed in 75% yield. Treatment with F^–^ (e.g., as CsF or TBAF) enables the facile removal of the TMS group due to the formation of the strong Si–F bond compared with the C–Si bond.

**Scheme 25:**
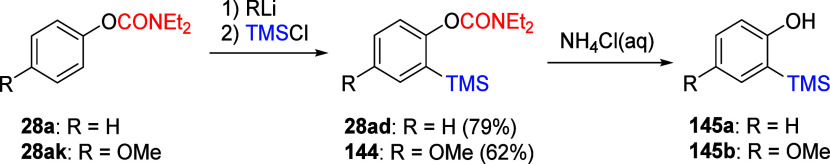
Synthesis of *ortho*-TMS Phenols **145a** and **145b** via Base-Catalyzed Hydrolysis of ArOAms **28ad** and **144**, Respectively Adapted from ref (17). Copyright 1983 American Chemical Society.

Because the *O*-carbamate functionality can be hydrolyzed to OH, silylated *O*-carbamates are also useful for the regiospecific synthesis of *ortho*-substituted phenols. For example, **145a** and **145b**, can be prepared by the initial formation of carbamates **28ad** and **144** from **28a** and **28ak**, respectively, prior to base-catalyzed carbamate removal as shown in Scheme 25.^17^

Reductive cleavage by the Schwartz reagent is another recently reported methodology for conversion of ArOAms to phenols.^184^ The manipulation of the ArOAm functionality forms the focus of section 7 (reductive cleavage and other methodologies will be discussed in more detail in section 7).

**Scheme 26:**
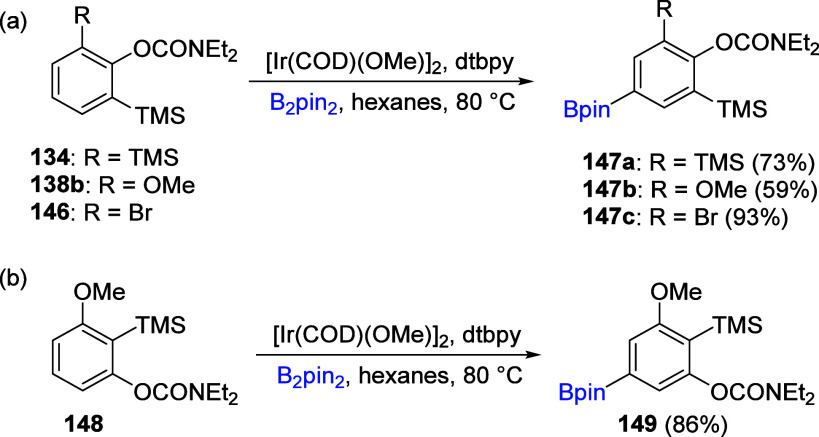
Ir-Catalyzed Borylation of 1,2,3-Trisubstituted Silylated ArOAms **134**, **138b**, **146**, and **148** Adapted with permission from ref (210). Copyright 2010 John Wiley and Sons.

Silyl groups also play a significant role in the Ir-catalyzed borylation of ArOAms in which 1,3-disubstituted systems generally lead to *meta*-borylation with high regioselectivity and efficiency.^210^ For example, in Ir-catalyzed borylation of selected 1,2,3-trisubstituted silylated ArOAms **134**, **138b**, **146**, and **148**, all derived by efficient D*o*M chemistry, borylated products **147a**–**c**, and **149** could be obtained in synthetically useful yields (Scheme 26).

**Scheme 27:**
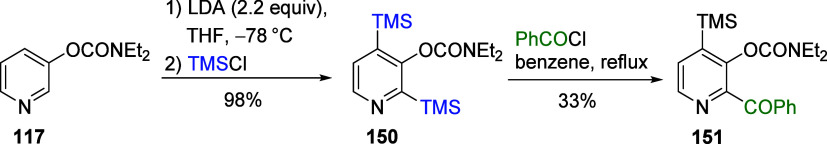
Synthesis of 2,4-Disilylated Derivative **150** Followed by a Mono *ipso*-Carbodesilylation to Form **151** Adapted from ref (211). Copyright 1993 The Japan Institute of Heterocyclic Chemistry.

Silyl groups as *ortho*-blocking groups during D*o*M is also especially useful for promoting *ortho*-substitution in π-electron deficient heteroaromatics such as pyridine via D*o*M chemistry where such substrates with low-lying LUMO molecular orbitals typically undergo nucleophilic attack by RLi reagents.^211^ For example, the deprotonation of *O*-3-pyridyl carbamate (**117**) can be achieved by adapting the blocking tactic: when **117** was subjected to 2.2 equiv of LDA followed by treatment with excess TMSCl, the 2,4-disilylated derivative (**150**) was obtained in quantitative yield (Scheme 27). Treatment of **150** with a suitable electrophile (e.g., benzoyl chloride) resulted in mono *ipso*-carbodesilylation to form **151**.

If 4-(trimethylsilyl)-3-pyridinyl carbamate (**152**) is treated with 1.2 equiv of LiTMP followed by a suitable electrophile, a variety of 2-substituted pyridine derivatives (including **153a**–**f** and **154a**–**f**) can be formed (Table 9), taking advantage of the C4 protection by a silicon group.^211^ The silyl group can then be removed through the addition of F^–^ (due to the formation of the strong Si–F bond resulting in desilylation)^212^ to form **154**.

**Table 9:**
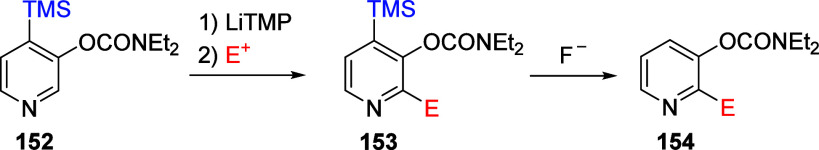
Synthesis of Pyridine Derivatives (**153** and **154**) Using LiTMP as Base and TMS as PG^c^

entry	method^a^	E^+^	product	E	yield (%)
1	A	CD_3_OD	**153a**	D	87^b^
2	A	PhCHO	**153b**	CH(OH)Ph	82
3	A	PhCOCl	**153c**	COPh	41
4	B	CCl_3_CCl_3_	**153d**	Cl	64
5	B	(PhS)_2_	**153e**	SPh	79
6	B	Et_3_SiCl	**153f**	SiEt_3_	89

a Method A: 1.5 equiv of LiTMP. Method B: 1.2 equiv of LiTMP.

b 58% D-incorporation (by mass spectrometry).

c Adapted with permission from ref (211). Copyright 1993 The Japan Institute of Heterocyclic Chemistry.

**Scheme 28:**
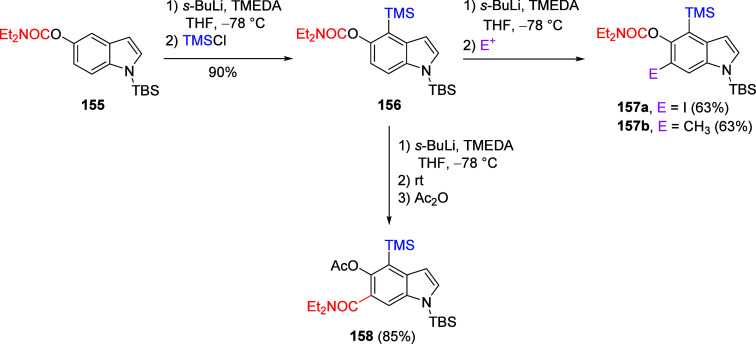
Silylation of **155** to Form **156**, Which Is Treated under Standard Metalation Conditions Followed by Quenching with I_2_ or Mel to Give **157** Adapted from ref (213). Copyright 1995 American Chemical Society. When **156** undergoes A*o*F followed by acylation, **158** is afforded in 85% yield.

This tactic can also be used to facilitate 4- and 6-substitution of π-excessive heterocycles such as indoles.^213^ For example, *N*,*N*-diethylindole 5-*O*-carbamate can be silicon protected as **155** and further protected as **156**; when **156** is subjected to metalation and quenched with electrophiles, the 6-substituted products **157** are provided (Scheme 28). When quenched with iodine, **157a** is formed in 63% yield, as is **157b** (quench with iodomethane). Under standard conditions^17^ followed by acylation, **158** is afforded via the A*o*F rearrangement. Both products (**157** and **158**) are amenable to further metalation chemistry.

**Scheme 29:**
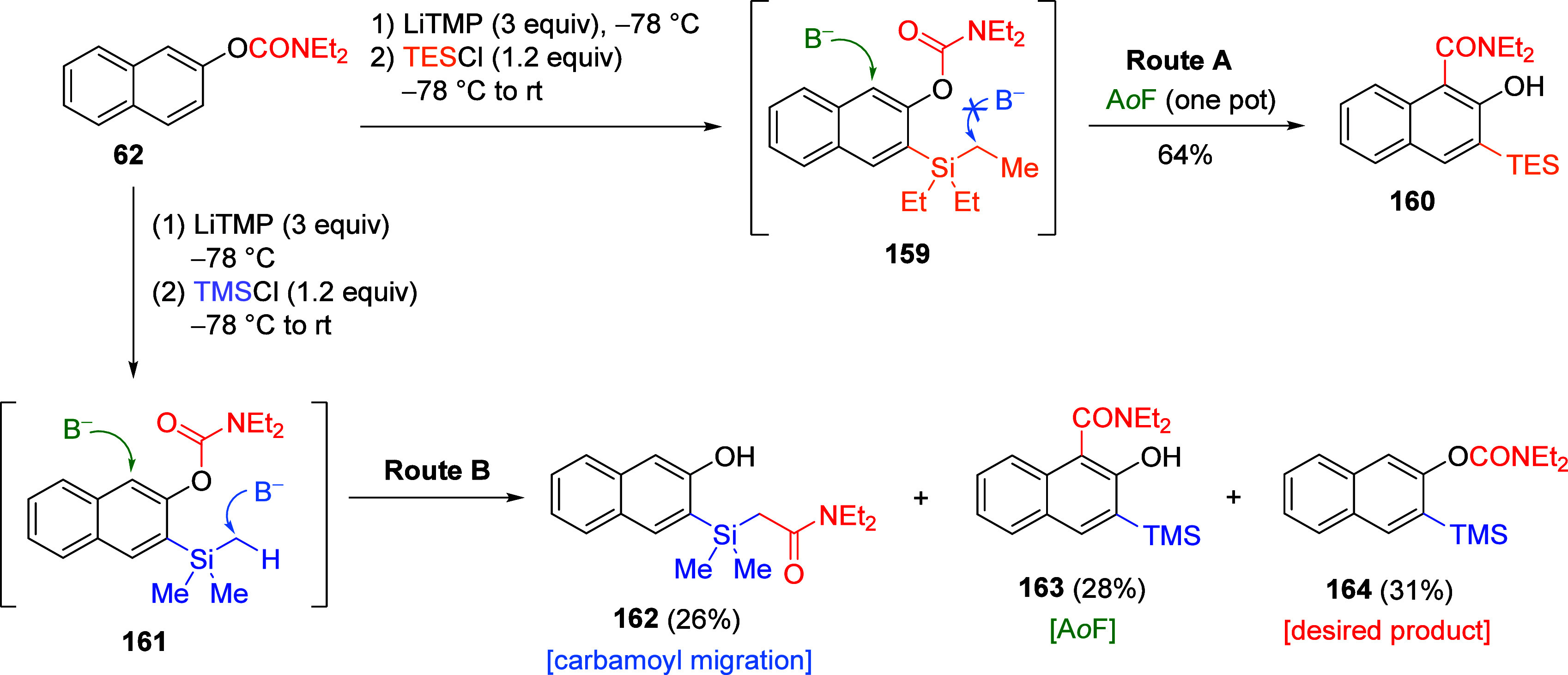
Results of A*o*F Rearrangements When Silyl Blocking Group Is Either TES (Route A) or TMS (Route B) Adapted from ref (205). Copyright 2014 American Chemical Society.

Another interesting application involving the A*o*F rearrangement was reported by Groom et al. for silylated naphthalene compounds.^205^ As shown in Scheme 29, when silylated compound **159** (prepared by D*o*M of substrate **62**, followed by quench with TESCl) is allowed to warm to room temperature instead of electrophile quench, **160** forms via a “sequential C3-deprotonation–silylation, C1-deprotonation–A*o*F rearrangement”.^205^ When the same reaction of **62** was carried out with TMSCl instead of TESCl, the analogous product **163** forms in 28% yield along with the desired product **164** (31%) and compound **162** (26%), the result of “CIPE-induced α-silyl methyl deprotonation–carbamoyl migration”. The greater acidity of the α-methyl of the TMS groups combined with a possible steric effect may be the reason for the observed difference in reactivity between intermediates **159** and **161**.

## 7 OAm Manipulation

The *O*-carbamate functionality is synthetically valuable due to its relative stability and capability as a directing group as well as its ability to be converted into other functional groups. This differs from the cross-coupling of ArOAms which is covered in section 8. We discuss 10 useful transformations in this section, commencing with the most widely investigated conversion: reductive cleavage of the *O*-carbamate group (section 7.1). Other transformations include A*o*F-product manipulation (section 7.2), decarboxylation of ArOAms to form aromatic amines (section 7.3), conversion of *N*,*O*-diaryl carbamates into ureas (section 7.4), ArOAms to ArOCONRCHO (section 7.5) and triflates (section 7.6), borylation of ArOAms (section 7.7), and sulfonamidation (section 7.8), amidation (section 7.9) and cyanation (section 7.10) of ArOAms.

### 7.1 Cleavage of ArOAm

#### 7.1.1 Reductive Cleavage to Phenols

The reductive cleavage of ArOAms by the Schwartz reagent (Cp_2_Zr(H)Cl) in the synthesis of polysubstituted phenols (method F)^184^ employs relatively mild conditions compared with methods A–E (Table 10).

**Table 10:**
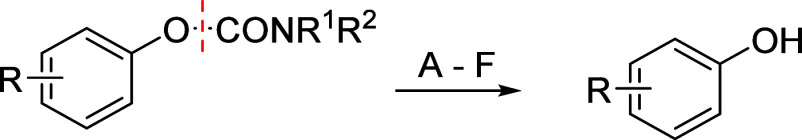
Methods for Cleavage of ArOAms to Phenols

method	reagents	ref
A	NaOH (large excess), EtOH, reflux (5–8 h)	Sanz et al. 2005;^214^ Zou et al. 2009^215^
B	DBU, Et_2_NH, MeCN, rt	Yoshida et al. 2014;^216^ Medina et al. 2014^217^
C	LiAlH_4_, THF, reflux^a,b^ LiAlH_4_, Et_2_O, reflux^c,d^	^a^Schön et al. 2012;^218^^b^Sibi et al. 1983^17 c^Yamazaki et al. 2010;^219^^d^Zhang et al. 2019^220^
D	cat. Rh/*i*-PrOH, toluene, K_3_PO_4_, 180 °C, 4 h	Yasui et al. 2017^221^
E	TFA (0 °C), 6 min (rt)	Metallinos et al. 1999^116^
F	Cp_2_Zr(H)Cl (Schwartz reagent), THF, rt, 2–12 h	Morin et al. 2013;^184^ Zhao et al. 2014^222^

Method F was based on the reduction procedure initially implemented by Spletstoser et al. in the reduction of amides to aldehydes.^223^ Under conditions using commercially acquired Schwartz reagent (3 equiv), an assortment of ArOAms afforded the corresponding phenols in moderate to excellent yields (Table 11).^185^

**Table 11:**
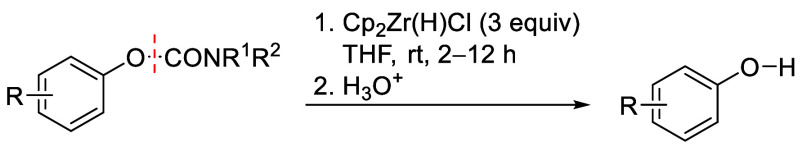

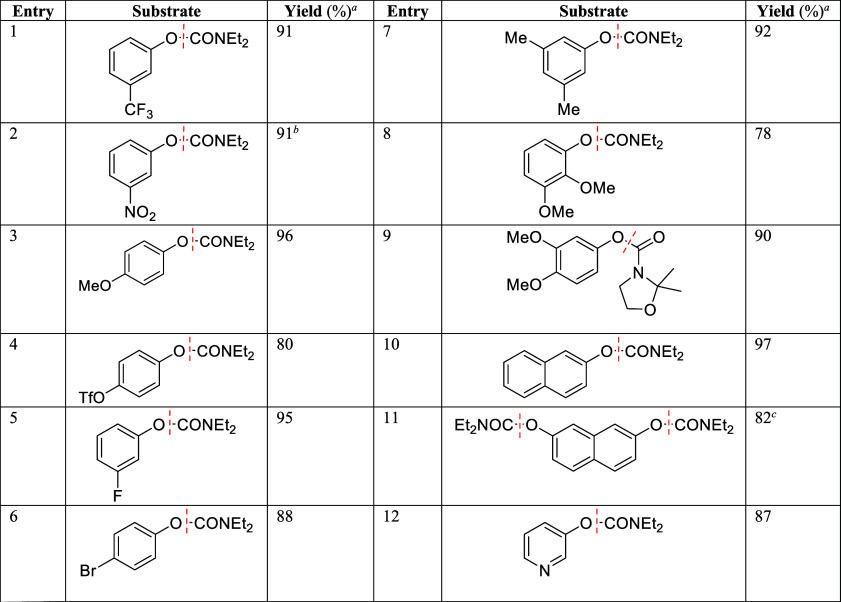
Schwartz Reagent Mediated Reduction of Select ArOAms Using the Georg Procedure^d^

a Yields of isolated products.

b 5 equiv.

c 6 equiv of Schwartz reagent required for full conversion.

d Adapted from ref (185). Copyright 2013 American Chemical Society.

Moreover, Zhao et al. developed economically valuable *in situ* generation of Schwartz conditions for the ArOCONR_2_ → ArOH transformation (see Table 12).^222,224^ The demonstrated *in situ* reduction method is highly effective, accomplished in several hours (TLC analysis), to provide good yields of isolated phenol products. When compared with the direct Schwartz reagent reduction, the *in situ* method is an equally efficient and less expensive practical approach for the reductive cleavage of aromatic *O*-carbamates to phenols.

**Table 12:**
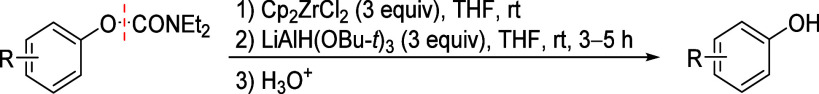

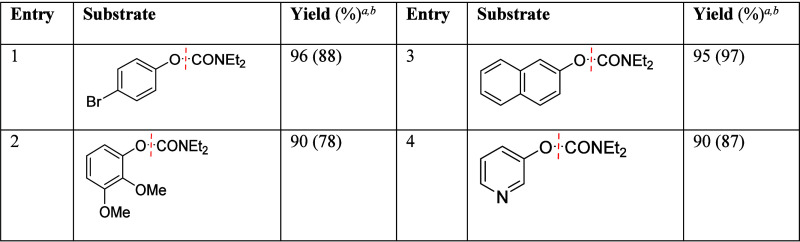
Reduction of Selected ArOAms to Phenols Using *in Situ* Generated Schwartz Reagent^c^

a Yields of isolated products.

b Yields in parentheses refer to direct Schwartz reduction; see Table 11.

c Adapted from ref (222). Copyright 2014 American Chemical Society.

**Figure 5:**
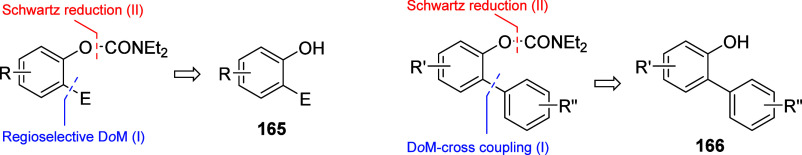
Synthetic potential of combined ArOAm D*o*M–Schwartz reduction chemistry.

Both direct (Spletstoser et al.^223^) and the greatly more economical *in situO*-carbamate reduction methods comprise practical procedures for the synthesis of a variety of phenols, especially costly and less available ones, such as 3,5-disubstituted phenols.^225^ Future valuable synthetic opportunities may be anticipated with combined D*o*M–Schwartz reduction and D*o*M–Suzuki CC–Schwartz reduction practices for the assembly of challenging-to-obtain multisubstituted phenols of type **165** or **166** (Figure 5).

A useful variation of *O*-carbamate D*o*M chemistry encompasses the *in situ* protection of secondary carbamates **167** (against the known fragmentation to phenols and isocyanates) by TMSOTf/TMEDA as the *N*-silylated species **168**, which, upon sequential treatment with *s*-BuLi and electrophile (E^+^) to the *ortho*-conditions, accomplishes, through **169**, provision of the phenols **170** (Table 13).^201,226^

**Table 13:**
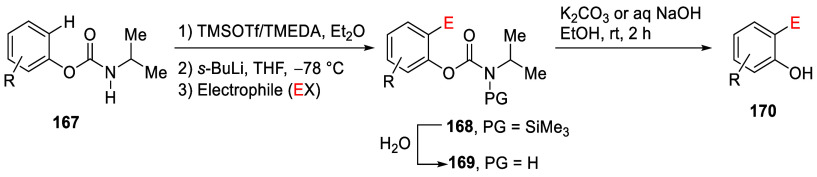

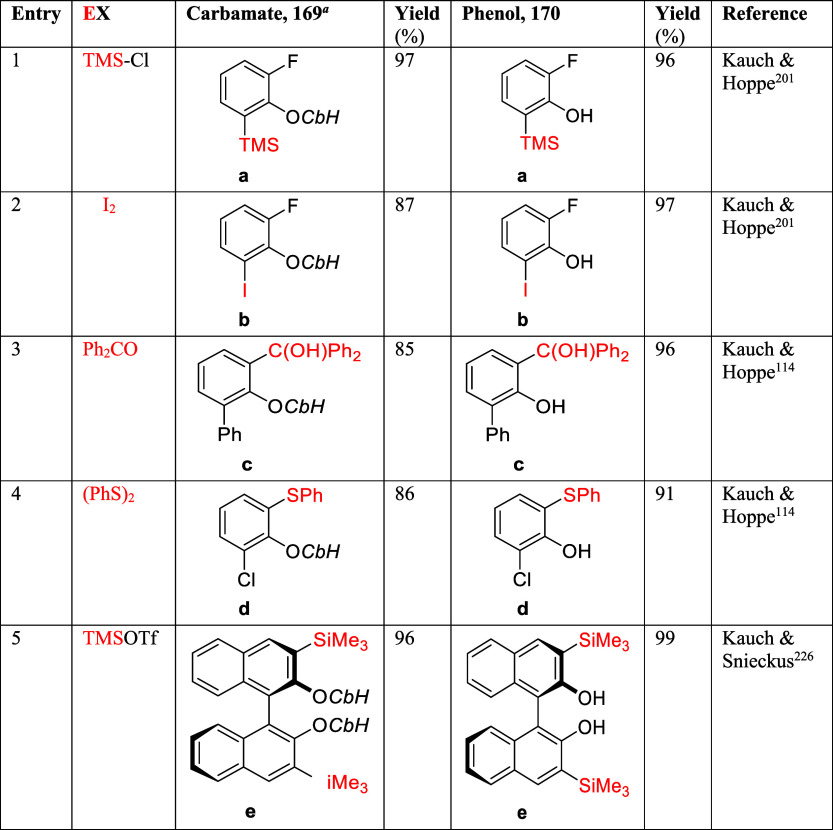
*In Situ* TMS Protection and D*o*M of **167** Followed by Electrophile Quench (to Form **168**), Deprotection of the *N*-Silyl Group (to **169**), and Hydrolytic Cleavage of the Aryl-*O*,*N*-Isopropylcarbamate Functionality to Form Phenols **170**

a *CbH* = CONH*i*-Pr.

Of particular interest was the use of this chemistry in the preparation of 3,3-disubstituted 2,2-dihydroxy-1,1-binaphthyls, frequently used as chiral ligands in enantioselective catalysis.^227^ In this case, the *N*,*N*-bis(trimethylsilyl)diurethane was reacted with *s*-BuLi/TMEDA before being quenched with excess TMSCl to form **169e** in 96% yield (entry 5). Subsequent carbamate cleavage using aqueous NaOH gave the bis-silylated BINOL derivative **170e** [the I-enantiomer] in 99% yield.^226^

**Scheme 30:**
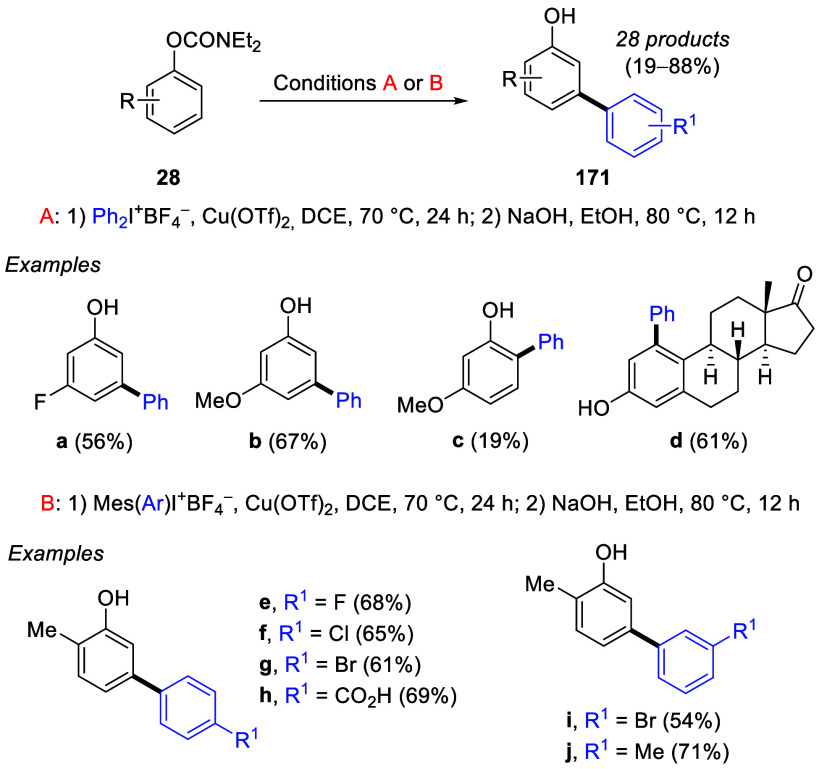
Scope of *meta*-Arylation of ArOAms Adapted from ref (228). Copyright 2021 American Chemical Society.

This chemistry was also useful when combined with the *meta*-arylation of ArOAms, enabling an easily accessible route to *meta*-aryl phenols (Scheme 30).^228^ Electron-donating and electron-withdrawing substrates were all well tolerated, generating the desired products (**171a**,**b**,**e**–**j**) in good yields. An *ortho*-arylated product (*para* to the OMe group), **171c**, was obtained in 19% yield as a side-product in the reaction to form **171b**. The authors attributed this to the strong influence exerted by the ArOAm group over the OMe group in directing the incoming aryl moiety. Compound **171d** was produced by exclusive *meta*-arylation of naturally occurring estrone.

#### 7.1.2 Reductive Removal of OAm

**Scheme 31:**
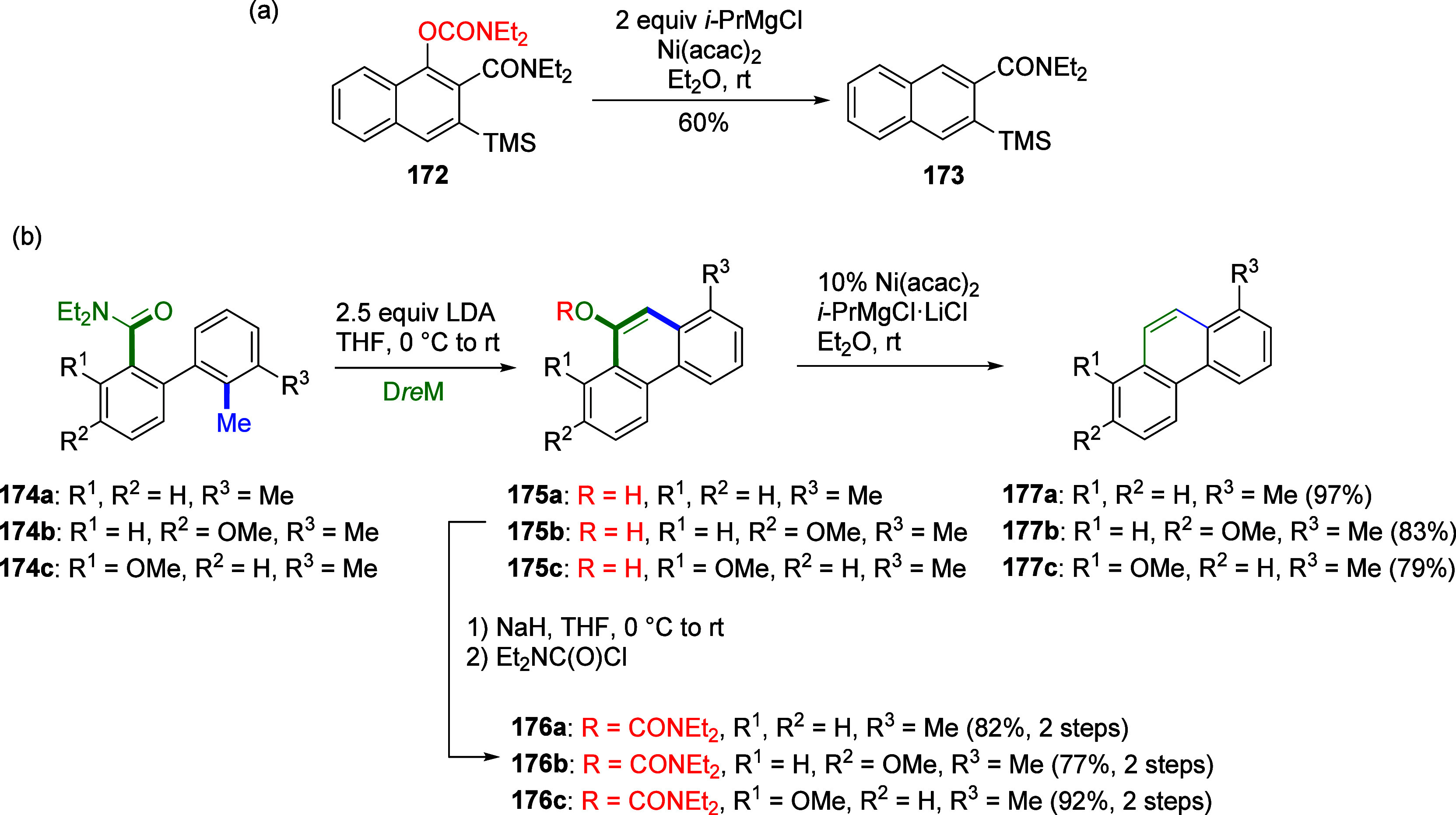
(a) Reductive Cleavage of **172** to **173** upon Treatment with *i*-PrMgCl/Ni(acac) (Adapted from ref (229), Copyright 1992 American Chemical Society) and (b) Transformation of Biaryls **174** through **175** and **176** to Phenanthrenes **177** (Adapted from ref (230), Copyright 2015 American Chemical Society)

ArOAms can also be reductively cleaved such that the *O*-carbamate group is removed completely. Sengupta et al.^229^ demonstrated this in 1992 for the first time by reductively cleaving **172** to **173**, using a Ni(0) catalyst and *i*-PrMgX as reducing agent (Scheme 31a). This was subsequently extended to the synthesis of phenanthrenes from biaryl amides (exemplified by **174a**–**c**), in which application of the turbo-Grignard reagent (*i*-PrMgCl·LiCl) in the Ni-catalyzed Corriu–Kumada reaction enabled efficient reductive decarbamoylation to form compounds **177a**–**c** (Scheme 31b).^230^

Later, Mesganaw and co-workers found that reductive cleavage at **178** occurred efficiently using catalytic amounts of NiCl_2_(PCy_3_)_2_ together with 1,1,3,3-tetramethyldisiloxane (TMDSO) and K_3_PO_4_ in toluene at 115 °C, affording arenes **179** (both fused and nonfused aromatics) in reasonable yields (Table 14).^231^ For example, entry 2 shows a convenient approach to the preparation of borylated indoles (**179b**), while entry 3 demonstrates that fused aromatics were excellent substrates, as **178c** gave rise to product **179c** via a double-decarbamoylation in 82% yield.

**Table 14:**
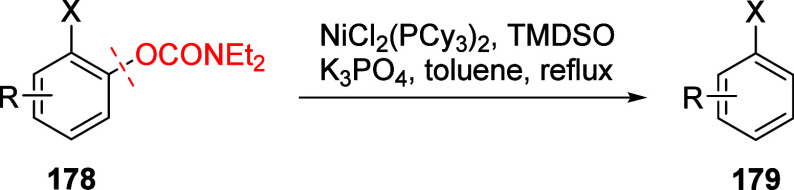

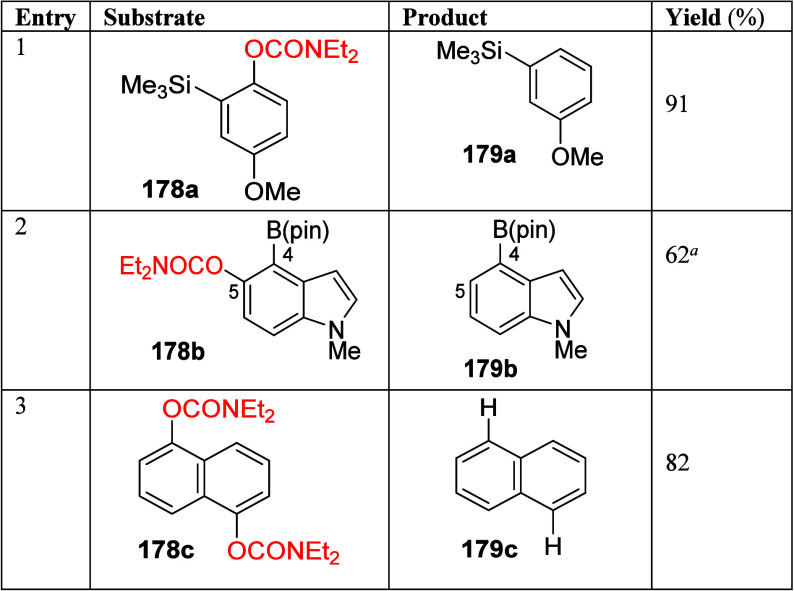
Select Examples of ArOAms **178** Undergoing Ni-Catalyzed Cine Substitution to **179**^b^

a 62% yield over 2 steps (D*o*M-borylation followed by cine substitution).

b Adapted from ref (231). Copyright 2012 American Chemical Society.

**Scheme 32:**
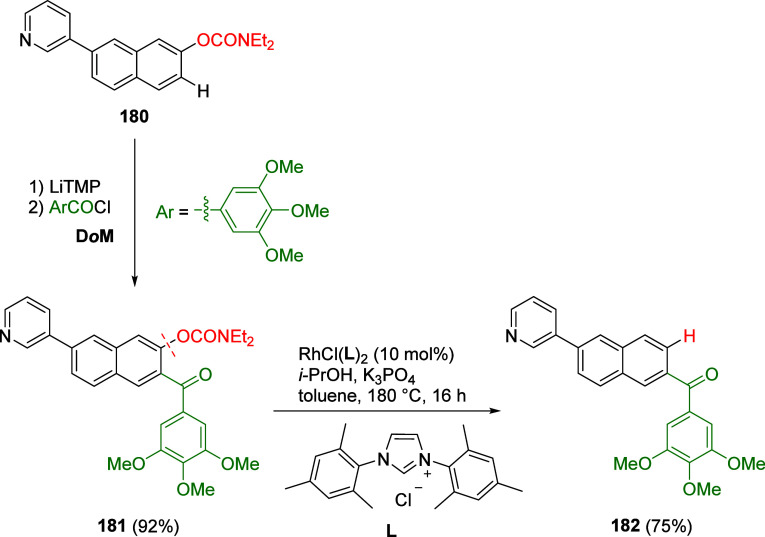
A Synthetic Application of the Reductive Cleavage Adapted with permission from ref (221). Copyright 2017 Thieme.

Yasui et al.^221^ demonstrated an alternative route which employs a rhodium catalyst along with *i*-PrOH as a milder reductant than either *i*-PrMgX or hydrosilane (which react with functional groups such as ketones, pyridyls, and unsaturated bonds). This reductive cleavage method extends the utility of the ArOAm group to the synthesis of multiply functionalized arenes, as exemplified by the synthesis of compound **182** via a combined D*o*M-reductive cleavage sequence (Scheme 32). However, from a green chemistry perspective, this new approach still operates under relatively harsh conditions (temperature) and utilizes a nonrecycled precious metal catalyst.

### 7.2 A*o*F Rearrangement-Product Manipulation

The use of the A*o*F rearrangement (section 4) provides various opportunities for one-pot conversions of the ArOAm group to other functional groups, including *o*-hydroxyacetophenones (**67**) and 2′-hydroxychalcones (**68**) via derivatives of *N*,*N*-diethyl-2-hydroxybenzamides, **66** (Scheme 14). The reader is referred to section 4.1 for more details.

In addition, the A*o*F rearrangement products **183**, upon mild lithium aluminum hydride (LAH) reduction, affords 2-(aminomethyl)phenols **184a**–**j** in yields ranging from 65% to 81% (Table 15).^232^ This provides an alternative facile route to the classical formaldehyde-amine reaction with phenols to prepare these Mannich bases,^233−235^ which, as incipient *ortho*-quinodimethide species, have seen considerable use for Diels–Alder cycloaddition chemistry.^236−238^

**Table 15:**
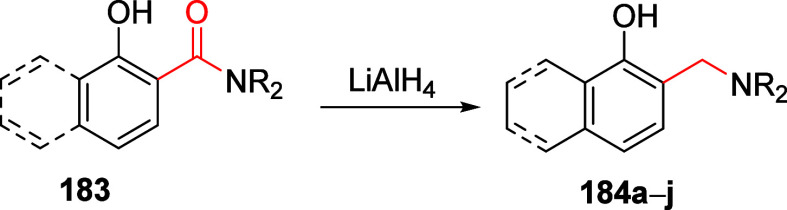

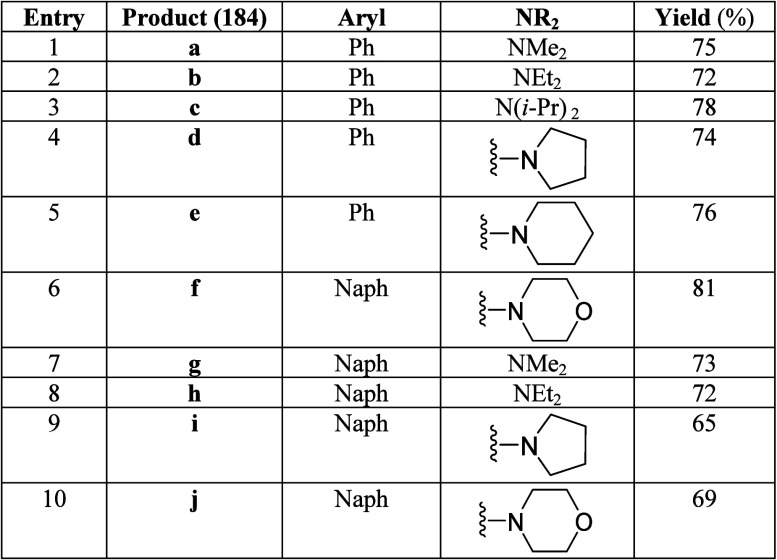
Reduction of the A*o*F Rearrangement Products to Mannich Bases^a^

a Adapted with permission from ref (232). Copyright 2009 Thieme.

### 7.3 Decarboxylation of ArOAm to Form Aromatic Amines

**Scheme 33:**
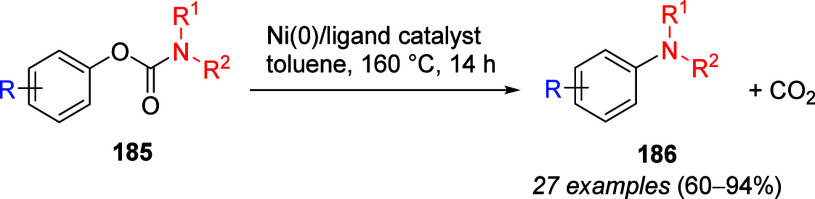
Nickel-Catalyzed Decarboxylation of ArOAms **185** to Amines **186** Adapted from ref (239). Copyright 2019 American Chemical Society.

Nishizawa and co-workers^239^ demonstrated that ArOAms **185** can be decarboxylated to form aryl amines **186** using a nickel catalyst and a bisphosphine ligand immobilized on a polystyrene support, producing CO_2_ gas as a byproduct (Scheme 33). The ligand is key to the success of this reaction, as it generates a catalytic species that is significantly more active than nonsupported variants by enabling more control over the metal complexation by the bisphosphine unit (suppressing any undesired comproportionation, which is initiated when two metal centers collide). This procedure tolerates a range of functionalities (including ketones) as it proceeds in the absence of free amines but has only been demonstrated for the formation of tertiary amines.

To prepare primary and secondary amines from ArOAms, the protocol involves nickel-catalyzed amination of ArOAms with ammonia and primary amines, respectively.^240−242^ More details concerning this latter chemistry are included in section 8.5.

### 7.4 Conversion of *N*,*O*-Diaryl Carbamates into Ureas

Yamasaki et al.^243^ developed a method to transform *N*,*O*-diaryl carbamates directly and spontaneously into *N*,*N*′-diarylureas (Table 16). The Et_3_N catalyzes the reaction by abstraction of the carbamate proton, forming the aniline derivative, which can then react with more of substrate **187** to form diarylurea **188**.

**Table 16:**
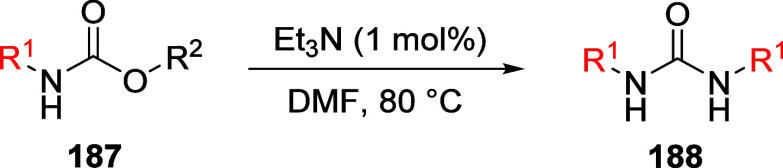
Select Examples of *N*,*O*-Diaryl Carbamates **187** Converted into Ureas **188** Based on the Method Developed by Yamasaki et al.^243^^a^

entry	**187**	R^1^	R^2^	yield (%)
1	a	4-nitrophenyl	C_6_H_5_	87
2	b	4-methoxyphenyl	C_6_H_5_	75
3	c	benzyl	C_6_H_5_	0
4	d	C_6_H_5_	4-nitrophenyl	91
5	e	C_6_H_5_	4-methoxyphenyl	80

a Reproduced with permission from ref (243). Copyright 2018 Pharmaceutical Society of Japan.

### 7.5 Conversion of ArOAm to ArOCONRCHO

**Scheme 34:**
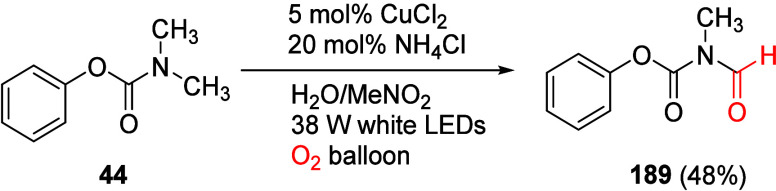
Simple and Economical Photoredox Oxidative Functionalization of Terminal C–H Bond in *O*-Carbamate **45** Adapted with permission from ref (244). Copyright 2022 Royal Society of Chemistry.

Lian et al.^244^ established a simple and economical CuCl_2_-photoredox system for the oxidative functionalization of a wide range of nonreactive amine derivatives, including carbamate **44** (Scheme 34). This method utilizes molecular oxygen for the oxidation of the terminal C–H bond and an inexpensive catalyst system under exceptionally mild conditions, complementing current methods that require stoichiometric toxic and expensive reagents.

### 7.6 Conversion of ArOAm to Triflates

**Scheme 35:**
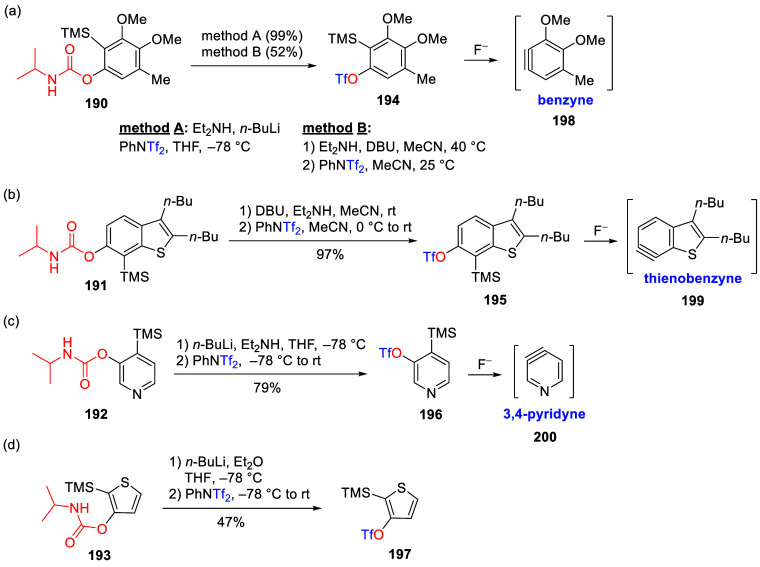
Selected Examples of One-Pot Transformation of ArOAm Group to Aryl Triflate as Demonstrated in (a) Substituted Arenes **190** (Adapted with Permission from ref (247), Copyright 2018 American Association for the Advancement of Science); (b) benzo[*b*]thiophenes **191** (Adapted with Permission from ref (246), Copyright 2018 Royal Society of Chemistry); (c) Pyridines **192** (Adapted with Permission from ref (248), Copyright 2013 Springer Nature)^248^ and (d) Thiophenes **193** (Adapted from ref (249), Copyright 2021 American Chemical Society); Structures of Selected Arynes That Can Be Formed Thereafter Are Also Shown (**198**–**200**)

One approach to convert ArOAms to aryl triflates involves initial cleavage of the *O*-carbamate to the phenol followed by deprotonation of the phenol to form a phenoxide, which then reacts with trifluoromethanesulfonic anhydride or some other analogue of Comin’s reagent.^216,245^ This process can also be performed in one pot.^217,246^ Some examples from the literature for various substrates (substituted arene **190**,^217,247^ benzo[*b*]thiophene **191**,^246^ pyridine **192**,^248^ and thiophene **193**(249)) are shown in Scheme 35a–d, respectively.

Overall, the process occurs via the initial deprotonation of the carbamate NH by a base (e.g., *n*-BuLi), leading to the loss of isopropyl isocyanate, which reacts irreversibly with the diethyl amine to form a urea, leaving a phenoxide (rather than ArOH). After warming, addition of the triflating agent (e.g., PhNTf_2_) gives the ArOTf. This transformation is limited to secondary ArOAms. To triflate tertiary ArOAms, it is necessary to cleave the ArOAm to form a ArOH before applying a triflating agent.^245,250,251^

The synthetic significance of introducing triflates *ortho* to TMS lies in the preparation of arynes. A typical procedure involves OAm-directed regioselective C-silylation via *ortho*-lithiation, removal of the directing OAm group, triflylation, and fluoride-mediated activation. It enabled the preparation of a benzyne,^216,217,245,247^ thienobenzyne,^246,249^ indolyne,^251^ pyridine,^248^ and chromene-type aryne^250^ precursors, for example. The structures of selected arynes (**198**–**200**) are shown in Scheme 35.

### 7.7 Borylation of ArOAm

Aryl, alkenyl, and heteroArOAms could be converted into boronic acid esters by nickel-catalyzed borylation.^252,253^ This was realized using two sets of catalytic systems (with either NaO*t*-Bu or K_3_PO_4_ as complementary base), covering a broad substrate scope, such as is represented by the preparation of neopentyl glycol boronic esters (Bneo) **201** (Table 17).

**Table 17:**
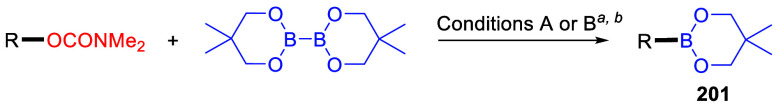

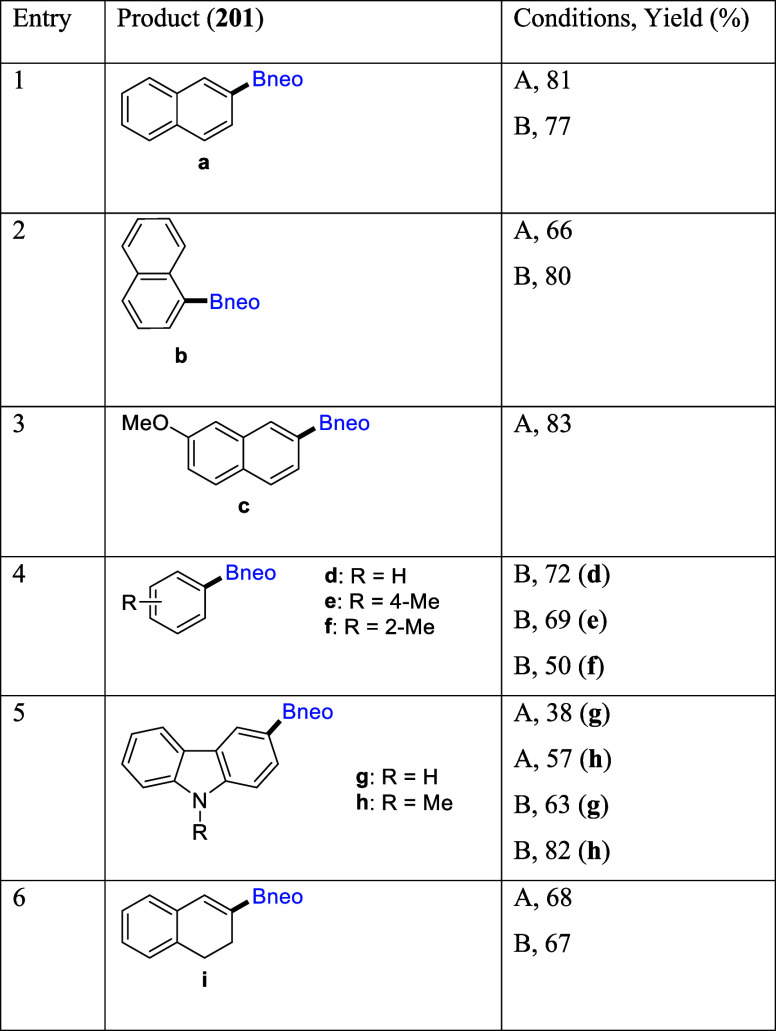
Borylation of Naphthyl, Aryl, and HeteroArOAms Using Two Sets of Systems (Bneo = Neopentyl Glycol Boronic Esters)^c^

a Condition A: [Ni(PCy_3_)_2_Cl_2_] (5 mol %), PCy_3_ (20 mol %), NaO*t*-Bu (1 equiv), 110 °C, 24 h.

b Condition B: [Ni(PCy_3_)_2_Cl_2_] (5 mol %), K_3_PO_4_ (1 equiv), PhMe:DME (1:1), 100 °C, 48 h.^252^

c Adapted with permission from ref (252). Copyright 2011 John Wiley and Sons.

Whereas NaO*t*-Bu gave better yields for naphthyl carbamates, K_3_PO_4_ was preferred for the borylation of phenyl and heteroaryl carbamates, due to effects of steric hindrance (compare the yields of neopentylglycolborylation reactions to give **201a**,**b**,**g**,**h**). Oxygen-containing groups such as MeO (**201c**) were compatible despite having been shown to be very reactive in previously reported CC transformations.^254^ The borylation was extended to alkenyl carbamates to afford **201i**, with both bases enabling similar yields. Steric hindrance as well as electronic properties influenced the efficacy of reactions carried out with different derivatives of 2-naphthol, e.g., borylation of naphthalen-2-yl dimethylcarbamate enabled the borylated product **201a** in 81% yield versus 76% for naphthalen-2-yl diethylcarbamate and 41% for naphthalen-2-yl acetate.

**Figure 6:**
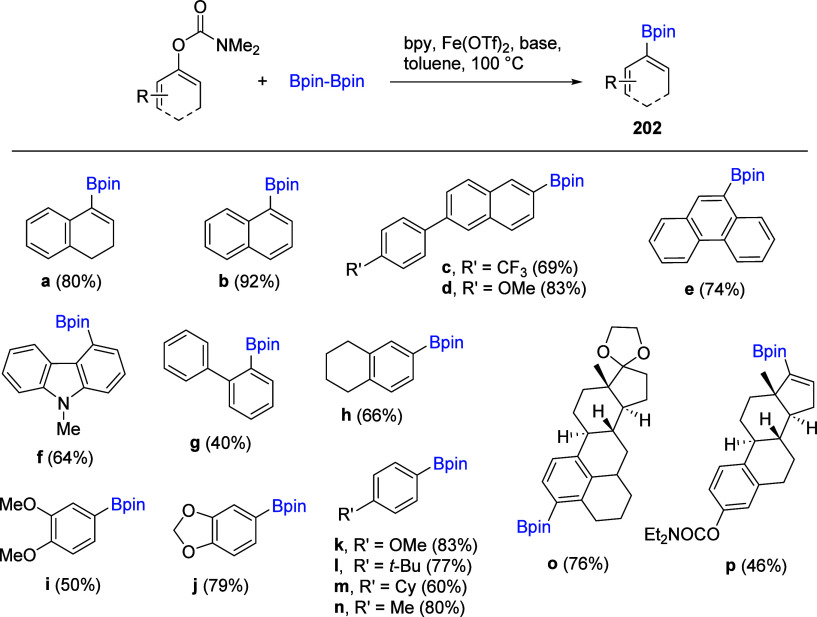
Selected examples of products of the borylation of alkenyl and ArOAms catalyzed by Fe(OTf)_2_ to demonstrate the scope of the chemistry developed by Geng et al.^255^ Adapted from ref (255). Copyright 2020 American Chemical Society.

Using an iron(II) triflate [Fe(OTf)_2_] catalyst to activate the C–O bond, Geng et al.^255^ borylated more than 55 alkenyl and aryl carbamates (and five biomolecules containing the ArOAm functionality). The resulting borylated products could be formed in reasonable yields with good functional group tolerance (e.g., Cl, F, CF_3_, Tips, 2-pyridyloxy, and benzyl ether). Select examples (**202a**–**p**) are shown in Figure 6. These boronic esters can then undergo Suzuki–Miyaura CC reactions with suitable aryl halides.

**Scheme 36:**
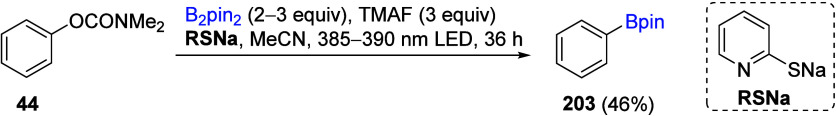
Photoinduced Thiolate-Catalytic Strategy for the *ipso*-Borylation of a Strong C–O Bond in Carbamate **44** Adapted with permission from ref (256). Copyright 2021 Springer Nature.

Wang et al.^256^ developed a photocatalytic strategy for the *ipso*-borylation of substituted arenes with a very broad range of inert C–X bonds, including C–O bonds of phenol derivatives, such as carbamate **44**, by using thiolate as a catalyst in a radical borylation reaction, thereby expanding the scope of available aryl radical precursors. Despite the high reducing power, this reaction leads to borylated products in moderate yield (Scheme 36).

### 7.8 Sulfonamidation of ArOAm

**Scheme 37:**
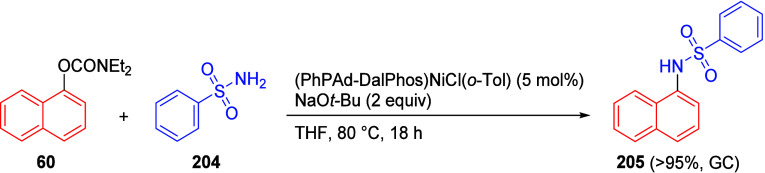
Ni-Catalyzed *N*-Arylation of a Primary Sulfonamide with Naphthyl *O*-Carbamate Adapted with permission from ref (257). Copyright 2020 John Wiley and Sons.

McGuire et al.^257^ demonstrated that naphthyl *O*-carbamate **60** could be coupled with aryl sulfonamide **204** to form *N*-(naphthalen-1-yl)benzenesulfonamide (**205**) in 95% yield (by GC) using the air-stable precatalyst (PhPAd-DalPhos)NiCl(*o*-Tol) (CAS 2290490-29-6) (Scheme 37).

Given the potential applications of benzenesulfonamides as carbonic anhydrase inhibitors,^258−260^ it would be beneficial to expand the substrate scope, e.g., by evaluating substituted naphthyl *O*-carbamates and ArOAms as substrates.

### 7.9 Amidation of ArOAm

**Scheme 38:**
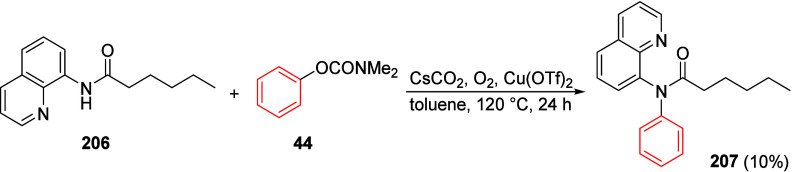
Amidation of Phenyl *O*-Carbamate **44** to Form *N*-Phenyl-*N*-Hexanamide (**207**) in 10% Yield Adapted with permission from ref (261). Copyright 2015 Royal Society of Chemistry.

There is also one isolated example of the amidation of phenyl *O*-carbamate **44** (Scheme 38). This was performed by Kathiravan et al.^261^ as part of a screening process involving the copper catalyzed amidation of aryl halides and other functionalized arenes.

### 7.10 Cyanation of ArOAm

**Scheme 39:**
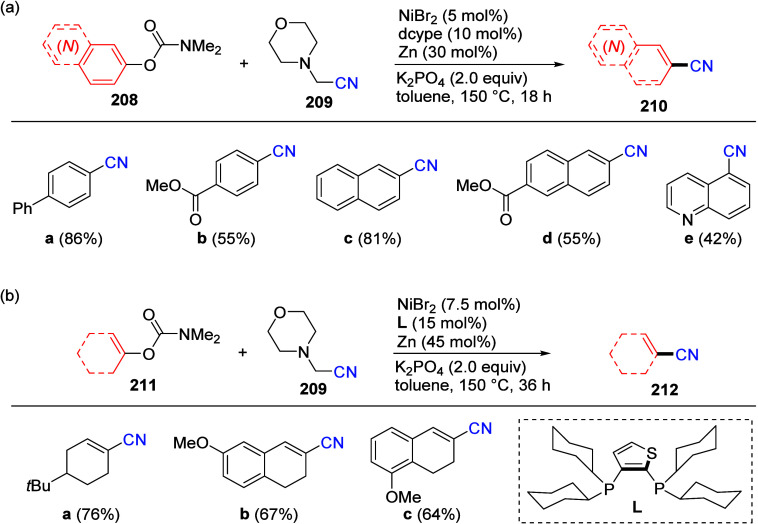
Selected Substrate Scope for Cyanation of (a) (hetero)ArOAms **208** to Form (Hetero)arylnitriles **210** and (b) Enols **211** to Give Alkenyl Nitriles **212** Adapted from ref (262). Copyright 2016 American Chemical Society.

Takise et al.^262^ developed an environmentally friendly and easy-to-use method for arylnitrile synthesis using carbamates (exemplified by **208**) and metal-free cyanating agents, aminoacetonitriles (Scheme 39a).

The reaction was catalyzed by Ni/dcype (1,2-bis(dicyclohexylphosphino)ethane) catalyst. Of the various aminoacetonitriles investigated, the morpholinoacetonitrile (**209**) gave the best yields of the products **210**. The method was also applicable to the synthesis of heteroaryl (HetOAm) **210e** and alkenyl nitriles **212** from the corresponding HetOAm **208e** and enol derivatives **211**, respectively (Scheme 39b).

## 8 Cross-Coupling (CC) Reactions Involving ArOAm

### 8.1 Introduction

The synthesis of new carbon–carbon (C–C), carbon–nitrogen (C–N), carbon–hydrogen (C–H) and carbon–silicon (C–Si) bonds usually occurs through transition metal-catalyzed CC reactions, with aryl halides generally playing the role of the electrophilic coupling partner.^263−268^ However, because of their costly synthesis and the halide waste produced, cheaper and greener alternatives have been explored such as phenols and phenolic derivatives, sometimes referred to as pseudohalides in this context.^269−272^ Thus, along with other DMGs,^135^ ArOAms have recently found utility as a C(sp^2^)–O electrophilic coupling partner^271,273−277^ due to their relative stability^270^ and their use as a directing group in metalation (section 2) and C–H activation chemistry (section 10). Indeed, combining D*o*M with CC for ArOAms is an effective strategy for synthesis of polyaromatic compounds. There are several CC strategies which complement the D*o*M reaction for ArOAms: arylation, alkylation, and alkynylation. In this section, we will discuss each coupling strategy according to the type of metal-catalyzed CC, which we have based on the coupling reagents, following a broad definition of CC set out by Reimann et al.^267^

### 8.2 Suzuki–Miyaura CC Reactions

#### 8.2.1 D*o*M Installation of Boronic Acid, Followed by Suzuki–Miyaura CC Reactions

The Nobel prize discovery of the Suzuki–Miyaura reaction^278−280^ provided opportunities for D*o*M-CC combined chemistry. Sharp and Snieckus were among the first to confirm this potential via efficient CC of aryl boronic acids derived by D*o*M with aryl, heteroaryl, and benzyl bromides to yield unsymmetrical biaryls,^281^ subsequently applied to the first steps of the large-scale synthesis of losartan (sold under the brand name Cozaar).^282^ The now-appreciated D*o*M strategy has witnessed numerous applications and extensions, including extensive application in the synthesis of bioactive molecules (section 9).

**Scheme 40:**
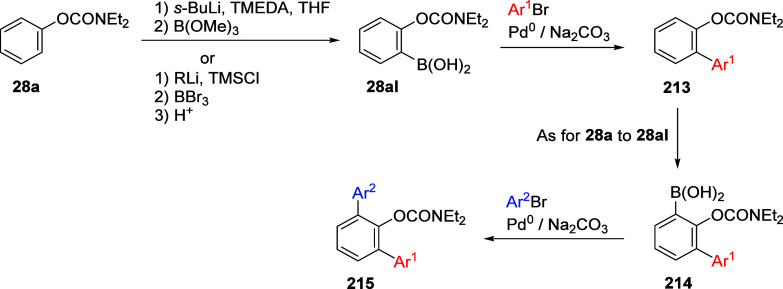
*ortho*-Boronation to Form **28al** Followed by Suzuki–Miyaura CC for the Synthesis of Triaryl Compound **215** Adapted from ref (113). Copyright 2011 American Chemical Society.

As a demonstration of the expectant value of the D*o*M-CC strategy, conversion of **28a** into *ortho*-boronic acid **28al** by direct D*o*M, quenching with trimethyl borate,^283^ or via the corresponding TMS reagent followed by *ipso*-borodesilylation,^202^ followed by the CC procedure under standard conditions (Ar^1^Br as limiting reagent/3 mmol % of Pd(PPh_3_)_4_/2 M Na_2_CO_3_(aq)/toluene/reflux/6–12 h) gives biaryl **213** (Scheme 40). Repetition of the sequence through **214** affords triaryl **215**. The incorporation of DMGs into Ar^1^ and/or Ar^2^ of **215** clearly suggests further iterative processes.

ArOAms can couple with aryl boronic acids via Suzuki–Miyaura–CC chemistry catalyzed by either nickel, rhodium, or iron catalysts. Each of these metal catalysts is discussed in more detail below.

#### 8.2.2 Nickel-Catalyzed CC Reactions of ArOAm

Consideration of bond dissociation energies suggested the expectation of using ArOAms directly in Suzuki–Miyaura CC.^113^ After considerable exploration, conditions of NiCl_2_(PCy_3_)_2_ catalysis under defined ratio of starting (ArBO)_3_ (aryl boroxine):ArB(OH)_2_ = 10:1 (4 equiv) resulted in a general synthesis of biaryls in good to excellent yields,^113^ as shown by the examples provided in Table 18.

**Table 18:**
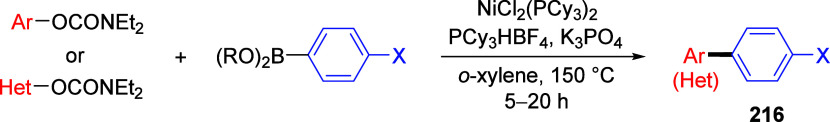

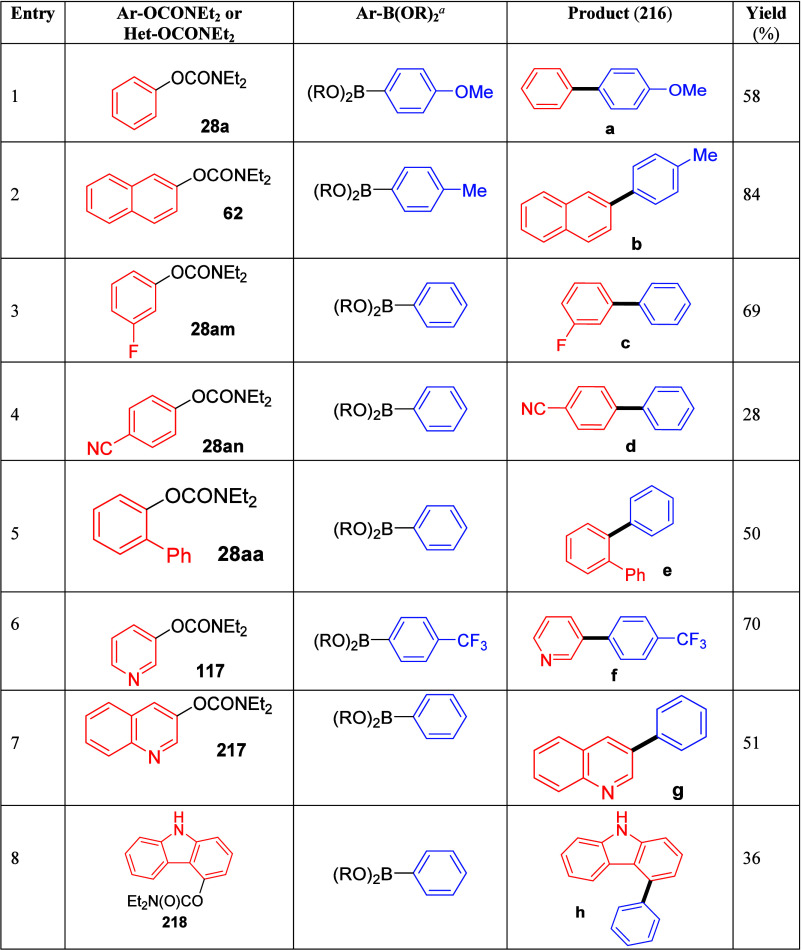
CC of Heteroaryl- and ArOAms with Select Aryl Boronates to Form Substituted Biaryl Compounds **216**^b^

a [Ar–B(OR)_2_], ratio of (ArBO)_3_:ArB(OH)_2_ = 10:1.

b Adapted from ref (113). Copyright 2011 American Chemical Society.

The discussed methodology is amenable to substrates bearing electron-donating and electron-withdrawing groups (entries 1–4), in addition to those that occupy *ortho*-substituents (entry 5) and heterocyclic structures (entries 6–7). In the case of electron-withdrawing groups, unlike the *meta*-F derivative **28am** (entry 3), which gave a high yield (69%), the *p*-cyano derivative **28an** (entry 4) gave an anomalous result (low yield of **216d**) because of CC at the cyano group, resulting in a mixture of products. Thus, the ability of other functional groups to participate in CC needs to be taken into consideration when designing D*o*M-CC procedures.

**Scheme 41:**
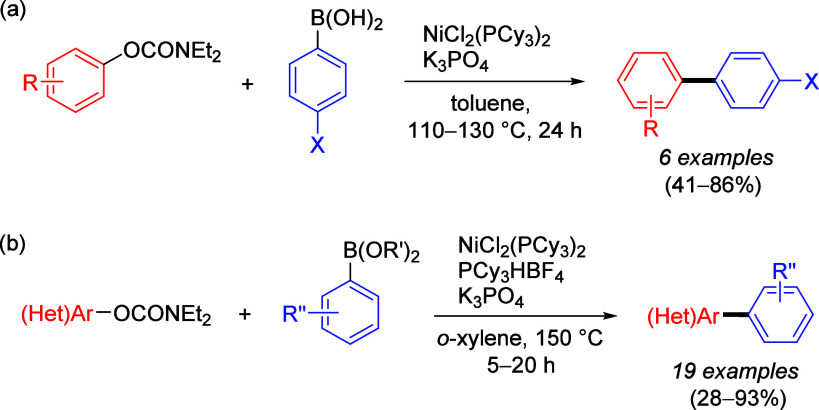
Ni-Catalyzed CC of ArOAms as Reported by (a) Quasdorf et al.^284^ and (b) Antoft-Finch et al.^20^ Separately but Simultaneously in 2009 (a) Adapted from ref (284). Copyright 2011 American Chemical Society. (b) Adapted from ref (20). Copyright American Chemical Society.

The first CC of ArOAms with aryl boronic acids was simultaneously published in 2009 by Quasdorf et al.^284^ and Antoft-Finch et al.^20^ Both groups employed the same stable commercially available and air/moisture stable catalyst [NiCl_2_(PCy_3_)_2_] and similar conditions (base, heating) except that Antoft-Finch et al. also used an air-stable ligand, tricyclohexylphosphine tetrafluoroborate (PCy_3_HBF_4_) for the CC and included several reactions with heteroArOAm substrates (Scheme 41). Quasdorf et al.^284^ also used aryl boronic acid ArB(OH)_2_ which reversibly forms aryl boroxine (ArBO)_3_ at elevated temperatures (Scheme 41a), while Antoft-Finch et al. employed a 10:1 mixture of (ArBO)_3_ and ArB(OH)_2_, commonly referred to as ArB(OR)_2_ (Scheme 41b).^20^

**Scheme 42:**
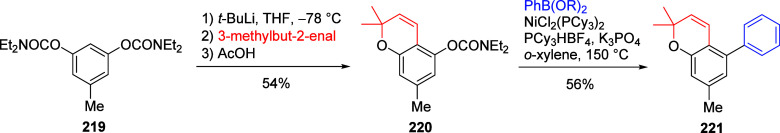
Synthesis of 5-Phenyl-2*H*-chromene **221** Beginning from Bis(carbamate) Precursor **219** ([PhB(OR)_2_], Ratio of (PhBO)_3_:PhB(OH)_2_ = 10:1 Adapted from ref (113). Copyright 2011 American Chemical Society.

A few years later, in a collaborative effort, the two groups joined together and extended this work^113^ to improve the yields for some of the compounds already appearing in their separate 2009 work and to demonstrate CC of new *ortho*-substituted ArOAms. This included the synthesis of polysubstituted aromatic compounds (PAHs) as exemplified by the synthesis of 5-phenyl-2*H*-chromene **221** (Scheme 42), a heterocyclic scaffold of interest for bioactive compounds^285^ and natural products.^286^ The synthesis of **221** commences from bis(carbamate) precursor **219**: sequential treatment with *t*-BuLi, 3-methylbut-2-enal, and AcOH in one-pot enables the conversion of compound **219** into 2*H*-chromene carbamate **220**. Suzuki–Miyaura CC of **220** with PhB(OR)_2_ provided biaryl **221** in 56% yield. More examples of the use of D*o*M-CC sequence to form bioactive compounds and PAHs can be found in section 9.

**Scheme 43:**
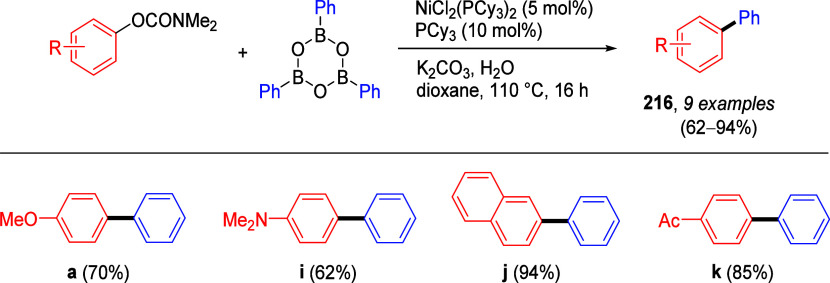
Suzuki–Miyaura CC of ArOAms with Aryl Boroxines Using [NiCl_2_(PCy_3_)_2_] as a Precatalyst Adapted from ref (253). Copyright 2010 American Chemical Society.

Shortly after the initial 2009 reports, Xu et al.^253^ developed the Suzuki–Miyaura reaction of ArOAms with a mixture of aryl boroxine (ArBO)_3_ and 0.88 equiv of H_2_O, using [NiCl_2_(PCy_3_)_2_] as a precatalyst (selected substrates shown in Scheme 43). Using aryl boroxines results in an improvement in the coupling efficiency for electron rich ArOAms. These are typically challenging substrates for this type of chemistry due to deactivation of the ArOAms by the electron-donating substituents.

Thus, coupled products (e.g., **216a**,**i**) were produced, albeit in slightly lower yields than for naphthyl and phenyl carbamates with electron-withdrawing groups (e.g., in forming **216j**,**k**). The use of aryl boroxines also offered lower catalyst loading (1 mol %) for alkenyl *O*-carbamates and easy scale-up.

Baghbanzadeh et al.^287^ demonstrated that microwave (MW) heating enabled a shorter reaction time and reduced amount of phosphine ligand for both fused and nonfused aromatics (Table 19). Remarkably, the yields of challenging substrates (those with electron-donating groups) were significantly higher when prepared using MW heating compared with conventional heating (e.g., entry 2).

**Table 19:**
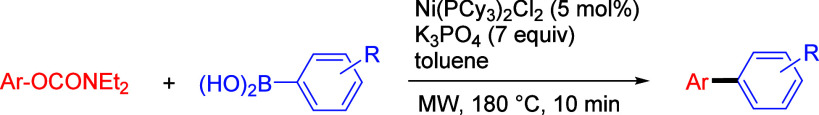

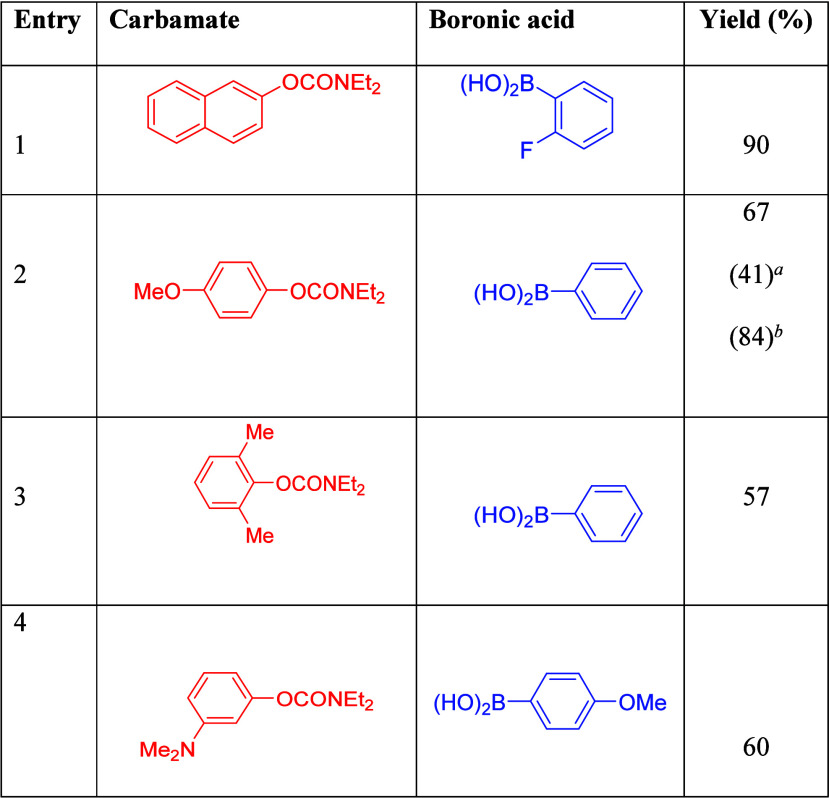
ArOAms in Microwave-Assisted Suzuki–Miyaura CC Reactions^c^

a NiCl_2_(PCy_3_)_2_ (5 mol %), ArB(OH)_2_ (2.5 equiv), K_3_PO_4_ (4.5 equiv), toluene (0.3 M), 110 °C, 24 h.^284^

b [Ni(Triaz^Nme2^-*I*-Pr)Cl] (2 mol %), ArB(OH)_2_ (1.5 equiv), K_3_PO_4_ (2 equiv), toluene (0.025 M), 135 °C, 16 h.^288^

c Adapted from ref (287). Copyright 2011 American Chemical Society.

**Scheme 44:**
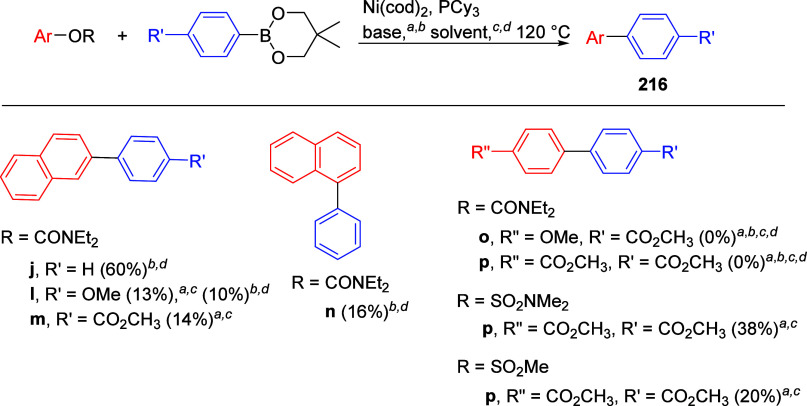
CC of ArOAms with *para*-Substituted Aryl Neopentylglycolboronates Adapted from ref (284). Copyright 2012 American Chemical Society. *^a^* 4.5 equiv of CsF, *^b^* 3 equiv of K_3_PO_4_, *^c^* toluene, and *^d^* THF.

Shortly after this, Leowanawat et al.^289^ explored CC of ArOAms in Ni-catalyzed CC with phenyl neopentylglycolboronates containing electron-rich and electron deficient substituents in the *para*-position (Scheme 44). Generally, low yields were reported for the coupling products (**216l**–**p**). In some cases, 0% yield was recorded, although this could be improved using sulfamate and sulfonate directing groups to give **216p**.

**Scheme 45:**
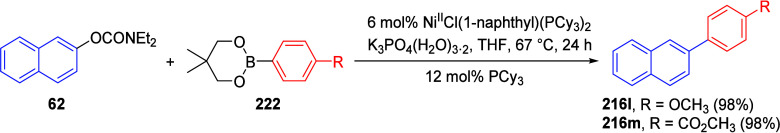
CC of Naphthyl OAm with Aryl Neopentylglycolboronates Catalyzed by Ni^II^Cl(1-naphthyl)(PCy_3_)_2_/PCy_3_ Adapted with permission from ref (290). Copyright 2016 Thieme.

Malineni et al.^290^ went further to develop an air stable and yet reactive Ni(II) precatalyst (more reactive than currently employed Ni(II)phosphine complexes and π-Ni(0) precatalysts) for the CC of naphthyl *O*-carbamate (**62**) with aryl neopentylglycolboronates **222** containing *para*-electron-rich and electron deficient substituents in quantitative yields (Scheme 45).

Ohtsuki et al.^291^ were the first to demonstrate Ni/NHC-catalyzed CC of ArOAms with arylboron reagents (Table 20), with the optimal catalyst/ligand combination consisting of bis(cyclooctadiene) nickel(0) [Ni(COD)_2_] and NHC ligands bearing 2-adamantyl groups (generated the most active nickel species among the ligands examined).

**Table 20:**
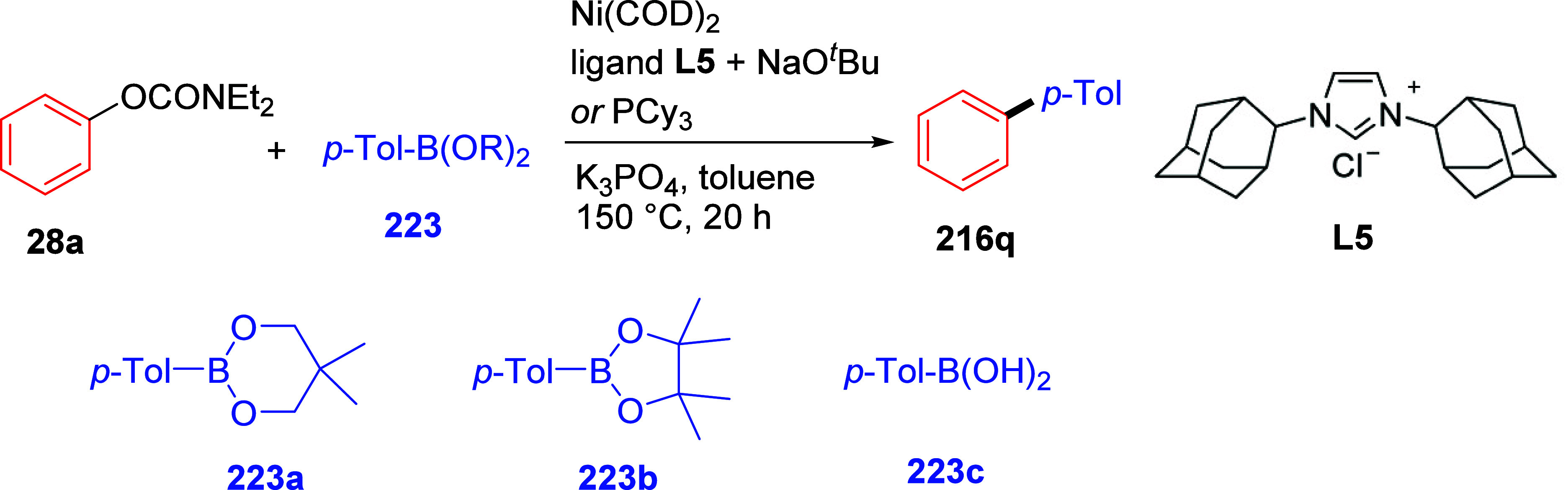
Nickel/NHC-Catalyzed CC of ArOAm **28a** with *p*-Tolylboronic Esters **223**^a^

entry	boron reagent (**223**)	ligand	yield of **216q** (%)
1	a	**L5**	70
2	a	PCy_3_	trace
3	b	**L5**	39 (61)^b^
4	b	PCy_3_	trace
5	c	**L5**	81
6	c	PCy_3_	75

a Adapted from ref (291). Copyright 2016 American Chemical Society.

b 160 °C.

An advantage of using an NHC over the PCy_3_ ligand in the nickel-catalyzed Suzuki–Miyaura reaction is the anhydrous nature of the reaction, and therefore its tolerance of aryl boronic esters (essentially boronic acid PGs), enabling reliable understanding of reaction stoichiometry (aryl boronic esters have known molecular masses), and facilitating reactions when the analogous boronic acids are unstable,^292^ or for boron compounds prepared by C–H borylation (typically boronic esters).^293^

In another study involving a variation of the nickel catalyst, Mastalir et al.^288^ focused on 4-methoxyphenyl diethylcarbamate (**28ak**), demonstrating that it could be coupled with phenylboronic acid using an air-stable triazine-based Ni(II) PNP pincer complex using K_3_PO_4_ as base. The yield for this reaction was 84%, higher than what was achieved using alternative conditions (67%,;^287^ 41%,^284^) (Table 19, entry 2). Hence, it is unfortunate that no other ArOAms were tested in their study.

**Scheme 46:**
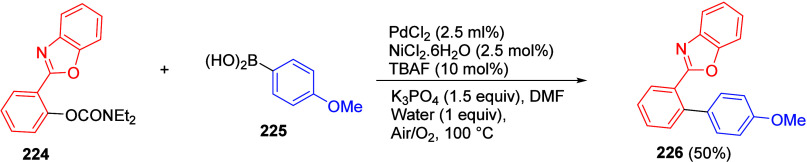
Ni–Pd Binary NP-Catalyzed Suzuki–Miyaura Cross-Coupling of *ortho*-Heteroaryl-Tethered Aryl *O*-Carbamate **224** with Boronic Acid **225** to Form Cross-Coupled Product **226** Adapted from ref (294). Copyright 2017 American Chemical Society.

Yet another variation of the nickel catalyst was reported by Purohit et al.:^294^ nickel–palladium heterobimetallic nanoparticles (NPs) were developed for activation of C–O bonds in *ortho*-heteroaryl-tethered aryl carbonates for Suzuki–Miyaura cross-coupling with aryl boronic acids. However, these NP catalysts (generated *in situ* from NiCl_2_·6H_2_O and PdCl_2_) were also found to catalyze the coupling of an aryl *O*-carbamate (**224**) with boronic acid **225** to form **226** in a modest 50% yield (Scheme 46).

**Scheme 47:**
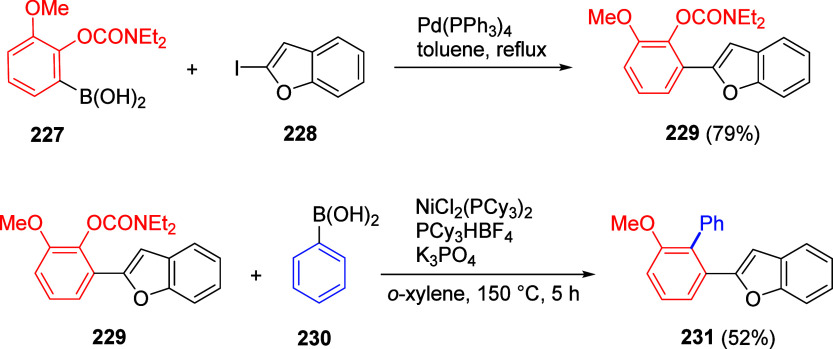
Synthetic D*o*M-CC Interconnections of ArOAms **227** and **229**: (a) Pd-Catalyzed Coupling of **227** and **228** to Form **229**; (b) **229** Is Coupled with Phenylboronic Acid (**230**) Using a Ni-Catalyst to Form **231** Adapted from ref (295). Copyright 2009 American Chemical Society.

As already mentioned, the *O*-carbamate functionality is inert to certain catalysts, e.g., Pd(PPh_3_)_4_. This allows ArOAm to assume an orthogonal CC partner role when linked with the Ni coupling strategy.^20,295,296^ For example, the synthesis of **229** (Scheme 47) involves the Suzuki–Miyaura CC of **227** and 2-iodobenzofuran **228**;^295^**229** can then be coupled with phenylboronic acid (**230**) using a Ni catalyst to form compound **231** in 52% yield.

**Scheme 48:**
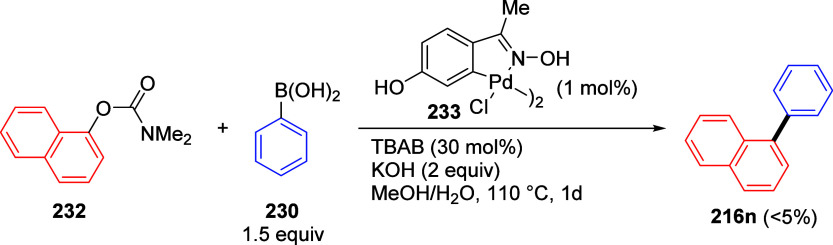
An Attempt to Use Oxime Palladacycle **233** for the Suzuki–Miyaura Arylation of Naphthalen-2-yl Dimethylcarbamate **232** Adapted with permission ref (297). Copyright 2012 John Wiley and Sons. TBAB = tetra-*n*-butylammonium bromide.

The inertness of *O*-carbamate functionality toward Pd-catalysts is also evident in the work of Cívicos et al.,^297,298^ who attempted to use an oxime palladacycle **233** as a catalyst for the CC of naphthalen-2-yl dimethylcarbamate **232** and phenylboronic acid **230** (Scheme 48). A disappointing <5% yield for the desired 2-phenylnaphthalene (**216n**) was reported.

**Scheme 49:**
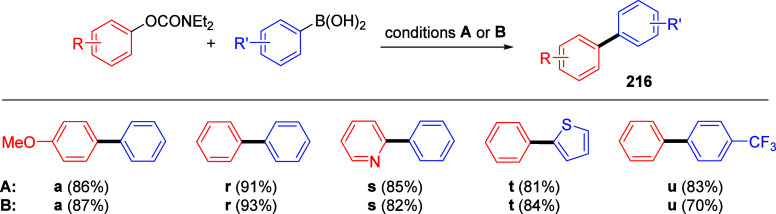
Suzuki–Miyaura CC of ArOAms Catalyzed by Ni(II) (Condition A) or Ni(0) (Condition B) Nanoparticles Immobilized on EDTA-Modified Fe_3_O_4_@SiO_2_ Adapted from ref (299). Copyright 2020 American Chemical Society.

Inaloo et al.^299^ developed recoverable and reusable magnetic catalysts to create a more sustainable approach to C–C bond formation. The catalysts consisted of nickel(II) nanoparticles immobilized on EDTA-modified iron catalysts for oxide coated with silica (Fe_3_O_4_@SiO_2_) nanospheres and were explored as recyclable ligand-free Suzuki–Miyaura coupling of ArOAms with phenylboronic acids. The nanoparticle catalysts were used to catalyze CC reactions between various ArOAms and phenylboronic acid substrates. As shown by a few selected examples in Scheme 49 (conditions A), the process enabled CC in good to excellent yields of product **216**. The yield of the reaction decreased negligibly over six cycles (catalyst recovered and regenerated before each fresh run), indicating the recyclability of these NPs.

The same authors modified their procedure to incorporate Ni(0) catalysts, i.e., nickel(0) nanoparticles immobilized on EDTA-modified Fe_3_O_4_@SiO_2_ nanospheres [Fe_3_O_4_@SiO_2_–EDTA–Ni(0)] as these were believed to lead to higher yields of coupled products compared with Ni(II).^300^Scheme 49 (conditions B) shows some examples of CC reactions performed with these recyclable catalysts. A close analysis of these results (and the other published results) reveals that there is not a significant difference between using Ni(II) versus Ni(0).

In a similar study intended to make the Suzuki–Miyaura process more sustainable, Ramgren et al.^301^ successfully performed Ni-catalyzed CC of ArOAms with aryl boronic acids in green solvents (2-methyltetrahydrofuran; *tert*-amyl alcohol). Five substrates including fused and nonfused ArOAms as well as a carbazole and quinoline *O*-carbamate were coupled with phenylboronic acid, providing coupled products in good to excellent yields (63–100%).

**Scheme 50:**
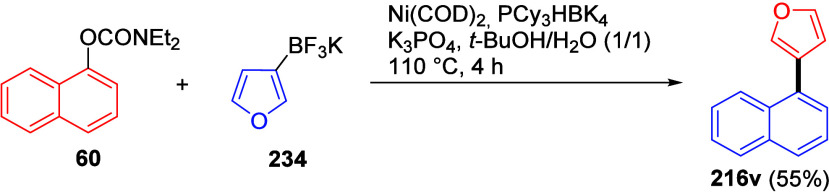
Reaction of 1-Naphthyl *O*-Carbamate (**60**) with Potassium Furan-3-yltrifluoroborate **234** Adapted from ref (302). Copyright 2010 American Chemical Society.

Molander and Beaumard^302^ developed a general method for the Ni-catalyzed C–O activation of various phenol derivatives (including that of 1-naphthyl *O*-carbamate) with potassium (hetero)aryltrifluoroborates. When evaluated alongside other phenol derivatives in a reaction with potassium furan-3-yltrifluoroborate (**234**), 1-naphthyl *O*-carbamate **60** afforded the product **216v** less efficiently in a 55% yield (Scheme 50), whereas the *O*-mesylate, tosylate, sulfamate, and pivaloyl provided the corresponding products in quantitative, 85%, 85%, and 68% yields, respectively.

**Scheme 51:**
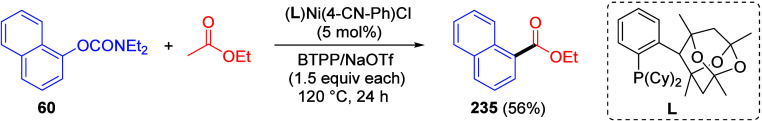
Reaction of 1-Naphthyl *O*-carbamate **60** with Ethyl Acetate to Form **235** Adapted with permission from ref (303). Copyright 2022 John Wiley and Sons.

There is also one isolated example of the Ni-catalyzed reaction of 1-naphthyl *O*-carbamate **60** with ethyl acetate as a feedstock solvent, reported by MacMillan et al.^303^ (Scheme 51).

#### 8.2.3 Rhodium-Catalyzed CC Reactions of ArOAm

**Scheme 52:**
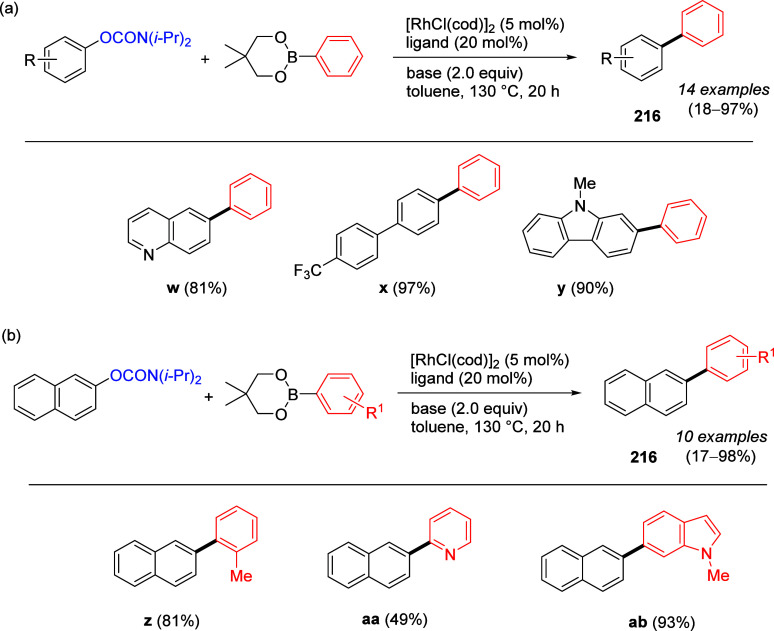
Examples of Products Prepared via Suzuki–Miyaura Reaction of (a) Phenyl *O*-Carbamates and (b) Naphthyl *O*-Carbamates with Boronic Esters (Ester Group = Neopentylglycolate), Using a Rhodium Catalyst Adapted with permission from ref (304). Copyright 2015 Elsevier.

The research groups of Tobisu and Chatani (Osaka University)^304^ collaboratively developed rhodium-catalyzed CC of phenyl (Scheme 52a) and naphthyl *O*-carbamates (Scheme 52b) with organoboron reagents using an NHC ligand to form coupled products **216**.

This reaction involves the rhodium-mediated activation of the relatively inert C(aryl)–O bond of the aryl carbamates. Similar to the work carried out by Mastalir et al.,^288^ the use of an NHC ligand bearing a 2-adamantyl group was essential to the success of the reaction.

#### 8.2.4 Iron-Catalyzed CC Reactions of ArOAm

**Scheme 53:**
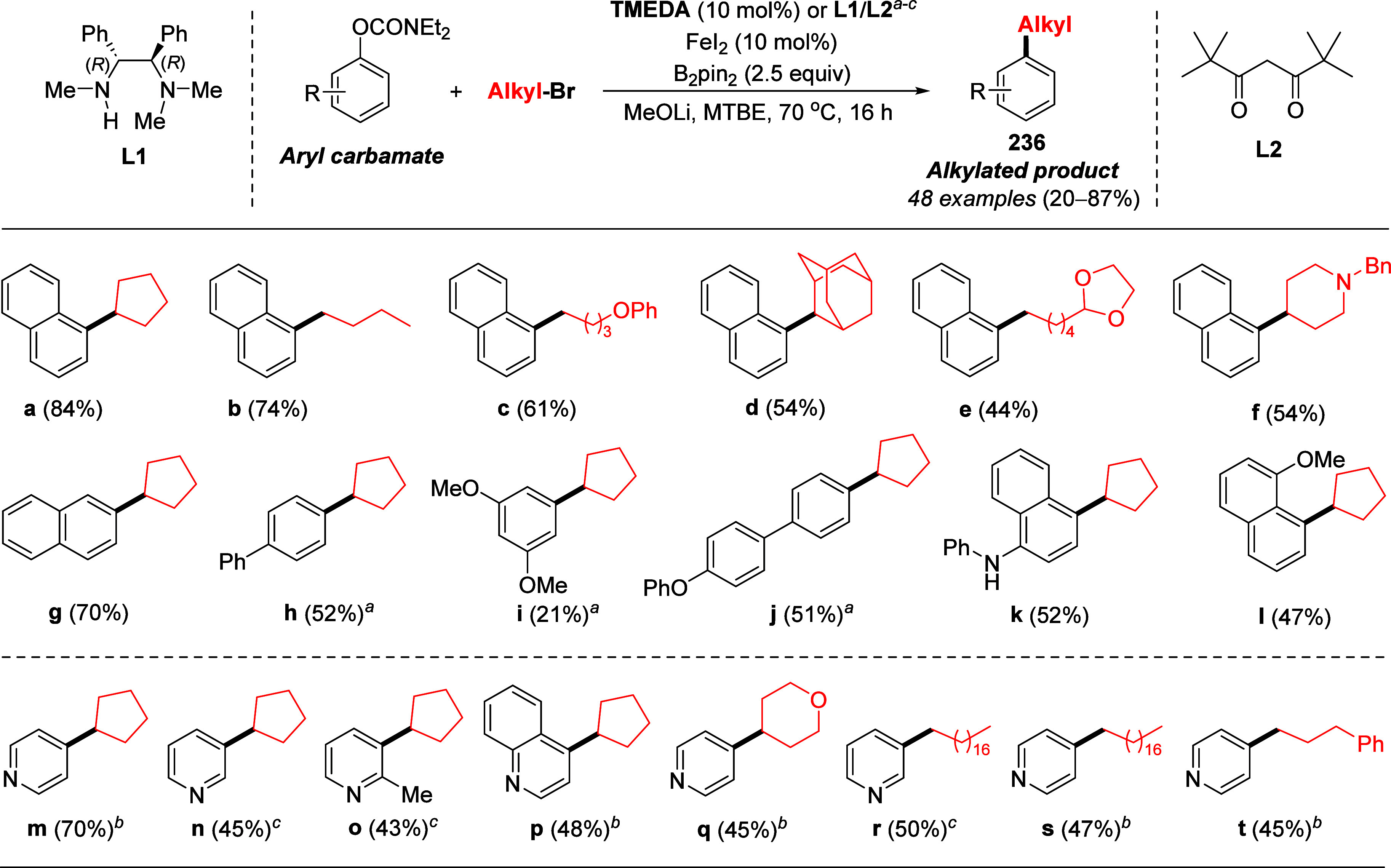
Selected Examples of Iron-Catalyzed CC Reaction of Aryl and HeteroArOAms with Alkyl Bromides into Alkylated Arenes and Pyridines **236*^d^*** **L1** (0.1 equiv), FeCl_2_ (0.1 equiv). **L2** (0.1 equiv), FeBr_2_ (0.1 equiv). **L1** (0.1 equiv), FeBr_2_ (0.1 equiv). This figure has been published in *CCS Chemistry* in 2023, ref (305).

An example of Fe-catalyzed CC reaction of aryl and heteroArOAms with alkyl bromides via inert C–O bond activation was reported by Chen et al.^305^ (Scheme 53). This reaction proceeds under mild conditions with good functional compatibility and yields the alkylated arenes and pyridines **236** with good efficiency. This protocol enables the late functionalization of bioactive compounds, the direct alkylation of phenols and the simple synthesis of multisubstituted arenes.

**Scheme 54:**
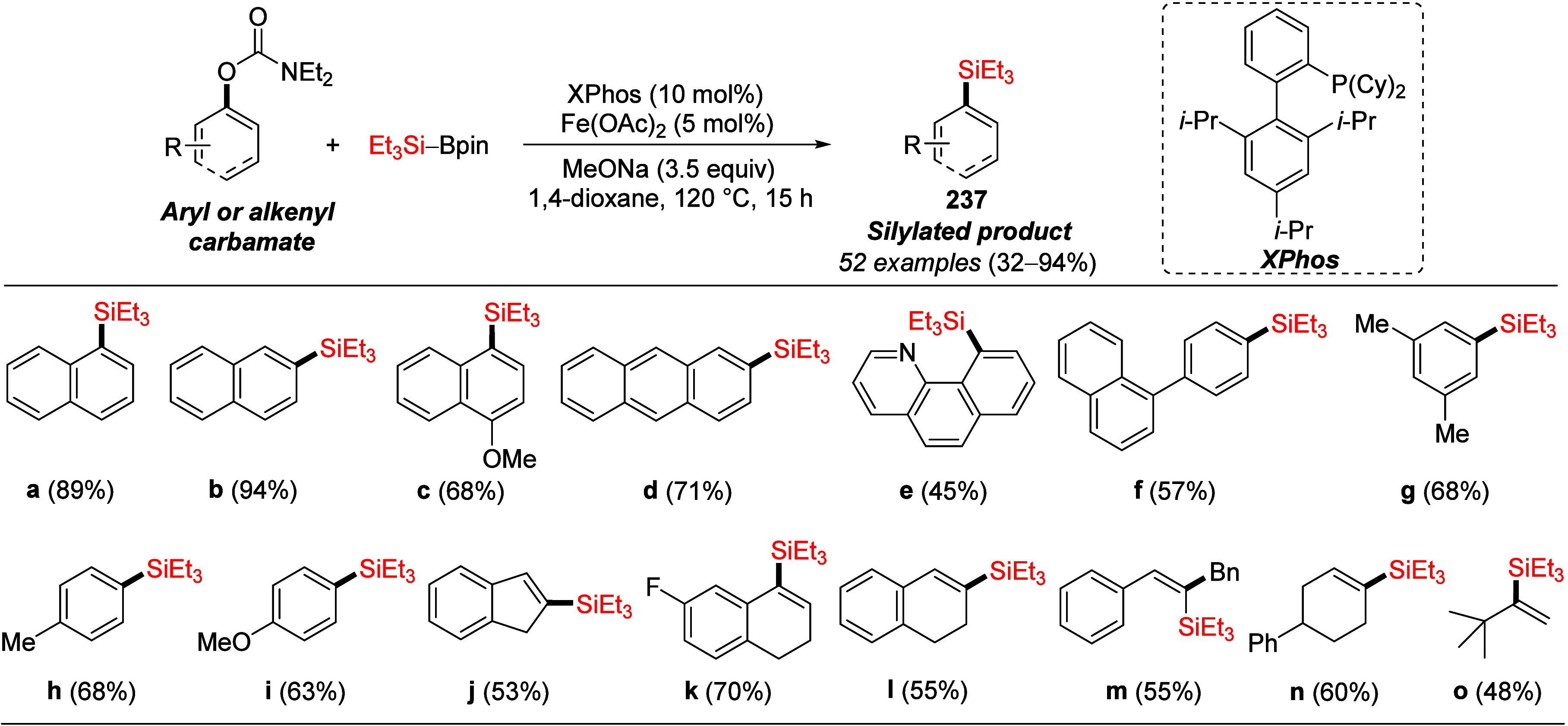
Examples of Iron-Catalyzed Silylation of Aryl and Alkenyl *O*-Carbamates into Silylated Products **237** Adapted from ref (306). Copyright 2020 American Chemical Society.

Another example of Fe-catalyzed coupling reaction is a simple silylation of aryl and alkenyl *O*-carbamates to **237** by activation of the C–O bond (Scheme 54), developed by Zhang et al.^306^ The reaction is highly efficient with a broad substrate range and high yield and could be used for late silylation of bioactive compounds, offering potential applications in drug discovery and development.

### 8.3 Kumada–Corriu CC Reactions

#### 8.3.1 Nickel-Catalyzed Kumada–Corriu CC Reactions

**Scheme 55:**
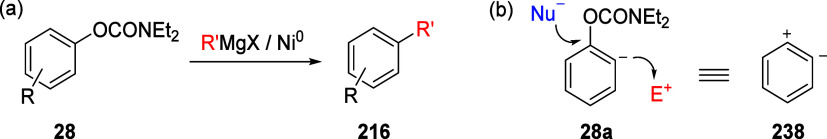
(a) General Ni(0)-Catalyzed CC Reactions of ArOAms with Grignard Reagents (to Form Biaryl **216**); (b) in the Combined D*o*M-Nucleophilic *ipso*-Substitution, the ArOAm Substrate Can Be Viewed as an Aromatic 1,2-Dipole Equivalent (**238**) Adapted from ref (229). Copyright 1992 American Chemical Society.

To test the possibility of the equally respected Kumada–Corriu CC reaction, Sengupta et al.^229^ applied Ni(0)-catalysis, demonstrated the first general application of ArOAms in CC with Grignard reagents to give substituted biaryls **216** (Scheme 55a). This discovery established a distinctive *ortho*-functionalization through combined DoM-nucleophilic *ipso-*substitution (**28a**), introducing a concept of 1,2-dipole equivalency **238** (Scheme 55b), different to what is observed for aryl *O*-triflates.^229^

**Scheme 56:**
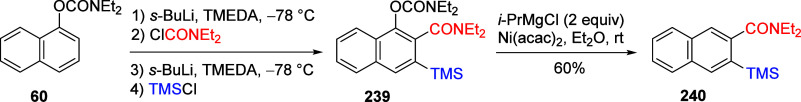
D*o*M-Mediated Consecutive Introduction of Carbamoyl and Silyl Electrophiles to Naphthyl *O*-Carbamate **60**, Leading to **239**, Which, upon Treatment with *i*-PrMgCl/Ni(acac)_2_, Gives **240** Adapted from ref (229). Copyright 1992 American Chemical Society.

In contrast to methyl-, TMSCH_2_-, and aryl-Grignards, affording the corresponding products **216** (R′ = Me, TMSCH_2_, and Ar, respectively), *n*-BuMgCl, and *i*-PrMgCl, caused reductive removal of the *O*-carbamate group (introduced in section 7.1.2.). The authors demonstrated the utility of the concept shown in Scheme 55b by consecutive D*o*M introduction of carbamoyl and silyl electrophiles on to **60** to form **239** (Scheme 56), which, upon treatment with *i*-PrMgCl/Ni(acac)_2_, afforded **240**, a compound offering further D*o*M chemistry options.

**Scheme 57:**
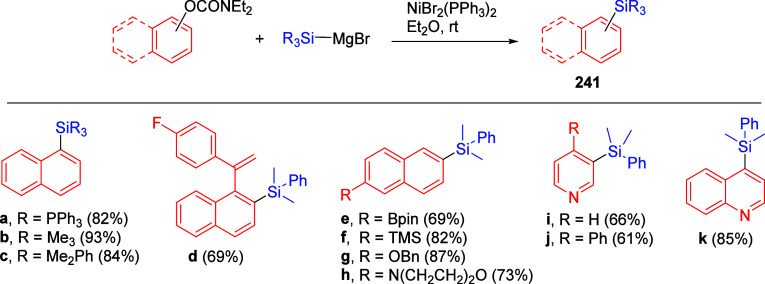
Examples Showing Scope of Aryl and Naphthyl *O*-Carbamate Substrates Undergoing Silylation Adapted with permission from ref (307). Copyright 2019 Elsevier.

Similar to the work done by Sengupta et al. (Scheme 55), Murugesan et al.^307^ achieved nickel-mediated CC of naphthyl and heteroArOAms with silylmagnesium reagents under ambient conditions for a wide range of ArOAms (Scheme 57). For instance, 1-naphthyl diethylcarbamate gave the silanes **241a**–**c** in 82–93% isolated yield depending on the bulkiness of the silylmagnesium reagent. It was interesting to note the compatibility of certain functional groups with this chemistry, notably fluoride (**241d**), Bpin (**241e**), TMS (**241f**), ethers (**241g**), and morpholine (**241h**). Three heterocycles were also shown to tolerate these reaction conditions namely pyrazole (not shown), pyridine (**241i**,**j**) and quinoline (**241k**).

**Scheme 58:**
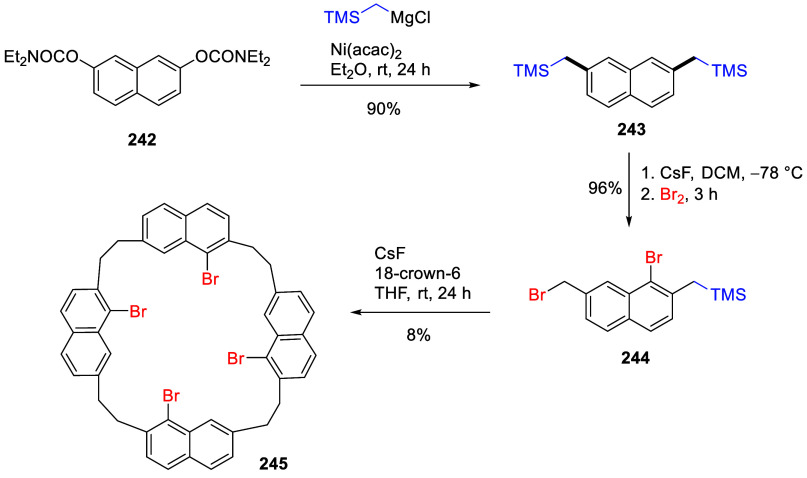
Synthesis of [2.2.2.2](2,7)-1-Bromonaphthalenophane **245** Commencing with Naphthalene-2,7-diyl Bis(diethylcarbamate) **242** Adapted with permission from ref (308). Copyright 2019 John Wiley and Sons.

There is also one report of methyltrimethylsilylation of naphthalene-2,7-diyl bis(diethylcarbamate) **242** (Scheme 58).^308^ This was carried out using [(trimethylsilyl)methyl]magnesium chloride in the presence of Ni(acac)_2_ to form **243**, which was subsequently transformed into the unsymmetrical dibromide **244**, found to undergo cyclo-oligocondensation to form tetrameric [2.2.2.2]naphthalenophane **245**, potentially useful as a chiral host molecule (through modification of the bromine atoms by larger groups, suppressing ring inversion) or as a precursor to curved aromatic compounds via intramolecular C–C coupling reactions. Interestingly, there are no reports of CC between nonfused ArOAms and silylmagnesium reagents in the literature. The poor reactivity of ArOAms (and nonfused aryl compounds in general) can be attributed to a dearomatization process (based on possible coordination from arene π-bond to metal via η^2^-fashion) that occurs during the oxidative addition step. This is not observed in fused π-systems due to the inherent stabilization they provide.^254,309−312^

Other examples showcasing nickel-catalyzed Kumada–Corriu CC reactions with aryl *O*-carbamates are shown in Table 21. Coupling of *m*-oxygen (entry 1) and *o*-oxygen (entry 2) EDGs with Grignard reagents (RMgX) occurs in reasonable yields, while comparison of triflate with *O*-carbamate shows reduced yield for the more highly hindered carbamates (entries 3a and 3b). The scope of this chemistry is illustrated by the functionalization of steroids (entry 4), although it has also been demonstrated with phenethylamines, naphthyls, phenanthryls, binaphthyls, pyridines (entry 5), quinolines, and uracils.^229^

**Table 21:**
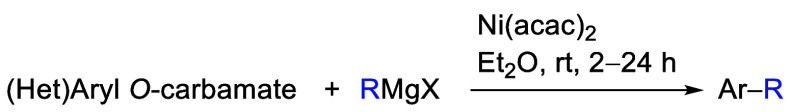

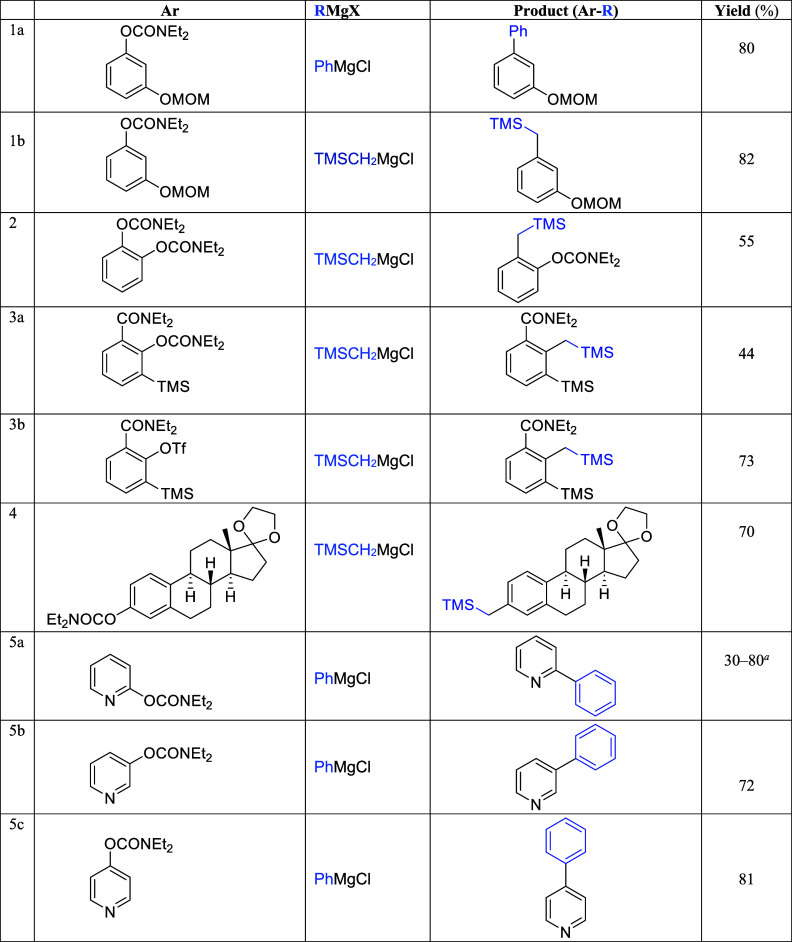
Ni(0)-Catalyzed CC of Aryl and HeteroArOAms with Grignard Reagents (RMgX)^b^

a Variable yield presumably due to possible Grignard-induced decarbamoylation.

b Adapted from ref (229). Copyright 1992 American Chemical Society.

**Scheme 59:**
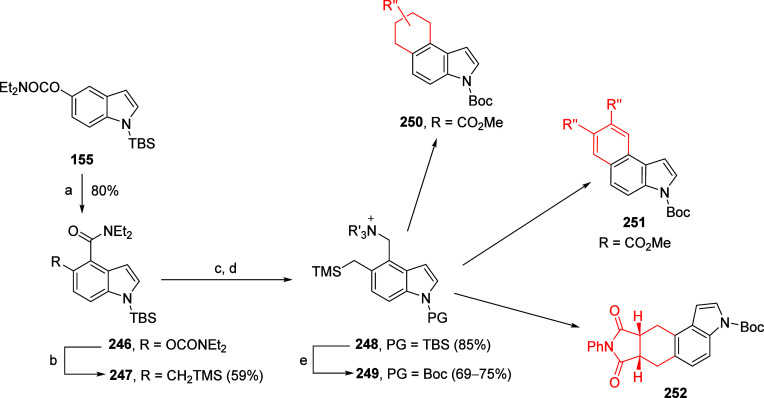
Synthesis of 4,5-Fused Indoles (**250** and **251**) and New Ring System **252** by Combined D*o*M/Nickel-Catalyzed CC Followed by Cycloaddition with Various Dienophiles Reaction conditions: (a) (i) s-BuLi, TMEDA, THF, −78 °C; (ii) Et_2_NCOCl; (b) TMSCH_2_MgCI, Ni(acac)_2_, THF, 50 °C, 4 h; (c) (i) DIBAL, THF, 0 °C to rt, (ii) Rochelle salt; (d) MeI, MeCN, rt, 12 h; (e) (Boc)_2_O, CsF, MeCN, rt, 12 h. *^a^* Adapted with permission from ref (313). Copyright 1999 Elsevier.

Kinsman and Snieckus^313^ applied this chemistry to the preparation of indole quaternary salts (**248**), useful for the synthesis of 4,5-fused indoles (**250** or **251**) via trapping with a variety of dienophiles such as methyl acrylate (to form **250**) or dimethyl acetylene dicarboxylate (to form benz[*e*]indole, **251**) (Scheme 59). Synthesis of **250** or **251** was achieved by the initial preparation of the precursor **246** by D*o*M of indole *O*-carbamate **155** (electrophile = Et_2_NCOCl), followed by nickel-catalyzed Grignard-*O*-carbamate CC to form benzyl silane **247**. Compound **247** was then converted to the quaternary salt **248** via DIBAL reduction followed by treatment with MeI. Compound **248** could then undergo cycloaddition with several dienophiles (in a large excess) in the presence of CsF or TBAF at ambient temperature to form the desired cycloadducts (**250** or **251**) in good to excellent yields (69–95%), except for cycloadduct **252** (trapping with *N*-phenylmaleimide and diisopropyl azodicarboxylate), formed in 40% yield.^313^

The product heteroaryl/naphthyl silanes shown in Schemes 55–58 and Table 21 have further utility because the silane groups can now be replaced by halogens,^307^ protodesilylated,^314,315^ borylated,^316^ carboxylated,^317^ or used as a site for further CC.^318−322^

In a separate study published by Yoshikai et al.^323^ involving similar chemistry to that shown in Scheme 58, when *p*-cresol derivatives were coupled with phenylmagnesium bromide, a considerable amount of the homocoupling product (**253**) was formed (Table 22, entry 1). In the presence of hydroxyphosphine ligands (PO ligands), however, the desired product (**216**) formed in 95% yield (entry 2). This could be replicated with a diversity of ArOAm substrates (entries 3–6).

**Table 22:**
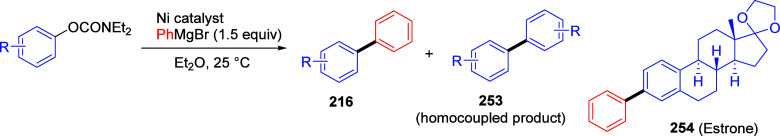
Ni-Catalyzed CC Reaction of ArOAms with Phenylmagnesium Bromide^a^

				yield (%)	
entry	R	Ni catalyst^*b*^	reaction time (h)	**216**	**253**
1	4-Me	Ni(acac)_2_ (5 mol %)	2.5	64	27
2	4-Me	Ni(acac)_2_/PO (3 mol %)	1	95	4
3	4-OMe	Ni(acac)_2_/PO (3 mol %)	1	90	
4	2-OMe	Ni(acac)_2_/PO (3 mol %)	18	87	
5	3-NH_2_	Ni(acac)_2_/PO (3 mol %)	1	96	
6	estrone	Ni(acac)_2_/PO (5 mol %)	6	89 (**254**)	

a Adapted from ref (323). Copyright 2009 American Chemical Society.

b PO = hydroxyphosphine ligands.

The PO ligands accelerate the Ni(0)-catalyzed CC reactions of ArOAms (and other unreactive electrophiles) with Grignard reagents via a nickel phosphine/magnesium alkoxide bimetallic species that activates the C–O bond in the aryl-*O*-carbamate functionality.

**Scheme 60:**
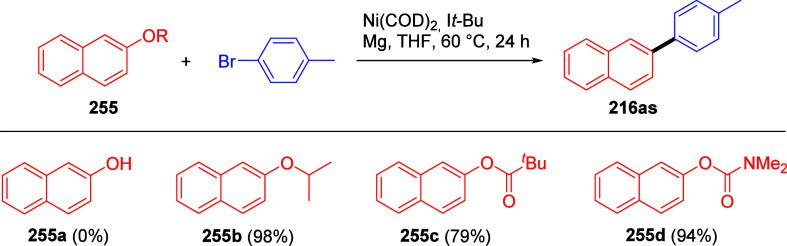
Yields of Select Examples (Product **216as**) from the CC of Naphthol (**255a**) and Protected Naphthol (**255b**–**d**) with 1-Bromo-4-methylbenzene (I*t*-Bu = 1,3-di-*tert*-butylimidazol-2-ylidene) Adapted from ref (324). Copyright 2016 American Chemical Society.

Only one example of a CC reaction involving an ArOAm (in this case, naphthyl *O*-carbamate) with an aryl halide has been reported in the literature.^324^ In this case, the authors reported Ni-catalyzed CC of various naphthol-based electrophiles with aryl bromides in the presence of magnesium in the synthesis of 2-(*p*-tolyl) naphthalene **216as** (Scheme 60), i.e., a nickel-catalyzed Kumada–Corriu CC. Under the given conditions, naphthol (**255a**) was unsuitable as a substrate unless protected as an isopropyl ether (**255b**), pivalate ester (**255c**), or *O*-carbamate (**255d**). No further reactions were carried out using the naphthyl *O*-carbamate (**255d**). To the best of our knowledge, CC between aryl bromides and *nonfused* ArOAms has not yet been reported but would be a suitably valuable chemo-transformation.

#### 8.3.2 Iron-Catalyzed Kumada–Corriu Alkylations and Arylations of ArOAm

**Scheme 61:**
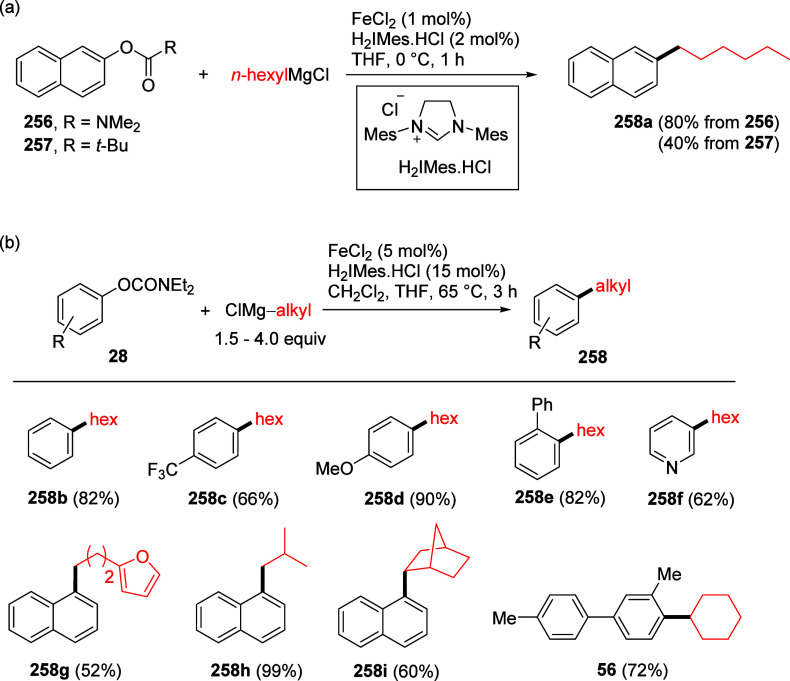
(a) Fe(II)-Catalyzed CC of Naphthalen-2-yl Dimethylcarbamate (**256**) with *n*-HexylMgCl (Adapted from ref (325), Copyright 2009 American Chemical Society); (b) Expansion of the Scope of Substrates for the Fe(II)-Catalyzed CC (Adapted from ref (174), Copyright 2008 American Chemical Society)

Li et al.^325^ demonstrated Fe(II)-catalyzed CC of naphthalen-2-yl dimethylcarbamate (**256**) with *n*-hexylMgCl to form 2-hexylnaphthalene (**258a**) in 80% yield (compared with naphthalen-2-yl pivalate (**257**), which only formed 2-hexylnaphthalene in 40% yield) (Scheme 61a). This chemistry was further explored with more substrates (ArOAms and alkyl-based Grignard reagents) by Silberstein et al.,^174^ obtaining a range of compounds (**56**, **258b**–**i**) in good to excellent yields (Scheme 61b).

**Scheme 62:**
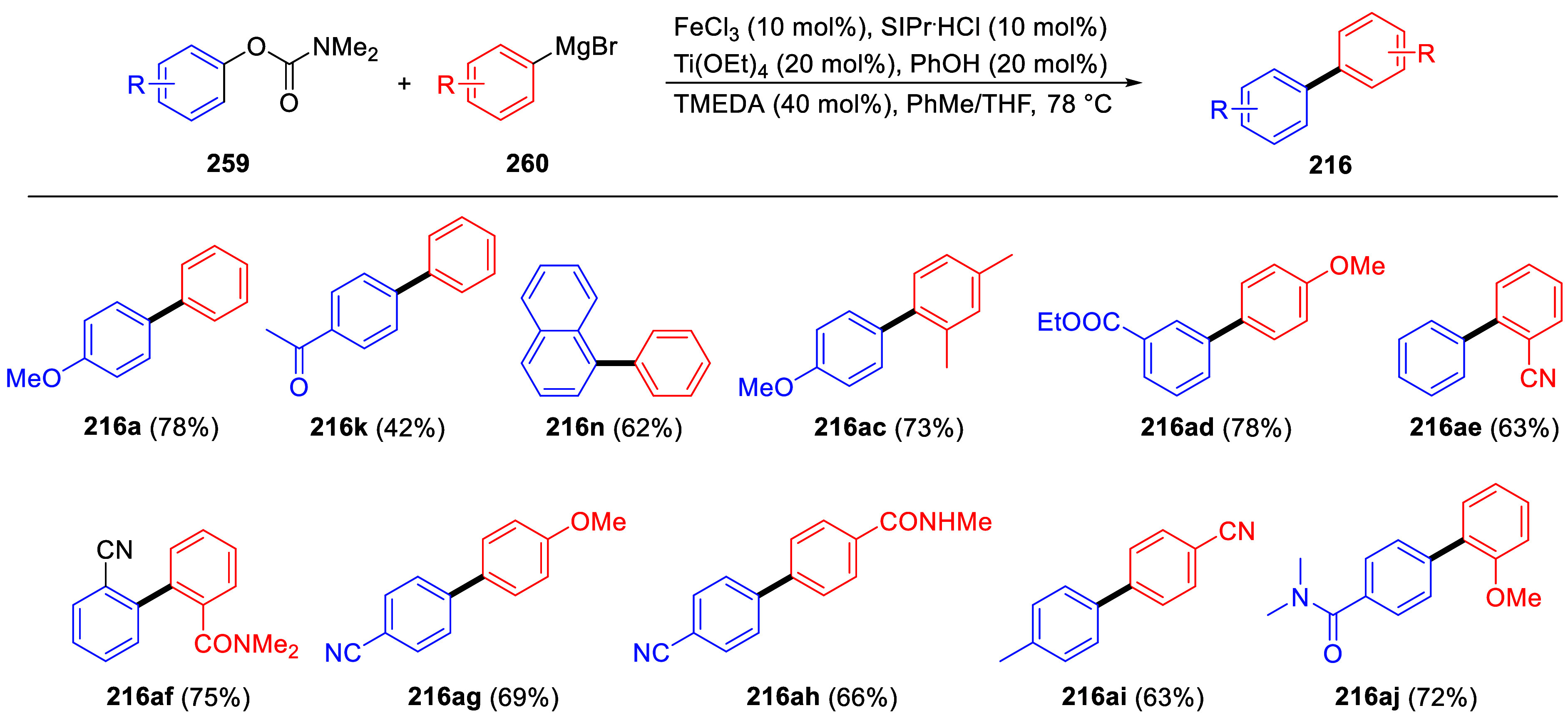
Selected Example of the Iron-Catalyzed CC of Aryl Dimethylcarbamates **259** with PhMgBr (**260**) to Give **216** and the Substrate Scope Adapted from ref (326). Copyright 2019 American Chemical Society.

Wang et al.^326^ reported a Fe/Ti-cocatalyst system that efficiently catalyzed the biaryl couplings between ArOAms and aryl Grignard reagents (Scheme 62). The catalyst system FeCl_3_/SIPr (SIPr = 1,3-bis(2,6-diisopropylphenyl)imidazolinium) with Ti(OEt)_4_/PhOMgBr transformed *O*-carbamate **259** and phenylmagnesium bromide **260** into the desired product **216a** in 78% yield. Various sensitive functional groups, including ester (**216ad**), nitrile (**216ae**,**af**,**ag**,**ah**,**ai**) and amide (**216af**,**ah**,**aj**) groups, were well tolerated on both the electrophilic (ArOAm) and nucleophilic (Ar’MgX) partners, with no notable addition of the Grignard reagents to these groups detected. CC to sterically hindered biaryls were also achieved smoothly (**216n**,**ac**,**ae**,**af**,**aj**).

Compared to aryl iodides (**261a**), ArOAm were less reactive and allowed selective coupling of iodide over OCONMe_2_ (entry 1, Table 23). On the other hand, the reactivity of the chloride **261c** was only slightly higher than that of the carbamate, resulting in a significant decrease in selectivity (entry 3).

**Table 23:**
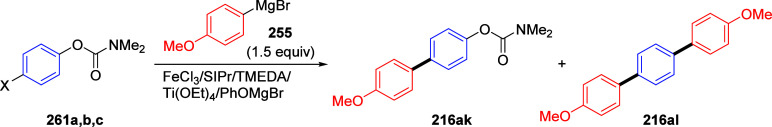
Reactivity Comparison of Selected Electrophiles in Kumada–Corriu Arylations Using an Fe/Ti Cocatalyst System^a^

entry	ArOAm	X	yield (%) of product **216ak**	yield (%) of product **216al**
1	**261a**	I	78	11
2	**261b**	Br	63	25
3	**261c**	Cl	56	31

a Adapted from ref (326). Copyright 2019 American Chemical Society.

The Kumada–Tamao–Corriu coupling reaction has also been catalyzed using a rhodium–aluminum bimetallic complex.^327^ However, prior screening showed that the *O*-carbamate was not as good a leaving group as chloride, fluoride, or the mercapto group.

### 8.4 D*o*M-Negishi CC Reactions

**Scheme 63:**
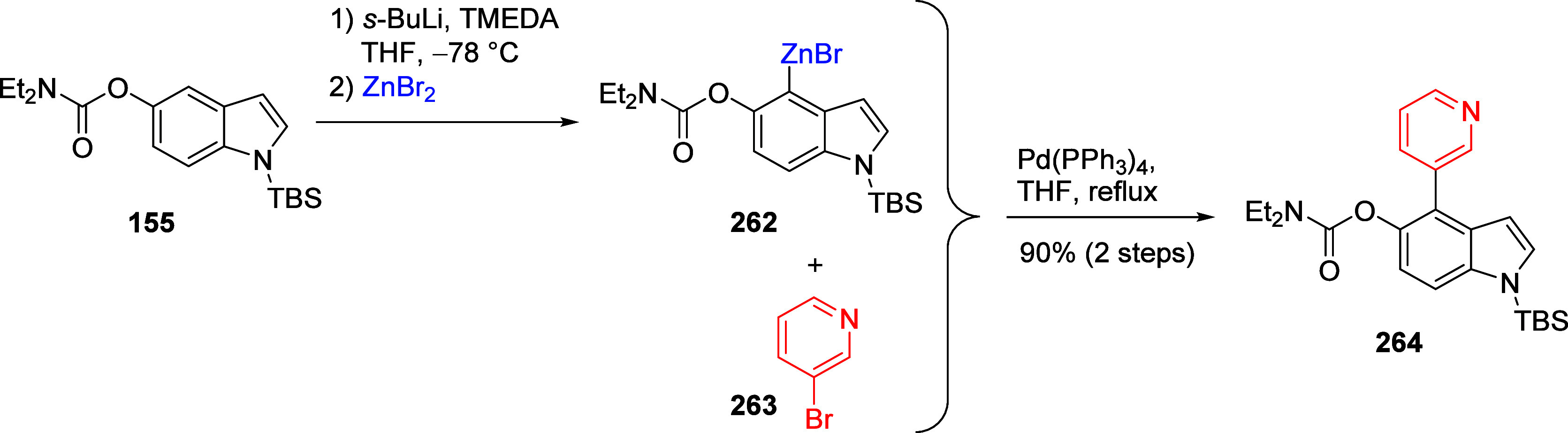
Preparation of 4-(Pyridinyl)indole **264** Adapted from ref (213). Copyright 1995 American Chemical Society.

Not surprisingly, the *O*-carbamate moiety is stable toward Pd-catalyzed Negishi CC reactions, thus providing the opportunity for chemoselective arene manipulations such as has been shown by Griffen et al.^213^ in the preparation of 4-(pyridinyl)indole **264** (Scheme 63). The coupling partner **262** was prepared by initial *s*-BuLi mediated D*o*M of **155**, quenching with ZnBr_2_.

**Scheme 64:**
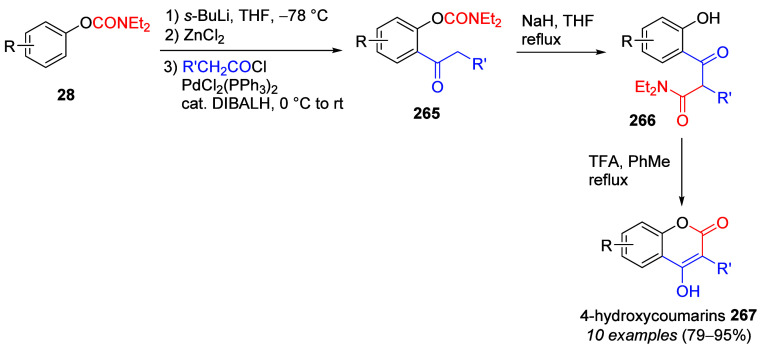
Scheme Showing the Synthesis of 4-Hydroxycoumarins **267** Commencing with ArOAms and Proceeding via a Carbamoyl Baker–Venkataraman Rearrangement Adapted with permission from ref (328). Copyright 1998 Taylor & Francis.

Kalinin et al.^328^ exploited the inertness of the ArOAm group to Negishi CC conditions further by using this to prepare arylalkyl ketones (**265**), which could then be transformed regiospecifically into substituted 4-hydroxycoumarins (**267**) via a carbamoyl Baker–Venkataraman rearrangement involving intermediate **266** (Scheme 64).

**Scheme 65:**
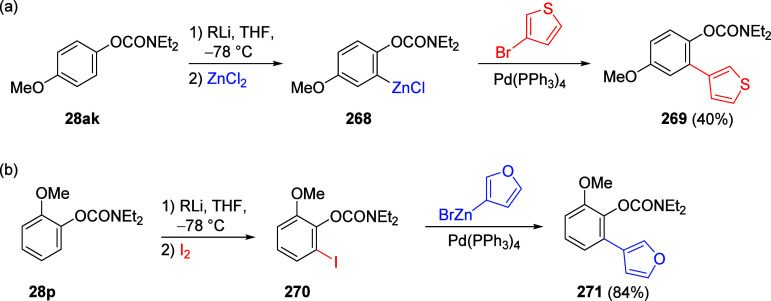
Synthesis of Representative: (a) 2-(3-Thienyl)-*O*-aryl Carbamate **269** and (b) Furyl Analogue **271** via Negishi CC Adapted with permission from ref (295). Copyright 2009 American Chemical Society.

This methodology was also used in the preparation of 2-(3-thienyl)-*O*-aryl carbamates, e.g., **269**, where the organozinc compound **268** was prepared by initial organolithium mediated D*o*M of **28ak**, quenched with ZnCl_2_ (Scheme 65a).^295^ The Negishi CC reactions to the furyl analogue **271**, in which the coupling partners were used with reversed functionality, starting from **28p** via the corresponding iodide **270**, was found to be more efficient (Scheme 65b).

### 8.5 Amine CC Reactions of ArOAm

Shimasaki et al.^329^ were the first to report the amination of an ArOAm: the nickel-catalyzed coupling of phenyl diethylcarbamate with morpholine to form 4-phenylmorpholine (Table 24, entry 1). Mesganaw et al.^330^ were the first to demonstrate this chemistry on a large scope of ArOAms and secondary amine (cyclic/acyclic) and aniline substrates using the same nickel catalyst [Ni(COD)_2_], NHC ligand (SIPr·HCl, 1,3-bis(2,6-diisopropylphenyl)imidazolium chloride), and base (NaO*t-*Bu) (Table 24, entries 2–8).^329,330^

**Table 24:**
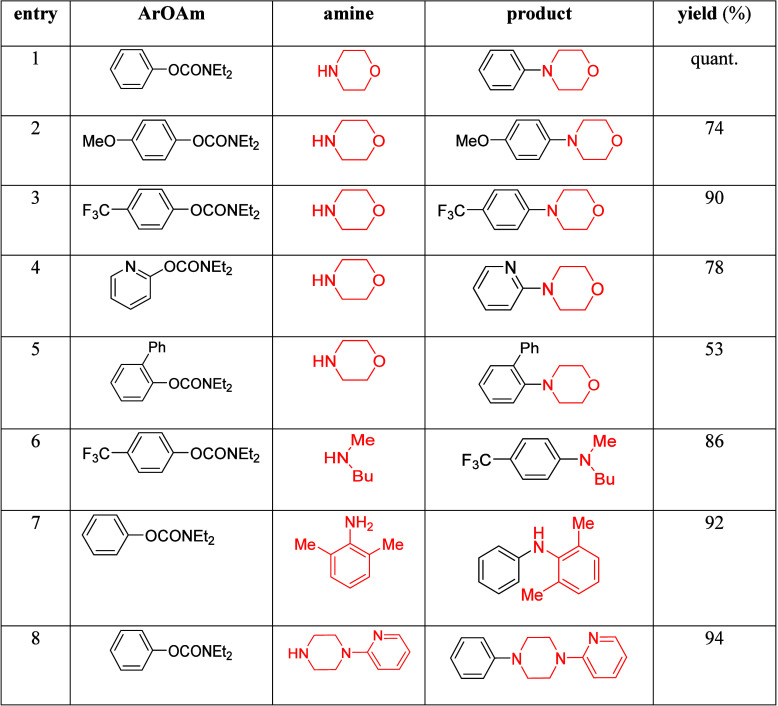
Amination of Aryl and Heterocyclic *O*-Carbamates^a^,^b^

a Reaction conditions: [Ni(COD)_2_], NHC ligand (SIPr·HCl), base (NaO*t*-Bu), toluene, 80 °C, 3 h.

b Entry 1: adapted with permission from ref (329). Copyright 2010 John Wiley and Sons. Entries 2–8 (Mesganaw et al.) adapted from ref (330). Copyright 2011 American Chemical Society.

For example, the reaction was successful with both electron-rich and electron poor ArOAms (entries 2, 3, and 6), heterocycles (entries 4 and 8), and sterically hindered carbamates (entry 5) and anilines (entry 7). A few years later, the research groups of Schranck^240^ and Stradiotto^241,242^ extended this scope to include ammonia and primary amines, both research groups using specially designed nickel(II) precatalysts. Schranck and co-workers^240^ realized the amination of naphthyl *O*-carbamates with ammonia and greener and cheaper ammonium salts (Table 25, entries 1–3), while Stradiotto’s group^241,242^ performed amination of aryl and naphthyl *O*-carbamates with ammonia and primary alkylamines (Table 25, entries 4–8).

**Table 25:**
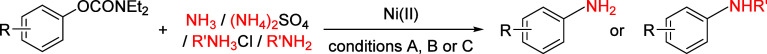

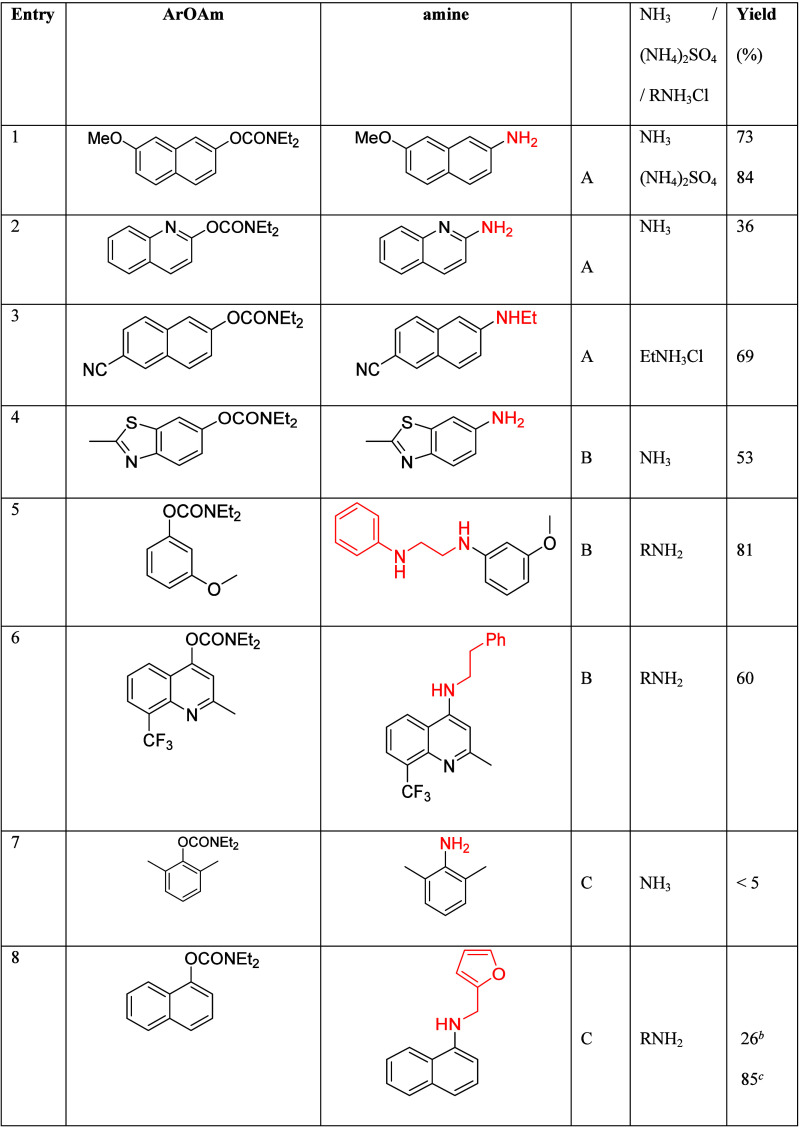
Nickel-Catalyzed Amination of ArOAms^a^,^d^

a Conditions A (Schrank et al.^240^): Nickel precatalyst (CAS no. 2049086-35-1), L, NH_3_/(NH_4_)_2_SO_4_/RNH_3_Cl, NaO*t*-Bu, *o*-xylene, 110 °C, 16 h. Conditions B (MacQueen et al.^241^): Nickel precatalyst (CAS no. 1998756-52-7), L, NH_3_/RNH_2_, NaO*t*-Bu, 1,4-dioxane, 80 °C, 16 h. Conditions C (Bodé et al.^242^): Nickel precatalyst (CAS no. 1594113-09-3/2688050-42-0), L, NaO*t*-Bu, 1,4-dioxane, 65 °C, 18 h.

b L = CyPF-Cy.

c L = CyPBn-Cy.

d Entries 1–3: Schrank et al., adapted with permission from ref (240). Copyright 2017 John Wiley and Sons. Entries 4–8: Stradiotto and coworkers, Adapted with permission from ref (241). Copyright 2017 Thieme.

**Scheme 66:**
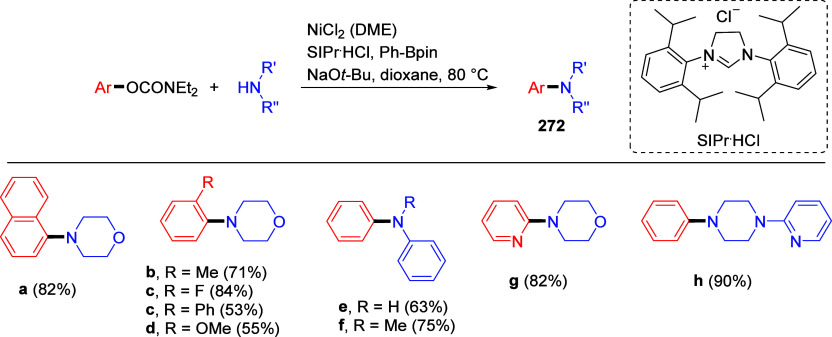
Amination of ArOAms Using Various Amines Adapted from ref (331). Copyright 2009 American Chemical Society.

Similarly, Hie et al.^331^ developed an approach to the amination of ArOAms that makes use of an air-stable Ni(II) precatalyst. In combination with a mild reducing agent (e.g., phenylboronic acid pinacol ester), this catalyst provides aminated products (select examples, **272a**–**h**) in reasonable to excellent yields (Scheme 66). As can be seen, the scope of the method is broad with respect to both coupling partners and includes heterocyclic substrates.

Similar to their work mentioned previously (section 8.2.2), Inaloo et al.^332,333^ developed recoverable and reusable magnetic Ni(0) catalysts in an effort to create a circular, more sustainable approach to C–N bond formation. Two different catalysts were prepared: nickel(0) and nickel(II) nanoparticles (NPs) immobilized on EDTA-modified Fe_3_O_4_@SiO_2_ nanospheres [Fe_3_O_4_@SiO_2_-EDTA Ni(0)]. Both catalysts were used to direct amination of (hetero)ArOAms without the need for an external ligand and reducing agent with a range of (hetero)arylamines and primary and secondary alkylamines (Table 26). For example, heterocyclic amines such as imidazole (entries 1, 6–8), indazole (entries 2 and 9), and indole (entry 10) gave the desired coupling product with various ArOAms in good yields. *N*-Arylation of nucleobases occurred in good yield (e.g., guanine, entry 3); it was interesting to note that arylation occurred exclusively over other potential coupling positions (amine groups). Aliphatic amines were successfully coupled as well, e.g., phenylmethanamine (entry 4) and dibutylamine (entry 5). Both catalysts were recovered and reused in six subsequent runs for the coupling of phenyl diethylcarbamate with pyrrole (the “model reaction”) with a reduction in yield of 3–4% by the end of the 6 runs. Thus, despite the initial synthesis and characterization, these magnetic NPs seem to be a worthwhile investment.

**Table 26:**
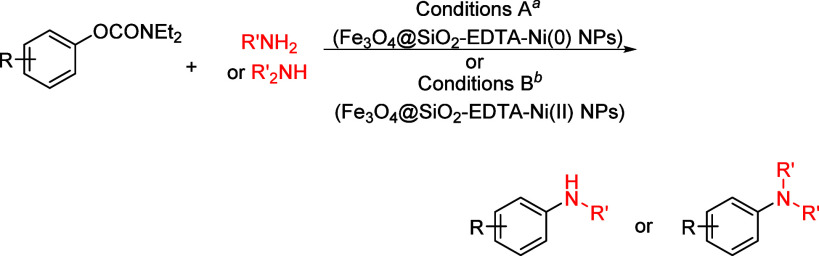

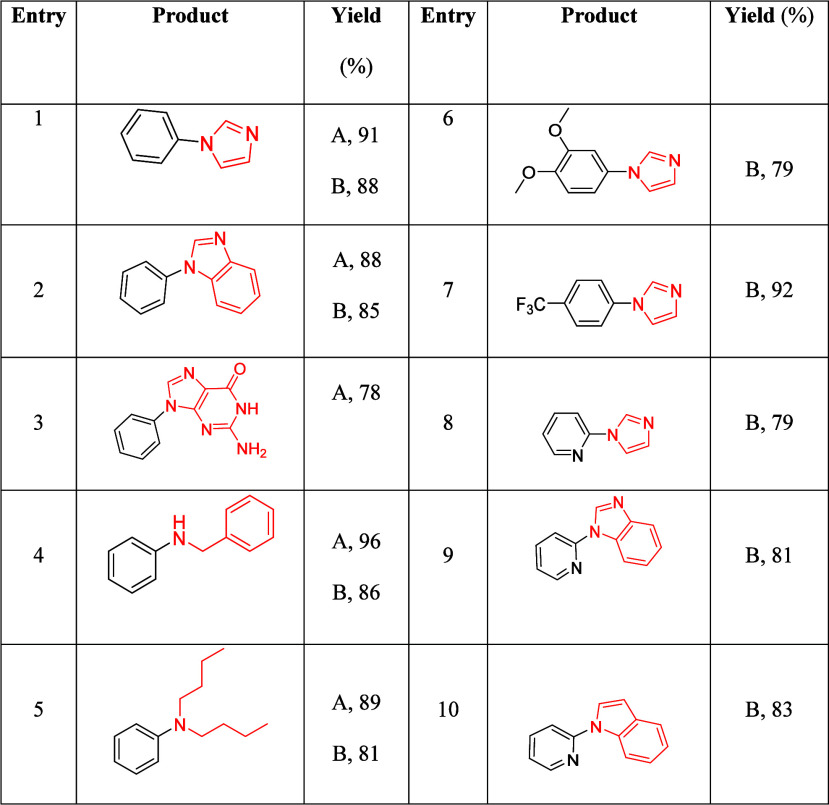
Nickel-Catalyzed Amination of ArOAms Using Fe_3_O_4_@SiO_2_-EDTA-Ni(0) and -Ni(II) NPs^c^

a Conditions A: Fe_3_O_4_@SiO_2_-EDTA-Ni(0) NPs, Ni catalyst (1 mol % Ni), NaO*t*-Bu (2 mmol), ethylene glycol (EG), 100 °C, 12 h.

b Conditions B: Fe_3_O_4_@SiO_2_-EDTA-Ni(II) NPs, Ni catalyst (1 mol % Ni), NaO*t*-Bu, EG, 100 °C, 12 h.

c Conditions A: adapted with permission from ref (332). Copyright 2020 Royal Society of Chemistry. Conditions B: adapted with permission from ref (333). Copyright 2020 Elsevier.

**Scheme 67:**
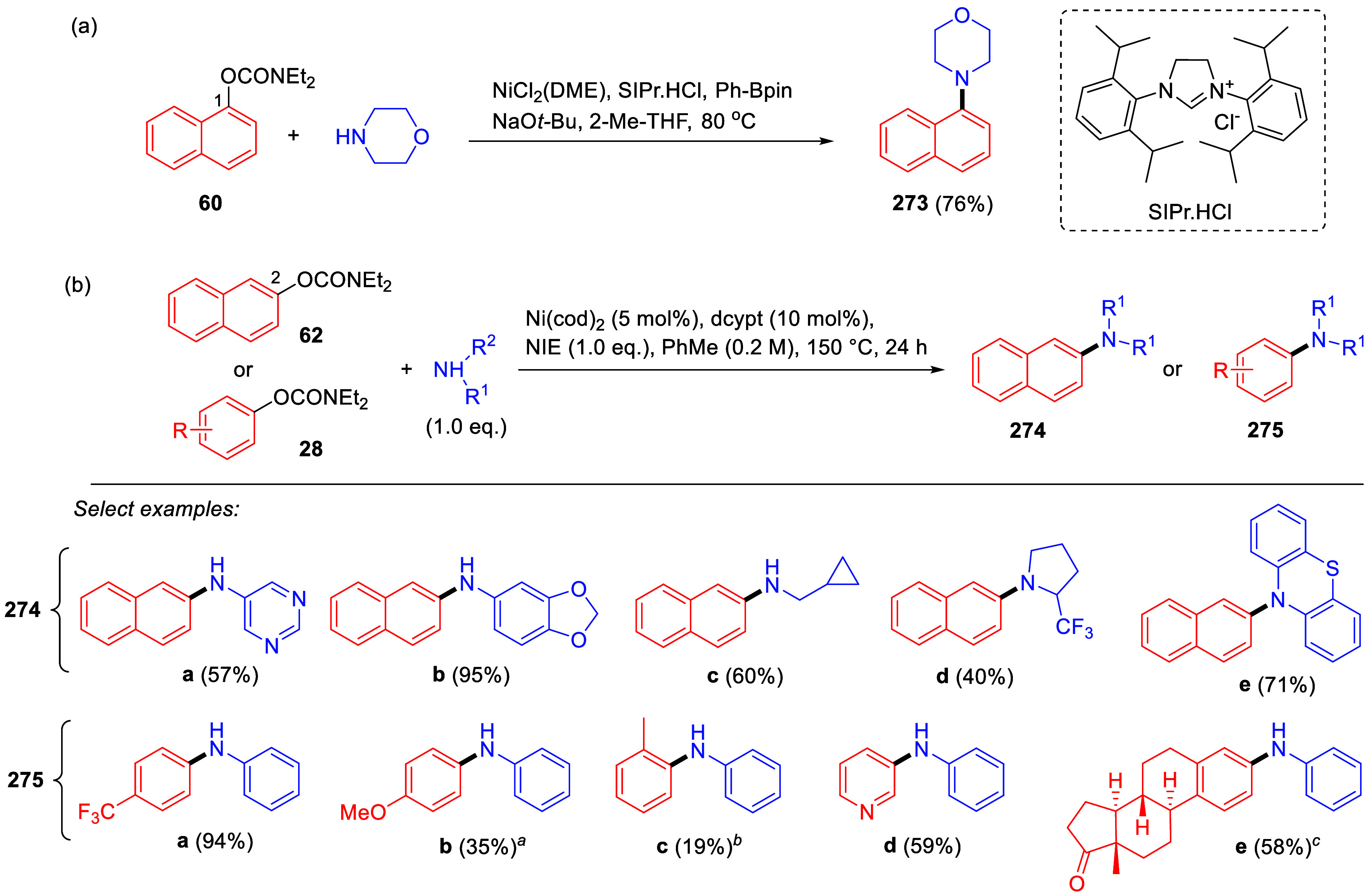
Scope of the Buchwald–Hartwig Amination Reaction with Naphthyl and (Hetero)aromatic OAms: (a) with NaO*t*-Bu (Adapted from ref (334), Copyright 2014 American Chemical Society); (b) under Exogeneous Base-Free Conditions (Adapted with Permission from ref (335), Copyright 2022 Springer Nature) (a) Ni(cod)_2_ (10 mol %), dcypt, 3,4-bis(dicyclohexylphosphaneyl)thiophene (20 mol %), NIE (2.0 equiv), amine (1.0 equiv), PhMe (0.2 M), 160 °C, 24 h. Ni(cod)_2_ (10 mol %), dcypt (20 mol %), NIE (1.5 equiv), amine (1.0 equiv), PhMe (0.2 M), 160 °C, 24 h. Ni(cod)_2_ (5 mol %), dcypt (10 mol %), amine (1.0 equiv), PhMe (0.2 M), 150 °C, 24 h.

Nathel et al.^334^ developed an amination procedure which could be performed in the green solvent 2-methyl-THF. Although the process was geared toward the amination of aryl chlorides and sulfamates, one example of the coupling of 2-naphthyl OAm with morpholine was demonstrated, forming the coupled product **273** in 76% yield (Scheme 67a).

Similarly, Toupalas and Morandi^335^ demonstrated new Buchwald–Hartwig amination conditions involving aryl and naphthyl *O*-carbamates without the use of an exogeneous inorganic base, problematic for the formation of heterogeneous reaction mixtures and are incompatible with certain substrates such as 6-bromoisoquinoline-1-carbonitrile^336^ and primary and secondary β-fluorinated alkyl amines.^337^ The major change was the use of the *O*-carbamates as “non-innocent electrophiles (NIE)”, as they contained a masked base (−N(*I*-Pr)_2_) that is released during the oxidative addition step. Substrates included diisopropyl aryl and naphthyl *O*-carbamates and a diverse set of primary as well as secondary aryl/alkyl amines to form naphthyl and aryl amines **274** and **275**, respectively (select examples shown in Scheme 67b). Aryl amines allowed for near-quantitative coupling (e.g., **274b**), while base-sensitive primary and secondary β-fluorinated alkyl amines provided reasonable yields of the corresponding products (e.g., **274d**). Electron-deficient ArOAm substrates afforded products in quantitative yield (e.g., **275a**), whereas electron-rich ones required slight adjustments to the reaction conditions to provide yields >15% (e.g., **275b**,**c**, and **275e**). The methodology was compatible with a range of FGs (e.g., phenothiazines, enabling synthesis of **274e**) and heteroaromatic ArOAms and amines (e.g., enabling the formation of **274a** and **275d**).

### 8.6 Arylations of ArOAm

#### 8.6.1 Ni/Cu-Catalyzed Coupling of Polyfluoroarenes with ArOAm

**Scheme 68:**
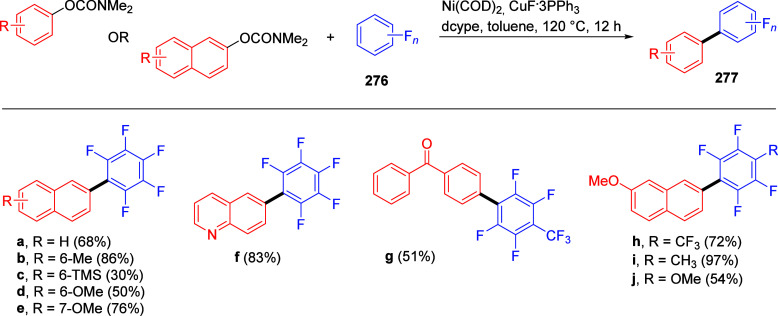
CC of Polyfluoroarenes with ArOAms by Cooperative Ni/Cu Catalysis Adapted from ref (338). Copyright 2016 American Chemical Society.

Wang et al.^338^ reported the CC of ArOAms with polyfluoroarenes using a cooperative Cu/Ni catalytic system (the Ni catalyst activates the C–O bond, while the Cu cocatalyst activates the C–H bond). Such fluorinated compounds are potentially useful due to their unique physicochemical and biological properties.^339^ Using Ni(COD)_2_ as catalyst, CuF·3PPh_3_·2EtOH as cocatalyst, and 1,2-bis(dicyclohexylphosphino)ethane (dcype) as ligand, various nonfused and fused ArOAms were reacted with a range of polyfluoroarenes (**276**), with examples shown in Scheme 68.

The methyl-substituted naphthyl *O*-carbamate provided a higher yield than the nonsubstituted analogue (**277a** versus **277b**) due to better solubility. The low yield of **277c** can be attributed to desilylation of the TMS group by the presence of fluoride anion. Also, as expected, an electron-deficient carbamate substrate (**277e**) enabled a higher yield than an electron-rich one (**277d**). Good yields were obtained for substrate quinolinyl *N*,*N*-dimethylcarbamate (**277f**), whereas the benzoylphenyl *O*-carbamate substrate required double the catalyst and ligand loading and yet **277g** was only obtained in 51% yield. The electronic character of the polyfluoroarene substrate can influence the yield (**277h**–**j**).

#### 8.6.2 Nickel-Catalyzed Arylation of ArOAm

**Scheme 69:**
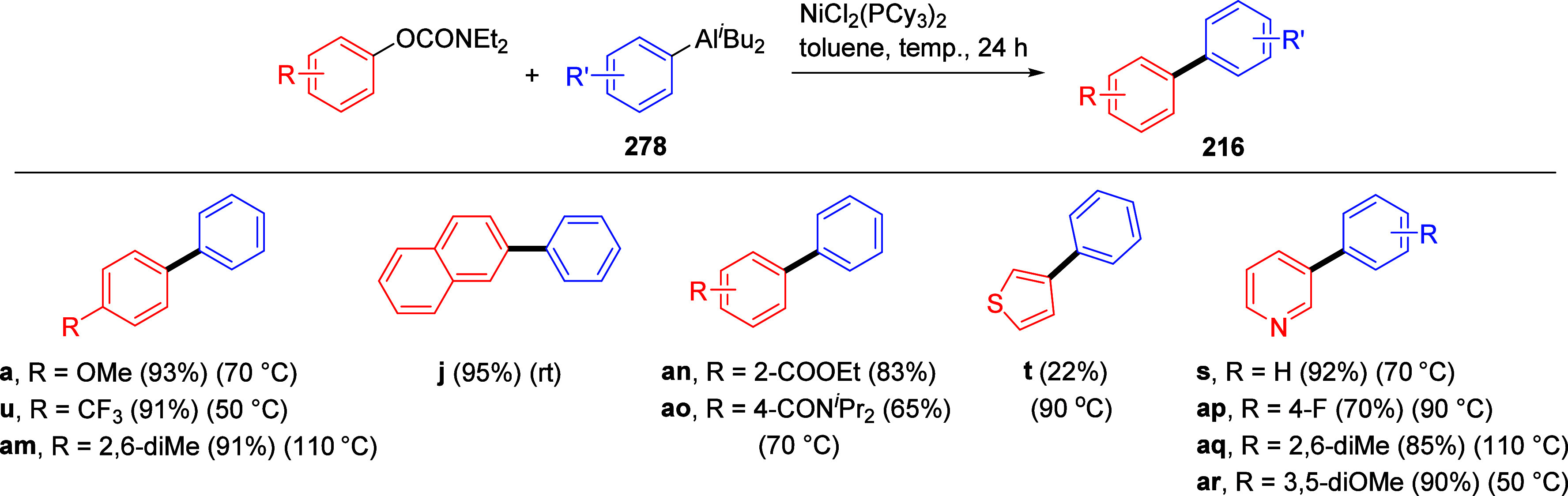
CC between Aluminum Reagents and ArOAms in the Presence of a Ni catalyst Adapted from ref (340). Copyright 2017 American Chemical Society.

Ogawa et al.^340^ developed CC chemistry between aromatic aluminum reagents **278** and ArOAms in the presence of a Ni catalyst [NiCl_2_(PCy_3_)_2_], as shown by selected substrates (Scheme 69). It was found that (1) the reactivity of the ArOAms was lower than their naphthyl counterparts (**216am**), although heating afforded biaryl products in high yields (**216a**,**u**,**am**); (2) the reaction conditions were tolerated by a variety of functional groups including methoxy (**216a**), trifluoromethyl (**216u**), ester (**216an**), amide (**216ao**), as well as various heterocycles, e.g., thiophene (**216t**) and pyridine (**216s**,**ap**,**aq**,**ar**); (3) electron-rich aromatic aluminums showed higher reactivity compared with their electron-deficient analogues (compare **216ap** with **216ar**); and (4) steric factors could be overcome with heating to achieve decent yields (**216am** and **216aq**).

It was further observed that alkenyl- and alkynyl-aluminum reagents could also be utilized for this CC, providing the corresponding products in reasonable yields (also see section 8.7).

**Scheme 70:**
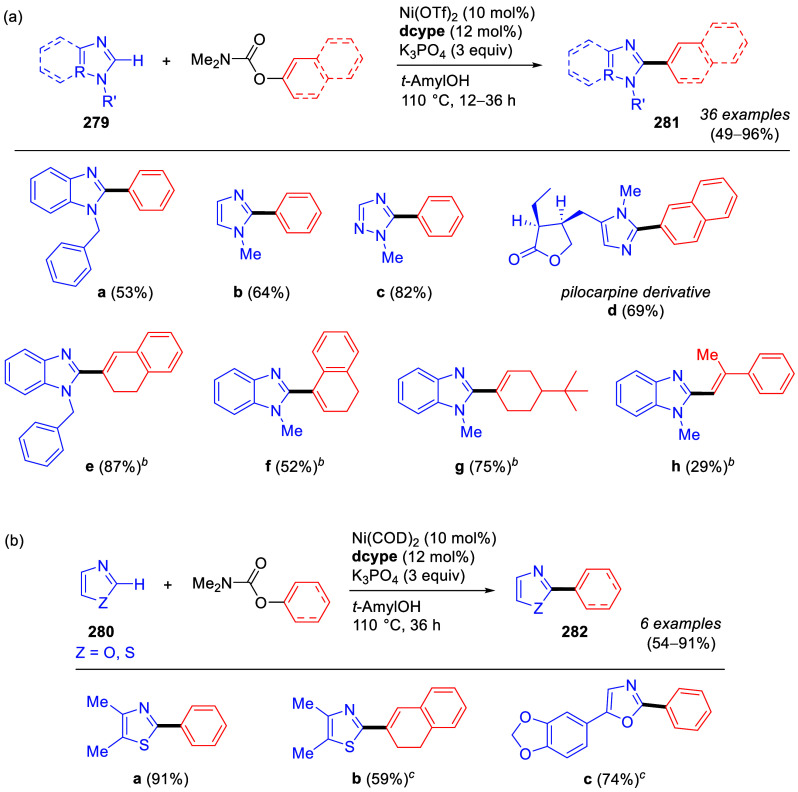
Substrate Scope of C–H Arylations and Alkenylations of (a) Imidazoles (**279**, Except for **c**, Which Is a Triazole) and (b) other 1,3-Azoles (**280**) with ArOAms *^a^* Adapted with permission from ref (341). Copyright 2015 Royal Society of Chemistry. *^b^* dcypt as ligand was used. ^*c*^ Ni(OTf)_2_ (10 mol %) and dcypt (12 mol %) were used. (dcype = 1,2-bis(dicyclohexylphosphino)ethane, dcypt = 3,4-Bis(dicyclohexylphosphino)thiophene).

Muto et al.^341^ demonstrated the first Ni-catalyzed C–H arylation and alkenylation of imidazoles (and other 1,3-azoles) **279**–**280** with ArOAms to form benzimidazoles/imidazoles/triazoles (**281a**–**h**) and thiazoles/oxazoles (**282a**–**c**) (Scheme 70). The arylations/alkenylations with ArOAms was accomplished using an Ni(OTf)_2_/diphosphine/K_3_PO_4_/*t*-amylOH system. The choice of diphosphine ligand determined the efficiency with which the arylation or alkenylation occurred. 1,2-Bis(dicyclohexylphosphino)ethane (dcype) facilitated the C–H arylations, while 3,4-bis(dicyclohexylphosphino)thiophene (dcypt) promoted C–H alkenylations. Notably, the success of the couplings was dependent on the use of a tertiary alcohol solvent, enabling the use of an air-stable Ni (II) salt (acts as the catalyst precursor).

**Scheme 71:**
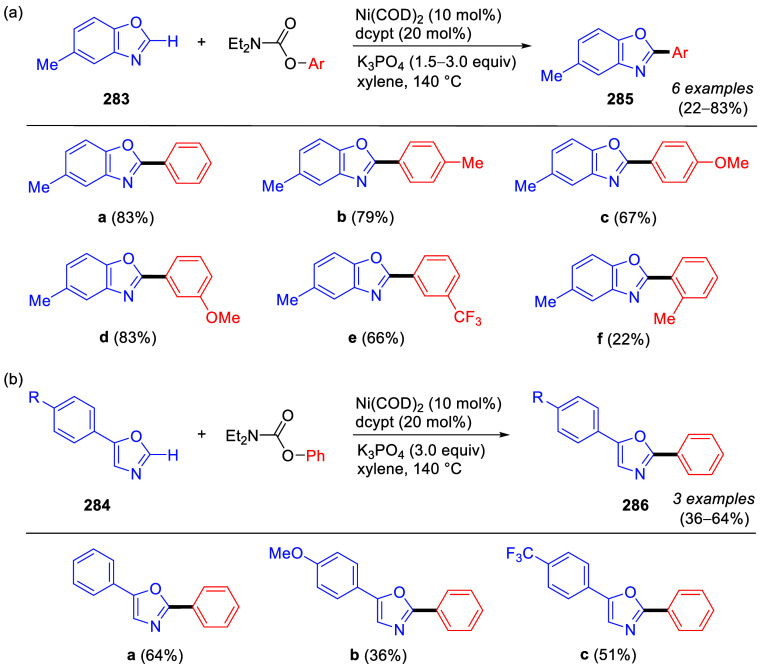
Substrate Scope of C–H Arylations of (a) Benzoxazoles **283** and (b) Oxazoles **284** with ArOAms into **285** and **286**, Respectively (dcypt = 3,4-Bis(dicyclohexylphosphino)thiophene) Adapted with permission from ref (342). Copyright 2017 Elsevier.

Steinberg et al.^342^ showed that it is also possible to cross couple ArOAms with benzoxazoles **283** and oxazoles **284** (Scheme 71). Under the optimal conditions, ArOAms performed similarly to the corresponding pivalates and mesylates.

#### 8.6.3 Cobalt-Catalyzed Arylation of ArOAm

**Scheme 72:**
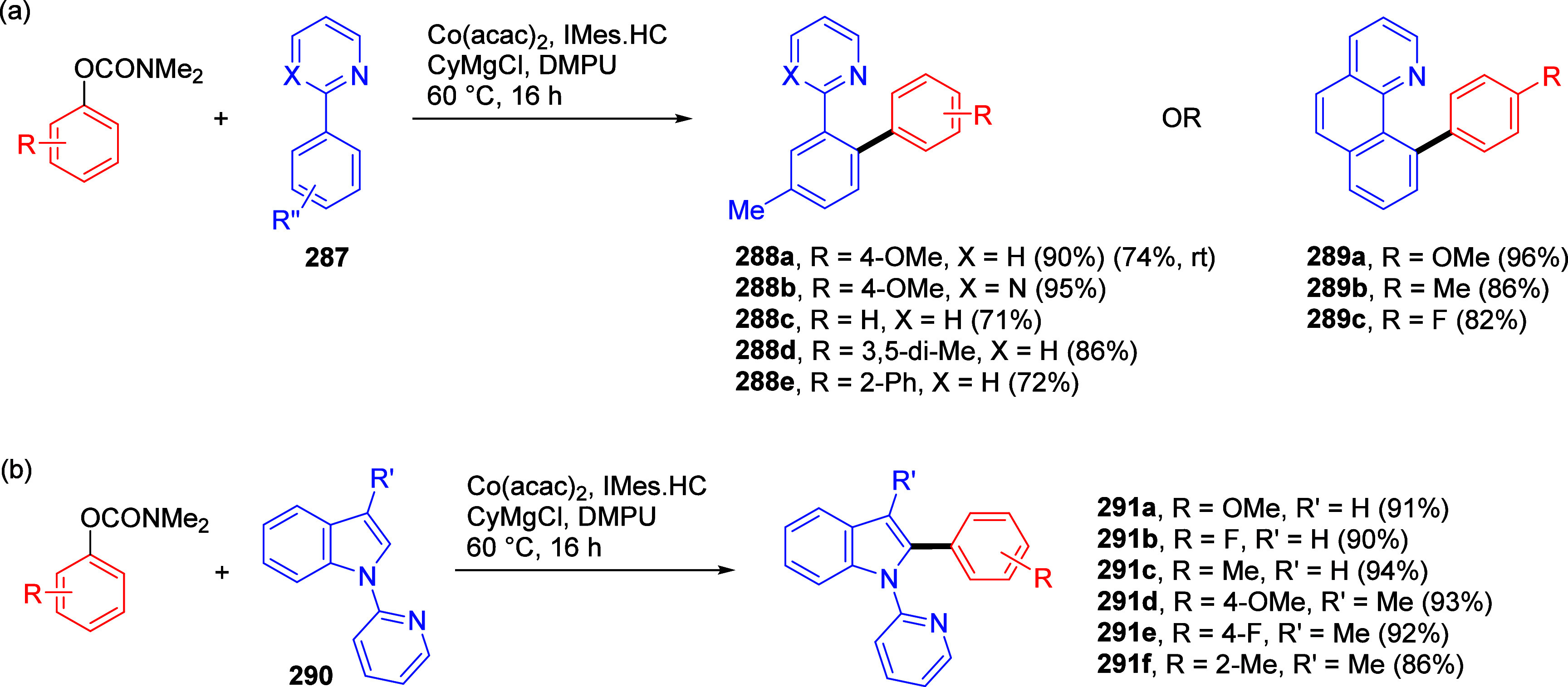
Cobalt-Catalyzed Direct Arylation of Heteroarenes: (a) Phenylpyridines/Phenylpyrimidines and (b) 1-(Pyridin-2-yl)-1*H*-indoles, with ArOAms to Form Coupled Products **288**, **289**, and **291** Adapted with permission from ref (343). Copyright 2012 John Wiley and Sons. DMPU = 1,3-dimethyl-3,4,5,6-tetrahydro-2-pyrimidinone.

Song and Ackermann^343^ used inexpensive cobalt catalysts to perform arylations of (hetero)arenes with ArOAms over a wide scope of substrates, including phenylpyridines and pyrimidines **287** and indoles **290**, with select examples shown in parts a and b of Scheme 72.

Arenes with electron-donating and electron-withdrawing substituents enabled synthesis of the desired products **288**, **289**, and **291** in overall high yields. The synthesis of **288a** proceeded in a 74% yield at room temperature, while the synthesis of phenylbenzo[*h*]quinolines **289** using cobalt catalysts gave yields equivalent to or higher than when using them when prepared with rhodium^344−346^ or ruthenium^347,348^ catalysts. Different pyridyl-directing groups as well as a less electron-donating 2-pyrimidyl substituent ensured selectivity at the C2 position (**288b**–**e**). Similarly, mono-*N*-substituted indoles were also selectively arylated at the C2 position, enabling the synthesis of sterically encumbered **291f**.^324^

#### 8.6.4 Rhodium-Catalyzed Arylation of Aryl Oxazoles

**Scheme 73:**
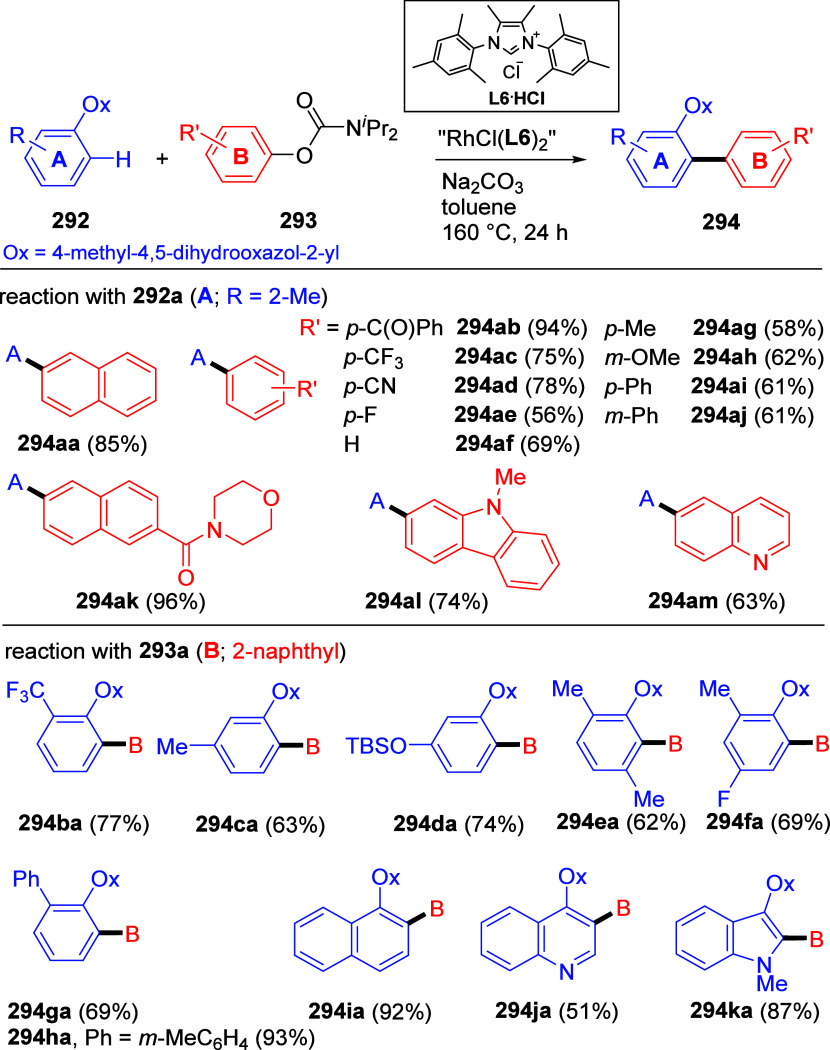
Arylation of a Series of Aryl Oxazoles **292** with Various ArOAms **293** Using *in Situ* Generated Rhodium Bis(NHC) Catalyst Adapted with permission from ref (349). Copyright 2017 John Wiley and Sons.

The research groups of Tobisu and Chatani^349^ developed chemistry enabling the arylation of aryl oxazoles using ArOAms as arylating agents (Scheme 73).

Key to the procedure was the *in situ* generation of a rhodium bis(NHC) catalyst, needed for activation of the inert C(sp^2^)–O bond of the ArOAms. A range of substrates could be coupled together, from aryl carbamates with a range of functional groups, including ketones (**294ab**), nitriles (**294ad**), fluorides (**294ac** and **294ae**), amides (**294ak**), and heteroaromatic carbamates (**294al** and **294am**), to arenes where arylation occurs exclusively at the less hindered C–H bond (**294ca** and **294da**), while a sterically congested C–H bond can be arylated if it is the only reactive site (**294ea**). This arylation is also applicable to fused arenes (**294ia**) and heteroarenes (**294ja** and **294ka**). The 4-methyl-4,5-dihydrooxazol-2-yl (Ox) group used as the directing group is easily converted to the carboxylic acid derivative, which allows further synthetic elaboration of the C–H/C–O coupling products. Because the carbamate group is completely stable under standard palladium-catalyzed CC conditions, further sequential functionalization of the C–X and C–O bonds is possible.

### 8.7 Alkynylation of ArOAm

**Scheme 74:**
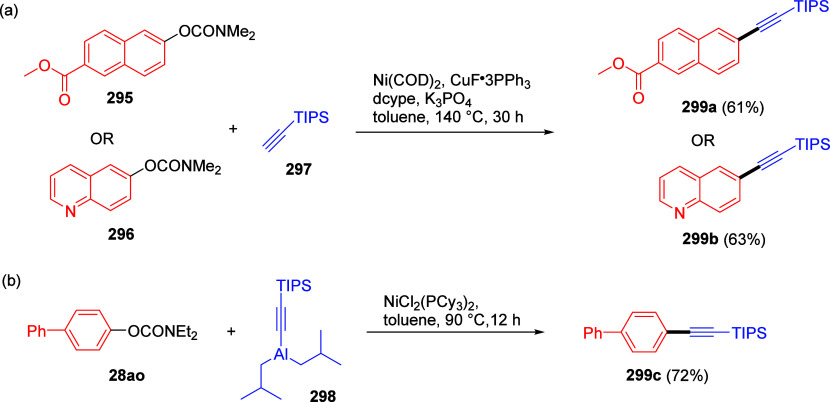
Extension of the (a) Cooperative Ni/Cu Catalytic System to the CC of Naphthyl *O*-Carbamates with Alkynes (Adapted from ref (338), Copyright 2016 American Chemical Society) and (b) Use of Organoaluminum Reagents to Prepare Phenyl-Based Alkynes (Adapted from ref (340), Copyright 2017 American Chemical Society)

The cooperative Cu/Ni catalytic system developed by Wang et al.^338^ (section 8.6.1) also proved to be effective for CC of terminal alkynes **297** with naphthyl *O*-carbamates, although only two substrates (**295** and **296**) were examined and under harsh reaction conditions (Scheme 74a). The organoaluminum reagent **298** developed by Ogawa et al.^340^ enabled the preparation of one phenyl alkyne **299c** in good yield using slightly milder conditions (Scheme 74b).

**Scheme 75:**
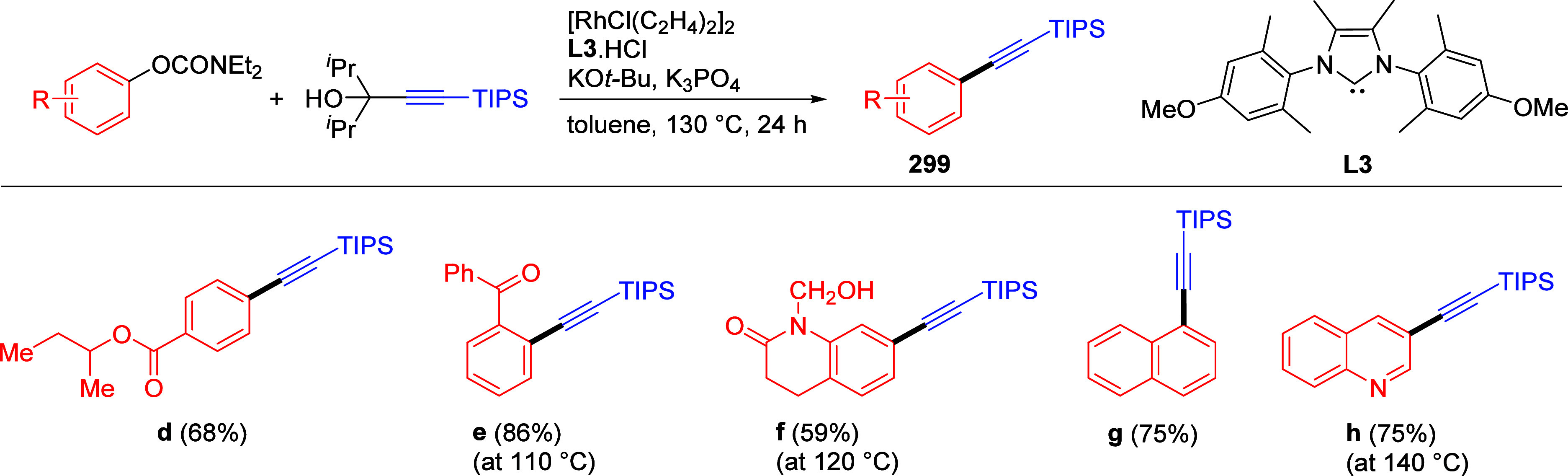
Examples of Aryl-*O*-carbamates Able to Participate in Rhodium-Catalyzed Alkynylation with Propargyl Alcohols Adapted from ref (350). Copyright 2018 American Chemical Society.

Similarly, Yasui et al.^350^ reported the alkynylation of 15 diverse substrates, e.g., containing esters (**299d**), ketones (**299e**), and amides (**299f**), typically incompatible with organometallic alkynylating reagents (Scheme 75).^292^ Naphthyl and heteroaromatic *O*-carbamates (e.g., quinoline) also provided the corresponding alkynylated products (**299g**,**h**).

### 8.8 Alkylation of ArOAm

**Scheme 76:**
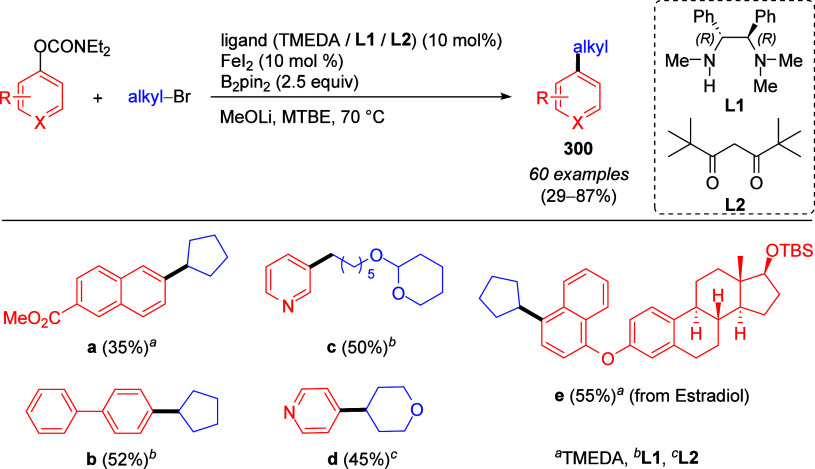
Example of Products Formed during the Alkylation of (Hetero)aryl-*O*-carbamates with Alkyl Bromides Adapted with permission from ref (351). Copyright 2023 Chinese Chemical Society.

Chen et al.^351^ demonstrated cross-electrophile coupling of (hetero)ArOAms with alkyl bromides, catalyzed by an iron/B_2_pin_2_ catalytic system to form alkylated (hetero)aryl compounds **300** (Scheme 76).

This reaction was found to proceed under mild conditions, enabling good functional group tolerability, as exemplified by the ester **300a**. Alkylated pyridines **300c** and **300d** were produced by the *meta*- and *para*-alkylations of pyridine *O*-carbamates, respectively. Late-stage functionalization was demonstrated for five bioactive compounds, e.g., **300e** (alkylation of estradiol with a cyclopentyl group). As was seen in the preceding example (Scheme 76), the type of reaction depended on the ligand used. Three different ligands were explored: TMEDA, (1*R*,2*R*)-*N*^1^,*N*^1^,*N*^2^-trimethyl-1,2-diphenylethane-1,2-diamine (**L1**) and (2,2,6,6-tetramethylheptane-3,5-dione) (**L2**). TMEDA was effective for over half of the transformations, while **L1** (containing an unprotected primary amine group) was effective mainly for biphenyl *O*-carbamates (e.g., synthesis of **300b**) and pyridines with the *O*-carbamate at the *meta* position (e.g., to form **300c**), while **L2** was only effective for facilitating transformations involving pyridines with the *O*-carbamate group at the *para*-position, e.g., to form compound **300d**.

**Scheme 77:**
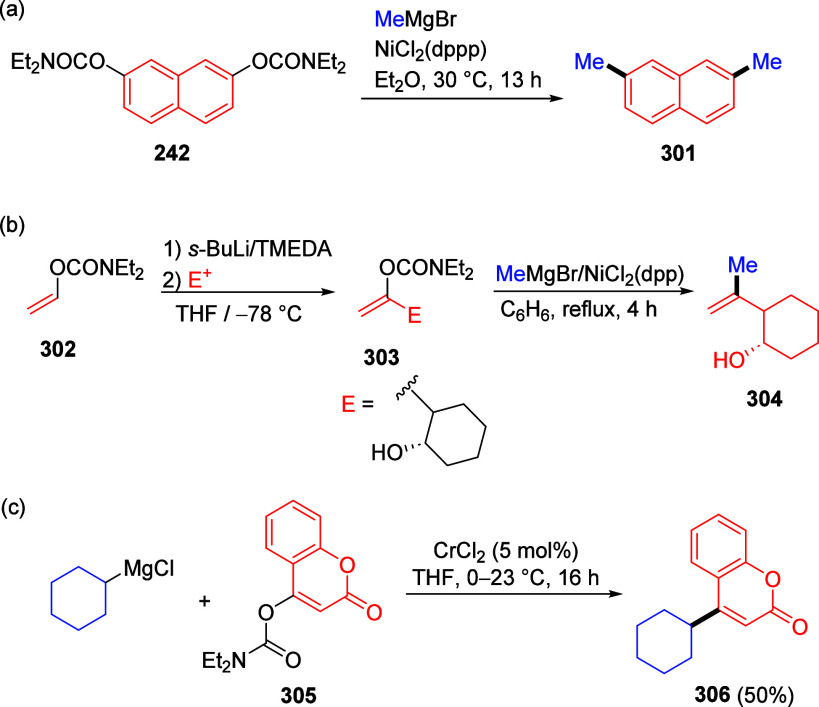
(a) Ni(0)-Catalyzed Kumada–Corriu CC of **242** with MeMgBr to Form 2,7-Dimethylnaphthalene **301** (Adapted with Permission from ref (352), Copyright 2021 Royal Society of Chemistry); (b) an Example of a Vinylic D*o*M Reaction Followed by a Kumada–Corriu CC Reaction with Methylmagnesiumbromide (MeMgBr) to Form Prop-1-en-derivative **304** (Adapted from ref (353), Copyright 1990 American Chemical Society); (c) Cr(II)-Catalyzed Grignard CC Reaction of **305** with *c*-Hex-MgCl to Form **306** (Adapted from ref (354), Copyright 2019 American Chemical Society)

A striking feature of the *O*-carbamate group is that it can also act as a leaving group; this was demonstrated by Leith et al.^352^ in the Ni(0)-catalyzed Grignard methylation of naphthyl *O*-carbamate (**242**), providing 2,7-dimethylnaphthalene **301** in the quest to prepare corannulene-based materials (Scheme 77a) (regrettably no yield for this reaction was provided). This aspect was first discovered by Sengupta and Snieckus,^353^ albeit with a vinylic substrate **302** (via **303**) to form **304** (Scheme 77b). A similar reaction was carried out by Li et al.^354^ on an alkenyl *O*-carbamate (**305**) using a CrCl_2_ catalyst with cyclohexylmagnesium chloride (*c*-Hex-MgCl) as alkylating agent (Scheme 77c). It would be interesting to see if this chemistry could be extrapolated to ArOAms and perhaps with other alkyl magnesium bromides, e.g., EtMgBr.

### 8.9 CC Reactions with Aryl Silane Reagents

**Scheme 78:**
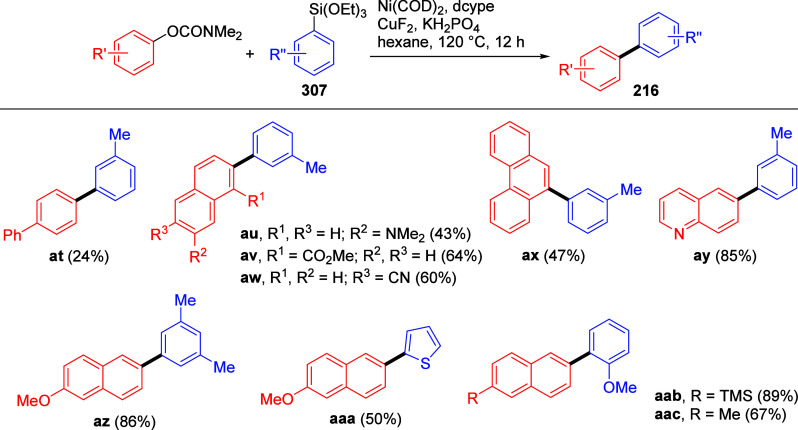
Examples Showing Scope of Aryl and Naphthyl *O*-Carbamate Substrates Coupling with Arylsilanes Adapted with -ermission from ref (311). Copyright 2016 John Wiley and Sons.

Shi et al.^311^ extended nickel-catalyzed Stille CC of C–O electrophiles (aryl sulfamates, tosylates, and mesylates, to nickel-catalyzed CC of ArOAms with arylsilanes (**307**) to yield biaryl compounds (select examples shown in Scheme 78). This new coupling reaction demonstrates good functional group tolerance, e.g., toward amines (**216au**), esters (**216av**), nitriles (**216aw**), quinolines (**216ay**), thiophenes (**216aaa**), and TMS (**216aab**), includes nonfused ArOAms (**216at**) and fused aromatics (**216ax**), and is not affected negatively by steric hindrance on the ArOAm (**216au**) or arylsilane (**216aab**).

**Scheme 79:**
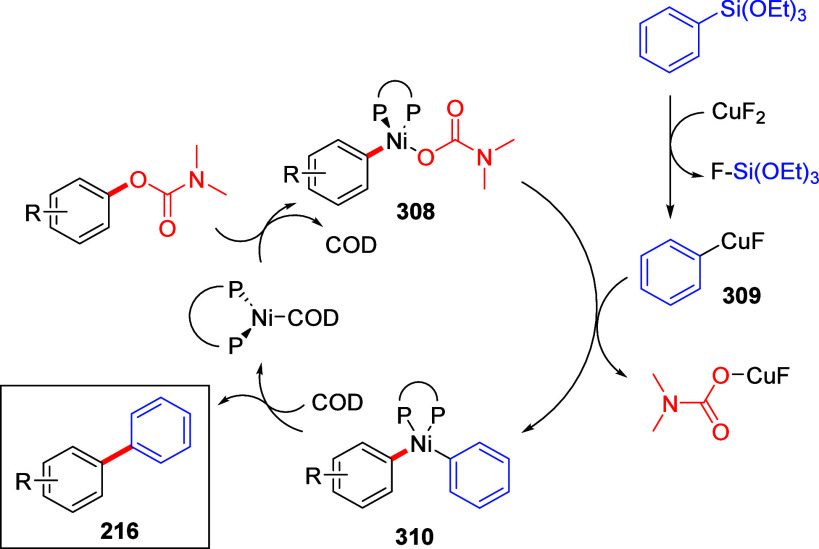
Proposed Mechanism of the Stille CC between ArOAms and Aryl Silanes, Showing the Roles Played by Ni(COD)_2_ and the CuF_2_ Adapted with permission ref (311). Copyright 2016 John Wiley and Sons.

The key to the success of these reactions was the use of CuF_2_ to facilitate the transmetalation step of silicon reagents (to form more reactive organocopper reagents, as **309**), shown in Scheme 79, and which has been observed in other similar scenarios.^355−357^

## 9 OAm in the Synthesis of Bioactive Molecules, Natural Products, and Polyaromatic Hydrocarbons (PAHs)

The methodology and utility of the OAm group presented in the prior sections has culminated in the synthesis of valuable end products in the form of bioactive molecules, natural products, and PAHs over the past decades. This section provides a summary of some of the compounds and compound architectures that have been prepared from substrates containing the OAm group. For more details and to compare the utility of OAm with other DMGs, we refer the reader to other in-depth publications.^135,358−360^

**Figure 7:**
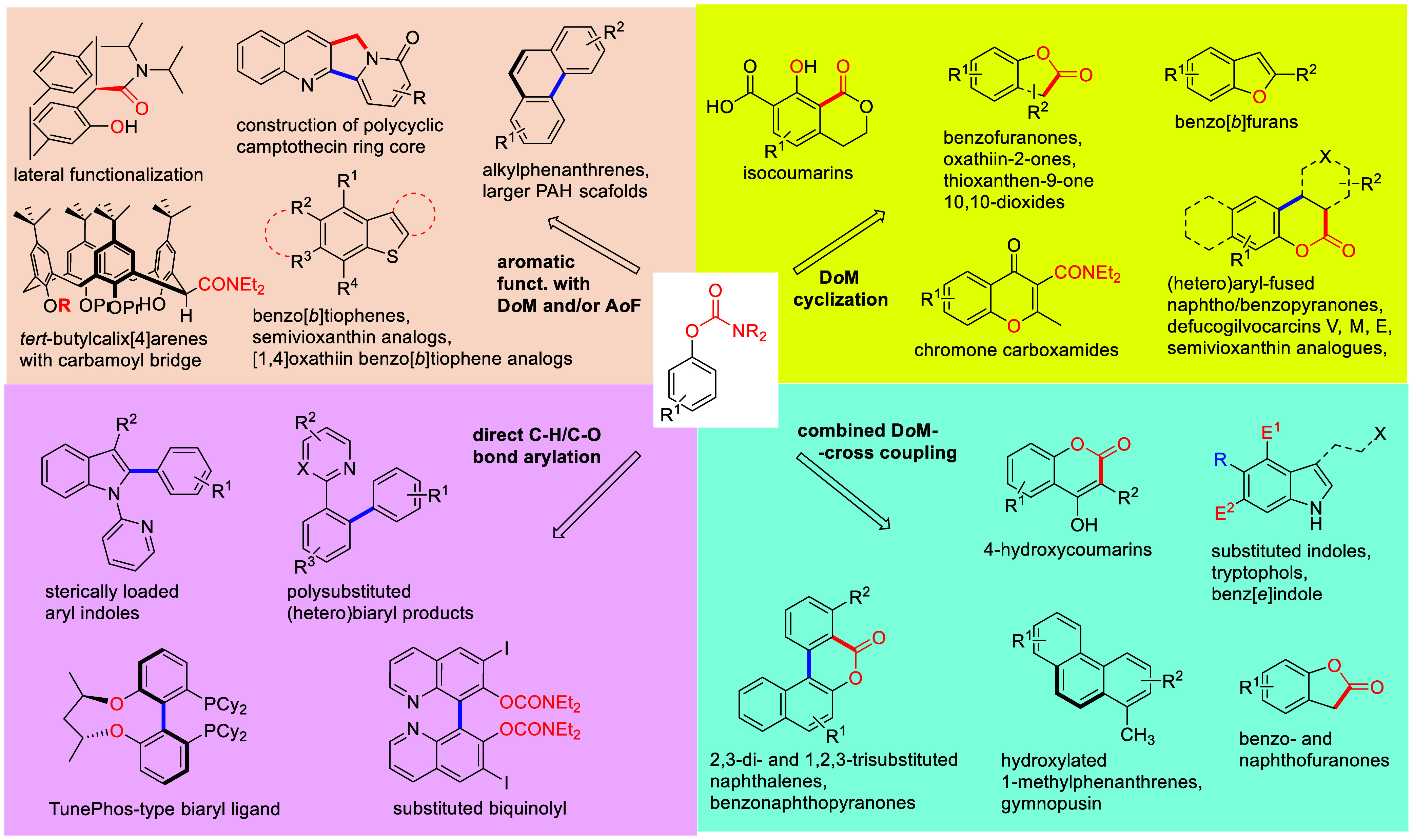
Summary of different ways in which ArOAm has been utilized in the synthesis of complex molecules and PAHs.

We have classified the various reactions into four main pathways, namely: (1) regioselective aromatic functionalization with D*o*M and/or A*o*F rearrangement, (2) D*o*M followed by annulation/cyclization, (3) combined D*o*M-CC reactions, and (4) direct C–H/C–O bond arylation (see overview in Figure 7 below).

### 9.1 Regioselective Aromatic Functionalization with D*o*M and/or A*o*F Rearrangement

**Scheme 80:**
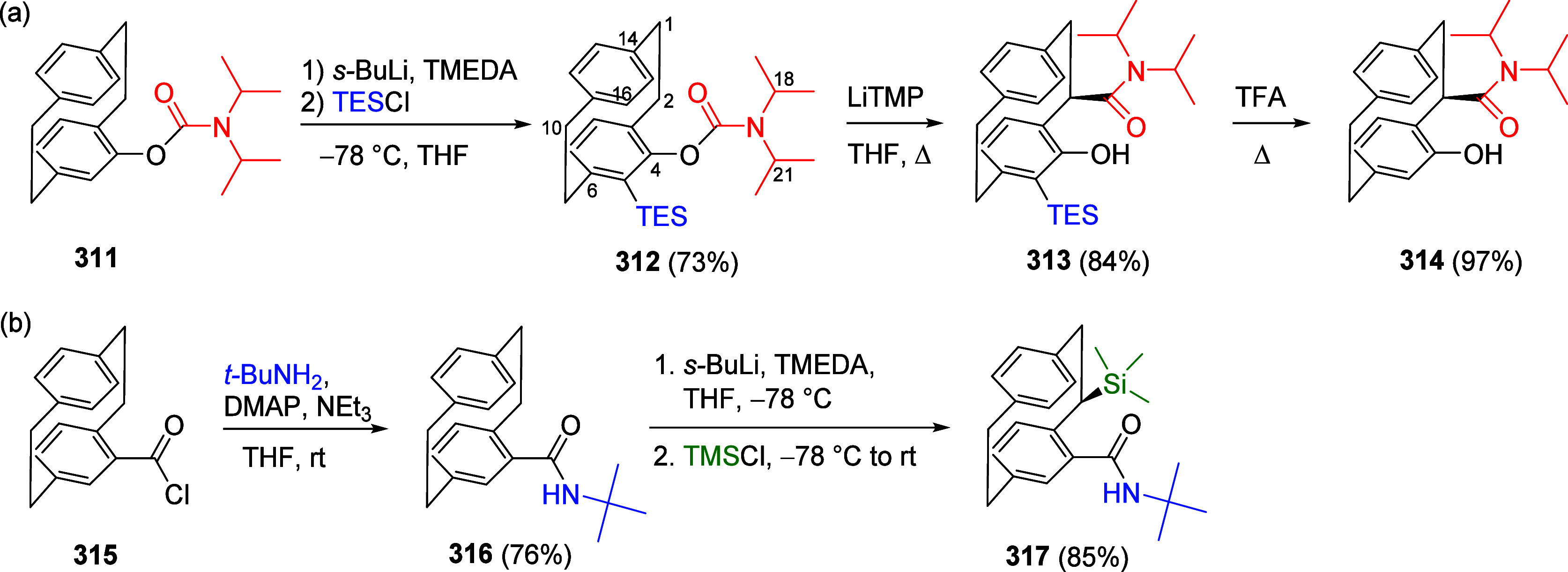
Two Routes for the Lateral Functionalization of Monosubstituted [2.2]Paracyclophanes **311**: (a) Protection of the *ortho* Site of **311**, A*o*F Rearrangement, and Deprotection Gives **314** in Excellent Yield; (b) D*o*M Reaction of **316** Gives Only the *syn*-Isomer **317** in High Yield Adapted with permission from ref (361). Copyright 2001 John Wiley and Sons.

Focken et al.^361^ developed two routes for the lateral functionalization of monosubstituted [2.2]paracyclophanes. After protecting the *ortho* site of an *O*-([2.2]paracyclophanyl) diisopropylcarbamate **311**, a remote Fries rearrangement led to the substitution of the benzylic position and gave *syn*-4-hydroxy-*N*,*N*-diisopropyl-5-triethylsilyl-2-[2.2]paracyclophanecarboxamide (**313**) in excellent yield and with a *syn*/*anti* diastereoselectivity of more than 99:1 (Scheme 80a). An alternative route involved direct functionalization of the lateral position of *N*-*tert*-butyl-4-[2.2]paracyclophanecarboxamide (**316**) by directed metalation. The reaction is highly stereoselective, and only *syn* isomer **317** is formed in high yields (Scheme 80b).

**Scheme 81:**
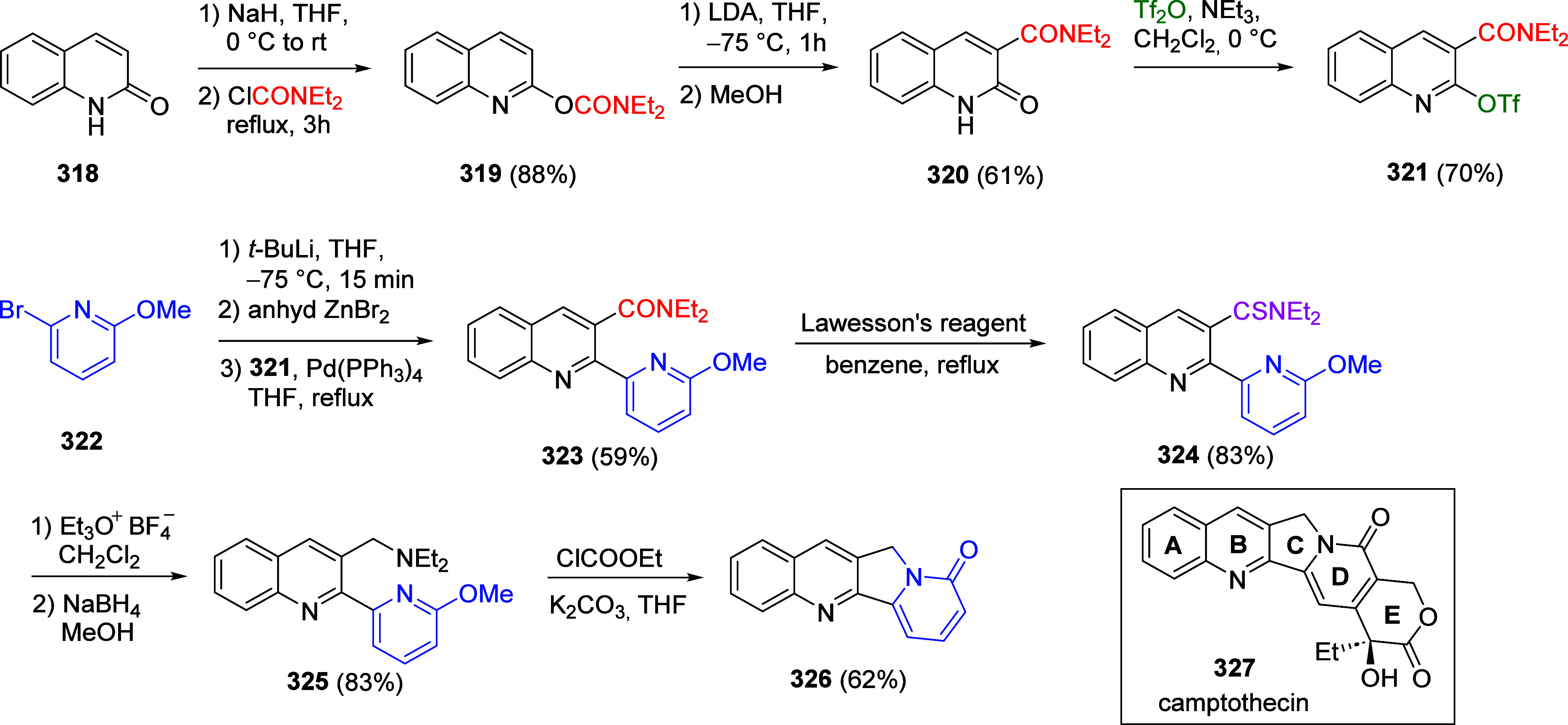
Synthesis of the Tetracyclic A/B/C/D Ring Core of the Antitumor Agent Camptothecin (**327**) Using a Combined D*o*M/Transition Metal-Catalyzed CC Approach as Key Steps in the Synthesis Adapted from ref (362). Copyright 2004 American Chemical Society.

Nguyen et al.^362^ synthesized the tetracyclic A/B/C/D ring core of the antitumor agent camptothecin (**327**) using a combined D*o*M/transition metal-catalyzed CC approach in seven steps and 11% overall yield (Scheme 81). The design is based on the initial coupling of **321**, prepared by A*o*F rearrangement of quinoline 2-*O*-carbamate **319**, with organozinc species (formed from **322** and ZnBr_2_) to give **323**, leading to the formation of the C-ring by simple functional group manipulation and cyclization (**326**). The simplicity of the steps, the ready availability of starting materials for both A/B- and D-ring fragments by D*o*M chemistry, and the potential for further modifications of the D-ring allow further synthetic studies of camptothecin.

**Scheme 82:**
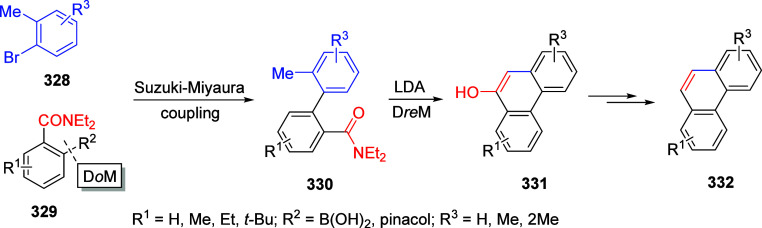
Regiospecific Synthesis of Alkylphenanthrenes **332** Using a Combined D*o*M/Suzuki–Miyaura CC/D*re*M Protocol Adapted with permission from ref (363). Copyright 2004 Canadian Science Publishing.

Cai et al.^363^ used a combined D*o*M/Suzuki–Miyaura CC/D*re*M approach that provides short, efficient, and regioselective synthesis of alkylphenanthrenes **332** in four to seven steps and 21–36% overall yield (Scheme 82). This general strategy provides the phenanthrene class of PAHs as single isomers in preparative amounts and high purity that can be used as analytical standards.

**Scheme 83:**
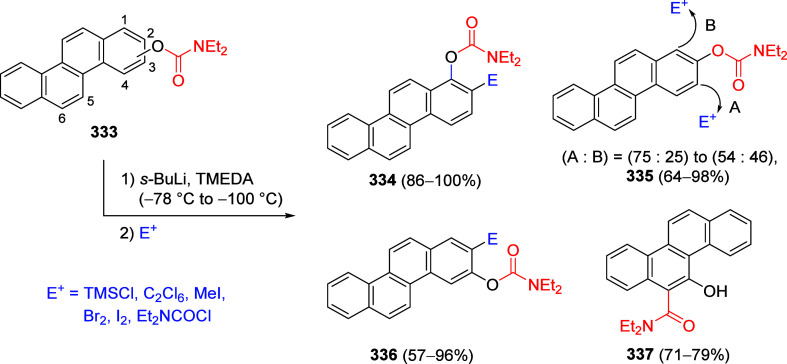
Derivatization of Chrysene, **333**, One of the Larger PAH Scaffolds Using the D*o*M Strategy Adapted from ref (87). Copyright 2018 American Chemical Society.

Kancherla et al.^87^ demonstrate the utility and performance of the D*o*M strategy for the synthesis and derivatization of other larger PAH scaffolds such as chrysene **333** (Scheme 83). The starting *O*-carbamates **333** were prepared from the corresponding chrysenols available by oxidative photochemical cyclization or D*re*M tactics. Chrysen-1-yl and chrysen-3-yl ring site selectivity of D*o*M and A*o*F rearrangement protocols with *s*-BuLi/TMEDA followed by electrophilic quenching using a selection of electrophiles was observed and led to new chrysenyl derivatives **334**–**337**. 5-Chrysenyl *N*,*N*-diethyl-*O*-carbamate underwent an immediate A*o*F rearrangement to chrysenyl *o*-hydroxycarboxamide **337** even at −100 °C.

**Scheme 84:**
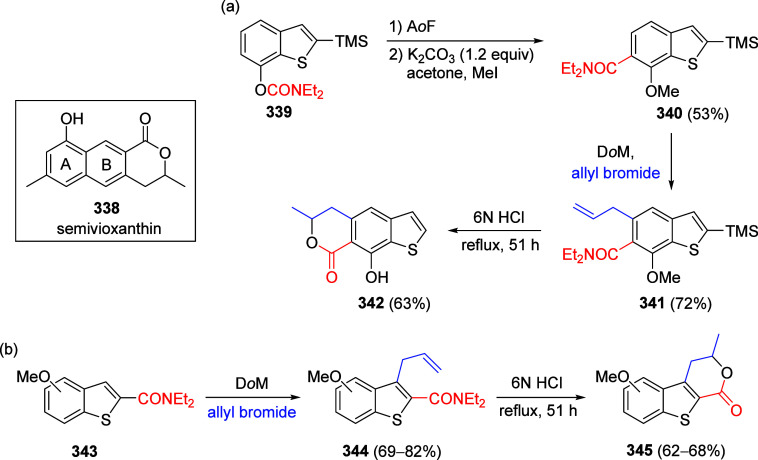
Examples of the Usefulness of the D*o*M Method in the Synthesis of Sulfur Analogues of Biologically Active Semivioxanthines (**338**) Adapted from ref (364). Copyright 2005 The Japan Institute of Heterocyclic Chemistry.

The synthesis of sulfur analogues of the biologically active linear naphthopyrone semivioxanthin (**338**) is an example of the usefulness of the D*o*M method introduced by Pradhan and De.^364^ Analogue (**342**) of semivioxanthin, in which ring A of the natural product was replaced by thiophene, was synthesized in high overall yield (68–72%) from **339**, using an A*o*F and transmetalation protocol (Scheme 84a). Both demethylation and desilylation occurred during cyclization to give an air-stable compound **342**. In another example of semivioxanthin analogues, ring B of the natural product was replaced by thiophene (**345**) (Scheme 84b). The starting material (**343**) was subjected to a D*o*M and acid-induced cyclization protocol accompanied by demethylation of the methoxy groups. The air sensitive phenolic compounds were immediately *O*-methylated in high yield to give the air-stable compound **345**.

**Scheme 85:**
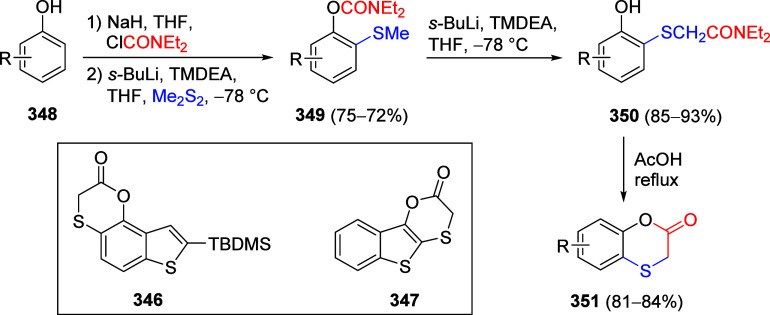
Synthesis of Fused Heterocycles with a [1,4]Oxathiin Ring (**346**, **347**) Using D*o*M, D*re*M-Rearrangement, and Cyclization Adapted from ref (364). Copyright 2005 The Japan Institute of Heterocyclic Chemistry.

The same authors also reported another class of fused heterocycles in which a [1,4]oxathiin ring is fused to an aromatic nucleus (**346**, **347**). This synthesis is another example of the versatility of the D*o*M method. The introduction of a methylsulfanyl group in the *ortho*-position to the *O*-carbamate function (**349**) is followed by lateral metalation of the introduced functionality and anionic rearrangement *in situ* (**350**). Acid-induced cyclization of the rearranged product completes the annulation process (**351**). Tricyclic compounds can be prepared with [1,4]oxathiin rings fused angularly (**346**) or linearly (**347**) to a benzo[*b*]tiophene core (Scheme 85).

**Scheme 86:**
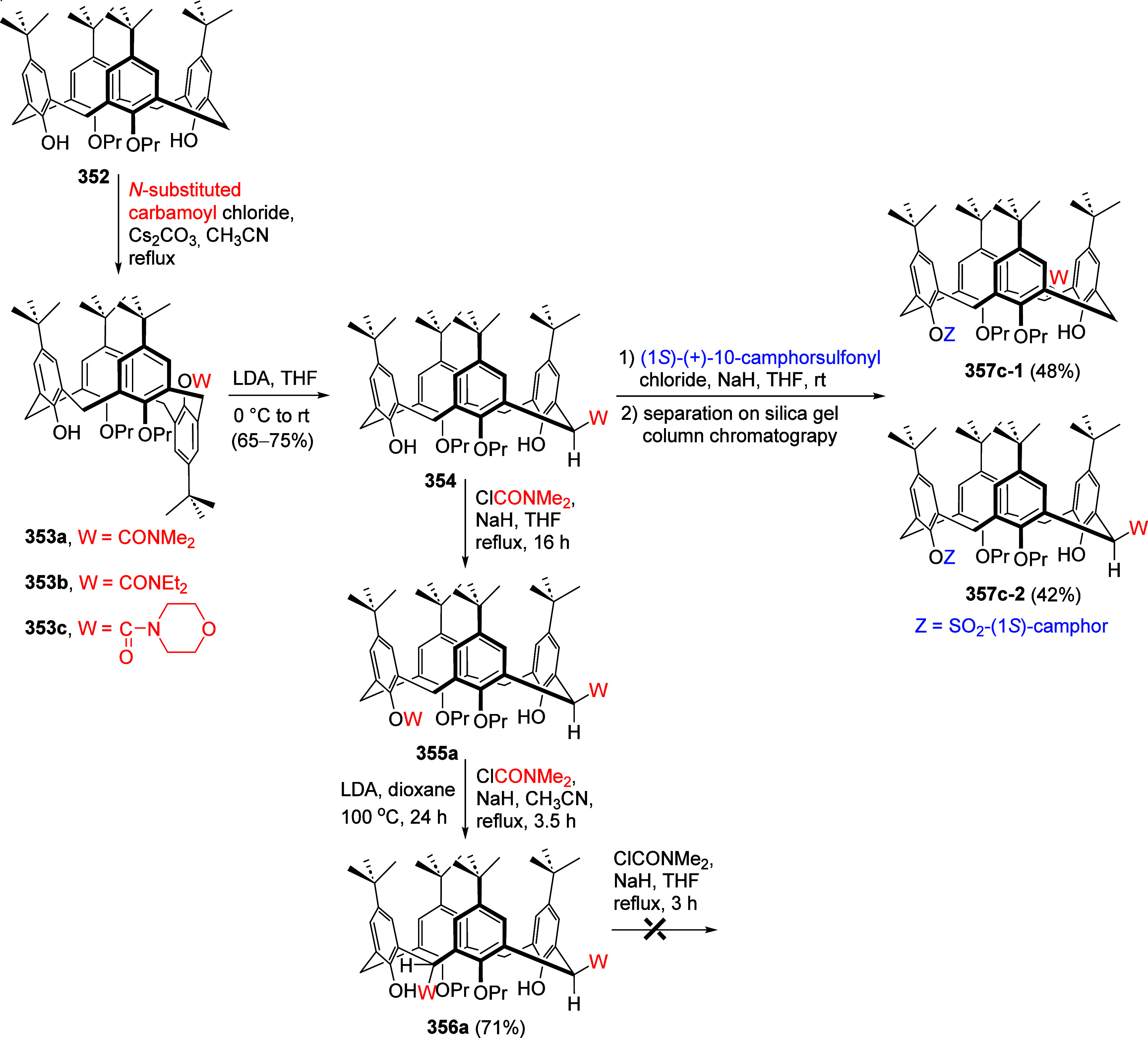
Chiral *p*-*tert*-Butylcalix[4]arenes (*p*-*t*-Bu-BCCs), **354a**–**c**, with Various *N*-Substituted Carbamoyl Bridge Substituents, and *p*-*t*-Bu-BCC with Two *N*,*N*-Dimethylcarbamoyl Bridge Substituents **356a**, Prepared by A*o*F Rearrangement, and Optical Resolution of **357c** into a Pair of Diastereomers **357c-1** and **357c-2** Adapted with permission from ref (365). Copyright 2020 John Wiley and Sons.

Ma et al.^365^ presented bridging chiral *p*-*tert*-butylcalix[4]arenes (*p*-*t*-Bu-BCC’s) with various *N*-substituted carbamoyl bridge-substituents (*N*,*N*-dimethylcarbamoyl, **354a**; *N*,*N*-diethylcarbamoyl, **354b**; morpholinocarbonyl, **354c**) by A*o*F rearrangement from mono-*O*-carbamates of 1,3-dipropyl-*p*-*tert*-butylcalix[4]arene **353** in 65–75% yield (Scheme 86). In addition, *p*-*t*-Bu-BCC with two *N*,*N*-dimethylcarbamoyl bridging substituents **356a** was also prepared by this method from mono-*O*-carbamate of *p*-*t*-Bu-BCC **355a** with one *N*,*N*-dimethylcarbamoyl bridging substituent in 71% yield. The racemic *p*-*t*-Bu-BCC with a morpholinocarbonyl bridging substituent **357c** was also optically resolved into a pair of diastereomers **357c-1** and **357c-2** in 90% overall yield (**357c-1**, 48%; **357c-2**, 42%).

**Scheme 87:**
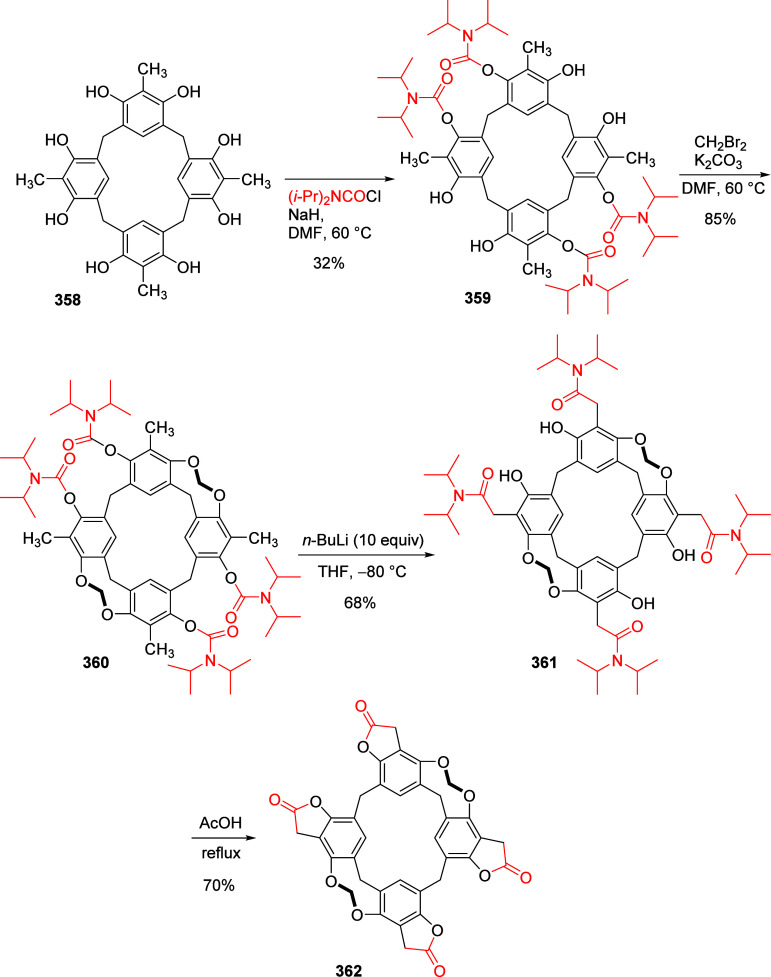
First Example of a Four-fold Homologous A*o*F Rearrangement of **360** to the Double Bridged Tetrakis(carboxamidomethyl)resorc[4]aren **361**, a Potentially Interesting Precursor for Supramolecular Chemistry (via **362**) Adapted with permission from ref (366) Copyright 2001 Canadian Science Publishing.

The Reinhoudt group applied the D*o*M concept for the introduction of upper rim functional groups via a 4-fold homologous A*o*F rearrangement in the doubly bridged tetra-*O*-carbamate **360** to prepare resorc[4]arene **361** in 68% yield (Scheme 87).^366^ Carbamate **360** was prepared by selective carbamoylation of methylresorc[4]arene **358**, followed by bridging of the remaining OH groups in tetra-*O*-carbamate **359**. Resorc[4]arene **361** could be further converted to **362**, an interesting building block for supramolecular chemistry.

**Scheme 88:**
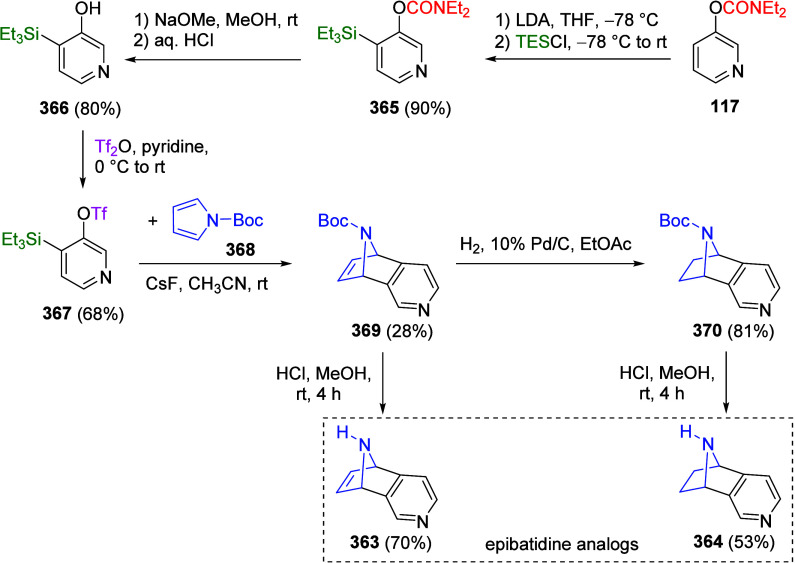
Synthesis of a Nicotinic Acetylcholine Receptor Epibatidine Analogues with Fused 3,4-Pyridine Ring (**363** and **364**) Adapted from ref (367). Copyright 2007 American Chemical Society.

Carroll et al.^367^ developed a method for the synthesis of epibatidine analogues with fused 3,4-pyridine ring (**363** and **364**), a nicotinic acetylcholine receptor, starting from readily available pyridin-3-yl-diethylcarbamate (**117**) (Scheme 88). D*o*M of **117**, followed by silylation to **365**, cleavage of the carbamate group and addition of trifluoromethanesulfonic anhydride leads to **367**, which reacts in a crucial step with *tert*-butyl-1*H*-pyrrole-1-carboxylate (**368**) to form **369**. Catalytic hydrogenation of **369** gave **370**. Treatment of **369** and **370** with hydrogen chloride in methanol gave the desired epibatidine analogues **363** and **364** in an overall yield of 6–10% each.

**Scheme 89:**
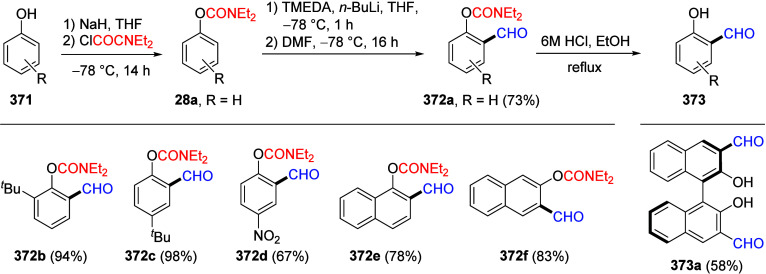
An Efficient Route to the Preparation of Useful *ortho*-Hydroxybenzaldehydes **373** via *ortho*-Lithiation of *O*-Carbamates, Exemplified by Chiral Binaphthol **373a** Adapted with permission from ref (368). Copyright 2008 CAOD (China/Asia on Demand).

Hao et al.^368^ presented another interesting *ortho*-lithiation of phenyl *O*-carbamates starting from phenols (**371**) treated with DMF (to form **372**) and hydrolyzed with HCl in the final step to demonstrate a general route for the regioselective preparation of various *ortho*-hydroxybenzaldehydes **373** in moderate to excellent yields (Scheme 89). The chiral binaphthol carbaldehyde **373a** is an example of a useful building block.

**Scheme 90:**
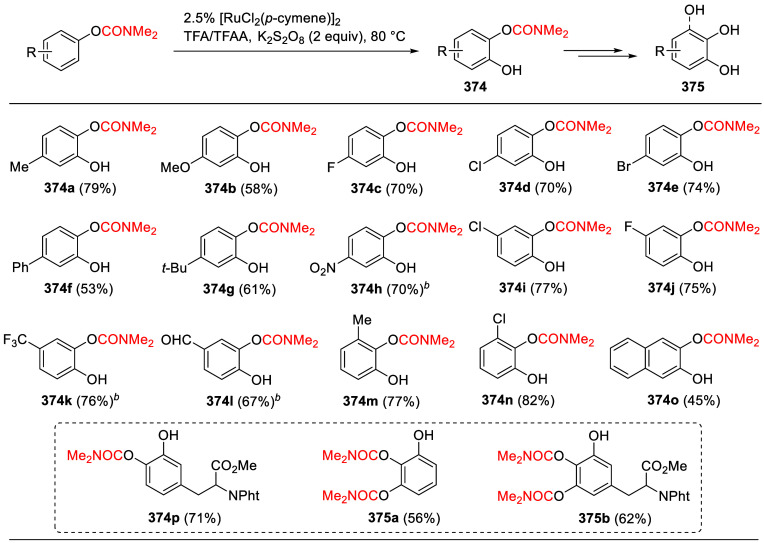
A New Approach for the Synthesis of Catechol (**374**) and Pyrogallol (**375**) Derivatives by Ru-Catalyzed C–H Hydroxylation *^a^*Adapted with permission from ref (369). Copyright 2014 John Wiley and Sons. *^b^* PhI(OAc)_2_ (1.5 equiv) as oxidant instead of K_2_S_2_O_8_.

An efficient ruthenium(II)-catalyzed C–H hydroxylation of ArOAms was developed for the facile synthesis of catechol (**374**) and pyrogallol derivatives (**375**) from readily accessible phenols (Scheme 90).^369^ This method has excellent regio- and chemoselectivity, good functional group tolerance and high yield, and offers a new route for the construction of some biologically important molecules. For example, l-DOPA can be easily obtained from the catechol derivative **374p**, which is synthesized from protected l-tyrosine. In addition, catechols can be further converted into synthetically sophisticated, valuable pyrogallol derivatives (**375a**,**b**).

**Scheme 91:**
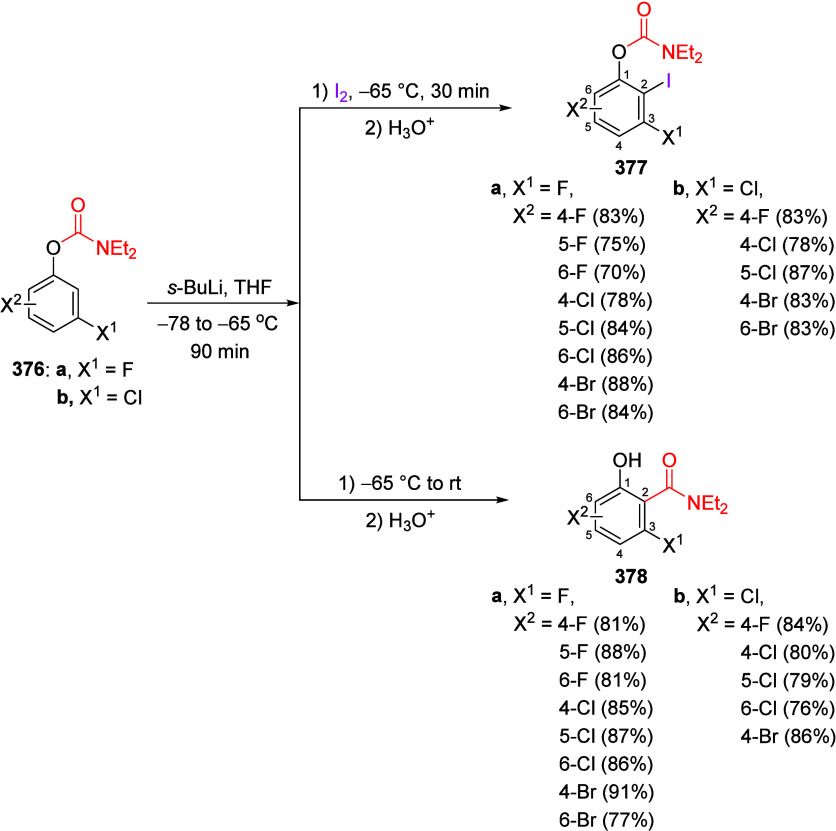
A Regioselective D*o*M of *meta*-Halogenated Carbamates **376** to Obtain Either Trihalophenol Derivatives **377** or Further A*o*F Rearranged Dihalosalicylamides **378** Adapted with permission from ref (370). Copyright 2016 John Wiley and Sons.

Finally, Feberero et al.^370^ found that the regioselective D*o*M of *O*-(3-halophenyl)-*N*,*N*-diethylcarbamates **376** can be extended to a wide range of related carbamates with an additional halogen substituent (Scheme 91). The cooperative effect of the halide in *meta*-position to the carbamate group influences the reaction outcome. The organolithium intermediate can either be trapped with iodine at low temperature to give a variety of trihalophenol derivatives **377** in high yield (70–88%), or A*o*F rearrangement of the organolithium intermediate at room temperature leads to a broad family of new dihalosalicylamides **378** in high yield (76–91%).

### 9.2 D*o*M Followed by Annulation/Cyclization

**Scheme 92:**
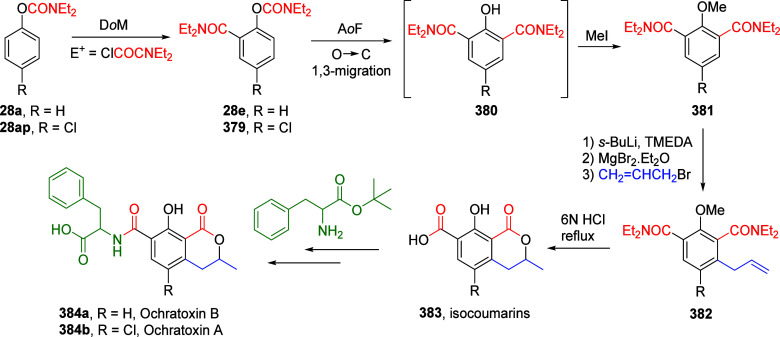
Synthetic Scheme to Ochratoxin A and B (**384**) via Isocoumarins (**383**) Which Can Be Prepared from ArOAm (**28a**, **28ap**) Using a Mix of D*o*M and A*o*F Chemistry (First Two Steps) Adapted from ref (371). Copyright 1984 American Chemical Society. Adapted from ref (372). Copyright 1985 American Chemical Society.

The earliest preparation of PAH compounds using ArOAm was reported by Sibi et al. in 1984^371^ and in 1985.^372^ The work focused on the synthesis of isocoumarins (**383**), precursors for the toxic fungal metabolites ochratoxin B (**384a**) and ochratoxin A (**384b**), respectively (Scheme 92).

The first steps were dependent on the ArOAm group for installation of the amide group followed by A*o*F to afford **380**, which could be methylated to **381**. Subsequent synthesis of isocoumarins **383a** and **383b** involved D*o*M/transmetalation chemistry followed by allylation and hydrolysis. The final step, as described by Bouisseau et al.,^373^ involves the coupling of **383** with l-phenylalanine-*tert*-butyl ester using oxalyl chloride followed by deprotection of the acid using TFA.

**Scheme 93:**
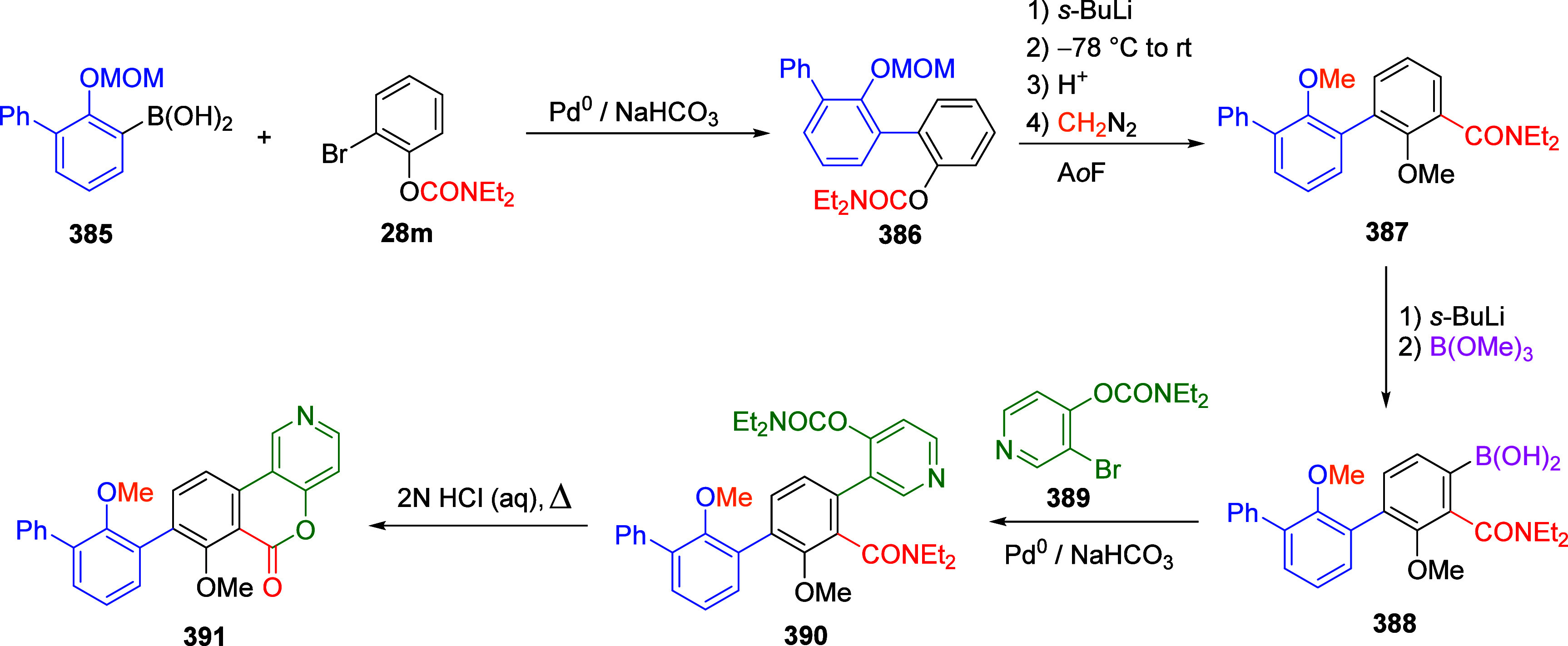
Synthesis of Chromenopyridinone **391** via a Metalation/A*o*F/Suzuki Coupling/Lactonization Adapted with permission from ref (374). Copyright 1987 Elsevier.

Two years later, a metalation/A*o*F/Suzuki coupling/lactonization was reported by Cheng and Snieckus in 1987^374^ for the synthesis of chromenopyridinone **391** (Scheme 93). The synthetic protocol commenced with synthesis of *m*-terphenyl **386** via Pd-catalyzed coupling of **385** and **28m**, exploiting the inertness of OAm to Pd catalysis. Compound **386** was then metalated and allowed to warm to room temperature before the crude product was hydrolyzed with aq HCl and methylated with diazomethane (CH_2_N_2_) to afford amide **387**. Using the amide functionality as a DMG, **387** was transformed into aryl boronic acid **388**, which, when coupled with **389**, provided the pyridotetraphenyl **390**.

Compound **390** was then converted to the chromenopyridinone **391** via a lactonization process. This scheme shows the old-fashioned approach for the cyclization of phenol to amide. In this case, the *O*-carbamate is basically just a protected phenol (spectator) and is hydrolyzed under rather harsh conditions. The diethylamide on the ring is probably also forced to lactonize under these conditions by the initial hydrolysis.

**Scheme 94:**
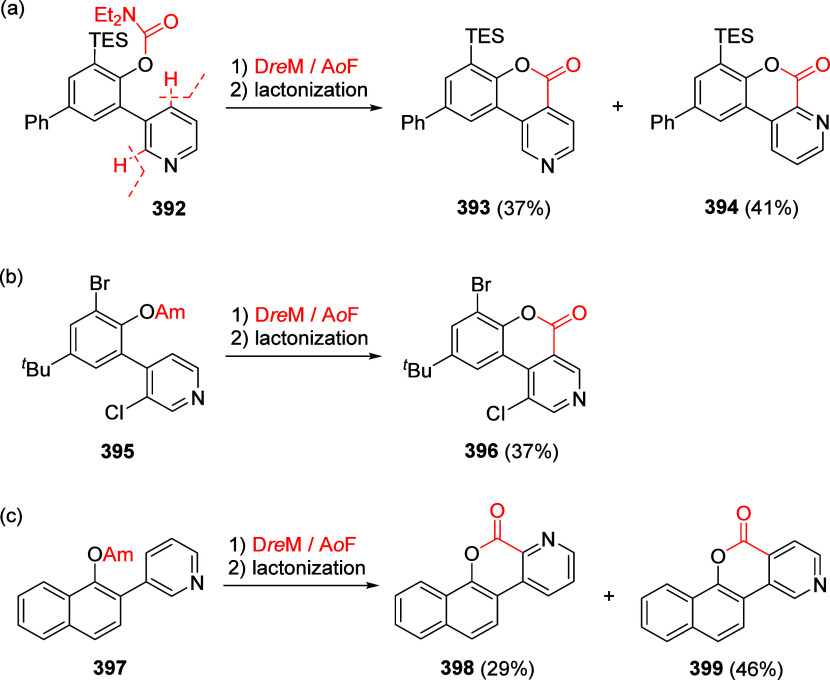
(a) D*re*M/A*o*F/Lactonization of **392** to Form Isomeric Chromenopyridinones **393** and **394**; (b) Regiospecific D*re*M Facilitated by *meta*-Substituted Pyridine to Form **396**; (c) Synthesis of Isomeric Benzochromenopyridinones **398** and **399** by DreM/A*o*F/Lactonization of Naphthalenylpyridine **397** Adapted from ref (375). Copyright 2012 The Japan Institute of Heterocyclic Chemistry.

Similar compounds (**393**, **394**, **396**, **398**, and **399**) have been prepared by Snieckus et al.^375^ using D*re*M/A*o*F rearrangement/lactonization sequence (examples shown in Scheme 94a–c).

**Scheme 95:**
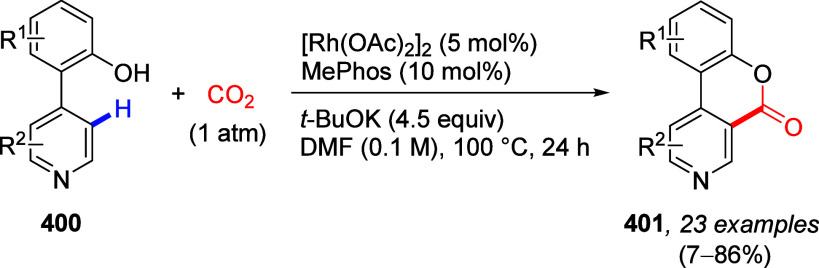
Rhodium-Catalyzed Carboxylation of Aryl and Alkenyl C–H Bonds with CO_2_ Adapted with permission from ref (376). Copyright 2018 John Wiley and Sons.

In comparison, an alternative synthetic approach to the same class of compounds (**401**) was developed by Li’s research group, involving the rhodium(II)-catalyzed aryl C–H carboxylation of 2-pyridylphenols (**400**) with CO_2_ (Scheme 95).^376,377^

Advantages of this approach include the use of CO_2_,^378^ compatibility with challenging electron-deficient pyridine heterocycles, and avoidance of the formation of isomeric compounds.

**Scheme 96:**
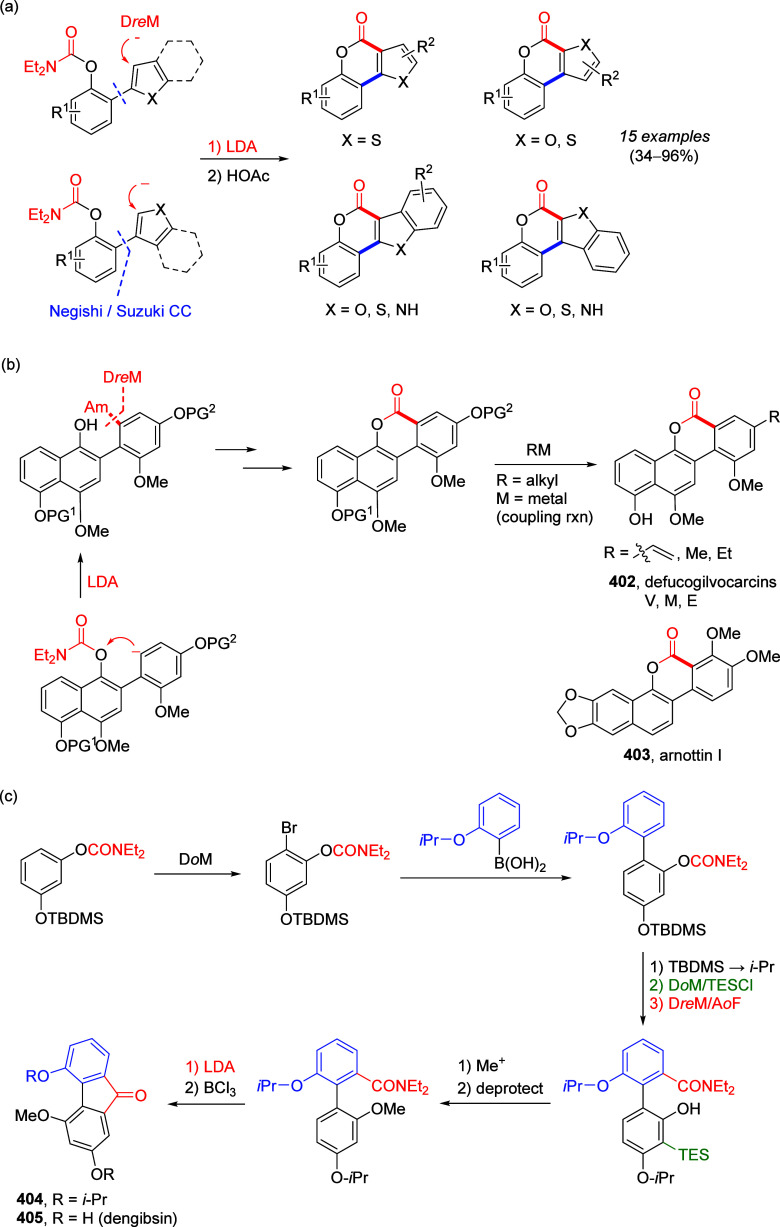
D*re*M/CC Sequence Used (a) in the Synthesis of Heteroaryl-Fused Benzopyranones (Adapted from ref (295), Copyright 2009 American Chemical Society) and (b) in the Synthesis of the Aglycones of the Gilvocarcins (V, M, and E), **402**, and Arnottin I, **403** (Adapted from ref (379), Copyright 2009 American Chemical Society); (c) The D*o*M/CC/D*re*M Sequence Used in the Synthesis of Dibenzo[*b*,*d*]pyranones, **404**, and the Fluorenone Dengibsin, **405** (Adapted from ref (19), Copyright 2009 American Chemical Society)

James and Snieckus extended D*re*M/A*o*F rearrangement/lactonization sequence to the synthesis of heteroaryl-fused benzopyranones^295^Scheme 96a) and the aglycones of the gilvocarcins (V, M, and E), **402**, and arnottin I (**403**)^379^ (Scheme 96b).

A recently (2017) developed method for preparing the same compounds uses an *N*-heterocyclic carbene (NHC)-catalyzed reaction between enals and furanones,^380^ which is a greener, metal-free option.^381,382^

Snieckus’ research group applied a D*o*M/Suzuki CC/remote A*o*F rearrangement/cyclization to produce dibenzo[*b*,*d*]pyranones (e.g., **404**), and dengibsin (**405**), a naturally occurring fluorenone (from orchids)^19,383^ (Scheme 96c). The synthesis of dengibsin was achieved in 15 steps (some steps omitted for clarity in Scheme 96c) by D*re*M. Since the publication of this synthesis in 1992, several synthesis protocols have been published for both dibenzo[*b*,*d*]pyranones and fluorenones. For example, dibenzo[*b*,*d*]pyranones have been prepared via a multicomponent (substituted salicylaldehyde, dimethyl glutaconate, cyclopentanone, base, solvent) domino reaction,^384^ a [3 + 3] cyclization of a 1,3-bis(silyl enol ether) with a 3-silyloxy-2-en-1-one,^385^ a [3 + 3] cyclization–Suzuki CC method,^386^ Pd-catalyzed cyclization of an enediyne,^387^ Rh(III)-catalyzed coupling of aryl ketone *O*-acetyl oximes and quinones,^388^ an “inverse electron demand Diels–Alder reaction,”^389^ and a one-pot Sonogashira coupling-benzannulation of aryl 3-bromopropenoates.^390^

Similarly, the synthesis of fluorenones has been reported using different approaches, nicely summarized by Jourjine et al.^391^ A gram-scale total synthesis of dengibsin was reported by Jones and Ciske in 1996.^392^

**Scheme 97:**
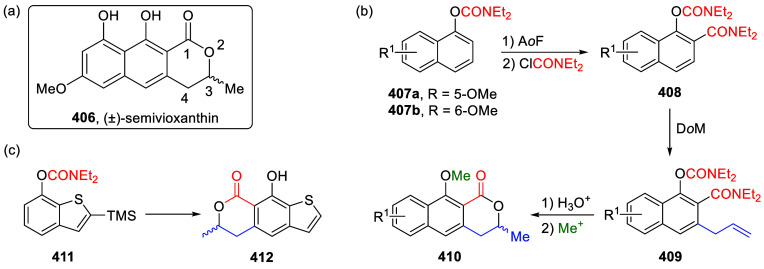
(a) A Naturally Occurring Linear Naphthopyrone (±)-Semivioxanthin (**406**); (b) The A*o*F/D*o*M Sequence Followed by Annelation Used for the Synthesis of (±)-Semivioxanthin Analogues **410**; (c) An Example of the A*o*F/D*o*M/Lactonization Sequence Used for the Synthesis of **412** Adapted with permission from ref (393). Copyright 2003 Elsevier.

Kamila et al.^393^ reported the synthesis of several analogues of (±)-semivioxanthin, a naturally occurring linear naphthopyrone with a stereogenic center at C-3 (**406**) (Scheme 97a), using an aryl-*O*-carbamate-mediated D*o*M to prepare functionalized naphthalene substrates (**408** and **409**) prior to annealing the pyrone (Scheme 97b). Critical to the success of the reaction sequence was the introduction of an allyl group *ortho*- to the amide function in **408** (using allyl bromide) and the cyclization of the resulting compound (**409**) to give the final product **410**. The harsh reaction conditions required for cyclization (heating under reflux with 6 N HCl for 36 h) resulted in hydrolysis of the *O*-carbamate. The somewhat labile phenolic compound formed was immediately *O*-methylated to afford the (±)-semivioxanthin analogues (**410**).

**Scheme 98:**
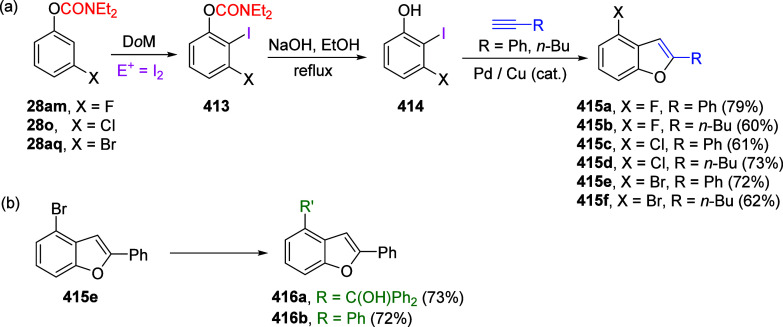
(a) The Synthesis of 2,3-Dihalophenols, **414**, Using D*o*M Protocol and Further Preparation of 4-Halobenzo[*b*]furans, **415**; (b) An Example of Further Transformation of 4-Halobenzo[*b*]furans Adapted from ref (214). Copyright 2005 American Chemical Society.

Sanz et al.^214^ used ArOAm to prepare selected 2,3-dihalophenols, which could then be used to form 4-functionalized benzo[*b*]furans. The synthesis commenced with the halogen substituted ArOAm compounds **28o**, **28am**, and **28aq**. After *O*-carbamate-mediated D*o*M (electrophilic quenching with I_2_) and subsequent hydrolysis of the *O*-carbamate group, **28o**, **28am**, and **28aq** gave the halophenols **414a**–**c**, which are otherwise only available from specialized suppliers at exorbitant prices (e.g., 2-fluoro-3-iodophenol (CAS no. 863870-85-3) costs USD$ 750/1 g from Matrix Scientific, May 2024) (Scheme 98a).

**Scheme 99:**
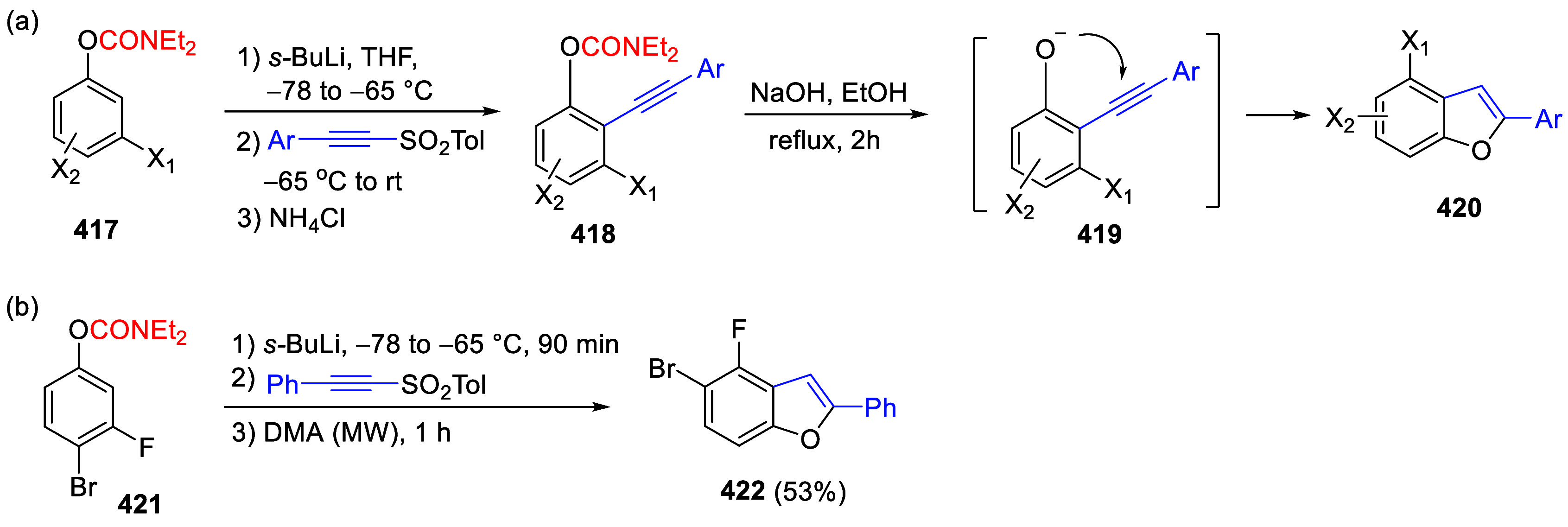
(a) Synthesis of Halobenzo[*b*]furans, **420**, Using an Improved D*o*M/Hydrolysis/Cyclization Sequence; (b) An Example of One-Pot Synthesis of the Halobenzo[*b*]furan, **422** Adapted from ref (200). Copyright 2022 MDPI.

Subsequent reaction of the 2-iodo-3-halophenol derivatives **414** with two different terminal alkynes (phenylacetylene and 1-hexyne) via a tandem Sonogashira coupling/5-*endo-dig* cyclization (catalyzed by [Pd(OAc)_2_(PPh_3_)_2_]/CuI) gave the expected 4-halobenzo[*b*]furans **415** in moderate (60%) to good (79%) yields (Scheme 98a). Substituted 4-halobenzo[*b*]furans could then be modified by other transformations, e.g., lithiation (quenching with benzophenone to form **416a**) or Suzuki coupling with phenylboronic acid under Pd catalysis (**416b**) (Scheme 98b). This work was later improved to extend the scope of regioselective lithiation of the *O*-dihalophenyl-*N*,*N*-diethylcarbamates to exclude the use of transition metals (Pd and Cu) and the need for an *ortho*-iodine (consumed in the Sonogashira coupling process) in the cyclization step.^200^ The *O*-carbamate (**417**) was still crucial, this time to access *O*-*o*-alkynylaryl *N*,*N*-diethylcarbamates (**418**) (by using arylsulfonylacetylenes as alkynylation reagents for the lithiated compound), which allowed the synthesis of halobenzo[*b*]furans by basic hydrolysis–cyclization of **419**, bypassing the Sonogashira step (Scheme 99a). Finally, the authors further developed this process into a one-pot procedure with microwave heating, as shown by the synthesis of halobenzo[*b*]furan **422** (Scheme 99b).

**Scheme 100:**
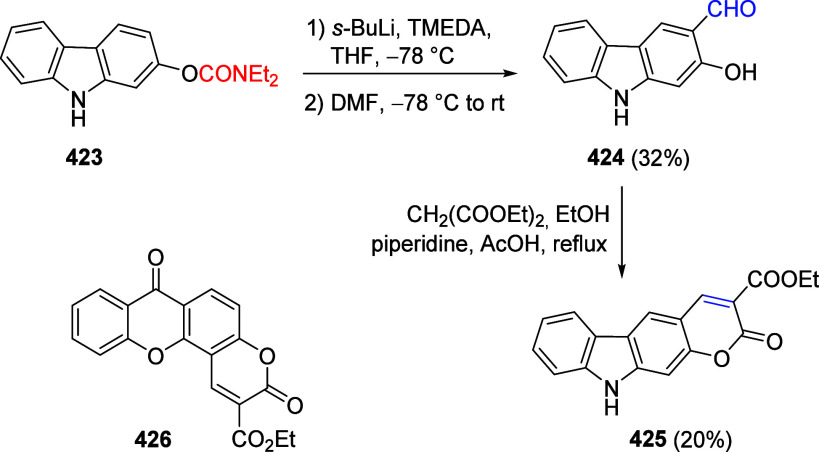
Synthesis of a Tetracyclic Benzopsoralen Analogue, **425**, with a 9*H*-Carbazole Framework, Commencing with *O*-Carbamate **423** Adapted with permission from ref (394). Copyright 2007 John Wiley and Sons.

Oliveira et al.^394^ used *O*-carbamate to form a tetracyclic benzopsoralen analogue (**425**), which was also tested for antitumor activity (Scheme 100). In this case, 9*H*-carbazol-2-yl diethylcarbamate (**423**) was converted to the aldehyde **424** by metalation, followed by treatment with DMF. Compound **424** was then cyclized with diethyl malonate to form **425** in 20% yield. Unfortunately, even at high concentrations (150 μm), the congener **425** proved to be ineffective as a growth inhibitor compared to 9*H*-xanthen-9-ones such as **426** (due to the difference in geometry, i.e., angular versus linear and functional groups present).

**Scheme 101:**
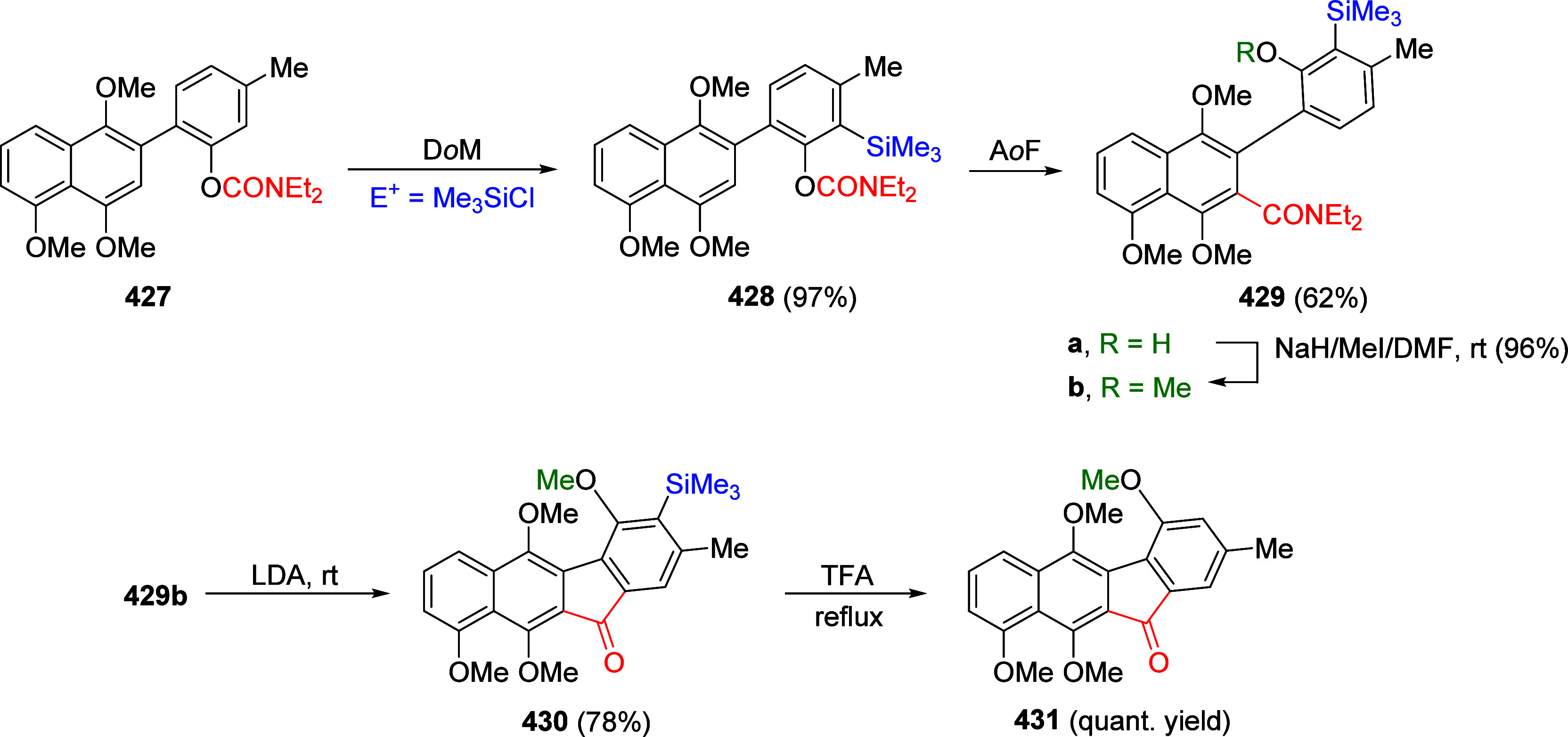
Synthesis of Benzo[*b*]fluorenone, **431**, via a Five-Step Method Adapted from ref (395). Copyright 1997 American Chemical Society.

Mohri et al.^395^ developed a five-step synthesis of benzo[*b*]fluorenone (**431**) based on the use of *O*-carbamate as DMG for D*o*M installation of the “blocker” TMS (to form **428**), followed by facilitation of a crucial O→C ring-to-ring carbamoyl transfer (with excess LDA) to provide **429a** in 62% yield (Scheme 101).

Phenol **429a** was methylated to give biarylamide **429b**, which was subjected to a second remote metalation/cyclization to give fluorenone **430** (78% yield). Finally, **430** was treated with TFA to give benzofluorenone **431** in quantitative yield. Compared to other routes to this material, the combined sequence of D*o*M and CC has the advantage of providing direct access to important and diverse intermediates of the antibiotic kinamycin.

**Scheme 102:**
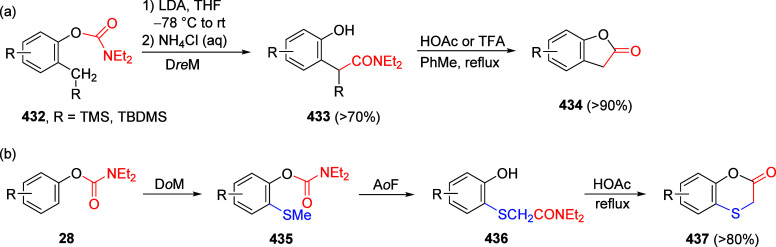
(a) Synthesis of Benzofuranones, **434**, Using LDA-Induced O→C-Carbamoyl Migration/Cyclization Sequence; (b) An Introduction of the Methyl Sulfanyl Function in the A*o*F–Cyclization Sequence Gives Condensed Oxathiin-2-ones **437** Adapted with permission from ref (397). Copyright 2003 Thieme.

Kalinin et al.^396^ discovered a new LDA-induced O→C-carbamoyl migration that provides easy access to phenylacetamides **433** and thus substituted benzo- and naphthofuranones **434** (Scheme 102a), extending the utility of D*o*M. Introduction of the methylsulfanyl function in *ortho-* position to the *O*-carbamate functionality and deprotonation of the side chain with *s*-BuLi at −78 °C afforded the rearranged products, *N*,*N*-diethyl-2-hydroxyarylthioacetamides **436** (Scheme 102b). Their heating with glacial acetic acid under reflux led to the condensed oxathiin-2-ones **437** in excellent yields.^397^

**Scheme 103:**
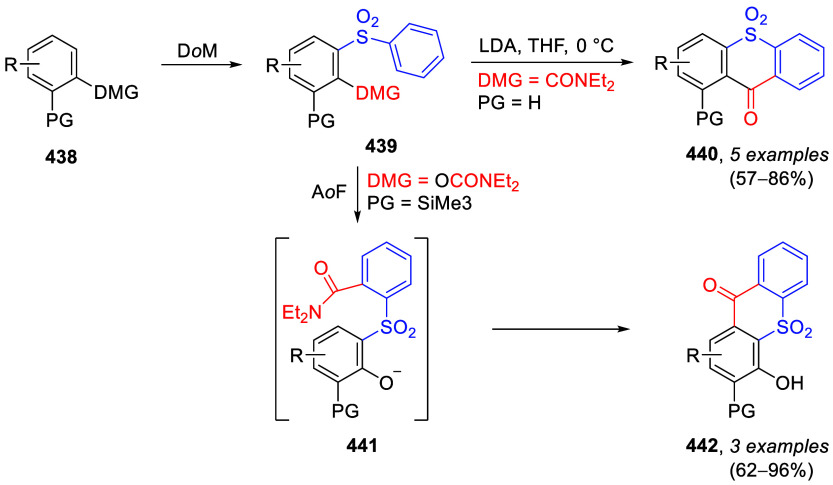
Synthesis of Thioxanthen-9-one 10,10-Dioxides, **440** and **442** via LDA-Mediated Reactions of 2-Carboxamido- and 2-*O*-Carbamoyl Diaryl Sulfones **439** Using Friedel–Crafts and Sequential Remote Fries Rearrangement and Friedel–Crafts Reaction Adapted from ref (398). Copyright 1994 American Chemical Society.

Beaulieu and Snieckus^398^ extended their work on heteroatom-bridged biaryl metalation by LDA-mediated reactions of 2-carboxamido- and 2-*O*-carbamoyl diaryl sulfones **439**, leading to thioxanthen-9-one 10,10-dioxides, **440** and **442**, respectively (Scheme 103). These formal anionic equivalents of the Friedel–Crafts reaction and the sequential remote Fries rearrangement and Friedel–Crafts reaction (via **441**) allow the construction of fused heterocycles from heteroatom-bridged biaryl systems that replace or effectively compete with the classical routes.

**Scheme 104:**
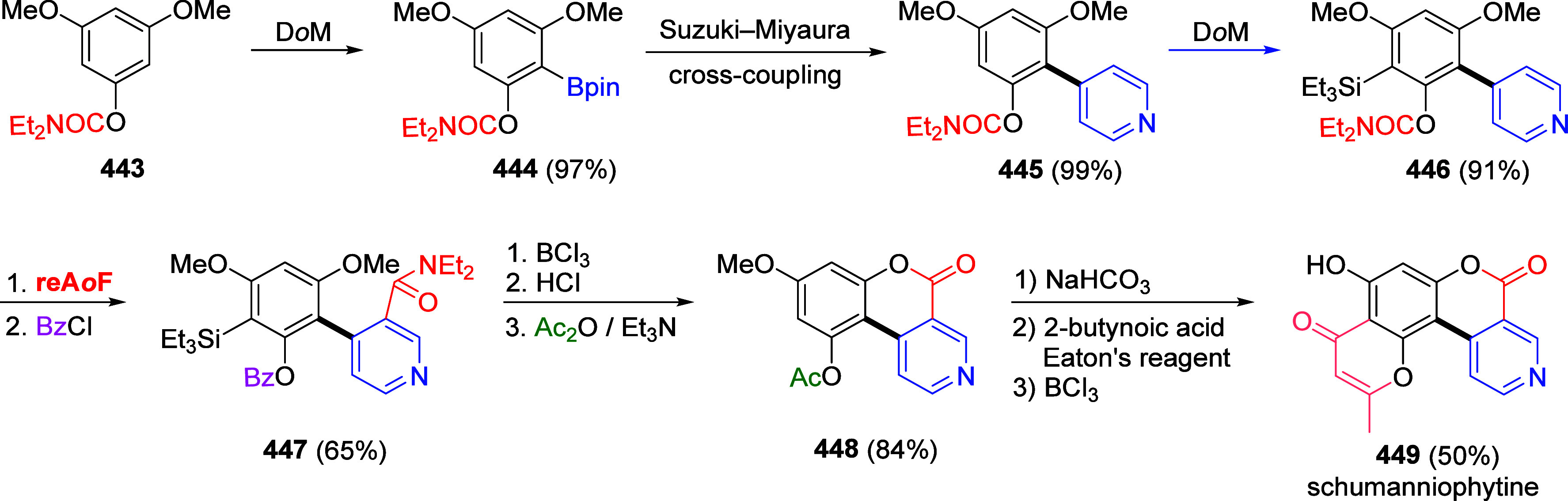
Synthesis of the Alkaloid Schumanniophytine (**449**) Starting from a Simple Building Block (**443**) and Involving D*o*M, Suzuki–Miyaura CC, and an *ortho*-Silicon Induced *O*-Carbamate-A*o*F Rearrangement Adapted with permission from ref (399). Copyright John Wiley and Sons.

Macklin et al.^399^ developed a short synthesis of the alkaloid schumanniophytine (**449**) starting with the metalation of symmetric *O*-carbamate **443** and, after boronation, yields the intermediate **444** in excellent yield (Scheme 104). Further Suzuki–Miyaura CC with commercial 4-bromopyridine hydrochloride led to azabiaryl **445**, followed by a regioselective second D*o*M reaction and silylation with TESCl to give highly hindered derivative **446**. With silicon protection present, remote A*o*F rearrangement led to a smooth carbamoyl translocation to the pyridine ring to give arylnicotinamide **447** after direct benzoylation. Regioselective demethylation with BCl_3_ gave the lactone **448** in high overall yield after acidic protodesilylation, debenzylation, and reacylation. Release of the free phenol from **448** with sodium bicarbonate, followed by adaptation of the versatile Eaton’s reagent and efficient demethylation under BCl_3_ conditions, gave schumanniophytine (**449**).

**Scheme 105:**
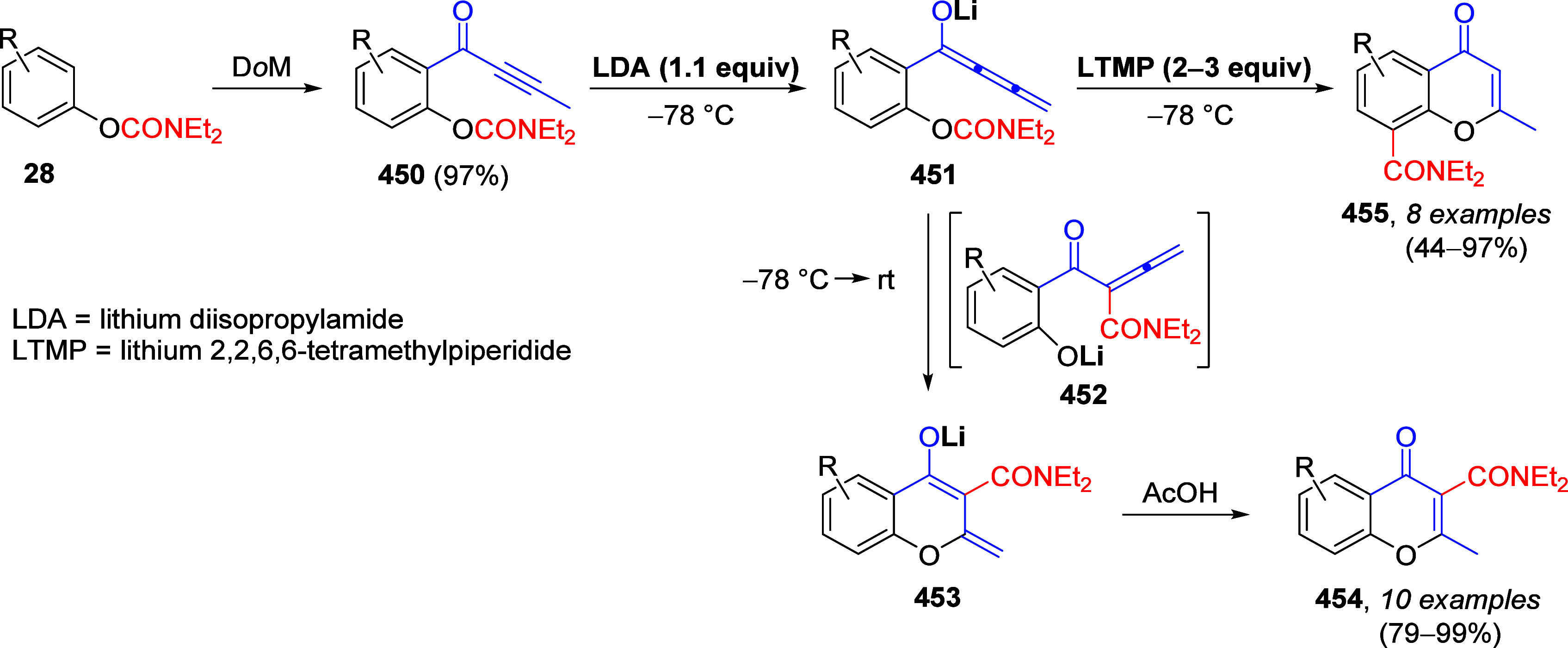
Regioselective Synthesis of Polysubstituted Chromone 3- and 8-Carboxamides by Anionic Carbamoyl Translocation Reactions with Sequential D*o*M, Intramolecular A*o*F, and Michael Addition via the Cumulenolate Intermediate **451** Additional amounts of base (LTMP) efficiently convert **450** to **455** via an A*o*F and Michael addition sequence. Adapted with permission from ref (23). Copyright 2008 John Wiley and Sons.

Based on initial model studies of schumanniophytine synthesis, Macklin et al.^23^ developed a new general and regioselective synthetic method for polysubstituted chromone 3- and 8-carboxamides, by anionic carbamoyl translocation reactions (Scheme 105). The reactions involve sequential intramolecular A*o*F rearrangement and Michael addition, which mechanistic studies indicate proceed via an intriguing coumulenolate intermediate **451** and open pathways to chromones that exhibit both unusual and elusive C8 substitutions and common and biologically significant 3-substitution patterns. When the cumulenolate **451** is heated to room temperature to promote carbamoyl transfer, a deep-red solution appears, indicating the formation of the lithium dienolate **453**. The color quickly disappears upon treatment with AcOH to give **454** in high yield. This result indicates a reaction pathway involving buta-2,3-dienamide, **452**, followed by intramolecular Michael addition of the resulting phenolate and subsequent protonation to give the chromone product **454**. The need for additional base amounts for effective conversion of **450** to **455** suggests that this reaction may also involve the cumulenolate **451**, which undergoes an A*o*F rearrangement followed by protonation and Michael addition.

**Scheme 106:**
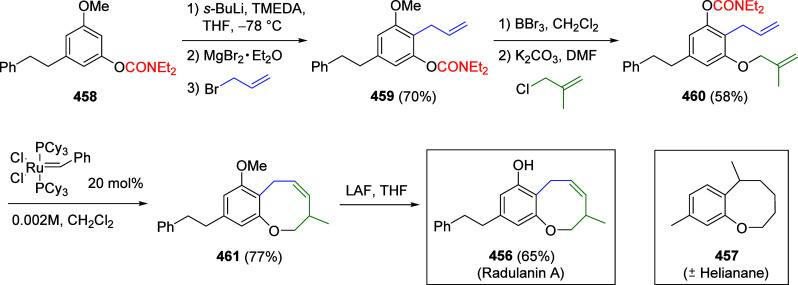
Synthesis of Benzene-Fused Oxygen Heterocycles with Multisized Rings by Combining D*o*M and Olefin Ring Closing Metathesis to Afford the First Synthesis of Radulanin A (**456**) and (±)-Helianane (**457**) Adapted from ref (400). Copyright 1998 American Chemical Society.

Stefinovic at al.^400^ prepared multisized benzannulated oxygen heterocycles by a combination of D*o*M and ring-closing metathesis (RCM) (Scheme 106). This first report of such RCM procedure for various ring sizes, demonstrating the synthetic link between RCM and regiospecific D*o*M strategy, finds its effective expression in the first total synthesis of the calmodulin inhibitor radulanin A (**456**) and helianane (**457**). In the synthesis of **456**, the readily prepared **458** was subjected to a D*o*M/transmetalation/C-allylation sequence to give **459**, and further *O*-allylation (**460**) and RCM reaction gave benzoxepine **461**. Reduction with LAH completed the synthesis of radulanin A (**456**) in 11 steps and 14% overall yield. Helianane (**457**), a racemic natural product, was synthesized in a very similar reaction sequence in nine steps with an overall yield of 9%.

**Scheme 107:**
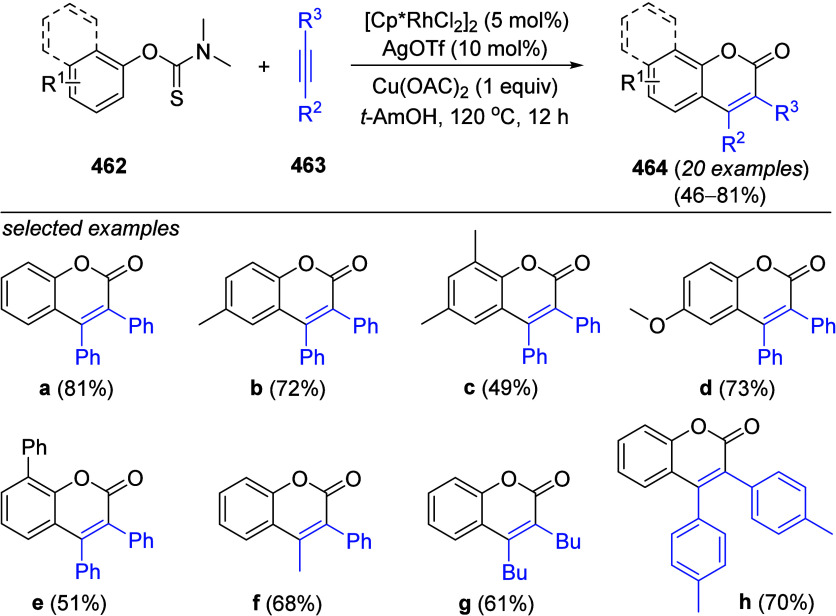
Synthesis of 3,4-Disubstituted Coumarins (**464**) by Combining D*o*M and Rh-Catalyzed Annulation of Aryl Thiocarbamates (**462**) with Alkynes (**463**) Adapted from ref (401). Copyright 2015 American Chemical Society.

Zhao et al.^401^ developed a Rh-catalyzed annulation of aryl thiocarbamates (**462**) with alkynes (**463**) via D*o*M C–H bond activation and a desulfurization to construct 3,4-disubstituted coumarins (**464**) in moderate yields (46–81%) (Scheme 107).

### 9.3 Combined D*o*M-CC Reactions

**Scheme 108:**
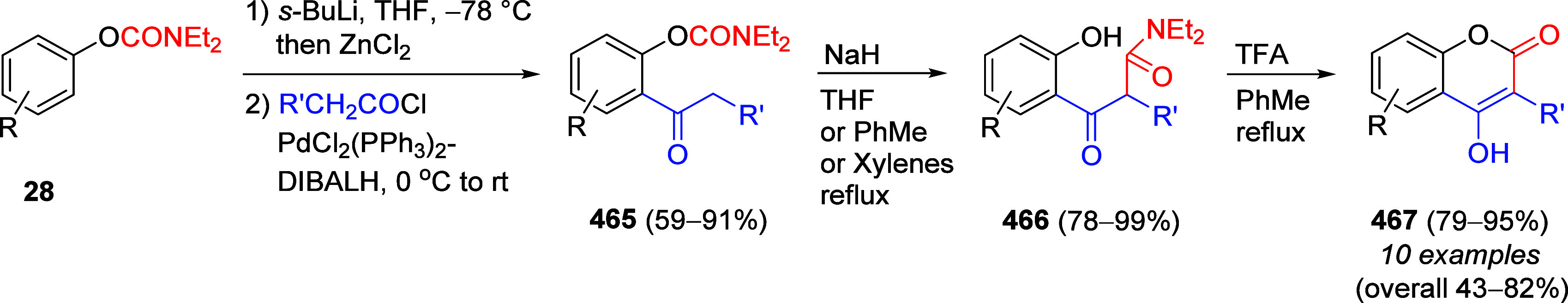
General Synthesis of Substituted 4-Hydroxycoumarins **467** Using a D*o*M/Negishi CC Protocol and a Carbamoyl–Baker–Venkataraman Rearrangement As the Key Step Adapted with permission from ref (328). Copyright 1998 Elsevier.

Kalinin et al.^328^ reported a general synthesis of substituted 4-hydroxycoumarins **467** in 43–82% overall yield by carbamoyl–Baker–Venkataraman rearrangement. Intermediate *ortho*-acylated ArOAms **465** were prepared from ArOAms **28** via a D*o*M/Negishi CC protocol (59–91% yield), and the overall sequence provides a regiospecific anionic Friedel–Crafts complement for the assembly of 2-hydroxyarylacetamides **466** (78–99% yield) and final coumarins **467** in high yield (79–95%) (Scheme 108).

**Scheme 109:**
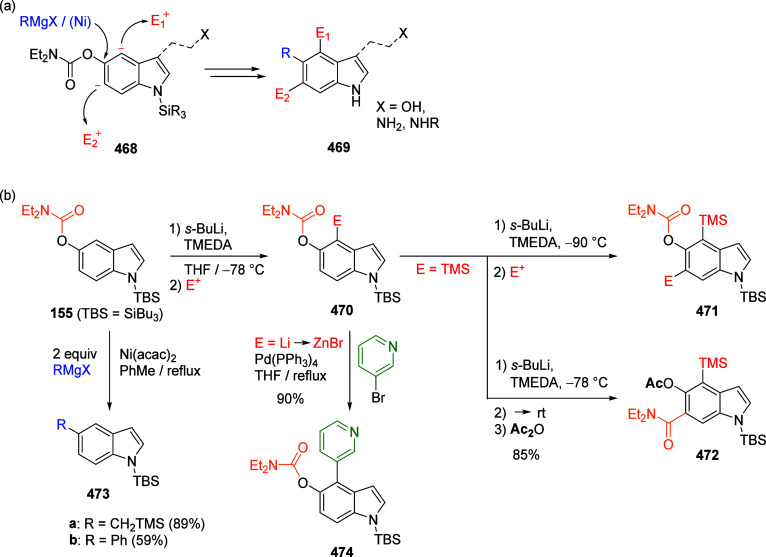
(a) General Strategies for the Regiospecific Construction of 4-, 5-, and 6-Substituted Indoles and Tryptophols **469** Using D*o*M and Transition Metal-Catalyzed CC Reactions on *N*,*N*-Diethylindole-5-*O*-carbamate, **468**; (b) Examples of Selective Regiospecific Construction of Indoles **471**–**474** Adapted with permission from ref (213). Copyright 2022 John Wiley and Sons.

The same research group^213^ demonstrated the regiospecific construction of 4-, 5-, and 6-substituted indoles and tryptophols, **469**, by adapting D*o*M and transition metal-catalyzed CC reactions to *N*,*N*-diethylindole-5-*O*-carbamate, **468** (Scheme 109a). Selective 4- and 6-substitutions can be achieved via TMS protection and A*o*F tactics (**470**–**472**) with moderate yields (50–99%). In addition, they have adapted the above organozinc and Grignard transition metal catalyzed CC reactions to develop powerful new protocols for C–C bond formation (**473a**,**b**, **474**) with excellent yields (≈ 90%) (Scheme 109b). The authors also demonstrated the preliminary chemistry of tryptophol metalation (**469**, X = OH), anticipating a broader perspective.

**Scheme 110:**
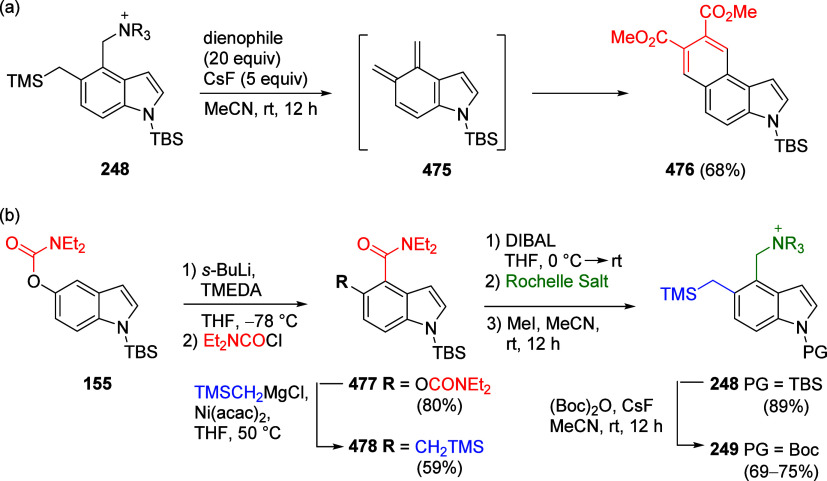
(a) Preparation of the Indolo-4,5-quinodimethane Intermediate (**475**) from **248** and Its Conversion with Various Dienophiles to New Ring Systems, Such as Benz[*e*]indole, **476**; (b) Preparation of **248**–**249** by *O*-Carbamate-D*o*M/CC Tactics Adapted with permission from ref (313). Copyright 1999 Elsevier.

Kinsman and Snieckus^313^ reported the first preparation of indolo-4,5-quinodimethane **475** as an intermediate over the *ortho*-quinodimethane precursor **248** by *O*-carbamate-D*o*M/Kumada–Corriu CC tactics and its conversion with various dienophiles to new ring systems, such as benz[*e*]indole **476** (Scheme 110a), in modest and excellent yields (40–95%). The preparation of the *ortho*-quinodimethane precursor **248** began with a highly regioselective metalation-carboxamidation of *O*-carbamate **155** to give **477** in 80% yield, leading to benzylsilane **478** (59%) after Grignard transition metal-catalyzed CC. DIBAL reduction followed by treatment with MeI gave the quaternary salt **248** in excellent yield (89%), which was treated with CsF and (Boc)_2_O to give the *N*-Boc derivative **249** in a convenient and mild one-pot procedure (Scheme 110b).

**Scheme 111:**
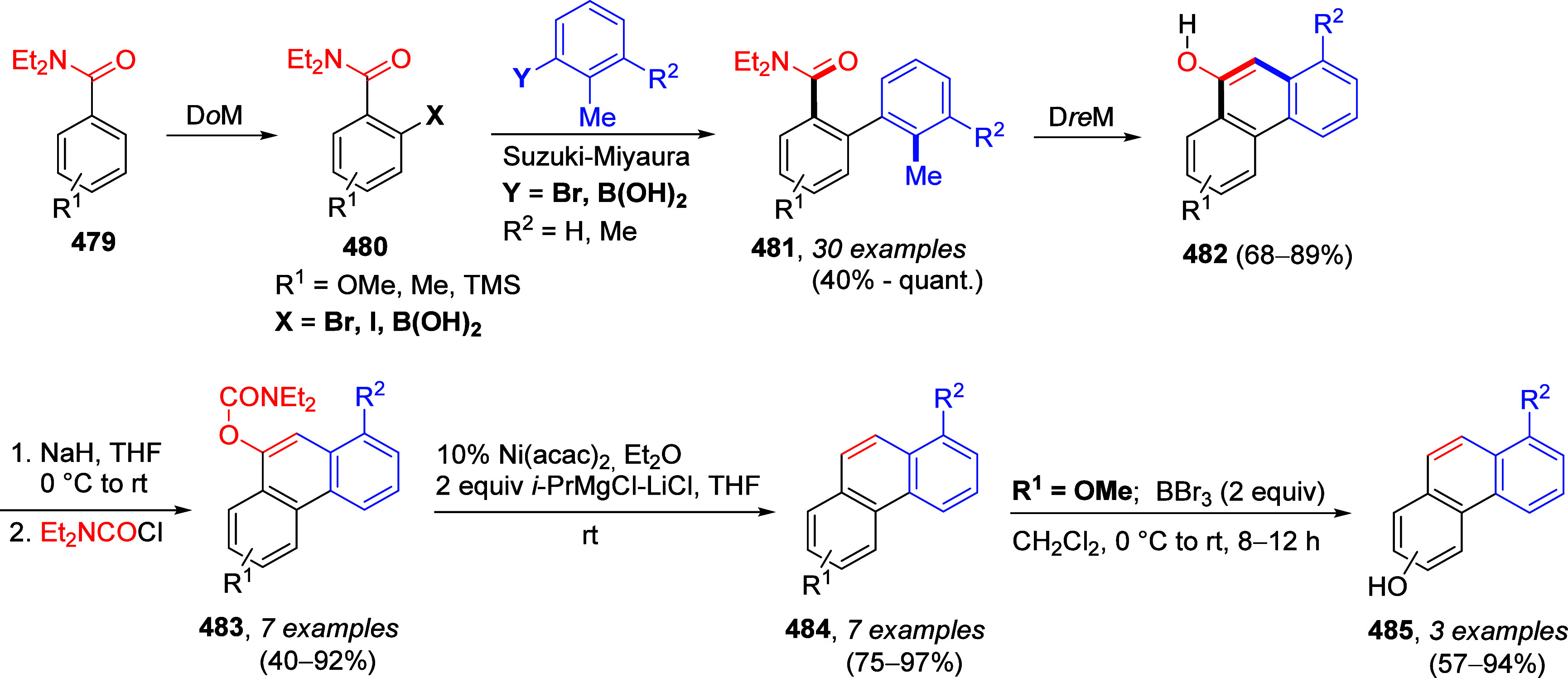
Synthesis of a Series of Hydroxylated 1-Methylphenanthrenes **485** by a Combined D*o*M/Suzuki–Miyaura CC/D*re*M Sequence Adapted from ref (230). Copyright 2015 American Chemical Society.

Jo̷rgensen et al.^230^ reported a general, efficient, and regioselective synthesis of a series of hydroxylated 1-methylphenanthrenes **485** by a combined D*o*M/Suzuki–Miyaura CC/D*re*M sequence (Scheme 111). Additional diversity of this method was achieved by a regioselective D*o*M reaction instead of a D*re*M reaction, which gave higher substituted phenanthrols. Boronic acid benzamides and halobenzamides, **480**, were prepared by D*o*M reactions using standard procedures. CC of boronic acid benzamides with bromoarenes or vice versa proceeded in high yield under two different conditions to give both monomethyl and dimethyl biaryls, **481**, in moderate to excellent yields (40% to near quantitative). Further conversion of biarylamides, **481**, to phenanthrols, **482**, was carried out in good yield (68–89%) under standard LDA conditions using the D*re*M method for the final reductive cleavage of *O*-arylcarbamates, **483**, to the oxygenated 1-methylphenanthrenes, **484**, the initial observation from the Ni-catalyzed Kumada–Corriu CC reaction was applied, namely, that Grignard reagents reductively cleave aryl halides and *O*-carbamates. With this step, efficient regioselective synthesis of the series of 4-OMe 1-methylphenanthrenes **484** was carried out in high yields (75–97%). Finally, BBr_3_ deprotection of the methoxy derivatives afforded the corresponding phenols **485** in good to excellent yields (57–94%).

**Scheme 112:**
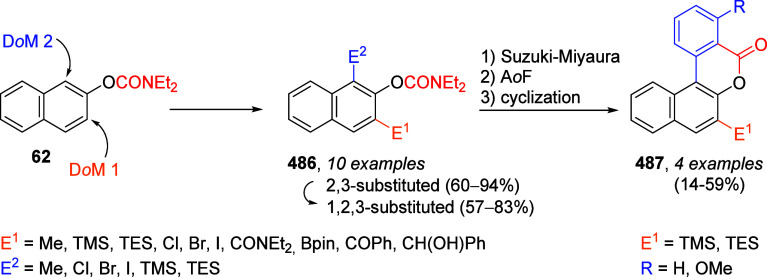
Regioselective Synthesis of 2,3-Di- and 1,2,3-Trisubstituted Naphthalenes **486** Using D*o*M strategies of carbamate **62**; Further Derivatization via Suzuki–Miyaura CC/A*o*F/Cyclization Sequence Leads to Benzonaphthopyranones **487** Adapted from ref (205). Copyright 2014 American Chemical Society.

Groom et al.^205^ developed a regioselective synthesis of 2,3-di- and 1,2,3-trisubstituted naphthalenes via D*o*M strategies of *N*,*N*-diethyl-*O*-naphthyl-2-carbamate, **62** (Scheme 112). Sequential LiTMP metalation–electrophilic quenching and *s*-BuLi/TMEDA (or *t*-BuLi)-electrophilic quenching of naphthyl-2-carbamate **62** provides a general route to contiguously substituted naphthalenes **486** with full regioselectivity and good yields. Further derivatization via *ipso*-halodesilylation and Suzuki–Miyaura CC leads to various phenyl-substituted halonaphthalenes (90–94% yield). Additional treatment with an excess of LDA in THF (to promote A*o*F rearrangement) followed by heating in acetic acid afforded the benzonaphthopyranones **487** in moderate yield.

**Scheme 113:**
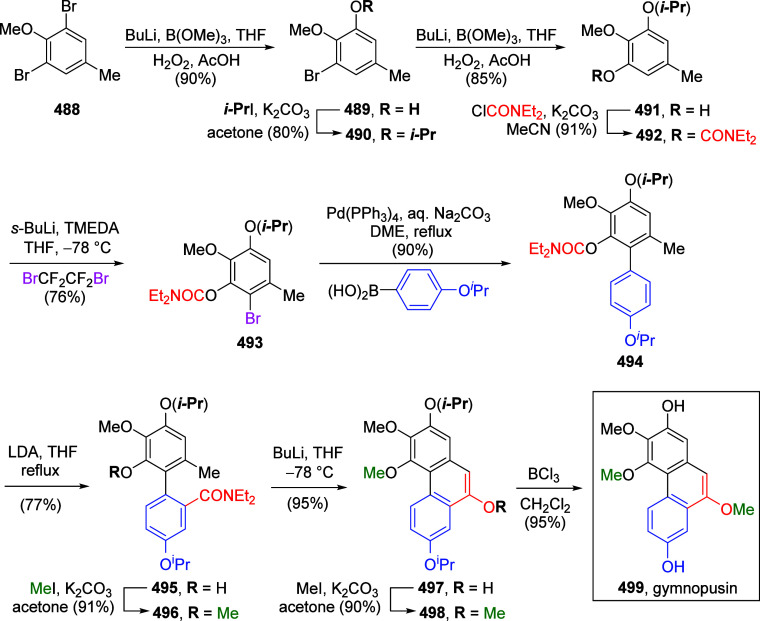
Total Synthesis of Gymnopusin, **499**, in 12 Steps Adapted with permission from ref (402). Copyright 2012 John Wiley and Sons.

Wang et al.^402^ prepared gymnopusin (**499**) in a total synthesis using a combined D*o*M, Suzuki–Miyaura CC, and D*re*M strategy to demonstrate the correct structure of **499** by regioselective assembly of the 9-phenanthrole core (Scheme 113). The natural gymnopusin was prepared from commercially available starting materials in 12 steps and with an overall yield of 18%. The value of this work lies in providing conditions for highly hindered Suzuki–Miyaura couplings, facilitating D*re*M-induced carbamoyl transfer (A*o*F) to produce hindered biaryls that are difficult or impossible to achieve by direct couplings, and in the advantages of D*o*M chemistry.

**Scheme 114:**
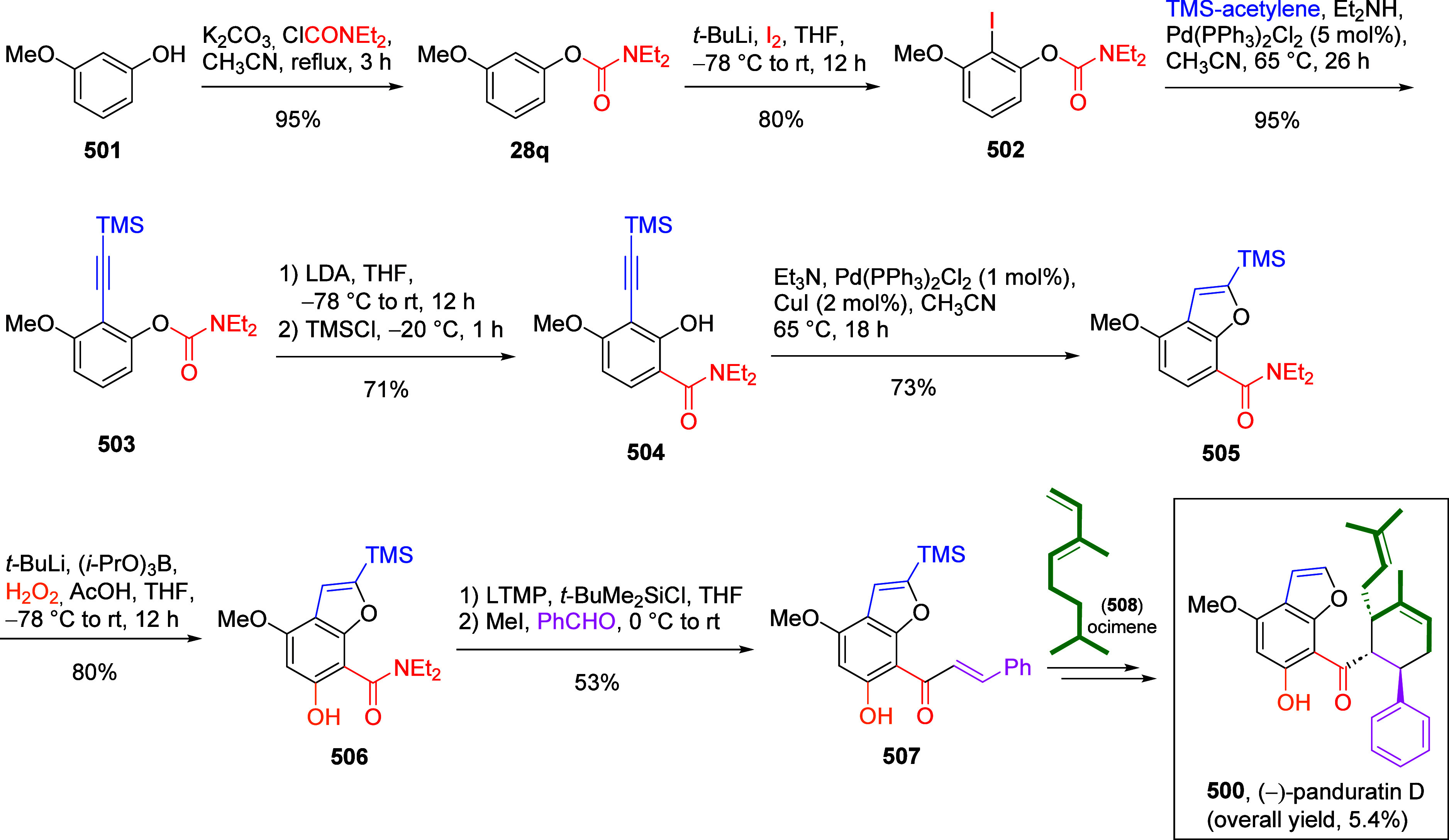
Enantioselective Total Synthesis of (−)-Panduratin D (**500**) via the Chalcone Key Structure (**507**) in Nine Steps Adapted with permission from ref (403). Copyright 1996 Royal Society of Chemistry.

Bavikar et al.^403^ reported an enantioselective total synthesis of (−)-panduratin D (**500**), a novel secondary metabolite against human pancreatic PANC-1 cancer cells, from commercial 3-methoxyphenol (**501**) in nine steps with an overall yield of 5.4% (Scheme 114). Key steps include Sonogashira coupling, A*o*F rearrangement, and D*o*M to form the furanochalcone unit (**506**) and tandem Si–C alkyl rearrangement/Claisen–Schmidt condensation to form the chalcone structure (**507**). The asymmetric Diels–Alder cycloaddition of chalcone **507** and ocimene (**508**) promoted by a chiral boron complex provides the final chiral cyclohexene core of (−)-panduratin D (**500**).

**Scheme 115:**
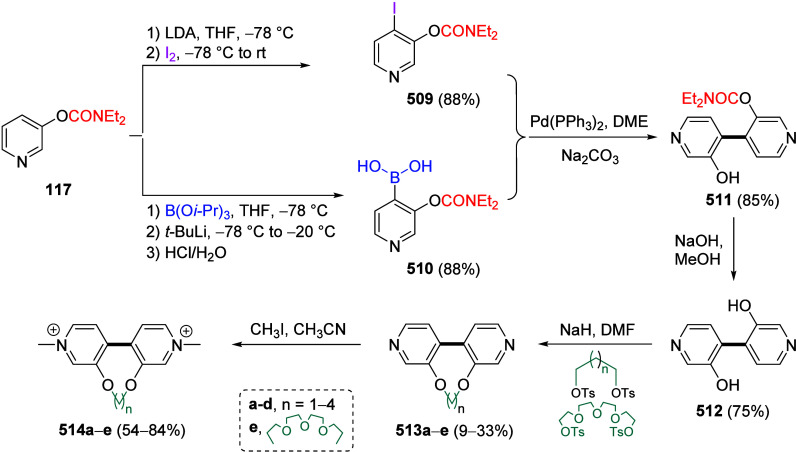
Synthesis of Viologens **514a**–**e** Using D*o*M/Suzuki CC Sequence as an Efficient Protocol to Prepare 4,4′-Bipyridine Derivatives (**511** and **512**) before the Final Tethering Reactions Adapted with permission from ref (404). Copyright 2007 John Wiley and Sons.

Benniston et al.^404^ synthesized a series of viologens using a successful alternative strategy via D*o*M of pyridin-3-yl diethylcarbamate (**117**) to generate 4-iodo- and 4-boronato-pyridin-3-yl carbamates **509** and **510**. They used a standard Suzuki coupling to combine them and isolate the monoprotected derivative **511** (Scheme 115). The last carbamoyl group was easily removed from **511** with NaOH to give **512** in 75% yield. Tethering reactions of **512** were carried out by deprotonation of the hydroxy groups and subsequent slow addition of ditosyloxyalkane/ether. The isolated yields of **513a**–**e** were modest (9–33%). The simple methylation of **513a**–**e** with iodomethane, followed by anion exchange and recrystallization, finally gave the desired viologen analogues **514a**–**e** as crystalline solids in 54–84% yield.

### 9.4 Direct C–H/C–O Bond Arylation, Benzylation, and Alkynylation

**Scheme 116:**
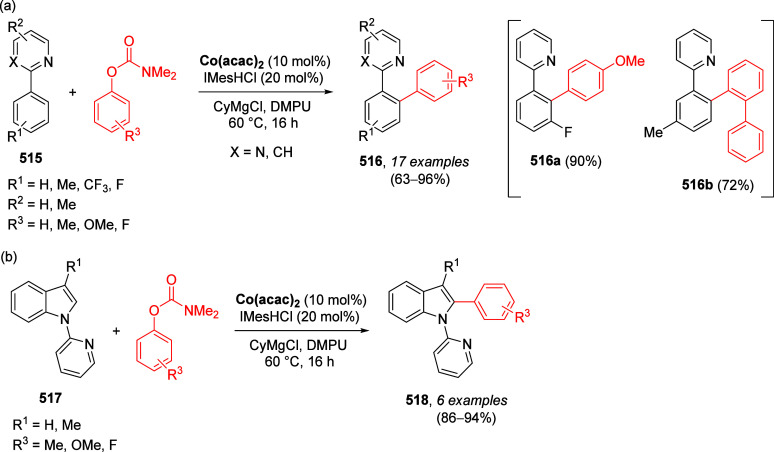
(a) Cobalt-Catalyzed C–H/C–O Arylation of Arenes **515** with Aryl *O*-Carbamates; (b) Cobalt-Catalyzed Direct Arylation of Heteroarenes **517** with Aryl *O*-Carbamates Adapted with permission from ref (343). Copyright 2012 John Wiley and Sons.

Song and Ackermann^343^ reported the use of a highly efficient cobalt catalyst for the direct C–H bond arylation of arenes with readily accessible aryl *O*-carbamates via a challenging C–H/C–O bond cleavage. Both electron-rich and electron-poor functionalized aryl *O*-carbamates were converted with remarkably high catalytic efficiency, even at room temperature (Scheme 116a). Moreover, both arenes with electron-donating substituents and arenes with electron-withdrawing substituents gave the desired products **516** in high yield. A variety of different pyridyl-directing groups could also be employed to achieve site selectivity. An intramolecular competition experiment gave the biaryl product **516a** in excellent yield (90%) by functionalization of the kinetically more acidic C–H bond. Aryl-*O*-carbamates functionalized with sterically hindered substituents in the *ortho* position gave sterically crowded product **516b** in high yield (72%), demonstrating the suitability of aryl-*O*-carbamates for strategies combining D*o*M and C–H bond functionalization. In addition, mono-*N*-substituted indoles **517** were selectively arylated at the C2 position, allowing the synthesis of sterically loaded heterobiaryls **518** (Scheme 116b). This capability could be useful in future applications involving asymmetric C–H bond arylation. The reader is referred to section 8.6.3. for more details.

**Scheme 117:**
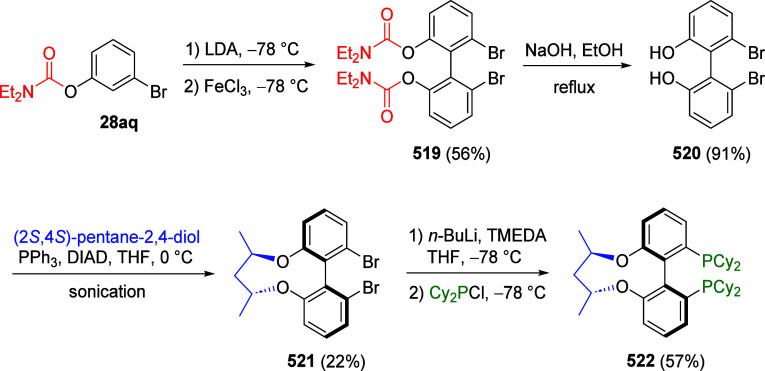
Synthesis of an Electron-Rich, Axially Chiral, TunePhos-Type Biaryl Ligand **521** Using Regioselective D*o*M Lithiation and Oxidative Coupling in the Crucial Reaction Step Adapted with permission from ref (215). Copyright 2009 Elsevier.

Zou et al.^215^ prepared an electron-rich, axially chiral TunePhos-type biaryl ligand, **522** (Scheme 117), which was used in a Rh-catalyzed asymmetric hydrogenation. The initial 3-bromophenyl carbamate, **28aq**, was subjected to D*o*M lithiation, followed by oxidative coupling with FeCl_3_ to form the biaryl bacbone **519** in 56% yield. The carbamate groups were then removed under basic conditions to form the dibromo substituted biphenol, **520**, which was then subjected to Mitsunobu reaction in the presence of a chiral 2,4-diol. Two diastereomers with opposite axial chiralities were obtained, from which the desired diastereomer **521** was obtained by column chromatographic purification in 22% yield. Finally, **521** was treated with *n*-BuLi at low temperature to perform Li–Br exchange. Subsequent quenching with Cy_2_PCl afforded the ligand **522** in moderate yield.

**Scheme 118:**
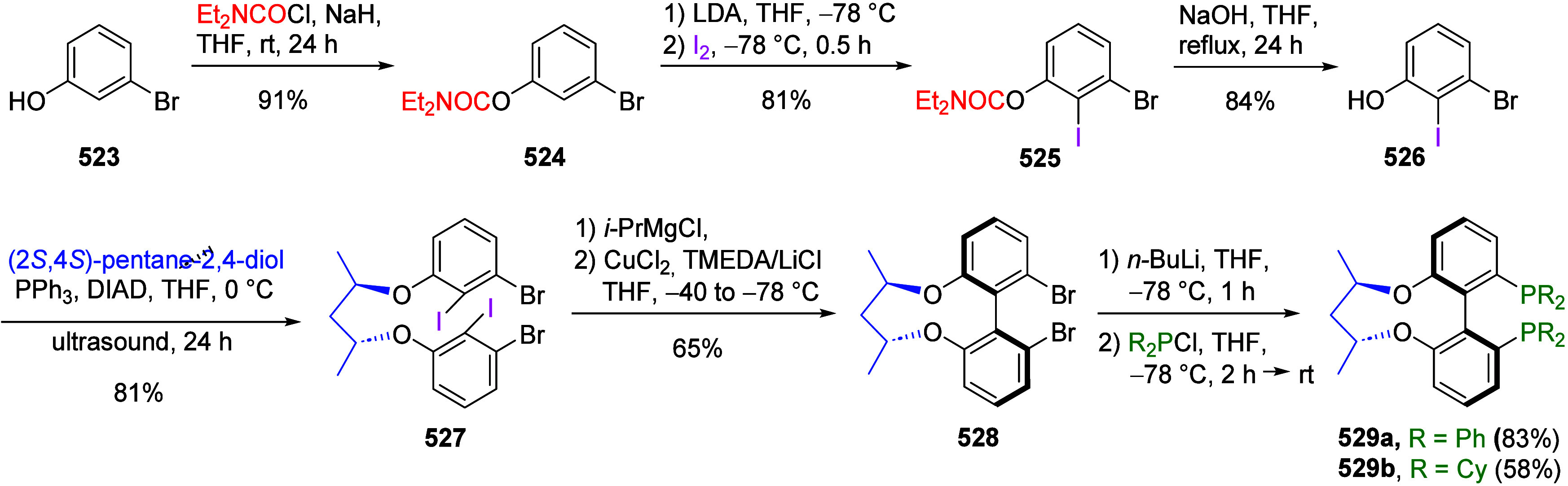
Synthesis of an Axially Chiral Bidentate Biaryl C3*-TunePhos Ligand **529a**, Using Regioselective D*o*M Lithiation, Quenching with I_2_, Deprotection, Mitsunobu Reaction with (2*S*,4*S*)-Pentane-2,4-diol, and Oxidative Coupling in the Crucial Reaction Step Adapted with permission from ref (405). Copyright 2017 Thieme.

Another example is an efficient and modular synthesis of an axially chiral bidentate biaryl ligand of the C3*-TunePhos type (**529a**) (Scheme 118) developed by Deng et al.^405^ The synthesis started with the protection of commercially available 3-bromophenol (**523**), giving carbamate **524** in excellent yield, which was subjected to D*o*M lithiation and then quenched with I_2_, giving the aryl iodide **525** in good yield. Saponification of compound **525** afforded phenol **526**, which was converted to the coupling precursor **527** in high yield by reaction with (2*S*,4*S*)-pentane-2,4-diol. The key oxidative coupling reaction with CuCl_2_ and TMEDA/LiCl led to the intermediate **528** in high yield, which could be prepared on a gram scale. The dibromide **528** was treated with *n*-BuLi at low temperature and quenched with Ph_2_PCl to give the ligand **529a** in 27% overall yield.

**Scheme 119:**
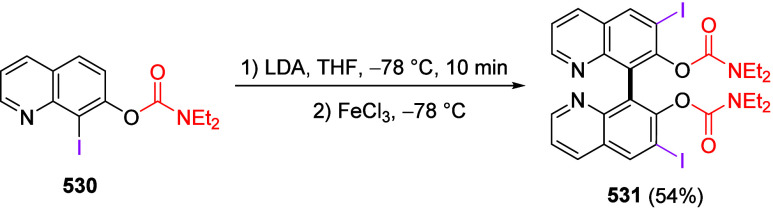
Direct Synthesis of Highly Substituted Biquinolyl **531** by an Efficient Tandem HalD-Dimerization Process Adapted from ref (406). Copyright 2004 American Chemical Society.

Blakemore et al.^406^ directly prepared 7,7′-bis(((diethylamino)-carbonyl)oxy)-6,6′-diiodo-8,8′-biquinolyl (**531**) in 54% yield with regioselective D*o*M of **530**, followed by the addition of anhydrous ferric chloride (Scheme 119). An efficient tandem HalD-dimerization process is an alternative for the synthesis of highly substituted biaryl molecules.

**Scheme 120:**
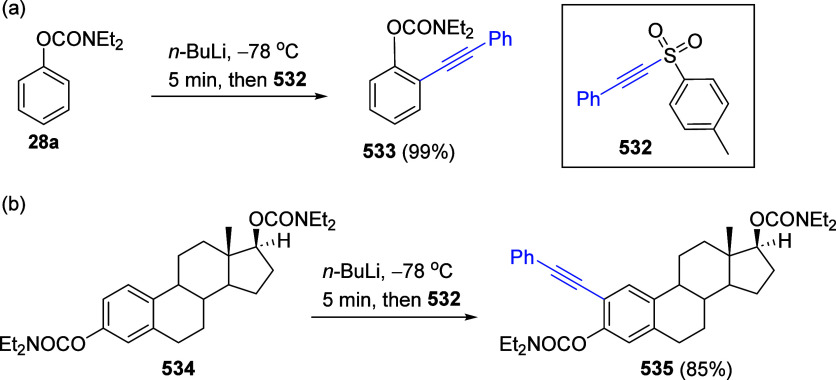
Regioselective Alkynylation of (a) Phenyl *O*-Carbamate, to Form **533**, and (b) Estradiol Derivative (**534**) Adapted with permission from ref (407). Copyright 2012 John Wiley and Sons.

Ruano et al.^407^ demonstrated a simple and efficient regioselective alkynylation, which made use of D*o*M quenching with electrophile source *p*-tolylsulfonyl acetylene (**532**), first with phenyl *O*-carbamate, to form **533** in a quantitative yield, followed by an estradiol derivative **534** to form **535** in high yield (Scheme 120).

## 10 OAm as Directing Group in *ortho*-Functionalization via C–H Activation Couplings

### 10.1 Introduction to C–H Activation

**Figure 8:**
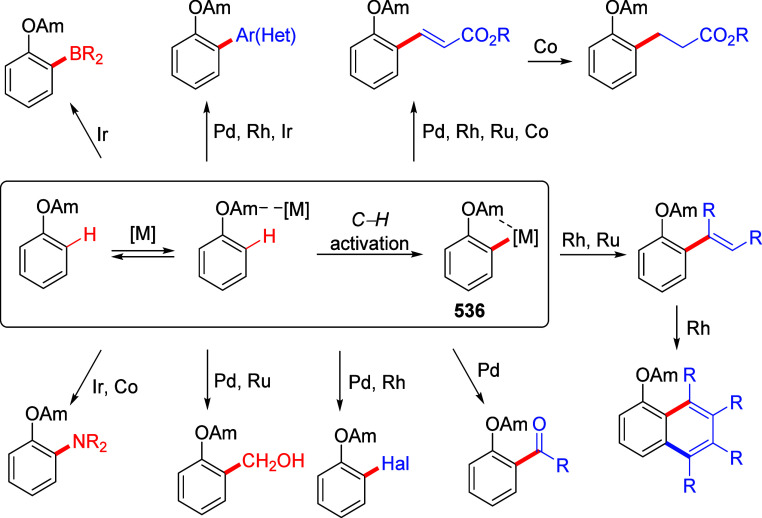
Scope of transition metal-catalyzed *O*-carbamate-directed C–H activation followed by coupling reaction to afford *ortho*-functionalized ArOAm products.

Selective C–H activation at a specific and tactical site among the occupancy of numerous C–H bonds of an aromatic compound is a powerful technique of C–H functionalization. The most effective of methodologies thus far utilized for accomplishing site selectivity has been via the application of Lewis-basic directing groups in proximal C–H bond functionalization.^408−421^ Similar to the utility of a DMG in D*o*M chemistry, these directing groups function to lead the transition metal to specific C–H bonds through applying distance and geometry as factors to differentiate between proximal and distal C–H bonds. However, installation and removal of directing groups has some drawbacks, as it can be costly and labor intensive. Therefore, to improve the effectiveness of this method, attachment of the DMG to the substrates by a transient covalent bond has been considered for rendering the directing group catalytic.^422^ In this way, the *O*-carbamate (OCONR_2_) DMG has been developed for Pd, Rh, Ru, Ir, and Co catalysts in a wide range of C–H activation transformations (Figure 8).

Due to the abundance of C–H bonds in organic molecules, endless opportunities reside in the structural alteration of compounds via C–H activation. However, attaining site selectivity in C–H functionalization can be extremely difficult to achieve. Therefore, a key concern involves identifying parameters which may help in guiding the catalysts to the desired C–H bond. One potentially valuable tactic involves the development of catalytic systems that can characterize C–H bonds based on their distal and geometric association with respect to an existing functional group. Thus, to advance this method, it is necessary to develop practical DMGs that are (1) easy to install and remove, (2) have a lower mass, and (3) coordinate weakly to metal catalysts so that external ligands can coordinate and exert a greater effect on the metal catalysts, thereby controlling reactivity, stereoselectivity, and site selectivity.

In general, pyridines, oxazolines, and imines are broadly used as DMGs in directed C–H activation with transition-metal catalysts to achieve reactivity through a chelating effect in the employment of coordinating heteroatoms. Throughout the literature, these DMGs have been widely successful at promoting transition-metal-catalyzed C–H cleavage, in part because the cyclometalated intermediates are thermodynamically stable and simple to separate. Yet, due to the thermodynamic stability of the metalacycle complexes (**536**) (Figure 8) created in association with strongly coordinating DMGs, the functionalization stage can be problematic at times when a lack of reactivity is present. Disadvantages of strongly coordinating DMGs also arise when (1) two equivalents of a strongly coordinating substrate could bind simultaneously to a metal, thus preventing a ligand from binding, and (2) a metal complex coordinated to an electron-donating ligand, and the substrate may not hold suitable electronic properties required for the C–H bond cleavage. As a result, an alternative methodology has been established in which weakly coordinating DMGs or existing functional groups are applied to drive the metalation of C–H bonds. In this way, thermodynamically less stable metalacycle complexes can form, which in turn can be more easily functionalized than strongly coordinated complexes, greatly expanding the knowledge of C–H activation transformations. The extensive progress made by the ArOAm DMG is a remarkable example of this strategy.

### 10.2 OAm as a Directing Group for C–H Activation

It is fundamental to investigate a variety of different catalysts or strategies that can selectively activate C–H bonds at numerous sites for the late-stage modification of sophisticated intermediates or end products. The effectiveness of the simple and weakly coordinating ArOAm directing group is demonstrated by its compatibility with a comprehensive variety of substrates, transformations, metal catalysts (Pd, Rh, Ru, Ir, and Co), and mild conditions (room temperature).

As in the case of other directing groups, the most studied transformation with ArOAm is Pd-catalyzed ligand-directed C–H activation followed by C–C coupling to give *ortho*-arylated, alkenylated, or carbonylated products. With rhodium and ruthenium catalysis, alkenylation predominates, while iridium proved to be superior for borylation reactions. Transition metal-catalyzed ligand-directed C–H functionalization has also been utilized for C–N, C–O, and C–halogen bond formation (Table 27).

**Table 27:**
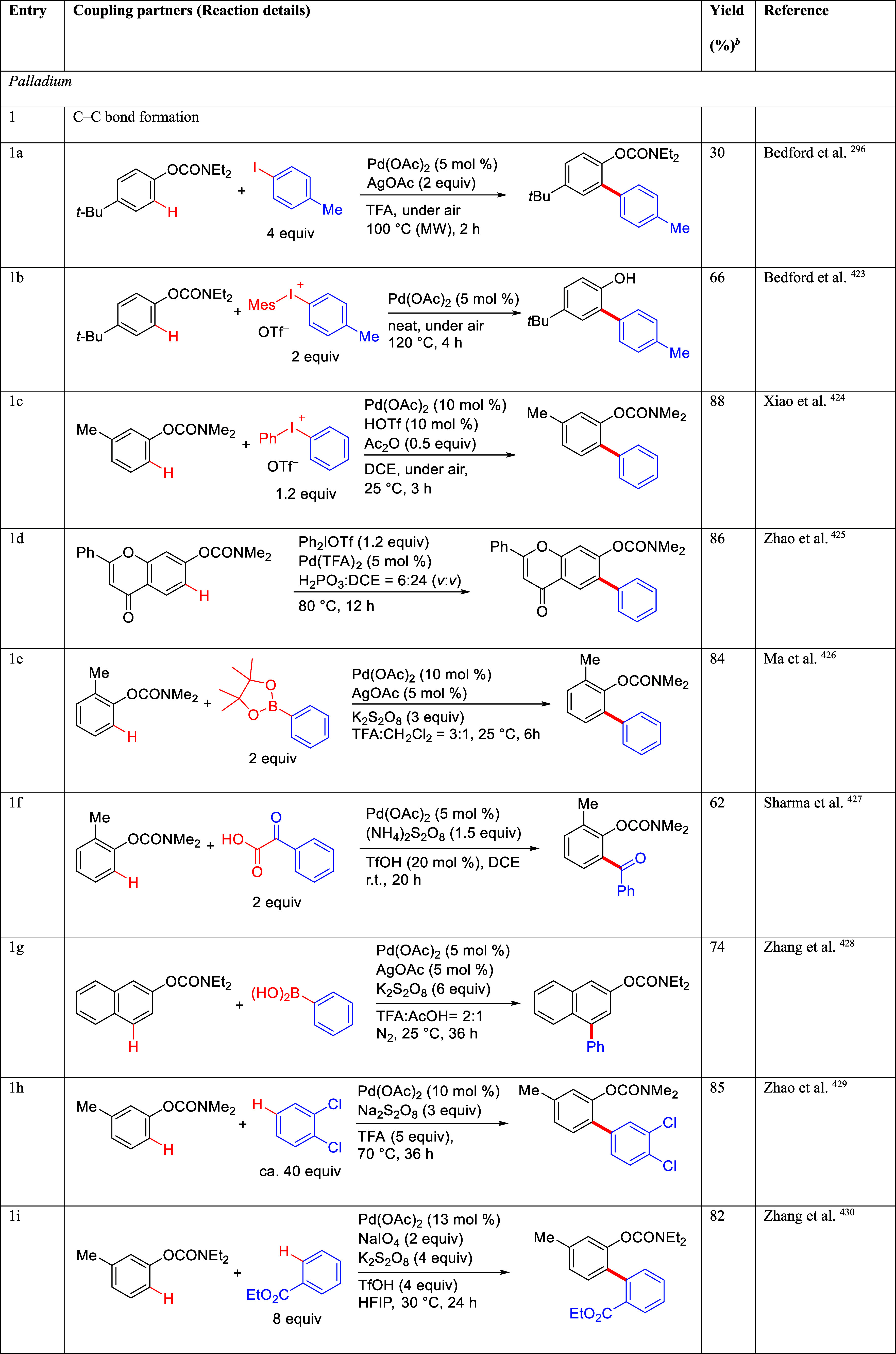

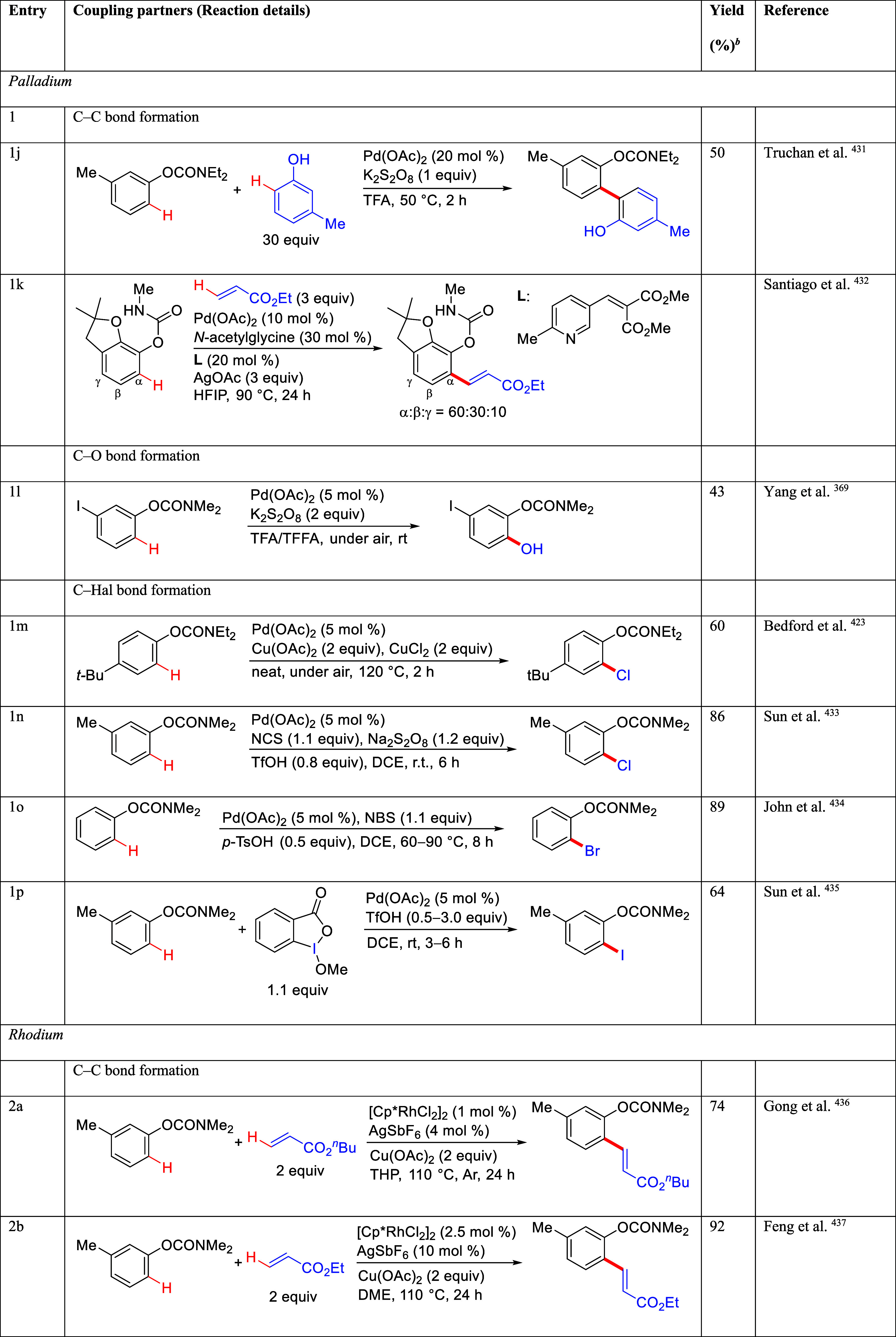

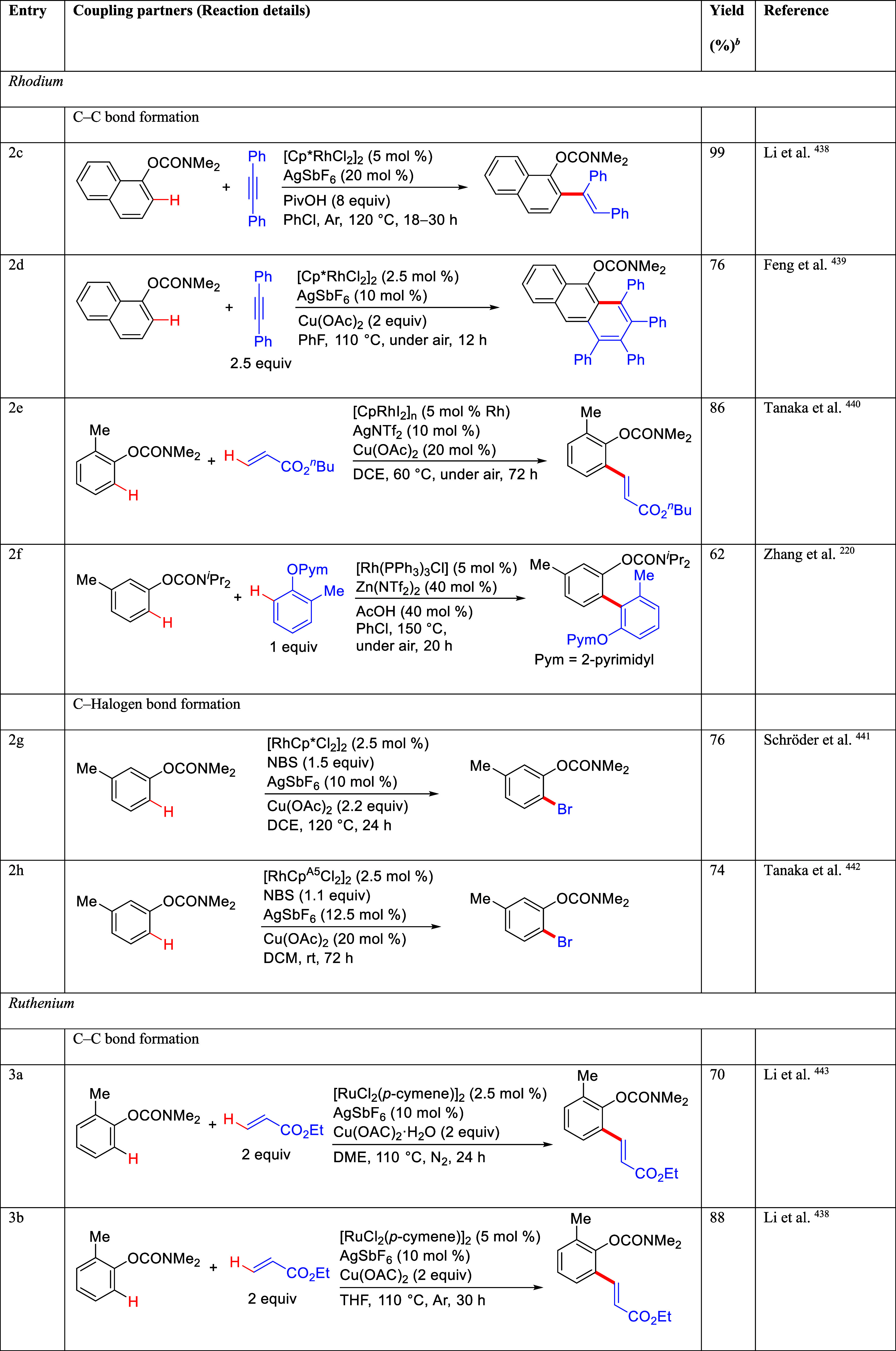

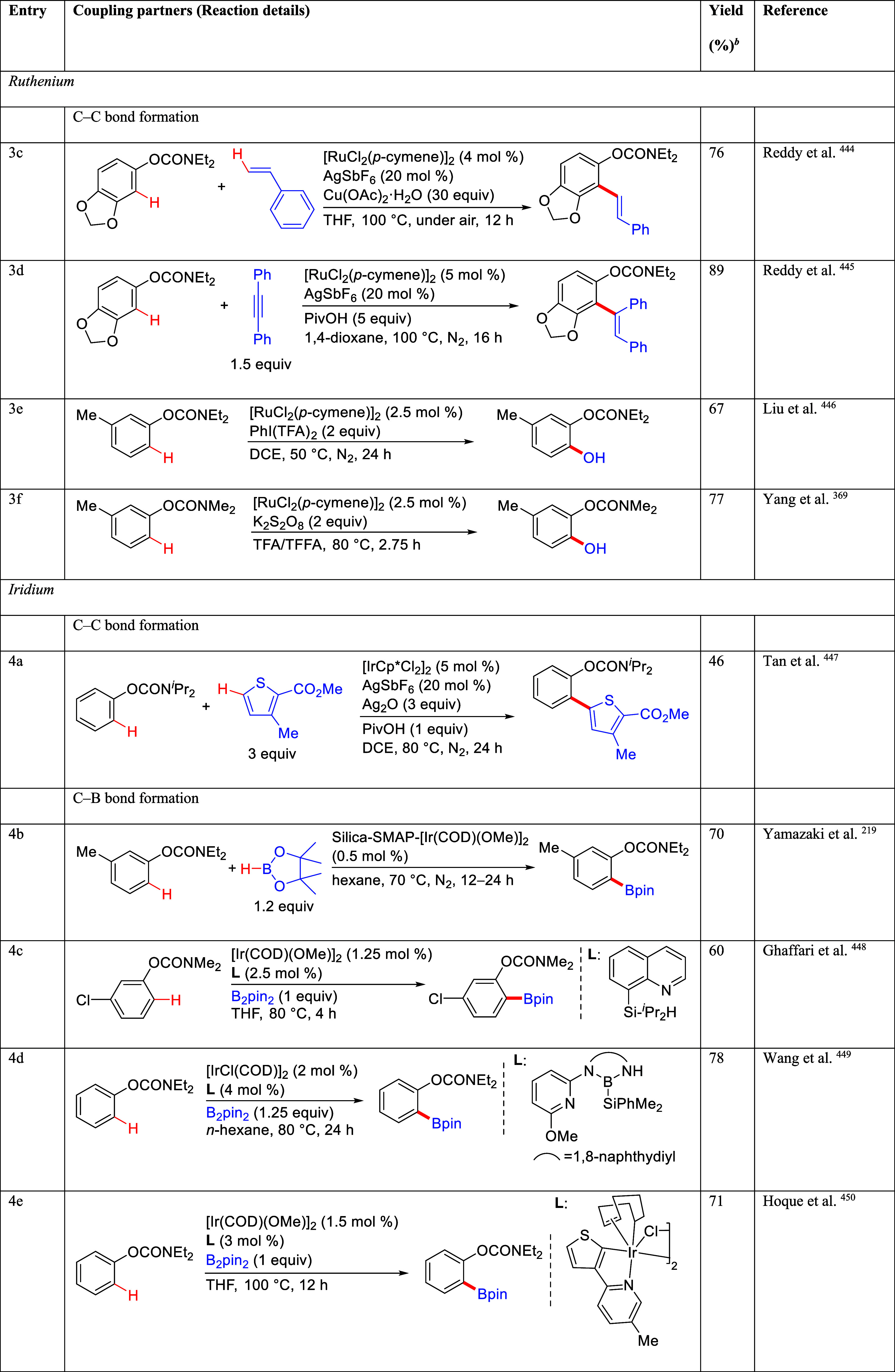

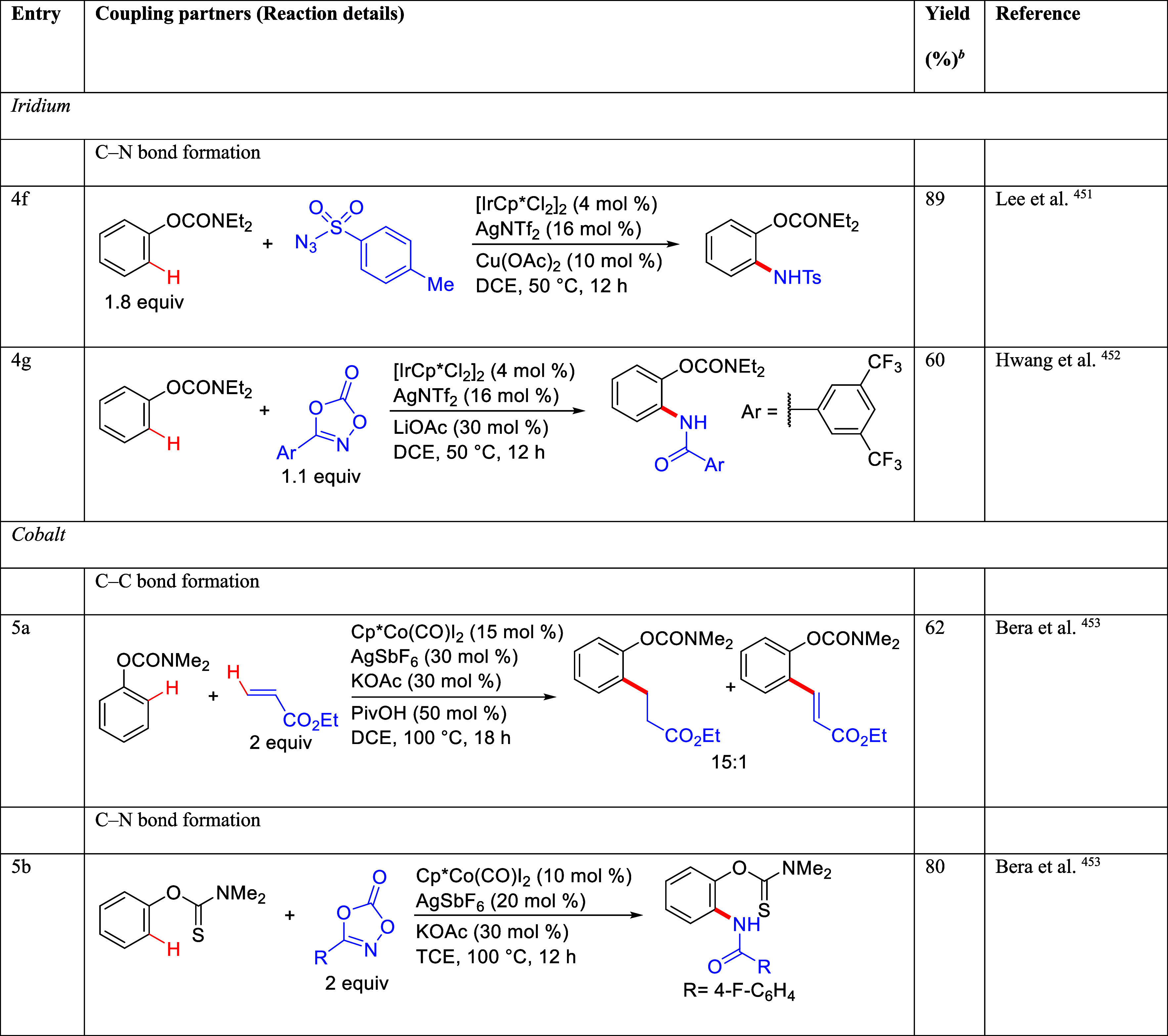
Representative Examples of Transition-Metal-Catalyzed Ligand-Directed C–H Functionalization at ArOAm^a^

a (a) An attempt has been made to show representative examples from the substrate scope described in each reference to allow comparison between methods. (b) Isolated yield of pure product.

#### 10.2.1 Palladium

**Figure 9:**
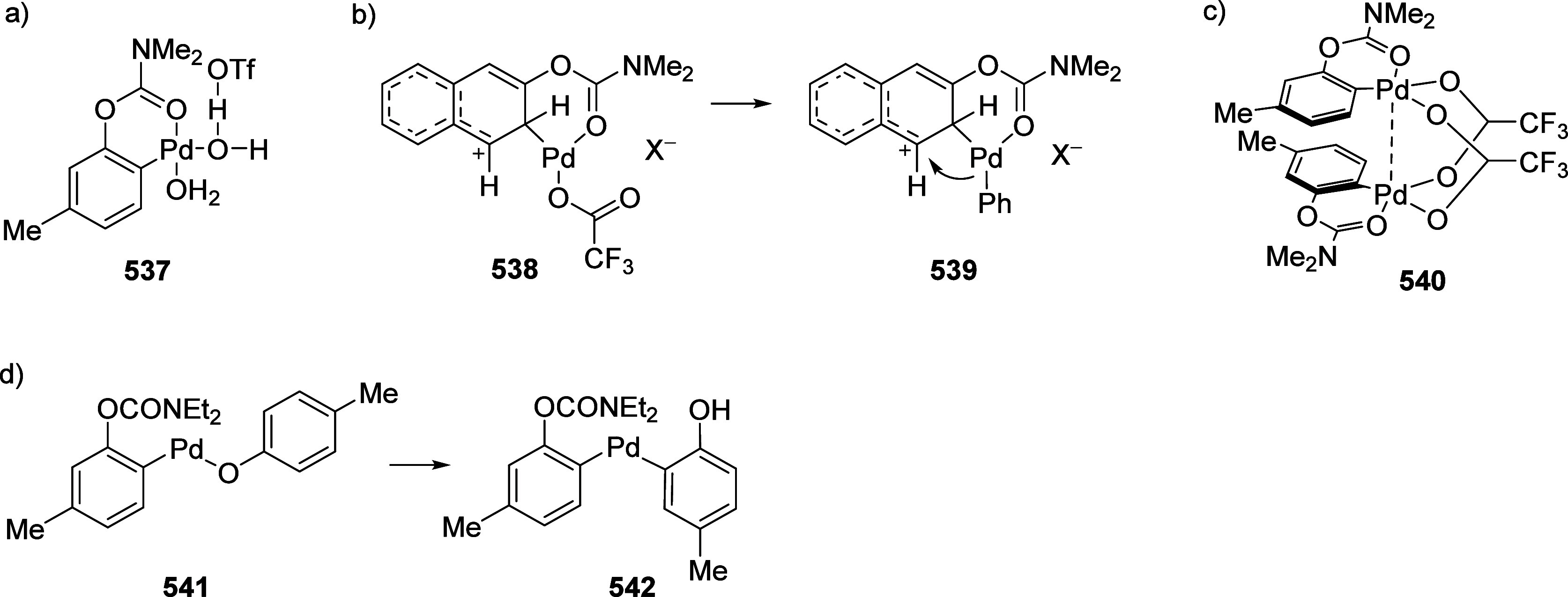
(a) *O*-Phenylcarbamate palladacycle intermediate **537** prepared and NMR characterized by Xiao et al.^424^ Adapted from ref (424). Copyright 2010 American Chemical Society. (b) Proposed intermediates **538** and **539** in *meta*-arylation of *O*- β-naphthyl carbamates by Zhang et al.^428^ Adapted with permission from ref (428). Copyright 2014 Royal Society of Chemistry. (c) A bimetallic Pd species **540** prepared, and X-ray characterized by Zhao et al. Adapted from ref (429). Copyright 2010 American Chemical Society. (d) Intermediates **541** and **542** in Pd-catalyzed oxidative Ar–H/Ar–H CC between ArOAm and *p*-cresol as coupling partners, as proposed by Truchan et al. Adapted with permission from ref (431). Copyright 2019 Thieme.

The first palladium-catalyzed *ortho*-arylation using the carbamate group as the directing group for *ortho*-C–H bond activation was developed by Bedford et al.^296^ in 2009 (Table 27, entry 1a). Relatively low monoselectivity and yields were subsequently improved by using diaryliodonium salts as coupling partners^423^ (entry 1b). In this case, in the absence of Ag salts under solvent-free conditions, CC and *in situ* carbamate deprotection were observed to afford monoarylated free phenol products with excellent selectivity. Under modified reaction conditions, Xiao et al.^424^ found that the acid additives were important for the outcome of the reaction, either by supporting the C–H deprotonation step or by stabilizing the active Pd intermediates (entry 1c). The authors succeeded in the preparation and NMR characterization of *O*-phenylcarbamate palladacycle **537**, a plausible reaction intermediate likely formed by acyloxy-directed Pd(II) insertion into the C–H bond *ortho* to the carbamate (Figure 9a), and they proposed a mechanism operating via a Pd(II)/Pd(IV) species. More recently, Zhao et al.^425^ used this methodology for a regioselective arylation of 7-hydroxyflavone derivatives at the C6 position (Table 27, entry 1d).

In addition to aryl iodides and diaryliodonium salts, a Pd-catalyzed *ortho*-directed arylation of *O*-phenylcarbamates was carried out by Ma et al.^426^ using pinacol aryl boronates (entry 1e). Sharma et al.^427^ developed *ortho*-acylation by using α-oxocarboxylic acids as coupling partners to provide benzophenone products in moderate to good yields (entry 1f). Recently, this method was used to prepare a series of 6-acyl 7-*O*-carbamate coumarins that exhibited anti-inflammatory activity against RAW 264.7 macrophage cell line.^454^ Selective *meta*-arylation of *O*-β-naphthyl carbamates with a series of phenylboronic acids as coupling partners was investigated out by Zhang et al.^428^ (entry 1g). The *O*-carbamate group proved to have a unique effect, allowing *ortho*-carbometalation to generate the Wheland intermediate **538**, which, during the reaction pathway, passes into the products after transmetalation to **539** and nucleophilic attack of the aryl group in the *meta*-position (Figure 9b). It is noteworthy that the C–H bond *ortho* to the carbamate group is preserved throughout the reaction. Similar observations in Pd-mediated arylation of *O*-β-naphthyl carbamates were made by Ma et al.^426^ The mechanistic considerations of ligand-directed transition-metal C–H activation have been reviewed comprehensively elsewhere.^408−421^

A complementary Pd-catalyzed oxidative Ar–H/Ar–H CC reaction of *O*-phenylcarbamates used excess amounts of simple arenes (e.g., benzene, *o*-dichlorobenzene) as coupling partners avoiding the need for aryl iodide, hypervalent iodine, or boronate reagents, was proposed by Zhao et al.^429^ in 2010 (entry 1h). The authors proposed a distinct Pd(0/II) catalytic cycle with a plausible dimeric bimetallic Pd intermediate **540** (Figure 9c), in which strongly acidic additive TFA plays an important role in the reaction. Complexes like these were prepared and studied by John et al.^434^

Five years later, Zhang et al.^430^ developed a unique Pd(II)-catalyzed chelate-assisted oxidative Ar–H/Ar–H CC reaction between two different coupling partners with weakly coordinating directing groups to afford a broad range of 2,2′-difunctional biaryls (entry 1i). Screening through a large variety of the reaction conditions, the authors reported that the choice of hexafluoroisopropanol (HFIP) as reaction solvent and sodium periodate/potassium persulfate as the oxidizing agents were crucial factors in this reaction. No products were observed in other common solvents such as (e.g., DMSO, DMF, ethanol), and the authors suggested that HFIP could serve not only as a solvent system but also as an effective ligand for palladium to promote this reaction. Symmetric biaryls, a result of homodehydrogenative CC, were obtained in good yields when only one coupling partner, e.g., ArOAm, was present in the reaction mixture.^430^

Phenols were subsequently described by Truchan et al.^431^ to generate 2,2′-dihydroxy- and 2,4′-dihydroxybiphenyls with relatively low regioselectivity in both coupling partners, phenols, and carbamates (entry 1j). In the proposed Pd(II)/Pd(0) mechanism with *p*-cresol as coupling partner, the aromatic ring was activated by initial coordination through the oxygen atom to the palladacyclic intermediate (similar to Figure 9a), giving rise to **541**, which then rearranged to the regioisomeric complex **542**, as shown in Figure 9d. In the case of *para*-unsubstituted phenol, two isomeric products were formed.

In the Pd-catalyzed oxidative C–H olefination of arenes, known as the Fujiwara–Moritani reaction, Santiago et al.^432^ observed some regioselectivity in the functionalization of the pesticide carbofuran with ethyl acrylate to give a 60:30:10 mixture of α:β:γ regioisomeric products, attributed to the *ortho*-directing properties of the *O*-carbamate group (entry 1k). In addition to the *ortho*-directed C–C bond forming reactions, ArOAm compounds were also found to be suitable substrates in the Pd-catalyzed formation of C–O (entry 1l)^369^ and C–halogen bonds [entries 1m–1p)].^423,433−435^ There is a strong desire to use more sustainable approaches for the synthesis of halogenated aromatic compounds.^455^ Under the same reaction conditions as in entry 1b, the use of the Cu(oAc)_2_/CuCl_2_ system instead of aryl iodide led to monochlorination (entry 1m).^423^ Under milder reaction conditions, *ortho*-halogenated products were obtained in good to excellent yields upon reaction with the corresponding *N*-halosuccinimide (entries 1n and 1o)^433,434^ Sun et al.^435^ developed an approach to access *ortho*-iodinated phenols through Pd(II) catalyzed C–H activation using cyclic hypervalent iodine(III) reagents (Togni’s reagent and related), which serve not only as an oxidant but also as a iodine source in sp^2^ C–H iodination (entry 1p). The developed Pd-catalyzed *ortho* C–H activation has seen many practical applications in synthetic organic chemistry,^255,456^ including the modification of estrone and estriol derivatives.^457,458^

#### 10.2.2 Rhodium

In contrast to palladium catalysis, directed *ortho* C–H olefination predominates in rhodium-catalysis. The first Rh(III)-catalyzed directed oxidative olefination of phenol carbamate was reported by Gong et al.^436^ (entry 2a) and Feng et al.^437^ (entry 2b). Treatment of a wide variety of phenol and 2-naphthol carbamates with acrylates and styrenes using [Cp*RhCl_2_]_2_ as catalyst, AgSbF_6_ as halide scavenger, and Cu(OAc)_2_ as oxidant, gave the desired olefins in moderate to excellent yields and high regioselectivity.^436,437^ This protocol was subsequently used to obtain a range of advanced phenol and naphthol derivatives.^459−462^ An *ortho*-directed Rh-catalyzed alkyne hydroarylation reaction was carried out using internal alkynes as coupling partners to afford substituted alkenes with high regio- and stereoselectivity (entry 2c).^438^ Under slightly modified reaction conditions (by using Cu(OAc)_2_), the reaction led to polycyclic aromatic compounds (entry 2d).^439^ Recently, Tanaka et al.^440^ succeeded in the oxidative *ortho*-olefination of phenyl carbamates with both acrylates and styrenes under mild conditions using Rh(III) complex bearing an unsubstituted cyclopentadienyl (Cp) supporting ligand in place of the commonly used pentamethylcylopentadienyl (Cp*) ligand, with a broad substrate scope (entry 2e). *Ortho*-unsubstituted substrates generally returned a mixture of mono- and disubstituted products. The reaction mechanism was scrutinized experimentally and theoretically, suggesting that the less sterically hindered CpRh(III) complex can stabilize the transition states of the reaction pathway more than the Cp*Rh(III) complex.

The first example of a dual Rh-catalyzed chelate-assisted oxidative Ar–H/Ar–H CC reaction between two different coupling partners (one being an *O*-carbamate) having strongly and weakly coordinating directing groups was reported by Zhang et al.^220^ in 2019 (entry 2f).

**Figure 10:**
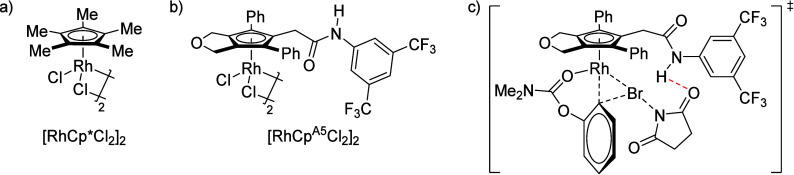
Structures of (a) [RhCp*Cl_2_]_2_, (b) [RhCp^A5^Cl_2_]_2_, and (c) suggested transition state in the mechanism of [RhCp^A5^Cl_2_]_2_-catalyzed *ortho*-bromination of ArOAm with NBS, indicating peculiar hydrogen bonding between the acidic NH group of the Cp^A5^ ligand and the carbonyl group of NBS as proposed by Tanaka et al.^442^ Adapted with permission from ref (442). Copyright 2020 John Wiley and Sons.

Carbamates were successfully used in the Rh-catalyzed directed *ortho*-C–H bond bromination of arenes. Unlike the palladium catalyzed reactions (entries 1m–1p), the Cp*Rh(III) complex (Figure 10a) developed by Schröder et al.^441^ avoided the use of Bro̷nsted acid additives (entry 2g). However, this reaction required an elevated temperature and a stoichiometric amount of Cu(oAc)_2_. In turn, a modified cyclopentadienyl-rhodium(III) complex based on a pentasubstituted fulvene Cp^A5^Rh(III) (Figure 10b) developed by Tanaka et al.^442^ was able to catalyze the *ortho*-bromination of *O*-phenyl carbamates with NBS at room temperature with only a catalytic 20 mol % amount of Cu(oAc)_2_ (entry 2h; compare with entry 2g). Unlike the Cp* ligand, the presence of the acidic secondary amide moiety on the Cp^A5^ ligand accelerated bromination through the hydrogen bonding between the acidic NH group of the Cp^A5^ ligand and the carbonyl group of NBS, as suggested in the transition state shown in Figure 10c.

**Scheme 121:**
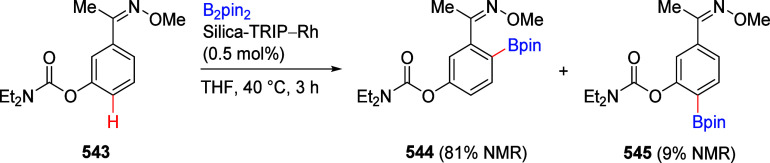
Competitive *ortho*-Borylation of Compound **543** Bearing Carbamate and Oxime Ether Directing Groups with (Silica-TRIP)–Rh System Prepared *in Situ* from Silica-SMAP and [Rh(OH)(COD)]_2_ Adapted from ref (463). Copyright 2011 American Chemical Society.

On the other hand, compared with oxime ether and other nitrogen-containing functional groups, carbamate acted as a pure directing group in Rh-catalyzed borylation with the supported triptycene-type bridgehead triarylphosphine (silica-TRIP)–Rh system, as shown by Kawamorita et al.^463^ Borylation *ortho*- to the carbamate into **545** was detected only in low (9%) NMR yield (Scheme 121).

#### 10.2.3 Ruthenium

Under the same reaction conditions used for Cp*Rh(III) catalysis, the groups of Ackermann (entry 3a),^443^ Wang (entry 3b),^438^ and Jeganmohan (entry 3c)^444^ reported the Ru(II)/*p*-cymene complex-catalyzed oxidative *ortho*-C–H alkenylation of ArOAm. The catalytic system was proved to be remarkably active and highly regioselective, even feasible in an aerobic manner with ambient air as the terminal oxidant. The protocol was suitable for acrylates, styrenes, and internal alkynes (entry 3d)^445^ as coupling partners.

Ruthenium catalysts enabled efficient *ortho*-directed oxygenations of ArOAms under remarkably mild reaction conditions. [RuCl_2_(*p*-cymene)]_2_, as the catalyst of choice, combined with PhI(TFA)_2_ and DCE as the best oxidant and solvent, respectively, enabled *ortho*-oxygenations at only 50 °C in moderate to good yields (entry 3e).^446^ Yang et al.^369^ investigated a range of oxidants including Selectfluor, PhI(OAc)_2_, (NH_4_)_2_S_2_O_8_, Na_2_S_2_O_8_, and K_2_S_2_O_8_, with the latter proving to be the most effective in the trifluoroacetic acid/trifluoroacetic anhydride (TFA/TFAA) solvent system (entry 3f). The practicality of this new method was tested successfully on a gram-scale synthesis. Furthermore, this reaction found application in the key step of the synthesis of 2-methoxyestradiol.^464^

#### 10.2.4 Iridium

The propensity of iridium(III) to activate *ortho*-C–H bonds in ArOAm, forming Ir-cyclometallates and undergoing an efficient redox-active catalytic cycle that enables a directed oxidative C–H/C–H CC reaction between two (hetero)arenes, was first reported in 2018 by Tan et al. (entry 4a).^447^ A comparative reactivity study of iridium catalysis with rhodium catalysis for this transformation performed by the authors showed that IrCp*(III) performed better than RhCp*(III). Under otherwise identical conditions, no reaction was observed when [RhCp*Cl_2_]_2_ was used for the coupling reaction shown in entry 4a.^447^

There has been significant development in the field of *ortho*-directed iridium-catalyzed C–H borylations of aromatic substrates.^414,419^ Iridium-catalyzed borylation using silicon-constrained monodentate trialkylphosphine (SMAP) ligands, pioneered by the group of Sawamura^219^ showed a broad scope and excellent selectivity for *ortho*-directed borylations (entry 4b). To stabilize the Ir catalyst, the groups of Smith^448^ (entry 4c) and Li^449^ (entry 4d) developed N,Si- and B,N-bidentate ligands, respectively. Recently, Chattopadhyay’s group^450^ discovered a new class of C–H borylation catalysts with anionic Ir–C(thienyl) ligands instead of the neutral ligands from entries 4c and 4d. These catalysts were air stable and showed remarkable efficiency and selectivity in site-selective C(sp^2^)–H and C(sp^3^)–H borylation of various classes of aromatic, heteroaromatic, and aliphatic substrates (entry 4e). The iridium-catalyzed *O*-carbamate-directed *ortho*-C–H amidation of arenes with sulfonyl azides and dioxazolones as amidating reagent was reported by Chang’s research group (entries 4f and 4g).^451,452^

#### 10.2.5 Cobalt

Cobalt has also been used in the *ortho*-directed C–H functionalization of ArOAm but with only one example in the literature. In 2020, Bera et al.^453^ developed a selective carbamate-directed C–H alkylation under stable, high-valent, cost-effective cobalt(III) catalysis (entry 5a). The use of pivalic acid was crucial to suppress the formation of unsaturated cinnamic acid derivatives. Under the same reaction conditions, but in the absence of pivalic acid and with thiocarbamate as a directing group, the authors reported the coupling of aryldioxazolones to give products of selective C–H amidation (entry 5b).

### 10.3 Conclusion and Outlook

Transition metal-catalyzed directed C–H functionalization is a complementary tool to the well-established directed *ortho*-metalation (D*o*M) methods. Better selectivity and tolerance to functional groups, which can be achieved by selecting the appropriate metal in combination with other (ancillary) ligands that can further finetune the reactivity, can be considered as the main advantage of the former over D*o*M. Also, the transformations with transition metals are often insensitive to moisture or air and do not require flammable reagents or cryogenic reaction conditions in contrast to reactions involving alkyl lithiums.

Among the selective intermolecular *ortho* C–H activation approaches, the *ortho*-C–H activation predominates. Reaching the distal positions of arene systems is very challenging, with ArOAm as the directing group, and there seems to be only one successful example of this (Table 27, entry 1g).

Despite their success, many transition metal-catalyzed directed C–H functionalization reactions using ArOAm as substrates remain unexplored, including C–H alkynylation, alkylation, alkoxylation, chalcogenation, trifluoromethylation, and deuteration,^465^ as well as C–H activation of distal bonds, already demonstrated with *N*-carbamates,^466^ to name a few. There are no reports of carboxylation with CO_2_,^467^ or carbonylation with CO or less toxic supplements, such as azodicarboxylates, for ArOAm.

**Scheme 122:**
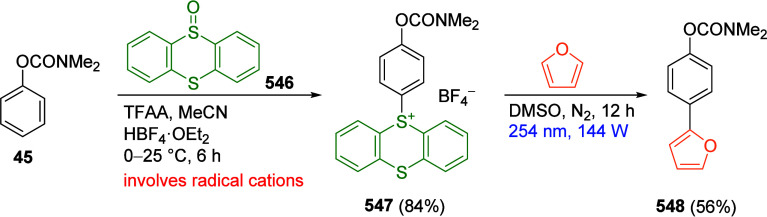
An Example of the Compatibility of the OAm Group for Thianthrenation under Radical Conditions (**45**→**547**) and Strong UV Light (**547**→**548**) Adapted from ref (469). Copyright 2019 Springer Nature. Adapted from ref (470). Copyright 2021 American Chemical Society.

Other avenues open for future exploration include more sustainable approaches to site-selective C–H activation such as those that utilize photoredox, electrochemistry, mechanochemistry, and flow processes.^420,468^ This may be enabled by the fact that the *O*-carbamate group is also tolerated by a variety of nonstandard reactive species and reaction conditions, including the radical cations proposed in the thianthrenation of phenyl dimethylcarbamate **45** with **546** to arylsulfonium salt **547** in 84% yield,^469^ as well as photoinduced arylation CC under strong UV light conditions giving **548** in 56% yield,^470^ as shown in Scheme 122.

## 11 ArOAm in D*o*M and A*o*F under Greener and More Sustainable Conditions

**Scheme 123:**
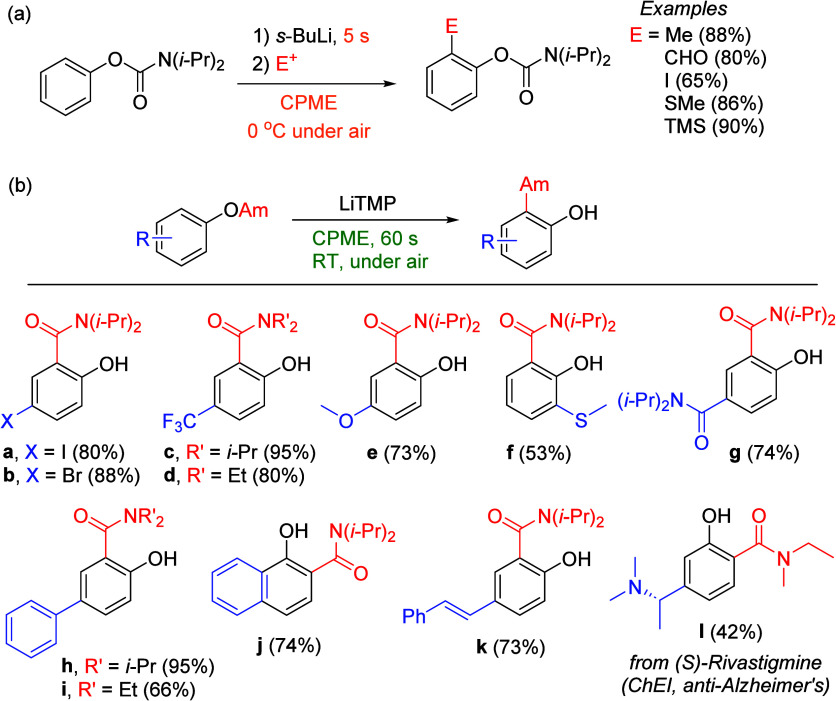
(a) Electrophilic *ortho*-Functionalization of *O*-Phenyl *N*,*N*-Diisopropylcarbamate under Aerobic Conditions ; (b) LiTMP-Promoted AoF Rearrangement of *O*-Aryl Carbamates in CPME under Aerobic Conditions. Reported Yields Refer to Isolated Products Adapted with permission from ref (484). Copyright 2022 John wiley and Sons.

To conform with trends in industry and academia,^471−476^ research has gone into the development of organometallic chemistry which uses fewer polluting reagents, less harsh conditions, and promotes the use of more sustainable and circular chemistry.^477−483^ Thus, far, regarding the D*o*M and A*o*F of aryl *O*-carbamates, this work has mainly focused on conducting the reactions under aerobic conditions using greener solvents. Ghinato et al.^484^ were the first to report efficient protocols for enabling D*o*M using and A*o*F of ArOAms with hindered lithium amide metalating agents, using cyclopentyl methyl ether (CPME) as sustainable reaction solvent^485^ under aerobic conditions. For example, when *O*-phenyl *N*,*N*-diisopropylcarbamate was treated with *s*-BuLi in CPME at 0 °C followed by quench (after 5 s) with various electrophilic reagents, *ortho*-functionalized adducts were obtained in good to excellent yields select examples are shown in Scheme 123a). A*o*F could be accomplished using LiTMP in CPME under air (Scheme 123b) for a variety of substrates (select examples shown): electron deficient (**a**–**d**), electron rich with competing DGs (**e**–**g**), polycyclic (**h**–**j**), substrates bearing a range of functional groups, e.g., double bonds (**k**), and pharmaceutical compounds, e.g., (*S*)-rivastigmine (**l**) in reasonable to very good yields. The choice of *N*-alky groups on the *O*-carbamate influenced the yield [Scheme 123(b), compare **c**/**d** and **h**/**i**], likely due to steric effects.

**Scheme 124:**
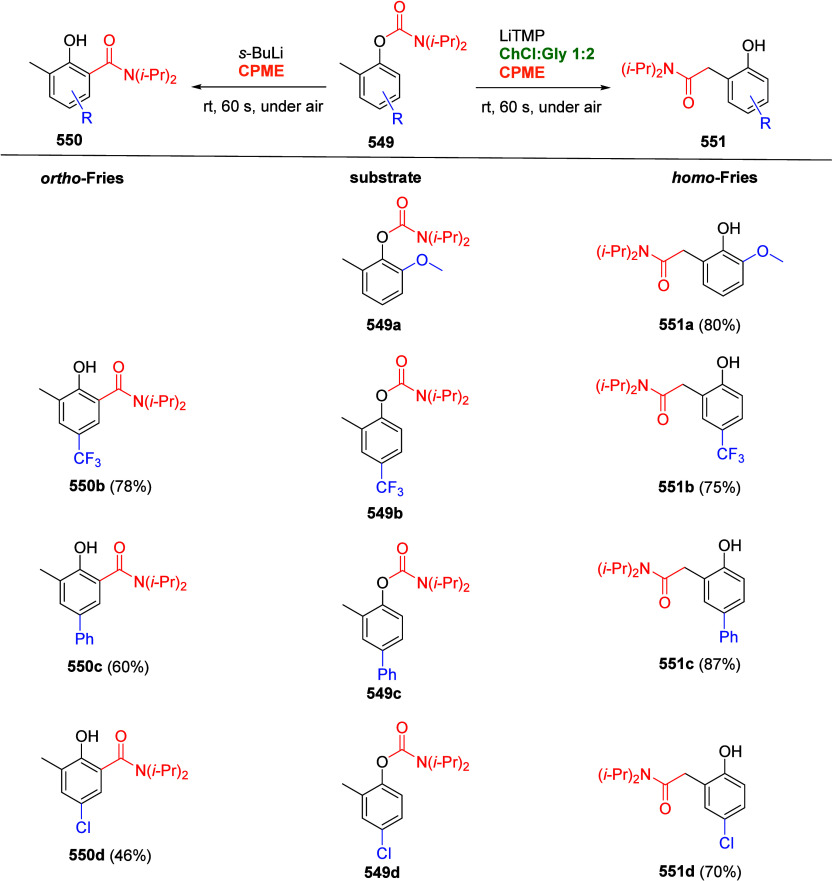
Regioselective *ortho*- vs *homo*-Fries Rearrangement of **549a**–**d** Adapted with permission from ref (484). Copyright 2022 John Wiley and Sons.

The authors then investigated *ortho*- versus *homo*-Fries rearrangements in *ortho*-tolyl *O*-carbamates to gain more insight into the regioselectivity of the metalation/migration sequence under aerobic conditions. In this case, a series of *ortho*-tolyl *N*,*N*-diisopropylcarbamates (**549**) decorated with different substituents, e.g., EDG (**a**), EWG (**b**), aryl (**c**), and halogenated (**d**), on the aromatic ring were subjected to one of two optimized metalation conditions: *s*-BuLi in CPME at room temperature and under air versus LiTMP in a heterogeneous mixture of CPME and deep eutectic solvents (DES) (in this case, a choline chloride (ChCl) and glycerol (Gly) mix) at room temperature and under air, to obtain either corresponding 1,2,3-contiguously substituted salicylamides (**550b**–**d**) or homologous α-arylacetamide derivatives **551a**–**d** with high regioselectivity (Scheme 124).

Metalation of **549** with *s*-BuLi in CPME (at room temperature and under air) favors the *ortho*-Fries rearrangement, likely driven by the relative stability of the corresponding phenoxide (described in detail by Miah et al.^85^), whereas using LiTMP in a CPME/DES mixture promotes the lateral lithiation/*homo*-Fries rearrangement reaction instead. This demonstrates the utility of eco-friendly solvents in causing A*o*F versus lateral rearrangements.

**Scheme 125:**
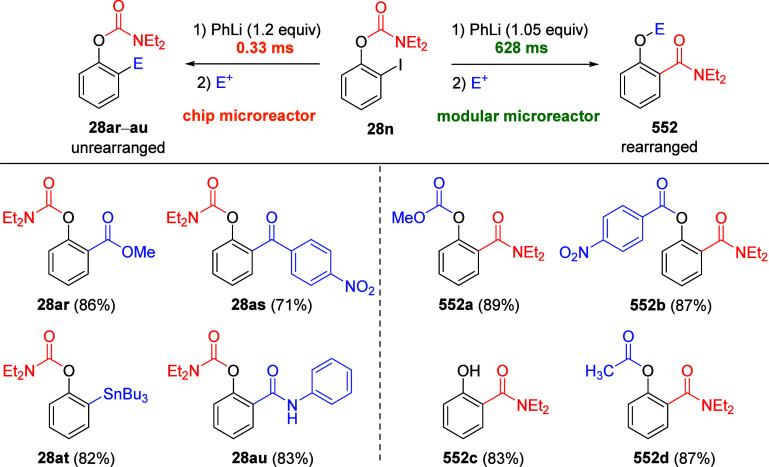
Chemoselective Functionalization of Iodophenyl Carbamates at the *ortho* Position (Unrearranged Products) Controlled by a Microfluidic Chip Microreactor That Outperforms Very Fast A*o*F Rearrangement (Rearranged Products) Adapted with permission from ref (488). Copyright 2016 The American Association for the Advancement of Science.

Flow chemistry is another approach that has been recognized to promote green and sustainable chemistry^486,487^ and, in recent times, has been extended to include metalation chemistry. For example, Kim et al.^488^ developed a microfluidic technique that outpaces the very rapid A*o*F rearrangement to chemoselectively functionalize iodophenyl carbamates at the *ortho* position, forming rearranged product **552** (Scheme 125). Central to the technique is a chip microreactor, which can shorten a reaction time in the submillisecond range, even at cryogenic temperatures.

Later, the same group reported improved control of the A*o*F reaction in a 3D-printed stainless steel microreactor with a circular fluidic channel of a few tenths of a micrometer in cross-section.^489^ They investigated the mixing efficiency of different channel configurations with different designs. The simple T-shaped channel structure showed superior synthetic yield of the unrearranged product at residence times of less than milliseconds, raising the prospect of upgrading microreactor technology for high-throughput production. Yoshida’s latest advances in this field encompass performing [1,4], [1,5], and [1,6] anionic Fries-type rearrangements using flow chemistry.^490^

## 12 Conclusions and Implications for Future Research

This review covers the main chemistry of the ArOAm group since Victor Snieckus’ seminal 1990 review.^21^ We focused on advances made with the D*o*M and D*re*M, A*o*F rearrangements, and C–H functionalization of aromatic compounds containing the *O*-carbamate group, including how accompanying silyl groups have been exploited as protecting groups as well as mediating both *ipso*- and non-*ipso*-substitutions. It was necessary to elaborate on possible manipulations and CC reactions of the *O*-carbamate group to highlight the utility of the ArOAm. We also included a comprehensive section highlighting practical applications of the ArOAm in the synthesis of various valuable bioactive compounds (section 9). Our work consolidates the many discoveries and developments made in the field over the past 30 years while exposing gaps in the literature and encouraging fresh research in these (and other) directions. Some ideas for research to address gaps in the literature are discussed as follows.

### 12.1 Metalation of ArOAm Using Alternative Metalating Agents

First, the development of alternative metalating reagents to what have already been tried (see section 2.3.4) is necessary for enabling less harsh conditions and to use alternative Group 1 and 2 metals, given the recent surge in demand for Li (in Li ion batteries).^491^ We point readers to a comprehensive 2022 review^492^ covering the latest “ate” complexes which might be explored. New metalating agents developed by the research group of Knochel^493,494^ (the creator of “TurboGrignards”) are also worth delving into.

### 12.2 Boron-Mediated Functionalization of ArOAms

We anticipate that ArOAms could form BBr_2_ or BF_2_ complexes in a similar fashion to what has already been described for phenylacetamides by Iqbal et al.^495^ and *N*-aryl amides by Shinde et al.^496^ These complexes could be utilized for further functionalization, e.g., halogenation. The BX_2_ group could also be tested as a coupling group with aryl halides via Suzuki coupling as has been demonstrated with potassium aryltrifluoroborates.^497,498^

### 12.3 CC of ArOAm

Although much has been achieved with developing chemistry enabling CC of the ArOAm group (see section 8), there are still some areas worth further study. These include the following:

(1) CC with aryl bromides: there are no examples of CC of fused *O*-carbamates with aryl bromides in the literature (only naphthyl *O*-carbamates) (see Scheme 60). This is an opportunity, and the prevalence of (hetero)aryl bromides implies that any methodology developed in this area would be of great value to various fields.
(2) CC of ArOAm with compounds containing boron-containing functional groups other than boronic esters such as BX2 (X = Br, F)^412,499^ would provide access to a diverse range of products.
(3) There is scope for investigating the CC of ArOAms with other FGs such as nitriles, organozincs, and organostannanes, as has been demonstrated with other phenol-derived electrophiles.^500−502^
(4) The lack of examples of CC between nonfused ArOAms and silylmagnesium reagents (during which the ArOAms are converted to aryl silanes, i.e., the process is silylation via C–O cleavage) hints at the difficulty of this procedure (outlined in section 8.9. above). Any solution to overcome this challenge would pay dividends.
(5) There is only one report of CC of alkyl-aluminum reagents with ArOAms (section 8.7). Thus, any advancement with this chemistry would provide opportunities for diversification of products using a relatively well-tolerated (and high yielding) procedure.
(6) Examples of CC incorporating green/sustainable chemistry have been investigated. In the future, more can be done to advance this area, especially with the current public motivation to convert from linear to circular chemistry.^503^ Related to this, the use of electrocatalysis, organocatalysis, photocatalysis (and combinations of these), as well as mechanochemistry, for the transformations of ArOAms, are worth future investigation. Thus, far, electrocatalysis has only been demonstrated for alkyne annulations via C–H/Het–H activation of aryl *O*-carbamates,^468^ while photocatalysis has been applied to facilitate the cycloamination of of prenyl *O*-carbamates and ureas.^504^ Reynes et al.^505^ reported mechanochemical nickel-catalyzed Suzuki–Miyaura coupling of aryl sulfamates.
(7) Although there are examples of CC of alkenyl O-carbamates (sections 8.2.2. and 8.2.4), progress made with CC of linear, cyclic, and annulated aliphatic and benzylic O-carbamates could unlock new, valuable new avenues of exploration.

### 12.4 Functionalization of 1,8-Naphthalenediyl Bis(diethylcarbamate)s

Apart from the previously mentioned 2,7-naphthalenediyl bis(diethylcarbamate) compounds (Table 8, Scheme 13), there is only one example in the literature of the functionalization of analogous 1,8-naphthalenediyl bis(diethylcarbamate) compounds using D*o*M.^206^ In this case, the metalated compounds were converted to 1,8-naphthalenediol derivatives which could be complexed with transition metals to form functional materials (e.g., capable of catalysis). The as-yet unexplored 2,7-arylation of 1,8-naphthalenediyl bis(diethylcarbamate)s followed by D*re*M or A*o*F of the 1,8-naphthalenediyl bis(diethylcarbamate) compounds could provide access to a new set of interesting materials. This has been carried out for the amide analogues.^506^

### 12.5 Broadening the Scope of Metalation Chemistry to Include Other Heteroaromatic *O*-Carbamates

Throughout this review, we have focused on the utilization of the OAm FG as a DMG in metalation chemistry in aryl and naphthyl substrates. Examples of heteroaryl compounds have appeared such as pyridyl and thiophene *O*-carbamates (e.g., see Scheme 35). However, there are several heteroaromatic *O*-carbamates which have *not* been functionalized using D*o*M chemistry. These include the following: furans, oxazoles, thiazoles, oxadiazoles, benzodioxoles, pyrazines, pyridazines, pyrimidines, and quinolines. The functionalization could involve metalation directed by an *O*-carbamate positioned on the N itself (restricted to heterocycles with an N–H, e.g., indole, imidazole). These heteroaromatic compounds are useful in many applications ranging from medicine and pharmaceutics^507−509^ to agriculture,^510^ catalysis,^511,512^ and new functional materials (e.g., in organic electronics);^513^ thus, further functionalization of these would be of interest.

### 12.6 Broadening the Scope of Metalation Chemistry to Include Alkenyl and Alkyl *O*-Carbamates

Another area of interest involves the extension of the metalation chemistry of ArOAms to alkenyl and alkyl *O*-carbamates, e.g., (hetero)benzylic compounds.^514,515^ Since this has not yet been reported, it is implicit that these systems are too unreactive. Nevertheless, the ready access to linear, cyclic, and annulated (e.g., adamantyl) aliphatic *O*-carbamates^516−518^ beckons a foray into this potentially rich field.

### 12.7 Further Exploration of Aryl Se- and S-Carbamates

Finally, as mentioned in Section 2.3.2, the metalation chemistry of aryl Se-carbamates, aryl S-carbamates, and aryl dithiocarbamates has hardly been explored. There is much research interest in developing new synthetic routes to dithiocarbamates (due to the importance of organosulfur compounds in biological compounds),^519^ but little investigation has taken place regarding the functionalization of these materials.

### 12.8 Comparison of ArOAm with Recently Reported Directing Groups

The introduction of DMGs such as triflones (trifluoromethyl sulfones),^520^ sulfonyl fluorides and fluorosulfates,^521^ aziridino groups,^522^ tetraethylphosphorodiamidate,^523^ phosphoric acids and *N*-triflylphosphoramides,^524^ sulfoximines,^525,526^ α-lithiobenzyloxy,^101^ THP-protected hydroxy group (OTHP),^126^ and small-ring heterocycles^527^ invites exploration into how these compare with (or how they might be used in tandem with) ArOAm for the synthesis of complex molecules.

In addition, there are still numerous functional groups yet to be discovered as directing groups for (hetero)aryl *ortho*-metalation, e.g., the *O*-protecting methoxyethoxymethyl ether (MEM), pivaloyl, and aryl glyoxal groups. These might be valuable given their facile transformational capabilities and wider utility.

### 12.9 ArOAm-Mediated Synthesis of Isotopically Labeled Compounds

The rising interest in isotopically labeled compounds (especially deuterated/tritiated pharmaceuticals) as therapeutic agents,^528,529^ bioanalytical standards^530,531^ as functional materials in organic electronic applications^532−535^ and in mechanistic studies^536,537^ has encouraged researchers to find new synthetic methodologies to these materials.^538,539^ However, there are scant examples where isotopologues have deliberately been prepared via *ortho* metalation chemistry.^540,541^ The utility of the ArOAm both as DMG (as described in this review) and within bioactive compounds containing ArOAm^6,11,509^ or compounds containing functionalities accessible from ArOAm, such as phenols,^542−544^ suggests that the ArOAm could be exploited more widely in this area, especially now that milder and greener/more sustainable reactions conditions are being developed (Section 11).

### 12.10 Final Word

Despite the progress made with developing the functionality of the ArOAm group, there are still numerous avenues worth exploration. These are especially significant given increased interest in converting readily available CO_2_ into organic carbamates^13,15,16,545−547^ and the functionalization of aromatic phenolic monomers obtained via the depolymerization of biomass-derived lignin.^548,549^ Furthermore, compounds containing the ArOAm functional group continue to be developed for a range of applications such as for potential Alzheimer’s disease treatments.^509,550−552^

## Dedication

^∥^ V.S. passed away in December 2020.

## Abbreviations

Acac: acetylacetone
AChE: acetyl cholinesterase
Am: amide
*t*-am: *tert*-amyl
A*o*F: anionic *ortho*-Fries
aq: aqueous
ArOAm: aryl *O*-carbamate
B_2_pin_2_: bis(catecholato)diboron
Bn: benzyl
Boc: *tert*-butyloxycarbonyl
Bpin: catecholatodiboron
bpy: 2,2′-bipyridine
BTPP: *tert*-butylimino-tri(pyrrolidino)phosphorane
*n*-BuLi: *n*-butyllithium
*s*-BuLi: *sec*-butyllithium
*t*-BuLi: *tert*-butyllithium
CC: cross coupling
CIPE: complex-induced proximity effect
COD: 1,5-cyclooctadiene
Cp*: pentamethylcyclopentadienyl
CPME: cyclopentyl methyl ether
Cy: cyclohexyl
DBU: 1,8-diazabicyclo[5.4.0]undec-7-ene
DCE: 1,2-dichloroethane
DCM: dichloromethane
dcype: 1,2-bis(dicyclohexylphosphino)ethane
dcypt: 3,4-bis(dicyclohexylphosphino)thiophene
DDQ: 2,3-dichloro-5,6-dicyano-1,4-benzoquinone
DES: deep eutectic solvent
DFT: density functional theory
DG: directing group
DIAD: diisopropyl azodicarboxylate
DIBAL: diisobutylaluminum hydride
DME: dimethyl ether
DMG: directed metalation group
DMPU: 1,3-dimethyl-3,4,5,6-tetrahydro-2-pyrimidinone
D*o*M: directed *ortho* metalation
D*re*M: directed remote metalation
dtbpy: 4,4′-di-*tert*-butyl-2,2′-bipyridine
E^+^: electrophile
EDG: electron donating group
EDTA: ethylenediaminetetraacetic acid
equiv: equivalent
EWG: electron withdrawing group
HalD: halogen dance
HF: Hartree–Fock
HFIP: hexafluoroisopropanol
HOESY: Heteronuclear Overhauser Effect Spectroscopy
HTMP: (2,2,6,6-Tetramethylpiperidine)
I*t*-Bu: 1,3-di-*tert*-butylimidazol-2-ylidene
KEM: kinetically enhanced metalation
LAH: lithium aluminum hydride
LDA: lithium diisopropylamide
LiTMP: lithium 2,2′,6,6′-tetramethylpiperidide
MNDO: modified neglect of diatomic overlap
MTBE: methyl *tert*-butyl ether
MW: microwave
NaDA: sodium diisopropylamide
NBS: *N*-bromosuccinimide
NCS: *N*-chlorosuccinimide
neo: neopentyl
NIS: *N*-iodosuccinimide
NKR: Newman–Kwart rearrangement
NMR: nuclear magnetic resonance
OAm: *O*-carbamate
OMEM: 2-methoxyethoxymethyl ether
OMOM: 2-methoxymethyl ether
OTHP: tetrahydropyranyl ether
Piv: pivalic acid
PMDTA: *N*,*N*,*N*′,*N*″,*N*″-pentamethyldiethylenetriamine
Py: pyridine
Pym: 2-pyrimidyl
SET: single electron transfer
SIPr: 1,3-bis(2,6-diisopropylphenyl)imidazolinium
SMAP: silicon constrained monodentate trialkylphosphine
TBAB: tetra-*n*-butylammonium bromide
TBAF: tetra-*n*-butylammonium fluoride
TBS: *tert*-butyldimethylsilyl
TES: triethylsilyl
Tf: trifluoromethanesulfonate, triflate
TFA: trifluoroacetic acid
TFAA: trifluoroacetic anhydride
THF: tetrahydrofuran
TIPS: triisopropylsilyl
TMAF: tetramethylammonium fluoride
TMDSO: 1,1,3,3-tetramethyldisiloxane
TMEDA: tetramethylethylenediamine
TMP: 2,2′,6,6′-tetramethylpiperidine
TMS: trimethylsilyl
TRIP: triptycene
Ts: tosyl
